# Supplementary material for: Prospective association between adherence to the Mediterranean diet and hepatic steatosis: the Swiss CoLaus cohort study
Source: BMJ Open. 2020 Dec 22;10(12):e040959. doi: 10.1136/bmjopen-2020-040959 (PMC7757450; doi:10.1136/bmjopen-2020-040959)
Supplement: Supplementary data [file bmjopen-2020-040959supp001.pdf]

Supplementary file

Title: The association between adherence to the Mediterranean diet and hepatic steatosis: the Swiss CoLaus prospective cohort study

Table of Content

Figure S1 Sample selection flow chart. 3

Table S1 Characteristics of the participants included and excluded from the analysis, CoLaus study, Switzerland. 4

Table S2 Baseline characteristics of the participants according to the risk of hepatic steatosis defined by the fatty liver index or the NAFLD-score, CoLaus study, Switzerland. 5

Table S3 Prospective association of the Mediterranean diet score with the risk of hepatic steatosis, CoLaus study, Switzerland: assessment of influence of other potential covariates. 6

Table S4 Sensitivity analyses for the associations of the Mediterranean diet score with the risk of hepatic steatosis, CoLaus study, Switzerland. 8

Table S5 Prospective association of the Mediterranean diet score with the risk of hepatic steatosis, CoLaus study, Switzerland: sensitivity analysis while excluding participants with only one of the indices high at baseline and using hepatic steatosis index as an alternative score. 11

Table S6 Prospective association of the Mediterranean diet score with the risk of hepatic steatosis, CoLaus study, Switzerland (n=1632), Sensitivity analysis while excluding participants with either indices high (using FLI>30 instead of FLI≥60). 13

Table S7 Association of the Mediterranean diet score with change in BMI and waist circumference, CoLaus study, Switzerland. 14

Table S8 Association of the Mediterranean diet score with the GGT, ALT, and AST, CoLaus study, Switzerland. 15

Figure S1 Sample selection flow chart.

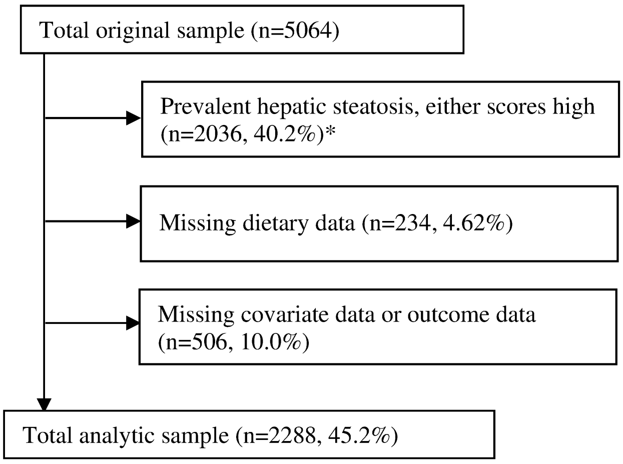

* Prevalent hepatic steatosis was defined as having either fatty liver index≥ 60 OR non-alcoholic fatty liver disease fatty liver score≥ -0.640.

Table S1 Characteristics of the participants included and excluded from the analysis, CoLaus study, Switzerland.

Characteristic	Included (n=2288)	Excluded (n=2776)	*P*-value*
Age, *years*	55.8±10.0	59.4±10.6	<0.001
Women (%)	65.4	43.6	<0.001
Marital status (%)†			0.038
Single	16.5	14.0	
Married/cohabiting	56.6	57.4	
Widowed/separated/divorced	26.8	28.6	
Employed (%)	63.5	50.4	<0.001
Education (%)†			<0.001
University	25.9	17.5	
High school	28.5	23.6	
Apprenticeship	33.2	37.4	
Mandatory education	12.5	21.4	
Current smoker (%)	20.1	23.1	<0.001
Alcohol consumption (%)†‡			<0.001
Abstainers	23.5	26.8	
Moderate	69.3	63.3	
Heavy	7.2	9.9	
Total energy intake (kcal/d)	1807±673	1853±784	0.033
Total protein (% energy)	15.4±3.3	15.6±3.5	0.015
Total carbohydrate (% energy)	47.0±8.6	45.4±9.1	<0.001
Total fat (% energy)	34.3±6.7	34.4±6.9	0.54
TEE (kcal/d)	2575±586	2790±669	<0.001
Metabolic syndrome (%)§	10.8	60.9	<0.001
BMI (kg/m^2)	23.7±2.8	28.3±4.8	<0.001
Waist circumference (cm)	84.4±8.9	98.3±12.5	<0.001
Triglycerides (mmol/l)	1.0±0.5	1.6±1.1	<0.001
median (iqr)	0.9 (0.7, 1.2)	1.4 (1.0, 2.0)	
GGT (U/l)	23.8±16.0	48.8±60.8	<0.001
median (iqr)	19 (14, 27)	32 (21, 53)	
≥50 (%)	5.6	27.5	<0.001
ALT (U/l)	21.8±8.8	32.5±20.7	<0.001
median (iqr)	20 (16, 25)	27 (20, 38)	
≥40 (%)	5.0	23.3	<0.001
AST (U/l)	25.9±6.2	31.6±14.1	<0.001
median (iqr)	25 (22, 29)	28 (24, 34)	
≥37 (%)	5.6	20.4	<0.001

Abbreviations: TEE, total energy expenditure; BMI, body mass index; iqr, interquartile range; GGT, gamma-glutamyl transferase; ALT, alanine aminotransferase; AST, aspartate aminotransferase.

Data are mean ± SD for continuous variables or percent for categorical variables, unless otherwise stated.

* P-value calculated using Chi-square test for categorical variables and student's t-test for continuous variables.

† Due to some missing data, percentages do not always add to 100%.

‡ Alcohol consumption categorized as "abstainers" (0 unit/week), "moderate" (1–21 units/week for men, 1–14 for women), and "heavy drinkers" (>21 units/week for men, >14 for women).

§ Metabolic syndrome defined according to the International Diabetic Federation (waist circumference ≥94 cm in men and ≥80 cm in women plus at least two of the following factors: serum triglycerides≥1.70 mmol/L or specific treatment for this lipid abnormality; serum high-density lipoprotein cholesterol <1.03 mmol/L in men and <1.29 mmol/L in women or specific treatment for this lipid abnormality; systolic blood pressure ≥130 mmHg or diastolic blood pressure ≥85 mmHg or treatment for previously diagnosed hypertension; and fasting plasma glucose ≥5.6 mmol/L or previously diagnosed type 2 diabetes).

Khalatbari-Soltani S, *et al. BMJ Open* 2020; 10:e040959. doi: 10.1136/bmjopen-2020-040959

Table S2 Baseline characteristics of the participants according to the risk of hepatic steatosis defined by the fatty liver index or the NAFLD-score, CoLaus study, Switzerland.

	Risk of hepatic steatosis					
	FLI			NAFLD-score		
Characteristic	No (n=2135)	Yes (n=153)	*P*-value*	No (n=2080)	Yes (n=208)	*P*-value*
Age, *years*	55.8±10.1	54.9±9.5	0.29	55.6±10	57.7±10.1	0.003
Women (%)	66.8	45.8	<0.001	66.7	52.9	<0.001
Marital status (%)†			0.86			0.79
Single	16.6	15.0		16.7	14.9	
Married/cohabiting	56.5	58.2		56.5	58.2	
Widowed/Separated/divorced	26.8	26.8		26.8	26.9	
Employed (%)	63.3	66.0	0.50	64.5	53.8	0.002
Education (%)†			0.013			0.33
University	26.7	15.0		26.4	20.7	
High school	28.3	30.1		28.3	29.8	
Apprenticeship	32.6	40.5		32.8	36.5	
Mandatory education	12.3	14.4		12.4	13.0	
Current smoker (%)	19.7	25.5	0.006	20.3	17.8	0.52
Alcohol consumption (%)†‡			0.15			0.001
Abstainers	23.3	26.1		22.5	33.7	
Moderate	69.7	63.4		70.4	58.7	
Heavy	6.9	10.5		7.1	7.7	
Total energy intake (kcal/d)	1800±655	1906±879	0.062	1794±654	1938±833	0.003
Protein (% energy)	15.3±3.1	16.1±5.0	0.006	15.3±3.2	16.0±4.1	0.006
Carbohydrate (% energy)	47.1±8.5	45.6±10.1	0.037	47.0±8.5	46.5±9.5	0.36
Fat (% energy)	34.3±6.7	33.8±7.2	0.37	34.2±6.7	34.4±6.6	0.69
TEE (kcal/d)	2552±572	2912±677	<0.001	2562±582	2699±616	0.002
Metabolic syndrome (%)§	9.9	22.9	<0.001	9.8	21.2	<0.001
BMI (kg/m^2)	23.5±2.7	26.8±2.6	<0.001	23.5±2.8	26.0±2.5	<0.001
Waist circumference (cm)	83.8±8.7	93.4±7.0	<0.001	83.7±8.7	92.0±7.4	<0.001
Triglycerides (mmol/l)	1.0±0.5	1.2±0.5	<0.001	1.0±0.5	1.2±0.5	<0.001
median (iqr)	0.9 (0.7, 1.2)	1.1 (0.7, 1.2)		0.9 (0.7, 1.2)	1.1 (0.8, 1.4)	
GGT (U/l)	23.1±15.2	32.4±22.2	<0.001	23.3±15.4	28.1±20.6	<0.001
median (iqr)	19 (14, 26)	26 (18, 40)		19 (14, 26)	22 (15, 34)	
≥50 (%)	4.9	15.0	<0.001	5.3	9.1	0.022
ALT (U/l)	21.6±8.8	23.5±8.9	0.013	21.3±8.4	26.0±11.6	<0.001
median (iqr)	19 (16, 25)	21 (17, 28)		19 (16, 25)	23 (18, 30)	
≥40 (%)	5.0	5.9	0.63	4.5	10.6	<0.001
AST (U/l)	25.9±6.2	26.3±6.3	0.50	25.9±6.2	26.9±6.2	0.026
median (iqr)	25 (22, 29)	25 (22, 29)		25 (22, 29)	26 (22, 30)	
≥37 (%)	5.5	7.2	0.39	5.3	8.2	0.096

Abbreviations: TEE, total energy expenditure; FLI, fatty liver index; NAFLD, non-alcoholic fatty liver disease; BMI, body mass index; iqr, interquartile range; GGT, gamma-glutamyl transferase; ALT, alanine aminotransferase; AST, aspartate Aminotransferase.

Data are mean ± SD for continuous variables or percent for categorical variables, unless otherwise stated.

* P-value calculated using Chi-square test for categorical variables and student's t-test for continuous variables.

† Due to some missing data, percentages do not always add to 100%.

‡ Alcohol consumption categorized as "abstainers" (0 unit/week), "moderate" (1–21 units/week for men, 1–14 for women), and "heavy drinkers" (>21 units/week for men, >14 for women).

§ Metabolic syndrome defined according to the International Diabetic Federation (waist circumference ≥94 cm in men and ≥80 cm in women plus at least two of the following factors: serum triglycerides≥1.70 mmol/L or specific treatment for this lipid abnormality; serum high-density lipoprotein cholesterol <1.03 mmol/L in men and <1.29 mmol/L in women or specific treatment for this lipid abnormality; systolic blood pressure ≥130 mm Hg or diastolic blood pressure ≥85 mm Hg or treatment for previously diagnosed hypertension; and fasting plasma glucose ≥5.6 mmol/L or previously diagnosed type 2 diabetes.

Table S3 Prospective association of the Mediterranean diet score with the risk of hepatic steatosis, CoLaus study, Switzerland: assessment of influence of other potential covariates.

	Risk ratio (95% CI) across quintiles of Mediterranean diet score*					*P*-trend	Risk ratio (95% CI) Per SD increase*
	Q1	Q2	Q3	Q4	Q5		
Range	1.83-7.63	7.64-8.35	8.36-8.92	8.93-9.59	9.60-12.1		
N total	458	458	457	458	457		
Fatty liver index†							
Different models							
N Cases (score≥60)	36	43	35	22	17		
Multivariable (Model 1)‡	1.00 (ref.)	1.11 (0.72, 1.72)	1.07 (0.68, 1.66)	0.71 (0.42, 1.22)	0.50 (0.28, 0.91)	0.006	0.84 (0.71, 0.99)
Model 1 + Changes in BMI categories§	1.00 (ref.)	1.11 (0.73, 1.69)	0.98 (0.64, 1.50)	0.73 (0.43, 1.24)	0.59 (0.32, 1.06)	0.024	0.85 (0.72, 1.02)
Model 1 + Alcohol	1.00 (ref.)	1.11 (0.72, 1.72)	1.06 (0.69, 1.65)	0.71 (0.41, 1.22)	0.50 (0.28, 0.91)	0.006	0.84 (0.70, 0.99)
Model 1 + Alcohol+ BMI‖	1.00 (ref.)	1.13 (0.74, 1.73)	0.92 (0.59, 1.42)	0.76 (0.45, 1.29)	0.63 (0.35, 1.14)	0.047	0.86 (0.72, 1.04)
Model 1 + Clinical variables**	1.00 (ref.)	1.30 (0.80, 2.11)	1.20 (0.74, 1.94)	0.77 (0.43, 1.39)	0.57 (0.30, 1.09)	0.023	0.84 (0.70, 1.01)
Model 1 + Clinical variables + BMI	1.00 (ref.)	1.28 (0.79, 2.07)	0.94 (0.58, 1.52)	0.80 (0.45, 1.41)	0.68 (0.36, 1.27)	0.076	0.86 (0.71, 1.04)
Model 1 + MetS††	1.00 (ref.)	1.14 (0.74, 1.75)	1.03 (0.66, 1.60)	0.68 (0.40, 1.16)	0.51 (0.28, 0.93)	0.005	0.83 (0.70, 0.99)
NAFLD-score‡‡							
Different models							
N Cases (score≥-0.640)	41	46	51	38	32		
Multivariable (Model 1)‡	1.00 (ref.)	1.13 (0.75, 1.70)	1.26 (0.85, 1.87)	1.01 (0.65, 1.56)	0.80 (0.50, 1.28)	0.28	0.95 (0.82, 1.10)
Model 1 + Changes in BMI categories§	1.00 (ref.)	1.13 (0.76, 1.69)	1.18 (0.80, 1.74)	1.04 (0.68, 1.60)	0.93 (0.58, 1.48)	0.65	0.99 (0.86, 1.16)
Model 1 + Alcohol	1.00 (ref.)	1.11 (0.73, 1.67)	1.22 (0.82, 1.82)	0.97 (0.63, 1.51)	0.77 (0.48, 1.23)	0.21	0.94 (0.81, 1.09)
Model 1 + Alcohol+ BMI‖	1.00 (ref.)	1.16 (0.78, 1.73)	1.15 (0.78, 1.68)	1.04 (0.68, 1.61)	0.93 (0.58, 1.48)	0.62	0.99 (0.85, 1.15)
Model 1 + Clinical variables**	1.00 (ref.)	1.20 (0.78, 1.84)	1.32 (0.88, 1.98)	0.99 (0.64, 1.55)	0.85 (0.52, 1.37)	0.33	0.95 (0.82, 1.11)
Model 1 + Clinical variables + BMI	1.00 (ref.)	1.21 (0.80, 1.84)	1.19 (0.80, 1.78)	1.06 (0.69, 1.64)	0.99 (0.61, 1.60)	0.76	0.99 (0.85, 1.16)

Abbreviations: SES, socio-economic status; BMI, body mass index; MetS, metabolic syndrome; NAFLD, non-alcoholic fatty liver disease; SD, standard deviation; CI, confidence interval.

Statistical analysis using Poisson regression with robust standard errors; results are expressed as risk ratios and (95% confidence intervals).

* In categorical analysis, the population was divided into five groups by quintiles (Q1-Q5) of the Mediterranean diet score; Standard deviation was 1.20 for different multivariable analyses.

† Calculated based on an algorithm including BMI, waist circumference, triglycerides, and gamma-glutamyl transferase.

‡ Adjusted for age (years), sex, marital status (single, married/cohabitant, and widowed/Separated/divorced), occupational status (working and not working), education level (university, high school, apprenticeship, and mandatory education), smoking status (never, former, and current), energy intake (kcal/day), total energy expenditure (kcal/day), and date of dietary assessment.

§ Changes in BMI categories defined as individuals with a normal BMI at baseline and follow-up, individuals with normal BMI at baseline and overweight or obese BMI at follow-up, individuals with overweight or obese BMI at baseline and at follow-up, or individuals with overweight or obese BMI at baseline and normal at follow-up.

‖ Results of further adjustment for waist circumference were broadly in line with of the further adjustment for BMI (data not shown).

**Family history of diabetes (yes/no), high blood pressure (yes/no), high triglyceride level (yes/no), low HDL level (yes/no), and high glucose level (yes/no).

†† Metabolic syndrome as defined by the International Diabetes Federation (yes/no).

‡‡ Calculated based on an algorithm including presence of the metabolic syndrome and type 2 diabetes, and concentrations of fasting serum insulin, fasting serum aspartate-aminotransferase (AST), and the AST/alanine-aminotransferase ratio.

Table S4 Sensitivity analyses for the associations of the Mediterranean diet score with the risk of hepatic steatosis, CoLaus study, Switzerland.

	Risk ratio (95% CI) across quintiles of Mediterranean diet score*						
	Q1	Q2	Q3	Q4	Q5	*P*-trend	Risk ratio (95% CI) Per SD increase*
Range	1.83-7.63	7.64-8.35	8.36-8.92	8.93-9.59	9.60-12.1		
N total	458	458	457	458	457		
Fatty liver index†							
Different models							
N Cases (score≥60)	36	43	35	22	17		
Multivariable (Model 1)‡	1.00 (ref.)	1.11 (0.72, 1.72)	1.07 (0.68, 1.66)	0.71 (0.42, 1.22)	0.50 (0.28, 0.91)	0.006	0.84 (0.71, 0.99)
Excluding alcohol from MDS component							
Model 1 + Alcohol	1.00 (ref.)	1.64 (1.04, 2.58)	1.08 (0.65, 1.80)	1.17 (0.70, 1.96)	0.65 (0.35, 1.18)	0.069	0.86 (0.73, 1.03)
Model 1 + Alcohol + BMI§	1.00 (ref.)	1.53 (0.98, 2.39)	0.94 (0.57, 1.54)	1.08 (0.67, 1.77)	0.75 (0.41, 1.35)	0.15	0.89 (0.75, 1.06)
Excluding participants with BMI≥30‖							
N Cases (score≥60)	35	40	28	19	16		
Model 1	1.00 (ref.)	1.08 (0.69, 1.68)	0.90 (0.56, 1.46)	0.64 (0.36, 1.12)	0.50 (0.27, 0.93)	0.005	0.83 (0.69, 0.98)
Model 1 + BMI§	1.00 (ref.)	1.02 (0.66, 1.58)	0.84 (0.53, 1.33)	0.65 (0.37, 1.14)	0.60 (0.33, 1.10)	0.031	0.85 (0.70, 1.02)
Excluding participants with excessive alcohol consumption**							
N Cases (score≥60)	33	42	34	21	17		
Model 1	1.00 (ref.)	1.17 (0.75, 1.84)	1.12 (0.71, 1.77)	0.70 (0.40, 1.23)	0.52 (0.28, 0.95)	0.007	0.82 (0.69, 0.98)
Model 1 + BMI§	1.00 (ref.)	1.17 (0.76, 1.81)	0.91 (0.58, 1.42)	0.73 (0.42, 1.24)	0.64 (0.35, 1.15)	0.037	0.85 (0.71, 1.02)
Excluding participants with implausible energy intake††							
N Cases (score≥60)	33	41	33	22	16		
Model 1	1.00 (ref.)	1.19 (0.63, 2.23)	1.16 (0.61, 2.20)	0.87 (0.45, 1.69)	0.69 (0.35, 1.38)	0.16	0.85 (0.71, 1.01)
Model 1 + BMI§	1.00 (ref.)	1.13 (0.60, 2.13)	0.90 (0.47, 1.73)	0.81 (0.43, 1.53)	0.74 (0.37, 1.49)	0.20	0.86 (0.72, 1.04)
Excluding participants with secondary causes of hepatic steatosis‡‡							
N Cases (score≥60)	37	42	35	22	17		
Model 1	1.00 (ref.)	1.18 (0.75, 1.85)	1.11 (0.70, 1.76)	0.78 (0.45, 1.34)	0.51 (0.27, 0.94)	0.010	0.85 (0.71, 1.01)
Model 1 + BMI§	1.00 (ref.)	1.17 (0.75, 1.83)	0.95 (0.60, 1.50)	0.78 (0.46, 1.31)	0.63 (0.34, 1.17)	0.050	0.86 (0.72, 1.04)
Excluding participants with diabetes§§							
N Cases (score≥60)	35	40	35	22	16		
Model 1	1.00 (ref.)	1.06 (0.67, 1.66)	1.11 (0.71, 1.73)	0.74 (0.43, 1.27)	0.49 (0.26, 0.91)	0.009	0.84 (0.71, 0.998)
Model 1 + BMI§	1.00 (ref.)	1.06 (0.69, 1.65)	0.92 (0.59, 1.43)	0.76 (0.45, 1.29)	0.58 (0.32, 1.06)	0.033	0.85 (0.71, 1.02)
NAFLD-score ¶¶							
Different models							
N Cases (score≥-0.640)	41	46	51	38	32		
Multivariable (Model 1)‡	1.00 (ref.)	1.13 (0.75, 1.70)	1.26 (0.85, 1.87)	1.01 (0.65, 1.56)	0.80 (0.50, 1.28)	0.28	0.95 (0.82, 1.10)

Excluding alcohol from MD component							
Model 1 + Alcohol	1.00 (ref.)	1.15 (0.78, 1.71)	0.92 (0.61, 1.41)	0.92 (0.59, 1.42)	0.89 (0.57, 1.38)	0.34	0.93 (0.81, 1.08)
Model 1 + Alcohol + BMI§	1.00 (ref.)	1.15 (0.78, 1.69)	0.88 (0.59, 1.32)	0.88 (0.57, 1.35)	1.02 (0.66, 1.57)	0.65	0.96 (0.83, 1.12)
Excluding participants with BMI≥30‖							
N Cases (score≥-0.640)	40	46	44	36	31		
Model 1	1.00 (ref.)	1.15 (0.76, 1.74)	1.12 (0.74, 1.69)	0.99 (0.63, 1.55)	0.81 (0.5, 1.30)	0.27	0.94 (0.81, 1.09)
Model 1 + BMI§	1.00 (ref.)	1.17 (0.78, 1.75)	1.09 (0.73, 1.63)	1.07 (0.69, 1.67)	0.97 (0.6, 1.55)	0.76	0.99 (0.85, 1.15)
Excluding participants with excessive alcohol consumption**							
N Cases (score≥-0.640)	38	43	50	37	32		
Model 1	1.00 (ref.)	1.10 (0.73, 1.68)	1.28 (0.85, 1.91)	1.00 (0.64, 1.56)	0.80 (0.5, 1.29)	0.32	0.96 (0.83, 1.11)
Model 1 + BMI§	1.00 (ref.)	1.15 (0.76, 1.74)	1.18 (0.80, 1.73)	1.06 (0.69, 1.65)	0.96 (0.6, 1.54)	0.75	1.00 (0.86, 1.17)
Excluding participants with implausible energy intake††							
N Cases (score≥-0.640)	39	46	48	38	30		
Model 1	1.00 (ref.)	1.32 (0.75, 2.35)	1.48 (0.84, 2.60)	1.06 (0.59, 1.91)	1.15 (0.64, 2.07)	0.92	0.95 (0.82, 1.10)
Model 1 + BMI§	1.00 (ref.)	1.34 (0.76, 2.38)	1.35 (0.77, 2.37)	1.06 (0.59, 1.89)	1.29 (0.72, 2.33)	0.67	0.99 (0.85, 1.16)
Excluding participants with secondary causes of hepatic steatosis‡‡							
N Cases (score≥-0.640)	41	46	50	37	31		
Model 1	1.00 (ref.)	1.19 (0.79, 1.81)	1.26 (0.84, 1.89)	1.06 (0.68, 1.65)	0.78 (0.48, 1.27)	0.25	0.95 (0.82, 1.10)
Model 1 + BMI§	1.00 (ref.)	1.23 (0.82, 1.85)	1.17 (0.79, 1.73)	1.11 (0.71, 1.72)	0.93 (0.57, 1.52)	0.66	0.99 (0.85, 1.16)
Excluding participants with diabetes§§							
N Cases (score≥-0.640)	37	43	50	36	30		
Model 1	1.00 (ref.)	1.17 (0.76, 1.79)	1.37 (0.90, 2.06)	1.05 (0.67, 1.66)	0.85 (0.52, 1.39)	0.43	0.97 (0.83, 1.12)
Model 1 + BMI§	1.00 (ref.)	1.21 (0.79, 1.85)	1.26 (0.85, 1.87)	1.11 (0.71, 1.75)	1.01 (0.62, 1.63)	0.89	1.01 (0.86, 1.18)

Abbreviations: SES, socio economic status; BMI, body mass index; MD, Mediterranean Diet; NAFLD, non-alcoholic fatty liver disease; SD, standard deviation; CI, confidence interval.

Statistical analysis using Poisson regression with robust standard errors; results are expressed as risk ratios and (95% confidence intervals).

* In categorical analysis, the population was divided into five groups by quintiles (Q1-Q5) of the Mediterranean diet score; Standard deviation was 1.41 after excluding alcohol from Mediterranean Diet score components, 1.20 after excluding participants with BMI≥30, 1.18 after excluding participants with excessive alcohol consumption, 1.20 after excluding participants with probable implausible energy intake or after excluding participants with secondary causes of hepatic steatosis or diabetes.

† Calculated based on an algorithm including BMI, waist circumference, triglycerides, and gamma-glutamyl transferase.

‡ Adjusted for age (years), sex, marital status (single, married/cohabitant, and widowed/Separated/divorced), occupational status (working and not working), education level (university, high school, apprenticeship, and mandatory education), smoking status (never, former, and current), energy intake (kcal/day), total energy expenditure (kcal/day), and date of dietary assessment.

§ Results of further adjustment for waist circumference were broadly in line with of the further adjustment for BMI (data not shown).

‖ Excluded 36 participants with BMI≥30 kg/m^2.

** Excessive alcohol consumption defined as >21 units per week for men and >14 units per weeks for women; excluded 60 participants with excess alcohol consumption (n=2228).

†† Implausible energy intake defined as <500 and <800 kcal or >3,500 or >4,000 kcal in women and men, respectively; excluded 41 participants with probable implausible energy intake (n=2247).

‡‡ Secondary causes of hepatic steatosis defined as having hepatitis B, C or HIV, and with hepatotoxic medications (Glucocorticoids, isoniazid, methotrexate, amiodarone, and tamoxifen); excluded 36 participants with probable secondary causes of hepatic steatosis (n=2252).
§§ Diabetes defined as glycated haemoglobin≥48 mmol/mol, or fasting plasma glucose ≥7.0 mmol/L, or use of hypoglycaemic drugs or insulin; excluded 26 participants with probable secondary causes of hepatic steatosis (n=2262).
¶¶ Calculated based on an algorithm including presence of the metabolic syndrome and type 2 diabetes, and concentrations of fasting serum insulin, fasting serum aspartate-aminotransferase (AST), and the AST/alanine-aminotransferase ratio.

Khalatbari-Soltani S, *et al. BMJ Open* 2020; 10:e040959. doi: 10.1136/bmjopen-2020-040959

Table S5 Prospective association of the Mediterranean diet score with the risk of hepatic steatosis, CoLaus study, Switzerland: sensitivity analysis while excluding participants with only one of the indices high at baseline and using hepatic steatosis index as an alternative score.

	Risk ratio (95% CI) across quintiles of Mediterranean diet score*					*P*-trend	Risk ratio (95% CI) Per SD increase *
	Q1	Q2	Q3	Q4	Q5		
range	1.83-7.64	7.64-8.34	8.35-8.92	8.93-9.57	9.58-12.18		
N total (n=2652)	531	530	531	530	530		
Fatty liver index, *median* (iqr)†	23.4 (11.1, 41.4)	24.1 (10.6, 44.7)	22.3 (9.9, 39.7)	20.2 (8.4, 38.4)	17.5 (7.3, 34.5)		
N cases (score≥60)	51	56	54	35	24		
Unadjusted	1.00 (ref.)	1.10 (0.77, 1.58)	1.06 (0.74, 1.52)	0.69 (0.45, 1.04)	0.47 (0.29, 0.75)	<0.001	0.79 (0.69, 0.89)
Multivariable ‡	1.00 (ref.)	1.12 (0.77, 1.63)	1.25 (0.86, 1.81)	0.88 (0.57, 1.35)	0.56 (0.34, 0.92)	0.011	0.86 (0.74, 0.99)
Multivariable + BMI	1.00 (ref.)	1.08 (0.75, 1.54)	0.99 (0.70, 1.41)	0.85 (0.56, 1.30)	0.59 (0.36, 0.96)	0.017	0.84 (0.73, 0.98)
Multivariable + BMI & WC	1.00 (ref.)	1.01 (0.71, 1.45)	0.99 (0.70, 1.40)	0.84 (0.56, 1.28)	0.58 (0.35, 0.95)	0.019	0.84 (0.72, 0.98)
range	1.83-7.56	7.57-8.29	8.30-8.88	8.89-9.53	9.54-12.12		
N total (n=2568)	514	514	513	514	513		
NAFLD-score, *median* (iqr)§	-1.9 (-2.4, -1.3)	-1.8 (-2.3, -1.2)	-1.9 (-2.4, -1.3)	-2.0 (-2.5, -1.3)	-2.1 (-2.5, -1.6)		
N cases (score≥-0.640)	63	70	67	59	38		
Unadjusted	1.00 (ref.)	1.11 (0.81, 1.53)	1.07 (0.77, 1.47)	0.94 (0.67, 1.31)	0.60 (0.41, 0.89)	0.006	0.88 (0.79, 0.99)
Multivariable ‡	1.00 (ref.)	1.16 (0.83, 1.62)	1.16 (0.83, 1.61)	1.02 (0.72, 1.46)	0.66 (0.44, 1.00)	0.039	0.92 (0.81, 1.04)
Multivariable + BMI	1.00 (ref.)	1.27 (0.91, 1.76)	1.24 (0.89, 1.72)	1.16 (0.81, 1.65)	0.84 (0.55, 1.28)	0.34	0.98 (0.87, 1.11)
Multivariable + BMI & WC	1.00 (ref.)	1.25 (0.90, 1.73)	1.24 (0.89, 1.72)	1.16 (0.82, 1.65)	0.83 (0.54, 1.27)	0.34	0.99 (0.87, 1.12)
range	1.83-7.15	7.16-8.07	8.08-8.76	8.77-9.47	9.48-12.18		
N total (n=2351)	471	470	470	470	470		
Hepatic steatosis index, *median* (iqr)†	32.4 (30.5, 34.4)	32.3 (30.3, 34.6)	32.5 (30.4, 34.5)	32.6 (30.6, 34.4)	32.4 (30.3, 34.1)		
N cases (score>36)	166	123	120	120	103		
Unadjusted	1.00 (ref.)	0.74 (0.61, 0.90)	0.72 (0.59, 0.88)	0.72 (0.59, 0.88)	0.62 (0.50, 0.76)	<0.001	0.85 (0.79, 0.90)
Multivariable ‡	1.00 (ref.)	0.77 (0.61, 0.96)	0.79 (0.63, 1.00)	0.83 (0.65, 1.04)	0.70 (0.55, 0.91)	0.070	0.90 (0.82, 0.98)

Abbreviations: iqr, interquartile range; SES, socio-economic status; BMI, body mass index; WC, waist circumference; NAFLD, non-alcoholic fatty liver disease.
Statistical analysis using Poisson regression with robust standard errors; results are expressed as risk ratios and (95% confidence intervals).
* In categorical analysis, the population was divided into five groups by quintiles (Q1-Q5) of the Mediterranean diet score; Standard deviation was 1.21 for the multivariable analyses.
† Calculated based on an algorithm including BMI, waist circumference, triglycerides, and gamma-glutamyl transferase.
‡ Adjusted for age (years), sex, marital status (single, married/cohabiting, and widowed/Separated/divorced), occupational status (working and not working), education level (university, high school, apprenticeship, and mandatory education), smoking status (never, former, and current), energy intake (kcal/day), total energy expenditure (kcal/day), and date of dietary assessment.

Khalatbari-Soltani S, *et al. BMJ Open* 2020; 10:e040959. doi: 10.1136/bmjopen-2020-040959

BMJ Publishing Group Limited (BMJ) disclaims all liability and responsibility arising from any reliance placed on this supplemental material which has been supplied by the author(s)

§ Calculated based on an algorithm including presence of the metabolic syndrome and type 2 diabetes, and concentrations of fasting serum insulin, fasting serum aspartate-aminotransferase (AST), and the AST/alanine-aminotransferase ratio.

Khalatbari-Soltani S, *et al. BMJ Open* 2020; 10:e040959. doi: 10.1136/bmjopen-2020-040959

Table S6 Prospective association of the Mediterranean diet score with the risk of hepatic steatosis, CoLaus study, Switzerland (n=1632), Sensitivity analysis while excluding participants with either indices high (using FLI>30 instead of FLI≥60).

	Risk ratio (95% CI) across quintiles of Mediterranean diet score*					*P*-trend	Risk ratio (95% CI) Per SD increase*
	Q1	Q2	Q3	Q4	Q5		
range	1.83-7.63	7.64-8.35	8.36-8.92	8.93-9.59	9.60-12.1		
N total	327	326	327	326	326		
Fatty liver index, median (iqr)†	14.9 (7.6, 24.8)	12.7 (7.4, 23.7)	12.3 (5.5, 22.3)	11.8 (6.3, 22.7)	10.2 (5.5, 21.8)		
N cases (score≥60)	9	5	2	2	3		
Unadjusted	1.00 (ref.)	0.56 (0.19, 1.65)	0.22 (0.05, 1.02)	0.22 (0.05, 1.02)	0.33 (0.09, 1.22)	0.047	0.66 (0.45, 0.97)
Multivariable ‡	1.00 (ref.)	0.63 (0.20, 2.00)	0.14 (0.02, 0.96)	0.27 (0.05, 1.32)	0.39 (0.09, 1.68)	0.096	0.72 (0.47, 1.10)
Multivariable + BMI	1.00 (ref.)	0.68 (0.22, 2.05)	0.13 (0.02, 0.98)	0.26 (0.06, 1.15)	0.41 (0.10, 1.70)	0.087	0.73 (0.48, 1.11)
Multivariable + BMI + WC	1.00 (ref.)	0.50 (0.18, 1.42)	0.12 (0.01, 0.98)	0.22 (0.05, 1.03)	0.39 (0.09, 1.67)	0.093	0.71 (0.46, 1.09)
NAFLD-score, median (iqr)§	-2.2 (-2.5, -1.8)	-2.2 (-2.5, -1.7)	-2.2 (-2.5, -1.8)	-2.2 (-2.6, -1.8)	-2.2 (-2.6, -1.8)		
N cases (score≥-0.640)	13	14	10	13	12		
Unadjusted	1.00 (ref.)	1.08 (0.52, 2.26)	0.77 (0.34, 1.73)	1.00 (0.47, 2.13)	0.93 (0.43, 2.00)	0.79	0.94 (0.73, 1.20)
Multivariable ‡	1.00 (ref.)	1.13 (0.55, 2.32)	0.79 (0.35, 1.76)	1.10 (0.52, 2.34)	0.98 (0.45, 2.15)	0.94	0.95 (0.73, 1.24)
Multivariable + BMI	1.00 (ref.)	1.25 (0.60, 2.60)	0.81 (0.36, 1.84)	1.20 (0.56, 2.58)	1.13 (0.51, 2.52)	0.82	0.98 (0.75, 1.28)
Multivariable + BMI + WC	1.00 (ref.)	1.17 (0.57, 2.44)	0.85 (0.37, 1.93)	1.13 (0.52, 2.45)	1.12 (0.50, 2.53)	0.84	0.98 (0.75, 1.28)

Abbreviations: iqr, interquartile range; BMI, body mass index; WC, waist circumference; NAFLD, non-alcoholic fatty liver disease.
Statistical analysis using Poisson regression with robust standard errors; results are expressed as risk ratios and (95% confidence intervals).
* In categorical analysis, the population was divided into five groups by quintiles (Q1-Q5) of the Mediterranean diet score; Standard deviation was 1.21 for the multivariable analyses.
† Calculated based on an algorithm including BMI, waist circumference, triglycerides, and gamma-glutamyl transferase.
‡ Adjusted for age (years), sex, marital status (single, married/cohabiting, and widowed/Separated/divorced), occupational status (working and not working), education level (university, high school, apprenticeship, and mandatory education), smoking status (never, former, and current), energy intake (kcal/day), total energy expenditure (kcal/day), and date of dietary assessment.
§ Calculated based on an algorithm including presence of the metabolic syndrome and type 2 diabetes, and concentrations of fasting serum insulin, fasting serum aspartate-aminotransferase (AST), and the AST/alanine-aminotransferase ratio.

Khalatbari-Soltani S, *et al. BMJ Open* 2020; 10:e040959. doi: 10.1136/bmjopen-2020-040959

Table S7 Association of the Mediterranean diet score with change in BMI and waist circumference, CoLaus study, Switzerland.

	β coefficient (95% CI) across quintiles of Mediterranean diet score*					*P*-trend	β coefficient (95% CI) Per SD increase*
	Q1	Q2	Q3	Q4	Q5		
range	1.83-7.63	7.64-8.35	8.36-8.92	8.93-9.59	9.60-12.1		
N total	458	458	457	458	457		
ΔBMI, mean±SD†	0.48 ± 1.62	0.61 ± 1.53	0.42 ± 1.44	0.50 ± 1.52	0.40 ± 1.52		
Multivariable ‡	1.00 (ref.)	0.12 (-0.08, 0.33)	-0.08 (-0.28, 0.13)	-0.04 (-0.25, 0.16)	-0.16 (-0.37, 0.04)	0.038	-0.08 (-0.15, -0.02)
ΔWaist circumference, mean±SD§	1.04 ± 6.53	0.74 ± 6.42	0.19 ± 6.00	0.57 ± 6.33	0.17 ± 6.19		
Multivariable ‡	1.00 (ref.)	-0.51 (-1.36, 0.34)	-0.85 (-1.69, 0.00)	-0.47 (-1.32, 0.38)	-0.82 (-1.68, 0.03)	0.10	-0.33 (-0.61, -0.06)

Abbreviations: BMI, Body mass index.

Statistical analysis using linear regression; results are expressed as β coefficients and (95% confidence intervals).

* In categorical analysis, the population was divided into five groups by quintiles (Q1-Q5) of the Mediterranean diet score; Standard deviation was 1.20 for the multivariable analyses.

† Calculated by subtracting BMI at baseline from BMI at follow-up.

‡ Adjusted for age (years), sex, marital status (single, married/cohabitant, and widowed/Separated/divorced), occupational status (working and not working), education level (university, high school, apprenticeship, and mandatory education), smoking status (never, former, and current), energy intake (kcal/day), total energy expenditure (kcal/day), and date of dietary assessment.

§ Calculated by subtracting waist circumference at baseline from waist circumference at follow-up.

Khalatbari-Soltani S, *et al. BMJ Open* 2020; 10:e040959. doi: 10.1136/bmjopen-2020-040959

Table S8 Association of the Mediterranean diet score with the GGT, ALT, and AST, CoLaus study, Switzerland.

	β coefficient (95% CI) across quintiles of Mediterranean diet score*					*P*-trend	β coefficient (95% CI) Per SD increase *
	Q1	Q2	Q3	Q4	Q5		
range	1.83-7.63	7.64-8.35	8.36-8.92	8.93-9.59	9.60-12.12		
N total	458	458	457	458	457		
GGT (U/l), *median* (iqr)	21 (15, 29)	20 (14, 29)	19 (14, 26)	18 (14, 28)	17 (13, 26)		
Multivariable †	1.00 (ref.)	-2.63 (-9.01, 3.76)	-5.71 (-12.07, 0.66)	-4.24 (-10.64, 2.16)	-6.03 (-12.45, 0.38)	0.063	-1.53 (-3.60, 0.53)
Multivariable + BMI	1.00 (ref.)	-2.86 (-9.24, 3.53)	-5.70 (-12.06, 0.66)	-4.50 (-10.90, 1.90)	-6.52 (-12.95, -0.09)	0.047	-1.66 (-3.73, 0.41)
Multivariable + BMI & WC	1.00 (ref.)	-2.93 (-9.32, 3.45)	-5.72 (-12.08, 0.64)	-4.54 (-10.94, 1.86)	-6.53 (-12.96, -0.10)	0.047	-1.65 (-3.72, 0.41)
ALT (U/l), *median* (iqr)	20 (16, 26)	20.5 (17, 26)	20 (16, 26)	19.5 (16, 25)	20 (16, 25)		
Multivariable †	1.00 (ref.)	0.03 (-0.02, 0.08)	0.03 (-0.02, 0.08)	-0.013 (-0.06, 0.04)	0.002 (-0.05, 0.05)	0.45	-0.006 (-0.02, 0.01)
Multivariable + BMI	1.00 (ref.)	0.04 (-0.01, 0.08)	0.03 (-0.02, 0.08)	-0.009 (-0.06, 0.04)	0.008 (-0.04, 0.06)	0.60	-0.005 (-0.02, 0.01)
Multivariable + BMI & WC	1.00 (ref.)	0.04 (-0.01, 0.08)	0.03 (-0.02, 0.08)	-0.01 (-0.06, 0.04)	0.008 (-0.04, 0.06)	0.60	-0.005 (-0.02, 0.01)
AST (U/l), *median* (iqr)	22 (19, 25)	22 (19, 26)	22 (19, 25)	22 (19, 26)	22 (19, 25)		
Multivariable †	1.00 (ref.)	0.02 (-0.01, 0.06)	0.004 (-0.03, 0.04)	0.011 (-0.03, 0.05)	0.015 (-0.02, 0.05)	0.66	0.003 (-0.01, 0.01)
Multivariable + BMI	1.00 (ref.)	0.02 (-0.02, 0.06)	0.004 (-0.03, 0.04)	0.009 (-0.03, 0.04)	0.011 (-0.03, 0.05)	0.80	0.002 (-0.01, 0.01)
Multivariable + BMI & WC	1.00 (ref.)	0.02 (-0.02, 0.06)	0.004 (-0.03, 0.04)	0.009 (-0.03, 0.04)	0.011 (-0.03, 0.05)	0.80	0.002 (-0.01, 0.01)

Abbreviations: iqr, interquartile range; GGT, gamma-glutamyl transferase; SES, socio economic status; BMI, body mass index; WC, waist circumference; ALT, Alanine transaminase; AST, aspartate-aminotransferase.

Statistical analysis using linear regression; results are expressed as β coefficients and (95% confidence intervals).

* In categorical analysis, the population was divided into five groups by quintiles (Q1-Q5) of the Mediterranean diet score; Standard deviation was 1.20 for the multivariable analyses.

† Adjusted for age (years), sex, marital status (single, married/cohabitant, and widowed/Separated/divorced), occupational status (working and not working), education level (university, high school, apprenticeship, and mandatory education), smoking status (never, former, and current), energy intake (kcal/day), total energy expenditure (kcal/day), and date of dietary assessment.

Khalatbari-Soltani S, *et al. BMJ Open* 2020; 10:e040959. doi: 10.1136/bmjopen-2020-040959
