# Supplementary material for: Prospective association between adherence to the Mediterranean diet and hepatic steatosis: the Swiss CoLaus cohort study
Source: BMJ Open. 2020 Dec 22;10(12):e040959. doi: 10.1136/bmjopen-2020-040959 (PMC7757450; doi:10.1136/bmjopen-2020-040959)
Supplement: Author's manuscript [file bmjopen-2020-040959.draft_revisions.pdf]

BMJ Open

BMJ Open is committed to open peer review. As part of this commitment we make the peer review history of every article we publish publicly available.

When an article is published we post the peer reviewers' comments and the authors' responses online. We also post the versions of the paper that were used during peer review. These are the versions that the peer review comments apply to.

The versions of the paper that follow are the versions that were submitted during the peer review process. They are not the versions of record or the final published versions. They should not be cited or distributed as the published version of this manuscript.

BMJ Open is an open access journal and the full, final, typeset and author-corrected version of record of the manuscript is available on our site with no access controls, subscription charges or pay-per-view fees (http://bmjopen.bmj.com).

If you have any questions on BMJ Open's open peer review process please email info.bmjopen@bmj.com

BMJ Open

The prospective association between adherence to the Mediterranean diet and hepatic steatosis: the Swiss CoLaus study

Journal:	*BMJ Open*
Manuscript ID	bmjopen-2020-040959
Article Type:	Original research
Date Submitted by the Author:	27-May-2020
Complete List of Authors:	Khalatbari-Soltani, Saman; The University of Sydney School of Public Health, Faculty of Medicine and Health; ARC Centre for Excellence in Population Ageing Research<br>Marques-Vidal, Pedro; University Hospital of Lausanne, Department of Internal Medicine<br>Imamura, Fumiaki; University of Cambridge School of Clinical Medicine, Medical Research Council Epidemiology Unit<br>Forouhi, Nita; University of Cambridge School of Clinical Medicine, Medical Research Council Epidemiology Unit
Keywords:	NUTRITION & DIETETICS, EPIDEMIOLOGY, PUBLIC HEALTH, Hepatology < INTERNAL MEDICINE

SCHOLARONE™
Manuscripts

BMJ

I, the Submitting Author has the right to grant and does grant on behalf of all authors of the Work (as defined in the below author licence), an exclusive licence and/or a non-exclusive licence for contributions from authors who are: i) UK Crown employees; ii) where BMJ has agreed a CC-BY licence shall apply, and/or iii) in accordance with the terms applicable for US Federal Government officers or employees acting as part of their official duties; on a worldwide, perpetual, irrevocable, royalty-free basis to BMJ Publishing Group Ltd ("BMJ") its licensees and where the relevant Journal is co-owned by BMJ to the co-owners of the Journal, to publish the Work in this journal and any other BMJ products and to exploit all rights, as set out in our licence.

The Submitting Author accepts and understands that any supply made under these terms is made by BMJ to the Submitting Author unless you are acting as an employee on behalf of your employer or a postgraduate student of an affiliated institution which is paying any applicable article publishing charge ("APC") for Open Access articles. Where the Submitting Author wishes to make the Work available on an Open Access basis (and intends to pay the relevant APC), the terms of reuse of such Open Access shall be governed by a Creative Commons licence – details of these licences and which Creative Commons licence will apply to this Work are set out in our licence referred to above.

Other than as permitted in any relevant BMJ Author's Self Archiving Policies, I confirm this Work has not been accepted for publication elsewhere, is not being considered for publication elsewhere and does not duplicate material already published. I confirm all authors consent to publication of this Work and authorise the granting of this licence.

For peer review only

TITLE PAGE

Title: The prospective association between adherence to the Mediterranean diet and hepatic steatosis: the Swiss CoLaus study

Saman Khalatbari-Soltani[1,2,3], Pedro Marques-Vidal[3], Fumiaki Imamura[4*], and Nita G. Forouhi[4*]

[1] The University of Sydney School of Public Health, Faculty of Medicine and Health, New South Wales, Australia; [2] ARC Centre of Excellence in Population Ageing Research (CEPAR), University of Sydney, Sydney, Australia; [3] Department of Internal Medicine, Internal Medicine, Lausanne University Hospital (CHUV), rue du Bugnon 46, 1011 Lausanne, Switzerland; [4] Medical Research Council Epidemiology Unit, University of Cambridge, School of Clinical Medicine, Institute of Metabolic Science, Cambridge, CB2 0QQ, UK.

*FL and NGF are joint senior authors with equal contribution

Correspondence to:

Saman Khalatbari-Soltani, ARC Centre of Excellence in Population Ageing Research (CEPAR), School of Public Heath, Faculty of Medicine and Health, the University of Sydney, NSW, 2006, Sydney Australia, +61 (0) 431 711 144, saman.khalatbarisoltani@sydney.edu.au.

Nita G. Forouhi, Medical Research Council Epidemiology Unit, University of Cambridge, School of Clinical Medicine, Institute of Metabolic Science, Cambridge, CB2 0QQ, UK, +44 (0) 1223 769145, nita.forouhi@mrc-epid.cam.ac.uk.

Running title: Mediterranean diet and hepatic steatosis.

For peer review only - http://bmjopen.bmj.com/site/about/guidelines.xhtml

Financial Support: The CoLaus study was and is supported by research grants from GlaxoSmithKline, the Faculty of Biology and Medicine of Lausanne, and the Swiss National Science Foundation (grants 33CSCO-122661, 33CS30-139468 and 33CS30-148401). SKS was supported by the Swiss National Science Foundation (Doc.Mobility number P1LAP3-171805). NGF and FI acknowledge core MRC support (MC_UU_12015/5), and NGF acknowledges NIHR Biomedical Research Centre Cambridge: Nutrition, Diet, and Lifestyle Research Theme (IS-BRC-1215-20014).

Authors' contribution: SKS, FI, and NGF designed the study question and had full access to all the data in the study and took responsibility for the integrity and accuracy of the data. SKS performed the statistical analyses and she wrote the first draft with supervision from FI, PMV, and NGF. All authors: contributed to interpretation of data, revised the article critically for important intellectual content, and approved the final version of the manuscript.

Word count: 3643

Number of figures and tables: Two tables and one figure

For peer review only

ABSTRACT

Objective: The Mediterranean diet has been promoted as a healthy dietary pattern but, whether the Mediterranean diet may help to prevent hepatic steatosis is not clear. This study aimed to evaluate the prospective association between adherence to the Mediterranean diet and risk of hepatic steatosis.

Design: Population-based prospective cohort study.

Setting: The Swiss CoLaus study.

Participants: We evaluated 2288 adults (65.4% women, aged 55.8±10.0 years) without hepatic steatosis at first follow-up in 2009-2012. Adherence to the Mediterranean diet was scaled as the Mediterranean diet score (MDS) based on the Mediterranean-diet Pyramid ascertained with responses to food-frequency questionnaires.

Outcome measures: New onset of hepatic steatosis was ascertained by two indices separately: the fatty liver index (FLI, ≥60 points) and the non-alcoholic fatty liver disease (NAFLD) score (≥-0.640 points). Prospective associations between adherence to the Mediterranean diet and risk of hepatic steatosis were quantified using Poisson regression.

Results: During a mean 5.3-years of follow-up, hepatic steatosis was ascertained in 153 (6.7%) participants by FLI criteria and in 208 (9.1%) by NAFLD-score. After multivariable adjustment, higher adherence to MDS was associated with lower risk of hepatic steatosis based on FLI: risk ratio (95% confidence interval) 0.84 (0.73, 0.96) per one standard deviation of MDS; 0.85 (0.73, 0.99) adjusted for BMI; and 0.85 (0.71, 1.02) adjusted for both BMI and waist circumference. When using NAFLD-score, no significant association was found between MDS and risk of hepatic steatosis [0.95 (0.83, 1.09)].

Conclusion: A potential role of the Mediterranean diet in the prevention of hepatic steatosis is suggested by the inverse association observed between adherence to the Mediterranean diet and incidence of hepatic steatosis based on the FLI. The inconsistency of this association

For peer review only

For peer review only - http://bmjopen.bmj.com/site/about/guidelines.xhtml

when hepatic steatosis was assessed by NAFLD-score points to the need for accurate population-level assessment of fatty liver and its physiologic markers.

Keywords: Mediterranean diet; hepatic steatosis; fatty liver disease; prospective study.

For peer review only

For peer review only - http://bmjopen.bmj.com/site/about/guidelines.xhtml

STRENGHTS AND LIMITATION OF THE STUDY

- This study had the benefit of a relatively large sample size and an average of 5.3 years of follow-up.
- We applied a definition of the Mediterranean diet that has been shown to be valid in a non-Mediterranean population.
- Our ascertainment of hepatic steatosis was based on two indices that has been validated for use in large epidemiological studies.
- The findings highlight the need for further research with more accurate measures of hepatic steatosis.

For peer review only

INTRODUCTION

Hepatic steatosis is the most common cause of liver disease [1]. In westernized countries, hepatic steatosis affects up to 34% of the general population and up to 74% of obese individuals, depending on the definition used [2–4]. Hepatic steatosis—fat content of more than 5% of liver volume—is the first recognizable stage of non-alcoholic fatty liver disease (NAFLD) [1]. Hepatic steatosis, particularly NAFLD, may progress to end-stage liver disease including fibrosis, cirrhosis, and hepatocellular carcinoma [5]. Moreover, as hepatic steatosis increases the risk of metabolic syndrome, type 2 diabetes, and cardiovascular disease (CVD), its prevention is of public health importance [6]. An unhealthy dietary pattern remains one of the primary targets of lifestyle modification for the prevention and management of hepatic steatosis and NAFLD [7,8].

The Mediterranean diet has been recently recommended for treatment of NAFLD [9]; however, evidence regarding prevention of hepatic steatosis development is sparse [10]. Adherence to the Mediterranean diet has been reported to have a beneficial impact on risks of CVD [11,12], type 2 diabetes [13], and metabolic syndrome [14]. Trial evidence demonstrated the potential benefits of the Mediterranean diet against progress of hepatic steatosis focusing on individuals with existing hepatic steatosis, either alone [15–20] or associated with metabolic risk factors such as obesity or diabetes [20–23]. Research among those without clinically manifest hepatic steatosis is restricted to observational evidence, reporting an inverse association that greater adherence to a Mediterranean diet is associated with lower prevalence of hepatic steatosis [24,25]. However, the cross-sectional design of these studies limits inference for causal associations and can be used mainly for hypothesis generation. Relevant longitudinal evidence for the primary prevention of hepatic steatosis or NAFLD has been reported only by the Framingham Heart Study, with a significant inverse

For peer review only - http://bmjopen.bmj.com/site/about/guidelines.xhtml

association of adherence to the Mediterranean diet with risk of hepatic steatosis in 1521 adults over 6 years of follow-up [26], but evidence is lacking in Europe.

Given the limited evidence from population-based epidemiological studies thus far, we aimed to investigate the prospective association of adherence to the Mediterranean diet with the risk of developing hepatic steatosis. We hypothesized that greater adherence to the Mediterranean diet would reduce the risk of hepatic steatosis.

METHODS

Study population

We evaluated participants in the CoLaus study, an ongoing population-based cohort investigating the clinical, biological, and genetic determinants of CVD in the city of Lausanne, Switzerland [27]. Inclusion criteria of the recruitment were adults of European origin, aged 35 to 75 years [27]. There were three study phases: baseline recruitment in 2003-2006 (n=6733), the first follow-up in 2009-2012 (n=5064), and the second follow-up in 2014-2017 (n=4881). We conducted dietary assessment at the first follow-up and therefore considered the first follow-up as the study baseline. Fatty liver index (FLI) and NAFLD-score, two indices of hepatic steatosis, were available at baseline [25]. If participants met the joint criterion of FLI ≥ 60 or NAFLD-score ≥ -0.640 at baseline, we excluded them as prevalent cases (see below) (n=2036). We also excluded participants with missing information on diet, outcome, and covariates (n=740) (**Figure S1**).

Dietary assessment

Participants completed a self-administered, 97-item, semi-quantitative food frequency questionnaire (FFQ) about their habitual dietary intake over the last four weeks [28], the validity of which had been assessed in canton Geneva against 24-hour recalls [28,29]. For

For peer review only - http://bmjopen.bmj.com/site/about/guidelines.xhtml

each item, participants were instructed to report consumption frequencies by selecting one of the seven frequency options from “less than once during the last 4 weeks” to “2 or more times per day” and by selecting a usual serving size (smaller, equal, or bigger to a reference size).

Mediterranean diet scores

We derived the pyramid-based Mediterranean diet score (MDS) as a measure of adherence to the Mediterranean diet from responses to the FFQ as we conducted previously [25]. This MDS is based on the Mediterranean Dietary Pyramid proposed by the Mediterranean Diet Foundation for both Mediterranean and non-Mediterranean countries and accounting for the traditional Mediterranean diet, contemporary lifestyle, and food environment [30]. We have previously reported that this MDS scoring algorithm predicted CVD incidence [31], as well as the prevalence of hepatic steatosis [25] in non-Mediterranean populations. Briefly, a continuous score of 0 to 1 was assigned for each recommended level of the 15 components of the pyramid (vegetables, legumes, and fish as healthy items; red meat, processed meat, potato, and sweets as unhealthy items; and fruits, nuts, cereals, eggs, dairy, white meat, and alcoholic beverages as items for which moderate consumption was recommended). The resulting MDS ranges between 0 and 15 on a continuous scale. The MDS calculation was adjusted to an energy intake of 2000 kcal/d (8.37 MJ/day) by applying a regression-residual technique for energy adjustment to each food group variable [31,32].

Ascertainment of hepatic steatosis

Two indices of hepatic steatosis were evaluated: fatty liver index (FLI) [33] and NAFLD liver fat score [34]. FLI was calculated based on a logistic function including body-mass index (BMI), waist circumference, fasting triglycerides, and gamma-glutamyl transferase (GGT) levels as follows:

For peer review only - http://bmjopen.bmj.com/site/about/guidelines.xhtml

$$FLI = 1 / (1+e^{-(0.953 \times \ln(\text{triglycerides}) + 0.139 \times BMI + 0.718 \times \ln(GGT) + 0.053 \times \text{waist circumference} - 15.745)})$$

FLI×100 ranges from 0 to 100. Presence of hepatic steatosis was defined by FLI ≥ 60, a value with a sensitivity of 61% and a specificity of 86% [33]. FLI was tested previously in comparison to ultrasonography with an area under the receiver operating characteristic curve of 0.78 (OR 95% CI: 0.77, 0.83) [35].

The NAFLD-score was calculated based on an algorithm including a logistic function with the presence of metabolic syndrome defined by criteria of International Diabetes Federation [36], presence of type 2 diabetes, and fasting concentrations of insulin, aspartate-aminotransferase (AST), and the AST/ alanine transaminase (ALT) ratio:

$$\text{NAFLD-score} = -2.89 + 1.18 \times \text{metabolic syndrome (yes/no)} + 0.45 \times \text{type 2 diabetes (yes /no)} + 0.15 \times \text{fasting-insulin (mU/L)} + 0.04 \times \text{fasting-AST (U/L)} - 0.94 \times AST/ALT$$

Presence of hepatic steatosis was defined by a NAFLD-score ≥ -0.640, a value with a sensitivity of 86% and a specificity of 71%, when compared to proton magnetic resonance imaging [34].

Assessment of covariates at baseline

Sociodemographic, lifestyle and health characteristics were collected by self-administered questionnaires. Age, sex, marital status, occupational status, and educational level were included as indicators of sociodemographic condition. Smoking status was classified as 'never', 'former', and 'current'. Alcohol consumption was assessed by the number of alcoholic beverage units consumed in the past week and further categorized as 'abstainers' (0 unit/week), 'moderate' (1–21 units/week for men, 1–14 for women), and 'heavy' (>21 units/week for men, >14 for women) drinkers (one unit corresponds to 8 g of alcohol). Physical activity was assessed with a self-administered quantitative physical activity

For peer review only

frequency questionnaire [37]. Health characteristics included presence of metabolic syndrome and family history of diabetes. Anthropometric and blood pressure measurements were obtained using standard procedures and equipment as previously described [27]. Plasma triglycerides, high-density lipoprotein cholesterol, and glucose were measured using standard enzymatic methods and ALT, AST, and GGT were measured using reference methods as standardized by the International Federation of Clinical Chemistry.

Statistical analysis

Statistical analyses were performed using Stata (version 15; StataCorp, College Station, TX, USA) with a two-sided test with α=0.05. Descriptive statistics were obtained in the participants included in this study in comparison to those excluded from this study. Cohen's kappa statistics were calculated to assess the agreement between the FLI and NAFLD-score.

MDS as a measure of adherence to the Mediterranean diet was evaluated both categorically (quintiles) and continuously scaled as one SD unit. The association of MDS with the risk of hepatic steatosis was assessed using multivariable-adjusted Poisson regression models with robust standard errors and estimating risk ratios (RRs) and 95% confidence intervals (CIs). Models were adjusted for age, sex, marital status, occupational status, educational level, smoking status, energy intake, total energy expenditure, and date of dietary assessment (to adjust for seasonality). We further adjusted for BMI and waist circumference as potential confounders or factors on the causal pathway to assess the possible impact of overall and central adiposity on the association of the Mediterranean diet and hepatic steatosis.

Additionally, we also adjusted for changes in BMI categories between baseline and follow-up; for alcohol consumption (units/week); for clinical variables of metabolic risk

For peer review only

For peer review only - http://bmjopen.bmj.com/site/about/guidelines.xhtml

[blood pressure >130/85 mm Hg (yes/no), triglycerides >1.7 mmol/L (yes/no), high-density lipoprotein level <1.29 mmol/L for men and <1.03 mmol/L for women (yes/no), and glucose level ≥5.6 mmol/L (yes/no)] [36]; and for family history of diabetes and metabolic syndrome (only for FLI) to examine their influence on the association of interest.

Possible interactions between MDS and age, sex, BMI, and alcohol consumption were tested using the Wald test. Several sensitivity analyses were conducted to examine the robustness of the observed findings. First, to assess the role of alcohol consumption (as alcohol is a risk factor for fatty liver accumulation), we excluded the alcohol component from the MDS, while adjusting for alcohol consumption as a covariate. We took the same approaches for the other MDS components to assess the impact of each component on the observed associations. Second, we conducted separate analyses after excluding participants with BMI≥30 kg/m^2; implausible energy intake (<500 or >3500 kcal/day in women and <800 or >4000 kcal/day in men); excessive alcohol consumption; prevalent diabetes (defined as glycated haemoglobin≥48 mmol/mol, or fasting plasma glucose ≥7.0 mmol/L, or use of hypoglycaemic drugs or insulin); or probable secondary causes of hepatic steatosis such as hepatitis B or C, HIV, hepatotoxic, or autoimmune disease medications. We evaluated the robustness of the results to an alternative definition of prevalent hepatic steatosis. While we excluded participants with prevalent hepatic steatosis using the specified cut-offs of FLI or NAFLD-score in the primary analysis, we used each of the two indices separately in sensitivity analyses, whereby we evaluated 2652 adults in longitudinal analysis based on FLI; and 2568 adults, based on NAFLD. Finally, we used more restrictive cutpoints and excluded participants with NAFLD-score ≥-0.640 or with FLI>30.

In a post-hoc analysis pertaining to sensitivity of the results to model covariates and for better understanding of potential mechanisms, longitudinal associations of MDS with follow-up measures of log-transformed GGT, ALT, and AST levels and with changes in BMI

For peer review only - http://bmjopen.bmj.com/site/about/guidelines.xhtml

and waist circumference from baseline to follow-up were examined using multivariable-adjusted linear regression. These results were expressed as β coefficient (95% CIs) for changes in each measure per 1-SD difference in MDS.

RESULTS

Participant characteristics

Of the initial 5064 participants, 2776 (54.8%) were excluded, leaving 2288 participants (65.4% women; 55.8±10.0 years) for analysis. Participants included were more likely to be women, show higher sociodemographic characteristics and lower BMI, waist circumference, liver enzymes, or prevalence of metabolic syndrome in comparison to excluded individuals (**Table S1)**. Being in the highest quintile of MDS was higher among women compared to men, positively correlated with sociodemographic characteristics, and negatively correlated with being current smokers, heavy alcohol drinkers, and with BMI, waist circumference, GGT, and TG (**Table 1**).

Adherence to the Mediterranean diet and risk of hepatic steatosis

After a mean 5.3 (SD: 0.5) years of follow-up, there were 153 (6.7%) and 208 (9.1%) participants with hepatic steatosis based on FLI and NAFLD-score, respectively (**Table S2**). Case identification by FLI and NAFLD-score was modestly concordant (kappa=0.60).

Multivariable-adjusted analysis showed an inverse association between MDS quintiles and risk of hepatic steatosis based on FLI (p_{trend}<0.006) with RR (95% CI) comparing the top to the bottom category of 0.50 (0.28, 0.91). The inverse associations across quintiles of MDS weakened after adjustment for BMI (p_{trend}=0.031) or both BMI and waist circumference (p_{trend}=0.034) (**Table 2**): RR (95% CI)=0.61 (0.34, 1.09) and 0.60 (0.34, 1.08), respectively. In analyses using MDS as a continuous variable, the inverse association with risk of hepatic

For peer review only - http://bmjopen.bmj.com/site/about/guidelines.xhtml

For peer review only

steatosis based on FLI [RR 0.84 (0.73, 0.96) per one SD of MDS] remained unchanged but getting imprecise after adjustment for BMI [RR 0.85 (0.73, 0.99)] and adjustment for both BMI and waist circumference [0.85 (0.71, 1.02)]. In sensitivity analysis, the magnitude of the inverse associations little changed after further adjustment for alcohol consumption, presence of metabolic syndrome, changes in BMI categories or clinical variables (medication use or prevalent diseases) (**Table S3**), while adjustment for BMI and clinical variables increased standard errors.

Conversely, there was no association between MDS and the risk of hepatic steatosis defined by NAFLD-score criteria, with RRs (95% CIs) ranging from 0.93 (0.82, 1.05) to 1.00 (0.86, 1.17) over different regression models (**Table 2** and **Table S3**).

Interaction and sensitivity analyses

No significant interactions were found between MDS and age, sex, BMI, or alcohol consumption on risk of hepatic steatosis ($p_{interaction}$>0.05; results not shown). The contribution of each component of the MDS on risk of hepatic steatosis was assessed by sequential subtraction of components from the score (**Figure 1**). Excluding the components of the MDS did not substantially affect the inverse associations with hepatic steatosis based on FLI; the magnitude of the associations remained reasonably stable, but it became weaker (p>0.05) after excluding fruits, cereals, dairy products, red or processed meat, or alcohol.

In sensitivity analyses, when excluding the alcohol component from the MDS but adjusting for alcohol consumption as a covariate, the inverse associations between MDS and risk of hepatic steatosis based on FLI became weaker (**Table S4**). The primary results were not different when excluding participants with BMI≥30 kg/m^2, excessive alcohol consumption, or secondary causes of hepatic steatosis (**Table S4**). Excluding participants with implausible energy intakes weakened the associations (**Table S4**). The analysis of an

For peer review only - http://bmjopen.bmj.com/site/about/guidelines.xhtml

alternative definition of prevalent hepatic steatosis, excluding participants with only high FLI score at baseline, did not alter the significant inverse association between MDS and risk of FLI-based hepatic steatosis (**Table S5**). Effect sizes were of slightly higher magnitude when excluding those with FLI>30 or NAFLD-score≥-0.640 at baseline, but CIs were wider due to smaller sample size (**Table S6**).

For NAFLD-score, no significant associations were found in any of the sensitivity analyses (**Tables S4 and S5** and **Figure S2**). The sole exception was when participants with a high NAFLD-score at baseline were excluded, where an inverse association between MDS quintiles and risk of hepatic steatosis was present (p_{trend}=0.039), but this association was attenuated to the null after adjustment for BMI (**Table S5**).

Longitudinal analyses for adiposity and markers of hepatic function

In post-hoc exploratory analyses there were inverse associations of MDS with changes in BMI [β coefficient (95% CIs) per one SD higher MDS of -0.08 (-0.15, -0.02)] and in waist circumference [-0.33 (-0.61, -0.06)] (**Table S7**). For the markers of hepatic function, MDS showed a trend toward inverse association with GGT levels (p_{trend}=0.047) [β coefficient (95% CIs) per one SD higher MDS of -1.66 (-3.73, 0.41)], but not with ALT or AST levels (**Table S8**).

DISCUSSION

In this first population-based European study among adults free from clinically manifest hepatic steatosis to report on the prospective association between adherence to the Mediterranean diet and risk of hepatic steatosis, we found an inverse association between MDS and risk of hepatic steatosis based on FLI criteria. This relationship was attenuated to the null when controlled for general and central adiposity assessed by the BMI and waist

For peer review only - http://bmjopen.bmj.com/site/about/guidelines.xhtml

circumference. In contrast, there was no association between adherence to the Mediterranean diet and risk of hepatic steatosis based on NAFLD-score criteria.

Current findings in context of other evidence

Our finding based on FLI is consistent with the only published prospective study relating the Mediterranean diet to hepatic steatosis, where each SD increase in MDS was estimated to decrease the odds for incident hepatic steatosis by 26% (95% CI 10%-39%) [26]. By contrast, the point estimate of the effect size in our study was smaller [(16% (95% CI 4%-27%)]. A possible explanation partly lies in methodological differences. We used biochemical and anthropometric markers to estimate hepatic steatosis in the current study, while the previous study used computed tomography assessment.

No association was found between adherence to the Mediterranean diet and risk of hepatic steatosis based on the NAFLD-score. For possible explanations based on the differences in their components, the FLI includes GGT, while NAFLD-score includes AST and AST/ALT ratio; the FLI includes adiposity markers, while the NAFLD-score does not; the FLI includes a lipid marker (triglycerides) while NAFLD-score includes markers of glycaemic status. Previous studies showed a modest association of GGT and ALT (but not AST) with the prevalence of hepatic steatosis [1]. Indeed, our analysis showed an inverse association between MDS and GGT levels, but not with AST or ALT, and these findings could explain the discrepancy between the two indices. Notably, our sensitivity analyses using different definitions of hepatic steatosis showed an inverse association between MDS quintiles and risk of hepatic steatosis based on NAFLD-score after excluding participants with high baseline NAFLD-score only. This could be explained by modest concordance between the two measures. Of note, there were no statistically significant associations between MDS and risk of hepatic steatosis based on FLI after excluding participants with FLI>30 at

For peer review only - http://bmjopen.bmj.com/site/about/guidelines.xhtml

baseline, which led to a smaller sample size and consequently a lower statistical power in our study. The inverse association between MDS and risk of hepatic steatosis defined by FLI remained significant after adjusting for BMI only but became imprecise and not significant after adjusting for both BMI and waist circumference or for changes in BMI over the follow-up period. These results suggest the collinearity between the central adiposity and hepatic steatosis as the central adiposity may partly reflect hepatic steatosis. This biologically plausible finding is in agreement with previous cross-sectional findings for MDS and prevalent hepatic steatosis in CoLaus study and the British Fenland Study we reported [25] and a study in Hong Kong [38]. Our finding that the MDS was negatively associated with an increase in BMI after 5.3 years of follow-up is consistent with the findings from the Framingham Heart Study which observed the same trend over 6-year follow-up [26]; and from Nurses' Health Study and Health Professionals' Follow-up Study evaluating 20-year longitudinal data with repeated self-reported measures of diet and adiposity measures [39].

Sequential subtraction of different components of the MDS showed that fruits, cereals, dairy products, red or processed meat, or alcohol partially accounted for the observed association. These findings agree with previous studies [24,26,40,41] including the Framingham Heart Study suggesting benefits of low consumption of red meat and high consumption of fruits or whole grains [26]; and a cross-sectional analysis from the PREDIMED study suggesting the benefit of low consumption of red meat [24]. Moreover, the Mediterranean diet is characterized by a moderate-to-high consumption of whole grains, which has been inversely associated with the likelihood of having NAFLD [42].

Possible mechanisms and implications

Different components of the Mediterranean diet, mainly omega 3 polyunsaturated fatty acids (PUFA), fibre, and antioxidants, are inversely associated with hepatic steatosis [43,44].

For peer review only - http://bmjopen.bmj.com/site/about/guidelines.xhtml

For peer review only

High levels of polyphenols found in fruits and vegetables and high levels of monounsaturated fatty acids of olive oil have been shown to inhibit *de novo* lipogenesis, improve peripheral insulin sensitivity, and reduce cardiovascular risk mainly due to their antioxidant, anti-inflammatory, and anti-fibrotic effects [16,45]. One meta-analysis of interventional studies reported that omega-3 PUFA were negatively associated with hepatic steatosis [46]. The Mediterranean diet is also low in saturated fat, which has been demonstrated to increase hepatic triglycerides content and hepatic insulin resistance [47,48]. Finally, the high fibre content of the Mediterranean diet has been associated with reduced hepatic fat [16,43].

Hepatic steatosis is associated with cardiometabolic diseases and substantially impacts public health [11,13,14]. Thus, our finding of an inverse association between Mediterranean diet and risk of hepatic steatosis strongly reinforces the importance of dietary advice for the prevention of hepatic steatosis. However, future work should confirm whether or not the clinical importance of the Mediterranean diet for the prevention of hepatic steatosis is independent of obesity or central adiposity.

Strengths and limitations

To our knowledge, this is the first European prospective study assessing the association between the Mediterranean diet and risk of hepatic steatosis. The study had the benefit of a relatively large sample size and an average of 5.3 years of follow-up, and applied a definition of the Mediterranean diet that has been shown to be valid in a non-Mediterranean population [31].

Several limitations of this study merit consideration. Measurement error and recall bias are inevitable when using self-reported dietary instruments, limiting the ability to precisely measure adherence to the Mediterranean diet, although adjustment for energy intake may have reduced the magnitude of measurement error [49]. Our ascertainment of hepatic

For peer review only - http://bmjopen.bmj.com/site/about/guidelines.xhtml

steatosis was based on two indices, but not on liver biopsy or direct imaging assessment; hence, information bias (misdiagnosis) cannot be ruled out. Although liver biopsy is the gold standard for diagnosing hepatic steatosis, its use in apparently healthy participants would be unethical and unfeasible in a large population-based study. Further, previous studies have shown that FLI and NAFLD-score can accurately identify hepatic steatosis with good sensitivity and specificity, and these scores have been validated for use in large epidemiological studies [34,35,50,51]. Although we adjusted for many relevant confounders and performed a series of sensitivity analyses, we cannot rule out residual confounding due to unmeasured variables or covariates measured with error. Generalisability is limited because included participants seemed to be healthier than those excluded, and our findings were obtained in a single European population. Still, they confirm the findings from a previous prospective study conducted in the US [26], and might serve as a reference for other studies.

Conclusion

Adherence to the Mediterranean diet was inversely associated with risk of hepatic steatosis based on the FLI, and the association was independent of several known risk factors. Conversely, the association was not observed when using different criteria specifying the NAFLD-score. These findings support recommendations on following the Mediterranean diet for hepatic steatosis prevention in addition to the existing evidence for its benefit for cardiovascular disease prevention. Nonetheless, the findings also highlight the need for further research with more accurate measures of hepatic steatosis to replicate these findings in different populations and settings.

For peer review only - http://bmjopen.bmj.com/site/about/guidelines.xhtml

ABBREVIATIONS

NAFLD, non-alcoholic fatty liver disease; CVD; cardiovascular disease; FFQ, food frequency questionnaire; MDS, Mediterranean diet score; FLI, fatty liver index; BMI, body-mass index; TG, triglyceride; GGT, gamma-glutamyl transferase; AST, aspartate-aminotransferase; ALT, alanine transaminase; PUFA, polyunsaturated fatty acids.

DECLARATION

Acknowledgements: The authors are grateful to all the participants and staff of CoLaus study.

Conflict of Interest: None.

Ethics approval and consent to participate: The institutional Ethics Committee of the University of Lausanne, which afterwards became the Ethics Commission of Canton Vaud (www.cer-vd.ch) approved the baseline CoLaus study (reference 16/03); the approval was renewed for the first (reference 33/09) and the second (reference 26/14) follow-ups.

The CoLaus study was performed in agreement with the Helsinki declaration and its former amendments, and all participants provided their written informed consent before entering the study.

For peer review only

SUPPLEMENTARY INFORMATION

Figure S1 Sample selection flow chart.

Table S1 Characteristics of the participants included and excluded from the analysis, CoLaus study, Switzerland.

Table S2 Baseline characteristics of the participants according to the risk of hepatic steatosis, defined by the fatty liver index or the NAFLD-score, CoLaus study, Switzerland.

Table S3 Prospective association of the Mediterranean diet score with the risk of hepatic steatosis, CoLaus study, Switzerland: assessment of influence of other potential covariates.

Table S4 Sensitivity analyses for the association of the Mediterranean diet score with the risk of hepatic steatosis, CoLaus study, Switzerland.

Table S6 Prospective association of the Mediterranean diet score with the risk of hepatic steatosis, CoLaus study, Switzerland (n=1632). Sensitivity analysis after excluding participants with either indices high; using FLI>30 instead of FLI≥60.

Table S7 Association of the Mediterranean diet score with change in BMI and waist circumference, CoLaus study, Switzerland.

Table S8 Association of the Mediterranean diet score with GGT, ALT, and AST, CoLaus study, Switzerland.

For peer review only

For peer review only - http://bmjopen.bmj.com/site/about/guidelines.xhtml

REFERENCES

[1] Sattar N, Forrest E, Preiss D. Non-alcoholic fatty liver disease. BMJ 2014;349. https://doi.org/10.1136/bmj.g4596.

[2] Angulo P. Treatment of nonalcoholic fatty liver disease. AnnHepatol 2002;1:12–9.

[3] Williamson RM, Price JF, Glancy S, Perry E, Nee LD, Hayes PC, et al. Prevalence of and Risk Factors for Hepatic Steatosis and Nonalcoholic Fatty Liver Disease in People With Type 2 Diabetes: the Edinburgh Type 2 Diabetes Study. Diabetes Care 2011;34:1139 LP – 1144.

[4] Bellentani S, Scaglioni F, Marino M, Bedogni G. Epidemiology of non-alcoholic fatty liver disease. Dig Dis 2010;28:155–61. https://doi.org/10.1159/000282080.

[5] Angulo P, Keach JC, Batts KP, Lindor KD. Independent predictors of liver fibrosis in patients with nonalcoholic steatohepatitis. Hepatology 1999;30:1356–62. https://doi.org/10.1002/hep.510300604.

[6] Marchesini G, Petta S, Grave RD. Diet, Weight Loss, and Liver Health in Nonalcoholic Fatty Liver Disease: Pathophysiology, Evidence, and Practice. Hepatology 2016;63plos:2032–43. https://doi.org/10.1002/hep.28392.

[7] Marchesini G, Day CP, Dufour JF, Canbay A, Nobili V, Ratziu V, et al. EASL-EASD-EASO Clinical Practice Guidelines for the management of non-alcoholic fatty liver disease. J Hepatol 2016;64:1388–402.

[8] Chalasani N, Younossi Z, Lavine JE, Diehl AM, Brunt EM, Cusi K, et al. The diagnosis and management of non-alcoholic fatty liver disease: practice Guideline by the American Association for the Study of Liver Diseases, American College of Gastroenterology, and the American Gastroenterological Association. Hepatology 2012;55:2005–23. https://doi.org/10.1002/hep.25762.

[9] Sawangjit R, Chongmelaxme B, Phisalprapa P, Saokaew S, Thakkinstian A, Kowdley K V, et al. Comparative efficacy of interventions on nonalcoholic fatty liver disease (NAFLD): A PRISMA-compliant systematic review and network meta-analysis. Med 2016;95:e4529. https://doi.org/10.1097/md.0000000000004529.

[10] Plauth M, Bernal W, Dasarathy S, Merli M, Plank LD, Schütz T, et al. ESPEN guideline on

For peer review only - http://bmjopen.bmj.com/site/about/guidelines.xhtml

clinical nutrition in liver disease. Clin Nutr 2019;38:485–521. https://doi.org/https://doi.org/10.1016/j.clnu.2018.12.022.

[11] Estruch R, Ros E, Salas-Salvadó J, Covas M-I, Corella D, Arós F, et al. Primary Prevention of Cardiovascular Disease with a Mediterranean Diet Supplemented with Extra-Virgin Olive Oil or Nuts. N Engl J Med 2018;378:e34. https://doi.org/10.1056/NEJMoa1800389.

[12] Sofi F, Cesari F, Abbate R, Gensini GF, Casini A, Francesco S, et al. Adherence to Mediterranean diet and health status: meta-analysis. BMJ 2008;337:a1344. https://doi.org/10.1136/bmj.a1344.

[13] Salas-Salvado J, Bullo M, Babio N, Martinez-Gonzalez MA, Ibarrola-Jurado N, Basora J, et al. Reduction in the incidence of type 2 diabetes with the Mediterranean diet: results of the PREDIMED-Reus nutrition intervention randomized trial. Diabetes Care 2011;34:14–9. https://doi.org/10.2337/dc10-1288.

[14] Esposito K, Kastorini C-M, Panagiotakos DB, Giugliano D. Mediterranean diet and metabolic syndrome: an updated systematic review. Rev Endocr Metab Disord 2013;14:255–63. https://doi.org/10.1007/s11154-013-9253-9.

[15] Ryan MC, Itsiopoulos C, Thodis T, Ward G, Trost N, Hofferberth S, et al. The Mediterranean diet improves hepatic steatosis and insulin sensitivity in individuals with non-alcoholic fatty liver disease. J Hepatol 2013;59:138–43. https://doi.org/10.1016/j.jhep.2013.02.012.

[16] Properzi C, O'Sullivan TA, Sherriff JL, Ching HL, Jeffrey GP, Buckley RF, et al. Ad libitum Mediterranean and Low Fat Diets both Significantly Reduce Hepatic Steatosis: a Randomized Controlled Trial. Hepatology 2018. https://doi.org/10.1002/hep.30076.

[17] Misciagna G, del Pilar Diaz M, Caramia D V, Bonfiglio C, Franco I, Noviello MR, et al. Effect of a low glycemic index Mediterranean diet on non-alcoholic fatty liver disease. A randomized controlled clinici trial. J Nutr Heal AGING 2017;21:404–12. https://doi.org/10.1007/s12603-016-0809-8.

[18] Trovato FM, Catalano D, Martines GF, Pace P, Trovato GM. Mediterranean diet and non-alcoholic fatty liver disease: the need of extended and comprehensive interventions. Clin Nutr 2015;34:86–8. https://doi.org/10.1016/j.clnu.2014.01.018.

For peer review only - http://bmjopen.bmj.com/site/about/guidelines.xhtml

[19] Gelli C, Tarocchi M, Abenavoli L, Di Renzo L, Galli A, De Lorenzo A. Effect of a counseling-supported treatment with the Mediterranean diet and physical activity on the severity of the non-alcoholic fatty liver disease. World J Gastroenterol 2017;23:3150–62. https://doi.org/10.3748/wjg.v23.i17.3150.

[20] Baratta F, Pastori D, Polimeni L, Ernesti I, Del Ben M, Angelico F. Adherence to mediterranean diet and non-alcoholic fatty liver disease: Impact on metabolic profile. Nutr Metab Cardiovasc Dis 2017;27 (1):e8.

[21] Abenavoli L, Greco M, Nazionale I, Peta V, Milic N, Accattato F, et al. Effects of Mediterranean diet supplemented with silybin-vitamin E-phospholipid complex in overweight patients with non-alcoholic fatty liver disease. Expert Rev Gastroenterol Hepatol 2015;9:519–27. https://doi.org/10.1586/17474124.2015.1004312.

[22] Fraser A, Abel R, Lawlor DA, Fraser D, Elhayany A. A modified Mediterranean diet is associated with the greatest reduction in alanine aminotransferase levels in obese type 2 diabetes patients: results of a quasi-randomised controlled trial. Diabetologia 2008;51:1616–22. https://doi.org/10.1007/s00125-008-1049-1.

[23] Kontogianni MD, Tileli N, Margariti A, Georgoulis M, Deutsch M, Tiniakos D, et al. Adherence to the Mediterranean diet is associated with the severity of non-alcoholic fatty liver disease. Clin Nutr 2014;33:678–83. https://doi.org/10.1016/j.clnu.2013.08.014.

[24] Cantero I, Abete I, Babio N, Arós F, Corella D, Estruch R, et al. Dietary Inflammatory Index and liver status in subjects with different adiposity levels within the PREDIMED trial. Clin Nutr 2017. https://doi.org/http://dx.doi.org/10.1016/j.clnu.2017.06.027.

[25] Khalatbari-Soltani S, Imamura F, Brage S, De Lucia Rolfe E, Griffin SJ, Wareham NJ, et al. The association between adherence to the Mediterranean diet and hepatic steatosis: cross-sectional analysis of two independent studies, the UK Fenland Study and the Swiss CoLaus Study. BMC Med 2019;17:19. https://doi.org/10.1186/s12916-019-1251-7.

[26] Ma J, Hennein R, Liu C, Long MT, Hoffmann U, Jacques PF, et al. Improved Diet Quality Associates With Reduction in Liver Fat, Particularly in Individuals With High Genetic Risk Scores for Nonalcoholic Fatty Liver Disease. Gastroenterology 2018;155:107–17.

For peer review only - http://bmjopen.bmj.com/site/about/guidelines.xhtml

https://doi.org/10.1053/j.gastro.2018.03.038.

[27] Firmann M, Mayor V, Vidal PM, Bochud M, Pecoud A, Hayoz D, et al. The CoLaus study: a population-based study to investigate the epidemiology and genetic determinants of cardiovascular risk factors and metabolic syndrome. BMC Cardiovasc Disord 2008;8:6. https://doi.org/10.1186/1471-2261-8-6.

[28] Morabia A, Bernstein M, Kumanyika S, Sorenson A, Mabiala I, Prodolliet B, et al. Développement et validation d'un questionnaire alimentaire semi-quantitatif à partir d'une enquête de population. Soz Praventivmed 1994;39:345–69. https://doi.org/10.1007/BF01299666.

[29] Bernstein L, Huot I MA. Amélioration des performances d'un questionnaire alimentaire semi-quantitatif comparé à un rappel des 24 heures. Sante Publique (Paris) 1995;7:403–13.

[30] Bach-Faig A, Berry EM, Lairon D, Reguant J, Trichopoulou A, Dernini S, et al. Mediterranean diet pyramid today. Science and cultural updates. Public Health Nutr 2011;14:2274–84. https://doi.org/10.1017/S1368980011002515.

[31] Tong TYN, Wareham NJ, Khaw K-T, Imamura F, Forouhi NG. Prospective association of the Mediterranean diet with cardiovascular disease incidence and mortality and its population impact in a non-Mediterranean population: the EPIC-Norfolk study. BMC Med 2016;14:135. https://doi.org/10.1186/s12916-016-0677-4.

[32] Willett WC, Howe GR, Kushi LH. Adjustment for total energy intake in epidemiologic studies. Am J Clin Nutr 1997;65:1220S-1228S; discussion 1229S-1231S.

[33] Bedogni G, Bellentani S, Miglioli L, Masutti F, Passalacqua M, Castiglione A, et al. The Fatty Liver Index: a simple and accurate predictor of hepatic steatosis in the general population. BMC Gastroenterol 2006;6:33. https://doi.org/10.1186/1471-230X-6-33.

[34] Kotronen A, Peltonen M, Hakkarainen A, Sevastianova K, Bergholm R, Johansson LM, et al. Prediction of Non-Alcoholic Fatty Liver Disease and Liver Fat Using Metabolic and Genetic Factors. Gastroenterology 2009;137:865–72. https://doi.org/10.1053/j.gastro.2009.06.005.

[35] Ruhl CE, Everhart JE. Fatty liver indices in the multiethnic United States National Health and Nutrition Examination Survey. Aliment Pharmacol Ther 2015;41:65–76.

For peer review only - http://bmjopen.bmj.com/site/about/guidelines.xhtml

https://doi.org/10.1111/apt.13012.

[36] Alberti KGMM, Zimmet P, Shaw J. Metabolic syndrome--a new world-wide definition. A Consensus Statement from the International Diabetes Federation. Diabet Med 2006;23:469–80. https://doi.org/10.1111/j.1464-5491.2006.01858.x.

[37] Bernstein M, Sloutskis D, Kumanyika S, Sparti A, Schutz Y, Morabia A. Data-based approach for developing a physical activity frequency questionnaire. Am J Epidemiol 1998;147:147–54.

[38] Chan R, Wong VW, Chu WC, Wong GL, Li LS, Leung J, et al. Diet-Quality Scores and Prevalence of Nonalcoholic Fatty Liver Disease: A Population Study Using Proton-Magnetic Resonance Spectroscopy. PLoS One 2015;10:e0139310. https://doi.org/10.1371/journal.pone.0139310.

[39] Fung TT, Pan A, Hou T, Chiuve SE, Tobias DK, Mozaffarian D, et al. Long-Term Change in Diet Quality Is Associated with Body Weight Change in Men and Women. J Nutr 2015;145:1850–6. https://doi.org/10.3945/jn.114.208785.

[40] Freedman ND, Cross AJ, McGlynn KA, Abnet CC, Park Y, Hollenbeck AR, et al. Association of meat and fat intake with liver disease and hepatocellular carcinoma in the NIH-AARP cohort. J Natl Cancer Inst 2010;102:1354–65. https://doi.org/10.1093/jnci/djq301.

[41] Georgoulis M, Kontogianni MD, Yiannakouris N. Mediterranean Diet and Diabetes: Prevention and Treatment. Nutrients 2014;6:1406–23. https://doi.org/10.3390/nu6041406.

[42] Georgoulis M, Kontogianni MD, Tileli N, Margariti A, Fragopoulou E, Tiniakos D, et al. The impact of cereal grain consumption on the development and severity of non-alcoholic fatty liver disease. Eur J Nutr 2014;53:1727–35. https://doi.org/10.1007/s00394-014-0679-y.

[43] Zelber-Sagi S, Salomone F, Mlynarsky L. The Mediterranean dietary pattern as the diet of choice for non-alcoholic fatty liver disease: Evidence and plausible mechanisms. Liver Int 2017;37:936–49.

[44] Mahady SE, George J. Exercise and diet in the management of nonalcoholic fatty liver disease. Metab Exp 2016;65:1172–82. https://doi.org/10.1016/j.metabol.2015.10.032.

[45] Salomone F, Godos J, Zelber-Sagi S. Natural antioxidants for non-alcoholic fatty liver disease: molecular targets and clinical perspectives. LIVER Int 2016;36:5–20.

For peer review only - http://bmjopen.bmj.com/site/about/guidelines.xhtml

For peer review only

https://doi.org/10.1111/liv.12975.

[46] Parker HM, Johnson NA, Burdon CA, Cohn JS, O'Connor HT, George J. Omega-3 supplementation and non-alcoholic fatty liver disease: A systematic review and meta-analysis. J Hepatol 2012;56:944–51. https://doi.org/10.1016/j.jhep.2011.08.018.

[47] Hernandez EA, Kahl S, Seelig A, Begovatz P, Irmler M, Kupriyanova Y, et al. Acute dietary fat intake initiates alterations in energy metabolism and insulin resistance. J Clin Invest 2017;127:695–708.

[48] Bjermo H, Iggman D, Kullberg J, Dahlman I, Johansson L, Persson L, et al. Effects of n-6 PUFAs compared with SFAs on liver fat, lipoproteins, and inflammation in abdominal obesity: a randomized controlled trial. Am J Clin Nutr 2012;95:1003–12. https://doi.org/10.3945/ajcn.111.030114.

[49] Hu FB, Stampfer MJ, Rimm E, Ascherio A, Rosner BA, Spiegelman D, et al. Dietary fat anc coronary heart disease: a comparison of approaches for adjusting for total energy intake and modeling repeated dietary measurements. Am J Epidemiol 1999;149:531–40.

[50] Koehler EM, Schouten JNL, Hansen BE, Hofman A, Stricker BH, Janssen HLA. External Validation of the Fatty Liver Index for Identifying Nonalcoholic Fatty Liver Disease in a Population-based Study. Clin Gastroenterol Hepatol 2013;11:1201–4.

[51] Machado M V, Cortez-Pinto H. Non-invasive diagnosis of non-alcoholic fatty liver disease. A critical appraisal. J Hepatol 2013;58:1007–19. https://doi.org/10.1016/j.jhep.2012.11.021.

For peer review only

Table 1 Baseline characteristics of participants according to quintiles of the Mediterranean diet score, CoLaus study, Switzerland (n=2,288).

	Quintiles of Mediterranean diet score*					
Characteristic	Q1 (n=458)	Q2 (n=458)	Q3 (n=457)	Q4 (n=458)	Q5 (n=457)	*P*-value*
Age (years)	57.1±10.6	56.7±9.9	56.4±10.5	55±9.8	53.7±8.9	<0.001
Women (%)	59.0	62.9	67.6	66.6	71.1	0.001
Marital status (%)						0.51
Single	17.7	14.2	16.8	17.5	16.4	
Married/cohabitant	53.9	55.7	59.1	57.2	57.3	
Widowed/separated/divorced	28.4	30.1	24.1	25.3	26.3	
Employed (%)	57.9	60.3	59.1	68.3	72.0	<0.001
Education (%)						
University	19.2	21.6	26.0	31.7	31.1	
High school	26.4	28.2	28.4	29.7	29.5	
Apprenticeship	39.3	35.2	34.8	28.2	28.4	
Mandatory education	15.1	15.1	10.7	10.5	10.9	<0.001
Current smokers (%)	24.7	21.8	22.3	16.2	15.5	0.014
Alcohol intake (%)†						
Abstainers	21.8	24.0	25.8	24.7	21.2	
Moderate	63.8	66.2	69.4	70.7	76.6	
Heavy	14.4	9.8	4.8	4.6	2.2	<0.001
Total energy intake (kcal/day)	1819±705	1812±675	1781±729	1821±653	1801±595	0.87
Protein (%energy)	15.8±3.3	15.9±4.1	15.3±3.0	15.2±3.0	14.7±2.6	<0.001
Carbohydrates (%energy)	45.3±9.2	45.9±9.1	47.3±8.0	47.6±8.2	49.0±7.8	<0.001
Fat (%energy)	33.6±6.5	34.5±6.9	34.6±6.7	34.5±6.5	34.1±6.9	0.11
TEE (kcal/day)	2562±589	2600±618	2564±602	2558±555	2589±565	0.84
Metabolic syndrome (%)‡	11.8	9.8	12.3	12.7	7.4	0.063

For peer review only - http://bmjopen.bmj.com/site/about/guidelines.xhtml

BMI (kg/m^2)	24.0±2.7	23.8±2.9	23.9±3.0	23.6±2.8	23.3±2.7	0.003
Waist circumference (cm)	85.4±8.5	84.9±9.4	85.0±9.2	84.0±8.7	82.9±8.6	<0.001
Triglycerides (mmol/l)	1.1±0.5	1.0±0.5	1.1±0.5	1.0±0.5	1.0±0.5	
median (iqr)	1.0 (0.8, 1.3)	0.9 (0.7, 1.2)	1.0 (0.7, 1.3)	0.9 (0.7, 1.2)	0.9 (0.7, 1.2)	0.009
GGT (U/l)	25.0±16.5	25.1±17.9	23.2±14.5	24.0±17.9	21.5±12.1	0.002
median (iqr)	20 (15, 29)	21 (15, 28)	19 (14, 28)	19 (14, 26)	18 (14, 25)	
≥50 (%)	7.4	6.3	5.7	6.1	2.6	0.025
ALT (U/l)	21.3±7.8	22.1±8.9	22.5±9.7	21.3±8.7	21.5±8.7	0.12
median (iqr)	19 (16, 25)	20 (16, 26)	20 (16, 27)	19 (16, 25)	20 (16, 24)	
≥40 (%)	3.5	5.4	8.3	4.6	3.5	0.005
AST (U/l)	26.0±5.85	26.2±6.5	26.1±6.6	25.8±6.1	25.7±5.7	0.68
median (iqr)	25 (22, 29)	25 (22, 29)	25 (21, 29)	25 (22, 29)	25 (22, 29)	
≥37 (%)	4.1	8.3	6.3	5.0	4.4	0.038

Abbreviations: TEE, total energy expenditure; BMI, body mass index; iqr, interquartile range; GGT, gamma-glutamyl transferase; ALT, alanine aminotransferase; AST, aspartate aminotransferase.

* The population was divided into five groups by quintiles (Q1-Q5) of the Mediterranean diet score. P-values were computed by using ANOVA for continuous variables and Chi-square test for categorical variables.

† Alcohol consumption categorized as “abstainers” (0 unit/week), “moderate” (1–21 units/week for men, 1–14 for women), and “heavy drinkers” (>21 units/week for men, >14 for women).

‡ Metabolic syndrome defined according to the International Diabetic Federation (waist circumference ≥94 cm in men and ≥80 cm in women plus at least two of the following factors: serum triglycerides≥1.70 mmol/L or specific treatment for this lipid abnormality; serum high-density lipoprotein cholesterol <1.03 mmol/L in men and <1.29 mmol/L in women or specific treatment for this lipid abnormality; systolic blood pressure ≥130 mm Hg or diastolic blood pressure ≥85 mm Hg or treatment for previously diagnosed hypertension; and fasting plasma glucose ≥5.6 mmol/L or previously diagnosed type 2 diabetes).

For peer review only - http://bmjopen.bmj.com/site/about/guidelines.xhtml

Table 2 Prospective association of the Mediterranean diet score with the risk of hepatic steatosis, CoLaus study, Switzerland (n=2,288).

	Risk ratio (95% CI) across quintiles of Mediterranean diet score*					*P*-trend	Risk ratio (95% CI) Per SD increase*
	Q1	Q2	Q3	Q4	Q5		
range	1.83-7.63	7.64-8.35	8.36-8.92	8.93-9.59	9.60-12.1		
N total	458	458	457	458	457		
Fatty liver index, median (iqr)†	21.8 (10.4, 37.7)	20.4 (9.0, 39.4)	18.5 (8.9, 34.6)	18.1 (7.7, 33.1)	14.2 (6.6, 28.5)		
N cases (score≥60)	36	43	35	22	17		
Unadjusted	1.00 (ref.)	1.19 (0.78, 1.82)	0.97 (0.62, 1.52)	0.61 (0.37, 1.02)	0.47 (0.27, 0.83)	0.001	0.79 (0.70, 0.90)
Multivariable ‡	1.00 (ref.)	1.11 (0.72, 1.72)	1.07 (0.68, 1.66)	0.71 (0.42, 1.22)	0.50 (0.28, 0.91)	0.006	0.84 (0.73, 0.96)
Multivariable + BMI	1.00 (ref.)	1.12 (0.73, 1.71)	0.90 (0.58, 1.39)	0.74 (0.44, 1.24)	0.61 (0.34, 1.09)	0.031	0.85 (0.73, 0.99)
Multivariable + BMI + WC	1.00 (ref.)	1.07 (0.70, 1.64)	0.90 (0.59, 1.37)	0.74 (0.44, 1.26)	0.60 (0.34, 1.08)	0.034	0.85 (0.71, 1.02)
NAFLD-score, median (iqr)§	-2.1 (-2.4, -1.5)	-2.0 (-2.4, -1.4)	-2.0 (-2.4, -1.3)	-2.1 (-2.5, -1.5)	-2.1 (-2.5, -1.6)		
N cases (score≥-0.640)	41	46	51	38	32		
Unadjusted	1.00 (ref.)	1.12 (0.75, 1.67)	1.25 (0.84, 1.84)	0.93 (0.61, 1.41)	0.78 (0.50, 1.22)	0.17	0.93 (0.82, 1.05)
Multivariable ‡	1.00 (ref.)	1.13 (0.75, 1.70)	1.26 (0.85, 1.87)	1.01 (0.65, 1.56)	0.80 (0.50, 1.28)	0.28	0.95 (0.83, 1.09)
Multivariable + BMI	1.00 (ref.)	1.17 (0.78, 1.75)	1.17 (0.80, 1.71)	1.07 (0.69, 1.65)	0.95 (0.60, 1.52)	0.71	0.99 (0.86, 1.15)
Multivariable + BMI + WC	1.00 (ref.)	1.12 (0.75, 1.67)	1.17 (0.80, 1.72)	1.07 (0.70, 1.65)	0.96 (0.60, 1.53)	0.80	1.00 (0.86, 1.17)

Abbreviations: iqr, interquartile range; BMI, body mass index; WC, waist circumference; NAFLD, non-alcoholic fatty liver disease.

For peer review only - http://bmjopen.bmj.com/site/about/guidelines.xhtml

Statistical analysis using Poisson regression with robust standard errors; results are expressed as risk ratios and (95% confidence intervals).

* In categorical analysis, the population was divided into five groups by quintiles (Q1-Q5) of the Mediterranean diet score; Standard deviation was 1.20 for the multivariable analyses.

† Calculated based on an algorithm including BMI, waist circumference, triglycerides, and gamma-glutamyl transferase.

‡ Adjusted for age (years), sex, marital status (single, married/cohabiting, and widowed/Separated/divorced), occupational status (working and not working), education level (university, high school, apprenticeship, and mandatory education), smoking status (never, former, and current), energy intake (kcal/day), total energy expenditure (kcal/day), and date of dietary assessment.

§ Calculated based on an algorithm including presence of the metabolic syndrome and type 2 diabetes, and concentrations of fasting serum insulin, fasting serum aspartate-aminotransferase (AST), and the AST/alanine-aminotransferase ratio.

For peer review only

For peer review only - http://bmjopen.bmj.com/site/about/guidelines.xhtml

Figure 1 Prospective association of the Mediterranean diet score with the risk of hepatic steatosis, CoLaus study, Switzerland (n=2288): sensitivity analysis to examine influence of each component of Mediterranean diet.

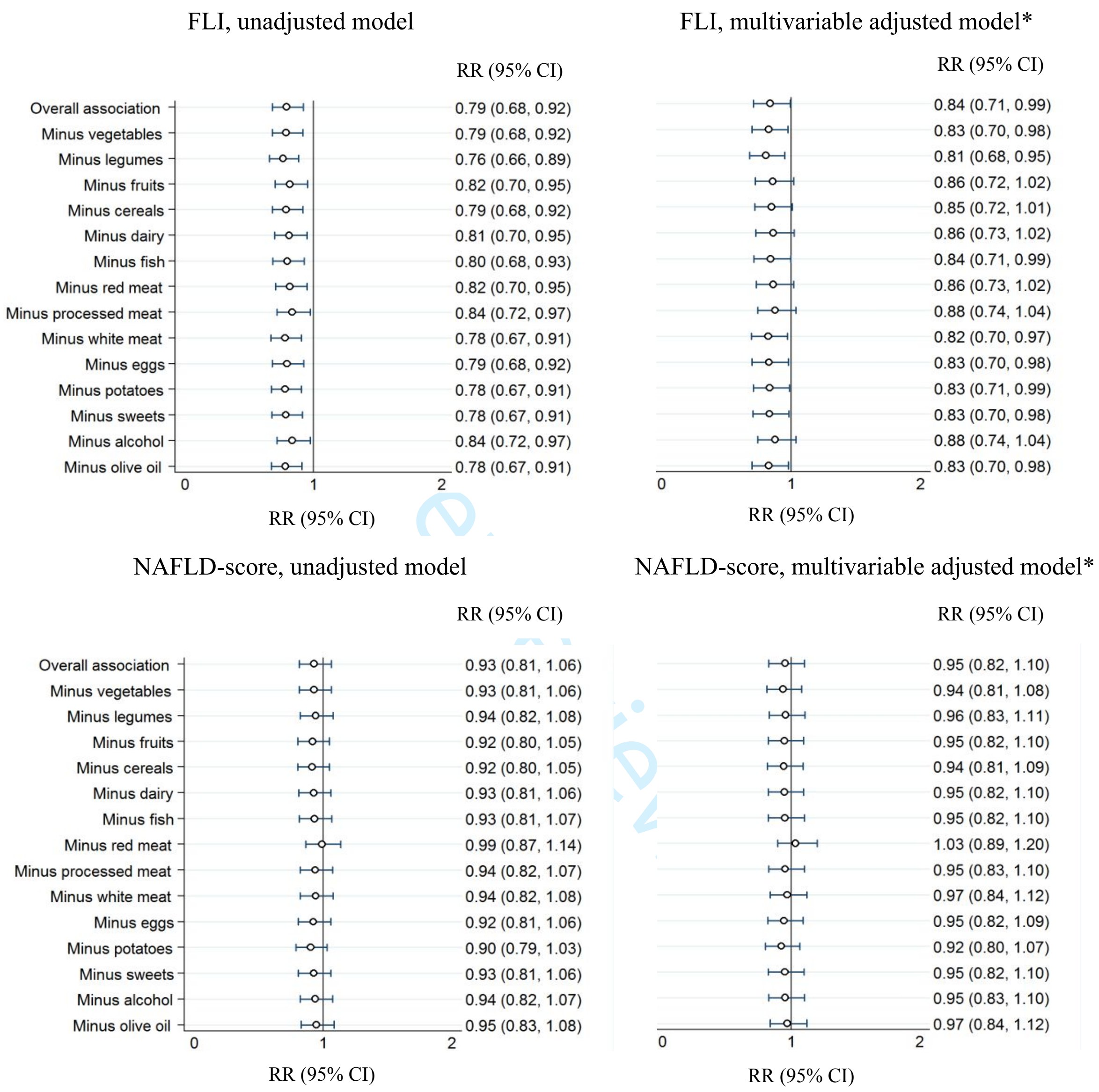

Abbreviations: FLI, fatty liver index; NAFLD, non-alcoholic fatty liver disease.

Statistical analysis using Poisson regression with robust standard errors; results are expressed as risk ratios and (95% confidence intervals).

Risk ratio (RR) and 95% confidence intervals (CI) were estimated per one standard deviation of MDS (overall association) or of each MDS computed after excluding one component.

* Adjusted for age (years), sex, marital status (single, married/cohabiting, and widowed/Separated/divorced), occupational status (working and not working), education level (university, high school, apprenticeship, and

For peer review only - http://bmjopen.bmj.com/site/about/guidelines.xhtml

mandatory education), smoking status (never, former, and current), energy intake (kcal/day), total energy expenditure (kcal/day), and date of dietary assessment.

For peer review only

For peer review only - http://bmjopen.bmj.com/site/about/guidelines.xhtml

Figure S1 Sample selection flow chart.

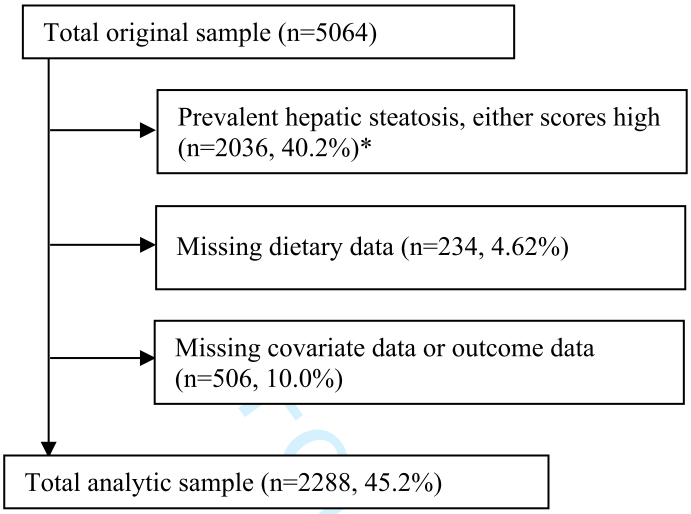

* Prevalent hepatic steatosis was defined as having either fatty liver index≥ 60 OR non-alcoholic fatty liver disease fatty liver score≥ -0.640.

For peer review only

Table S1 Characteristics of the participants included and excluded from the analysis, CoLaus study, Switzerland.

Characteristic	Included (n=2288)	Excluded (n=2776)	*P*-value*
Age, *years*	55.8±10.0	59.4±10.6	<0.001
Women (%)	65.4	43.6	<0.001
Marital status (%)†			0.038
Single	16.5	14.0	
Married/cohabiting	56.6	57.4	
Widowed/separated/divorced	26.8	28.6	
Employed (%)	63.5	50.4	<0.001
Education (%)†			<0.001
University	25.9	17.5	
High school	28.5	23.6	
Apprenticeship	33.2	37.4	
Mandatory education	12.5	21.4	
Current smoker (%)	20.1	23.1	<0.001
Alcohol consumption (%)†‡			<0.001
Abstainers	23.5	26.8	
Moderate	69.3	63.3	
Heavy	7.2	9.9	
Total energy intake (kcal/d)	1807±673	1853±784	0.033
Total protein (% energy)	15.4±3.3	15.6±3.5	0.015
Total carbohydrate (% energy)	47.0±8.6	45.4±9.1	<0.001
Total fat (% energy)	34.3±6.7	34.4±6.9	0.54
TEE (kcal/d)	2575±586	2790±669	<0.001
Metabolic syndrome (%)§	10.8	60.9	<0.001
BMI (kg/m^2)	23.7±2.8	28.3±4.8	<0.001
Waist circumference (cm)	84.4±8.9	98.3±12.5	<0.001
Triglycerides (mmol/l)	1.0±0.5	1.6±1.1	<0.001
median (iqr)	0.9 (0.7, 1.2)	1.4 (1.0, 2.0)	
GGT (U/l)	23.8±16.0	48.8±60.8	<0.001
median (iqr)	19 (14, 27)	32 (21, 53)	
≥50 (%)	5.6	27.5	<0.001
ALT (U/l)	21.8±8.8	32.5±20.7	<0.001
median (iqr)	20 (16, 25)	27 (20, 38)	
≥40 (%)	5.0	23.3	<0.001
AST (U/l)	25.9±6.2	31.6±14.1	<0.001
median (iqr)	25 (22, 29)	28 (24, 34)	
≥37 (%)	5.6	20.4	<0.001

Abbreviations: TEE, total energy expenditure; BMI, body mass index; iqr, interquartile range; GGT, gamma-glutamyl transferase; ALT, alanine aminotransferase; AST, aspartate aminotransferase.
Data are mean ± SD for continuous variables or percent for categorical variables, unless otherwise stated.
* P-value calculated using Chi-square test for categorical variables and student's t-test for continuous variables.
† Due to some missing data, percentages do not always add to 100%.
‡ Alcohol consumption categorized as "abstainers" (0 unit/week), "moderate" (1–21 units/week for men, 1–14 for women), and "heavy drinkers" (>21 units/week for men, >14 for women).
§ Metabolic syndrome defined according to the International Diabetic Federation (waist circumference ≥94 cm in men and ≥80 cm in women plus at least two of the following factors: serum triglycerides≥1.70 mmol/L or specific treatment for this lipid abnormality; serum high-density lipoprotein cholesterol <1.03 mmol/L in men and <1.29 mmol/L in women or specific treatment for this lipid abnormality; systolic blood pressure ≥130 mmHg or diastolic blood pressure ≥85 mmHg or treatment for previously diagnosed hypertension; and fasting plasma glucose ≥5.6 mmol/L or previously diagnosed type 2 diabetes).

For peer review only - http://bmjopen.bmj.com/site/about/guidelines.xhtml

Table S2 Baseline characteristics of the participants according to the risk of hepatic steatosis defined by the fatty liver index or the NAFLD-score, CoLaus study, Switzerland.

	Risk of hepatic steatosis					
	FLI			NAFLD-score		
Characteristic	No (n=2135)	Yes (n=153)	*P*-value*	No (n=2080)	Yes (n=208)	*P*-value*
Age, *years*	55.8±10.1	54.9±9.5	0.29	55.6±10	57.7±10.1	0.003
Women (%)	66.8	45.8	<0.001	66.7	52.9	<0.001
Marital status (%)†			0.86			0.79
Single	16.6	15.0		16.7	14.9	
Married/cohabiting	56.5	58.2		56.5	58.2	
Widowed/Separated/divorced	26.8	26.8		26.8	26.9	
Employed (%)	63.3	66.0	0.50	64.5	53.8	0.002
Education (%)†			0.013			0.33
University	26.7	15.0		26.4	20.7	
High school	28.3	30.1		28.3	29.8	
Apprenticeship	32.6	40.5		32.8	36.5	
Mandatory education	12.3	14.4		12.4	13.0	
Current smoker (%)	19.7	25.5	0.006	20.3	17.8	0.52
Alcohol consumption (%)†‡			0.15			0.001
Abstainers	23.3	26.1		22.5	33.7	
Moderate	69.7	63.4		70.4	58.7	
Heavy	6.9	10.5		7.1	7.7	
Total energy intake (kcal/d)	1800±655	1906±879	0.062	1794±654	1938±833	0.003
Protein (% energy)	15.3±3.1	16.1±5.0	0.006	15.3±3.2	16.0±4.1	0.006
Carbohydrate (% energy)	47.1±8.5	45.6±10.1	0.037	47.0±8.5	46.5±9.5	0.36
Fat (% energy)	34.3±6.7	33.8±7.2	0.37	34.2±6.7	34.4±6.6	0.69
TEE (kcal/d)	2552±572	2912±677	<0.001	2562±582	2699±616	0.002
Metabolic syndrome (%)§	9.9	22.9	<0.001	9.8	21.2	<0.001
BMI (kg/m^2)	23.5±2.7	26.8±2.6	<0.001	23.5±2.8	26.0±2.5	<0.001
Waist circumference (cm)	83.8±8.7	93.4±7.0	<0.001	83.7±8.7	92.0±7.4	<0.001
Triglycerides (mmol/l)	1.0±0.5	1.2±0.5	<0.001	1.0±0.5	1.2±0.5	<0.001
median (iqr)	0.9 (0.7, 1.2)	1.1 (0.7, 1.2)		0.9 (0.7, 1.2)	1.1 (0.8, 1.4)	
GGT (U/l)	23.1±15.2	32.4±22.2	<0.001	23.3±15.4	28.1±20.6	<0.001
median (iqr)	19 (14, 26)	26 (18, 40)		19 (14, 26)	22 (15, 34)	
≥50 (%)	4.9	15.0	<0.001	5.3	9.1	0.022
ALT (U/l)	21.6±8.8	23.5±8.9	0.013	21.3±8.4	26.0±11.6	<0.001
median (iqr)	19 (16, 25)	21 (17, 28)		19 (16, 25)	23 (18, 30)	
≥40 (%)	5.0	5.9	0.63	4.5	10.6	<0.001
AST (U/l)	25.9±6.2	26.3±6.3	0.50	25.9±6.2	26.9±6.2	0.026
median (iqr)	25 (22, 29)	25 (22, 29)		25 (22, 29)	26 (22, 30)	
≥37 (%)	5.5	7.2	0.39	5.3	8.2	0.096

Abbreviations: TEE, total energy expenditure; FLI, fatty liver index; NAFLD, non-alcoholic fatty liver disease; BMI, body mass index; iqr, interquartile range; GGT, gamma-glutamyl transferase; ALT, alanine aminotransferase; AST, aspartate Aminotransferase.
Data are mean ± SD for continuous variables or percent for categorical variables, unless otherwise stated.
* P-value calculated using Chi-square test for categorical variables and student's t-test for continuous variables.
† Due to some missing data, percentages do not always add to 100%.
‡ Alcohol consumption categorized as "abstainers" (0 unit/week), "moderate" (1–21 units/week for men, 1–14 for women), and "heavy drinkers" (>21 units/week for men, >14 for women).
§ Metabolic syndrome defined according to the International Diabetic Federation (waist circumference ≥94 cm in men and ≥80 cm in women plus at least two of the following factors: serum triglycerides≥1.70 mmol/L or specific treatment for this lipid abnormality; serum high-density lipoprotein cholesterol <1.03 mmol/L in men and <1.29 mmol/L in women or specific treatment for this lipid abnormality; systolic blood pressure ≥130 mm Hg or diastolic blood pressure ≥85 mm Hg or treatment for previously diagnosed hypertension; and fasting plasma glucose ≥5.6 mmol/L or previously diagnosed type 2 diabetes.

For peer review only - http://bmjopen.bmj.com/site/about/guidelines.xhtml

Table S3 Prospective association of the Mediterranean diet score with the risk of hepatic steatosis, CoLaus study, Switzerland: assessment of influence of other potential covariates.

	Risk ratio (95% CI) across quintiles of Mediterranean diet score*					*P*-trend	Risk ratio (95% CI) Per SD increase*
	Q1	Q2	Q3	Q4	Q5		
Range	1.83-7.63	7.64-8.35	8.36-8.92	8.93-9.59	9.60-12.1		
N total	458	458	457	458	457		
Fatty liver index†							
Different models							
N Cases (score≥60)	36	43	35	22	17		
Multivariable (Model 1)‡	1.00 (ref.)	1.11 (0.72, 1.72)	1.07 (0.68, 1.66)	0.71 (0.42, 1.22)	0.50 (0.28, 0.91)	0.006	0.84 (0.71, 0.99)
Model 1 + Changes in BMI categories§	1.00 (ref.)	1.11 (0.73, 1.69)	0.98 (0.64, 1.50)	0.73 (0.43, 1.24)	0.59 (0.32, 1.06)	0.024	0.85 (0.72, 1.02)
Model 1 + Alcohol	1.00 (ref.)	1.11 (0.72, 1.72)	1.06 (0.69, 1.65)	0.71 (0.41, 1.22)	0.50 (0.28, 0.91)	0.006	0.84 (0.70, 0.99)
Model 1 + Alcohol+ BMI‖	1.00 (ref.)	1.13 (0.74, 1.73)	0.92 (0.59, 1.42)	0.76 (0.45, 1.29)	0.63 (0.35, 1.14)	0.047	0.86 (0.72, 1.04)
Model 1 + Clinical variables**	1.00 (ref.)	1.30 (0.80, 2.11)	1.20 (0.74, 1.94)	0.77 (0.43, 1.39)	0.57 (0.30, 1.09)	0.023	0.84 (0.70, 1.01)
Model 1 + Clinical variables + BMI	1.00 (ref.)	1.28 (0.79, 2.07)	0.94 (0.58, 1.52)	0.80 (0.45, 1.41)	0.68 (0.36, 1.27)	0.076	0.86 (0.71, 1.04)
Model 1 + MetS††	1.00 (ref.)	1.14 (0.74, 1.75)	1.03 (0.66, 1.60)	0.68 (0.40, 1.16)	0.51 (0.28, 0.93)	0.005	0.83 (0.70, 0.99)
NAFLD-score‡‡							
Different models							
N Cases (score≥-0.640)	41	46	51	38	32		
Multivariable (Model 1)‡	1.00 (ref.)	1.13 (0.75, 1.70)	1.26 (0.85, 1.87)	1.01 (0.65, 1.56)	0.80 (0.50, 1.28)	0.28	0.95 (0.82, 1.10)
Model 1 + Changes in BMI categories§	1.00 (ref.)	1.13 (0.76, 1.69)	1.18 (0.80, 1.74)	1.04 (0.68, 1.60)	0.93 (0.58, 1.48)	0.65	0.99 (0.86, 1.16)
Model 1 + Alcohol	1.00 (ref.)	1.11 (0.73, 1.67)	1.22 (0.82, 1.82)	0.97 (0.63, 1.51)	0.77 (0.48, 1.23)	0.21	0.94 (0.81, 1.09)
Model 1 + Alcohol+ BMI‖	1.00 (ref.)	1.16 (0.78, 1.73)	1.15 (0.78, 1.68)	1.04 (0.68, 1.61)	0.93 (0.58, 1.48)	0.62	0.99 (0.85, 1.15)
Model 1 + Clinical variables**	1.00 (ref.)	1.20 (0.78, 1.84)	1.32 (0.88, 1.98)	0.99 (0.64, 1.55)	0.85 (0.52, 1.37)	0.33	0.95 (0.82, 1.11)
Model 1 + Clinical variables + BMI	1.00 (ref.)	1.21 (0.80, 1.84)	1.19 (0.80, 1.78)	1.06 (0.69, 1.64)	0.99 (0.61, 1.60)	0.76	0.99 (0.85, 1.16)

Abbreviations: SES, socio-economic status; BMI, body mass index; MetS, metabolic syndrome; NAFLD, non-alcoholic fatty liver disease; SD, standard deviation; CI, confidence interval.
Statistical analysis using Poisson regression with robust standard errors; results are expressed as risk ratios and (95% confidence intervals).
* In categorical analysis, the population was divided into five groups by quintiles (Q1-Q5) of the Mediterranean diet score; Standard deviation was 1.20 for different multivariable analyses.
† Calculated based on an algorithm including BMI, waist circumference, triglycerides, and gamma-glutamyl transferase.
‡ Adjusted for age (years), sex, marital status (single, married/cohabitant, and widowed/Separated/divorced), occupational status (working and not working), education level (university, high school, apprenticeship, and mandatory education), smoking status (never, former, and current), energy intake (kcal/day), total energy expenditure (kcal/day), and date of dietary assessment.
§ Changes in BMI categories defined as individuals with a normal BMI at baseline and follow-up, individuals with normal BMI at baseline and overweight or obese BMI at follow-up, individuals with overweight or obese BMI at baseline and at follow-up, or individuals with overweight or obese BMI at baseline and normal at follow-up.
‖ Results of further adjustment for waist circumference were broadly in line with of the further adjustment for BMI (data not shown).
**Family history of diabetes (yes/no), high blood pressure (yes/no), high triglyceride level (yes/no), low HDL level (yes/no), and high glucose level (yes/no).
†† Metabolic syndrome as defined by the International Diabetes Federation (yes/no).

For peer review only - http://bmjopen.bmj.com/site/about/guidelines.xhtml

‡‡ Calculated based on an algorithm including presence of the metabolic syndrome and type 2 diabetes, and concentrations of fasting serum insulin, fasting serum aspartate-aminotransferase (AST), and the AST/alanine-aminotransferase ratio.

For peer review only

Table S4 Sensitivity analyses for the associations of the Mediterranean diet score with the risk of hepatic steatosis, CoLaus study, Switzerland.

	Risk ratio (95% CI) across quintiles of Mediterranean diet score*						
	Q1	Q2	Q3	Q4	Q5	*P*-trend	Risk ratio (95% CI) Per SD increase*
Range	1.83-7.63	7.64-8.35	8.36-8.92	8.93-9.59	9.60-12.1		
N total	458	458	457	458	457		
Fatty liver index†							
Different models							
N Cases (score≥60)	36	43	35	22	17		
Multivariable (Model 1)‡	1.00 (ref.)	1.11 (0.72, 1.72)	1.07 (0.68, 1.66)	0.71 (0.42, 1.22)	0.50 (0.28, 0.91)	0.006	0.84 (0.71, 0.99)
Excluding alcohol from MDS component							
Model 1 + Alcohol	1.00 (ref.)	1.64 (1.04, 2.58)	1.08 (0.65, 1.80)	1.17 (0.70, 1.96)	0.65 (0.35, 1.18)	0.069	0.86 (0.73, 1.03)
Model 1 + Alcohol + BMI§	1.00 (ref.)	1.53 (0.98, 2.39)	0.94 (0.57, 1.54)	1.08 (0.67, 1.77)	0.75 (0.41, 1.35)	0.15	0.89 (0.75, 1.06)
Excluding participants with BMI≥30‖							
N Cases (score≥60)	35	40	28	19	16		
Model 1	1.00 (ref.)	1.08 (0.69, 1.68)	0.90 (0.56, 1.46)	0.64 (0.36, 1.12)	0.50 (0.27, 0.93)	0.005	0.83 (0.69, 0.98)
Model 1 + BMI§	1.00 (ref.)	1.02 (0.66, 1.58)	0.84 (0.53, 1.33)	0.65 (0.37, 1.14)	0.60 (0.33, 1.10)	0.031	0.85 (0.70, 1.02)
Excluding participants with excessive alcohol consumption**							
N Cases (score≥60)	33	42	34	21	17		
Model 1	1.00 (ref.)	1.17 (0.75, 1.84)	1.12 (0.71, 1.77)	0.70 (0.40, 1.23)	0.52 (0.28, 0.95)	0.007	0.82 (0.69, 0.98)
Model 1 + BMI§	1.00 (ref.)	1.17 (0.76, 1.81)	0.91 (0.58, 1.42)	0.73 (0.42, 1.24)	0.64 (0.35, 1.15)	0.037	0.85 (0.71, 1.02)
Excluding participants with implausible energy intake††							
N Cases (score≥60)	33	41	33	22	16		
Model 1	1.00 (ref.)	1.19 (0.63, 2.23)	1.16 (0.61, 2.20)	0.87 (0.45, 1.69)	0.69 (0.35, 1.38)	0.16	0.85 (0.71, 1.01)
Model 1 + BMI§	1.00 (ref.)	1.13 (0.60, 2.13)	0.90 (0.47, 1.73)	0.81 (0.43, 1.53)	0.74 (0.37, 1.49)	0.20	0.86 (0.72, 1.04)
Excluding participants with secondary causes of hepatic steatosis‡‡							
N Cases (score≥60)	37	42	35	22	17		
Model 1	1.00 (ref.)	1.18 (0.75, 1.85)	1.11 (0.70, 1.76)	0.78 (0.45, 1.34)	0.51 (0.27, 0.94)	0.010	0.85 (0.71, 1.01)
Model 1 + BMI§	1.00 (ref.)	1.17 (0.75, 1.83)	0.95 (0.60, 1.50)	0.78 (0.46, 1.31)	0.63 (0.34, 1.17)	0.050	0.86 (0.72, 1.04)
Excluding participants with diabetes§§							
N Cases (score≥60)	35	40	35	22	16		
Model 1	1.00 (ref.)	1.06 (0.67, 1.66)	1.11 (0.71, 1.73)	0.74 (0.43, 1.27)	0.49 (0.26, 0.91)	0.009	0.84 (0.71, 0.998)
Model 1 + BMI§	1.00 (ref.)	1.06 (0.69, 1.65)	0.92 (0.59, 1.43)	0.76 (0.45, 1.29)	0.58 (0.32, 1.06)	0.033	0.85 (0.71, 1.02)
NAFLD-score ¶¶							
Different models							
N Cases (score≥-0.640)	41	46	51	38	32		
Multivariable (Model 1)‡	1.00 (ref.)	1.13 (0.75, 1.70)	1.26 (0.85, 1.87)	1.01 (0.65, 1.56)	0.80 (0.50, 1.28)	0.28	0.95 (0.82, 1.10)

For peer review only - http://bmjopen.bmj.com/site/about/guidelines.xhtml

Excluding alcohol from MD component							
Model 1 + Alcohol	1.00 (ref.)	1.15 (0.78, 1.71)	0.92 (0.61, 1.41)	0.92 (0.59, 1.42)	0.89 (0.57, 1.38)	0.34	0.93 (0.81, 1.08)
Model 1 + Alcohol + BMI§	1.00 (ref.)	1.15 (0.78, 1.69)	0.88 (0.59, 1.32)	0.88 (0.57, 1.35)	1.02 (0.66, 1.57)	0.65	0.96 (0.83, 1.12)
Excluding participants with BMI≥30‖							
N Cases (score≥-0.640)	40	46	44	36	31		
Model 1	1.00 (ref.)	1.15 (0.76, 1.74)	1.12 (0.74, 1.69)	0.99 (0.63, 1.55)	0.81 (0.5, 1.30)	0.27	0.94 (0.81, 1.09)
Model 1 + BMI§	1.00 (ref.)	1.17 (0.78, 1.75)	1.09 (0.73, 1.63)	1.07 (0.69, 1.67)	0.97 (0.6, 1.55)	0.76	0.99 (0.85, 1.15)
Excluding participants with excessive alcohol consumption**							
N Cases (score≥-0.640)	38	43	50	37	32		
Model 1	1.00 (ref.)	1.10 (0.73, 1.68)	1.28 (0.85, 1.91)	1.00 (0.64, 1.56)	0.80 (0.5, 1.29)	0.32	0.96 (0.83, 1.11)
Model 1 + BMI§	1.00 (ref.)	1.15 (0.76, 1.74)	1.18 (0.80, 1.73)	1.06 (0.69, 1.65)	0.96 (0.6, 1.54)	0.75	1.00 (0.86, 1.17)
Excluding participants with implausible energy intake††							
N Cases (score≥-0.640)	39	46	48	38	30		
Model 1	1.00 (ref.)	1.32 (0.75, 2.35)	1.48 (0.84, 2.60)	1.06 (0.59, 1.91)	1.15 (0.64, 2.07)	0.92	0.95 (0.82, 1.10)
Model 1 + BMI§	1.00 (ref.)	1.34 (0.76, 2.38)	1.35 (0.77, 2.37)	1.06 (0.59, 1.89)	1.29 (0.72, 2.33)	0.67	0.99 (0.85, 1.16)
Excluding participants with secondary causes of hepatic steatosis‡‡							
N Cases (score≥-0.640)	41	46	50	37	31		
Model 1	1.00 (ref.)	1.19 (0.79, 1.81)	1.26 (0.84, 1.89)	1.06 (0.68, 1.65)	0.78 (0.48, 1.27)	0.25	0.95 (0.82, 1.10)
Model 1 + BMI§	1.00 (ref.)	1.23 (0.82, 1.85)	1.17 (0.79, 1.73)	1.11 (0.71, 1.72)	0.93 (0.57, 1.52)	0.66	0.99 (0.85, 1.16)
Excluding participants with diabetes§§							
N Cases (score≥-0.640)	37	43	50	36	30		
Model 1	1.00 (ref.)	1.17 (0.76, 1.79)	1.37 (0.90, 2.06)	1.05 (0.67, 1.66)	0.85 (0.52, 1.39)	0.43	0.97 (0.83, 1.12)
Model 1 + BMI§	1.00 (ref.)	1.21 (0.79, 1.85)	1.26 (0.85, 1.87)	1.11 (0.71, 1.75)	1.01 (0.62, 1.63)	0.89	1.01 (0.86, 1.18)

Abbreviations: SES, socio economic status; BMI, body mass index; MD, Mediterranean Diet; NAFLD, non-alcoholic fatty liver disease; SD, standard deviation; CI, confidence interval.

Statistical analysis using Poisson regression with robust standard errors; results are expressed as risk ratios and (95% confidence intervals).

* In categorical analysis, the population was divided into five groups by quintiles (Q1-Q5) of the Mediterranean diet score; Standard deviation was 1.41 after excluding alcohol from Mediterranean Diet score components, 1.20 after excluding participants with BMI≥30, 1.18 after excluding participants with excessive alcohol consumption, 1.20 after excluding participants with probable implausible energy intake or after excluding participants with secondary causes of hepatic steatosis or diabetes.

† Calculated based on an algorithm including BMI, waist circumference, triglycerides, and gamma-glutamyl transferase.

‡ Adjusted for age (years), sex, marital status (single, married/cohabitant, and widowed/Separated/divorced), occupational status (working and not working), education level (university, high school, apprenticeship, and mandatory education), smoking status (never, former, and current), energy intake (kcal/day), total energy expenditure (kcal/day), and date of dietary assessment.

§ Results of further adjustment for waist circumference were broadly in line with of the further adjustment for BMI (data not shown).

‖ Excluded 36 participants with BMI≥30 kg/m^2.

** Excessive alcohol consumption defined as >21 units per week for men and >14 units per weeks for women; excluded 60 participants with excess alcohol consumption (n=2228).

†† Implausible energy intake defined as <500 and <800 kcal or >3,500 or >4,000 kcal in women and men, respectively; excluded 41 participants with probable implausible energy intake (n=2247).

For peer review only - http://bmjopen.bmj.com/site/about/guidelines.xhtml

‡‡ Secondary causes of hepatic steatosis defined as having hepatitis B, C or HIV, and with hepatotoxic medications (Glucocorticoids, isoniazid, methotrexate, amiodarone, and tamoxifen); excluded 36 participants with probable secondary causes of hepatic steatosis (n=2252).
§§ Diabetes defined as glycated haemoglobin≥48 mmol/mol, or fasting plasma glucose ≥7.0 mmol/L, or use of hypoglycaemic drugs or insulin; excluded 26 participants with probable secondary causes of hepatic steatosis (n=2262).
¶¶ Calculated based on an algorithm including presence of the metabolic syndrome and type 2 diabetes, and concentrations of fasting serum insulin, fasting serum aspartate-aminotransferase (AST), and the AST/alanine-aminotransferase ratio.

For peer review only

Table S5 Prospective association of the Mediterranean diet score with the risk of hepatic steatosis, CoLaus study, Switzerland: sensitivity analysis while excluding participants with only one of the indices high at baseline.

	Risk ratio (95% CI) across quintiles of Mediterranean diet score*					*P*-trend	Risk ratio (95% CI) Per SD increase *
	Q1	Q2	Q3	Q4	Q5		
range	1.83-7.64	7.64-8.34	8.35-8.92	8.93-9.57	9.58-12.18		
N total (n=2652)	531	530	531	530	530		
Fatty liver index, *median* (iqr)†	23.4 (11.1, 41.4)	24.1 (10.6, 44.7)	22.3 (9.9, 39.7)	20.2 (8.4, 38.4)	17.5 (7.3, 34.5)		
N cases (score≥60)	51	56	54	35	24		
Unadjusted	1.00 (ref.)	1.10 (0.77, 1.58)	1.06 (0.74, 1.52)	0.69 (0.45, 1.04)	0.47 (0.29, 0.75)	<0.001	0.79 (0.69, 0.89)
Multivariable ‡	1.00 (ref.)	1.12 (0.77, 1.63)	1.25 (0.86, 1.81)	0.88 (0.57, 1.35)	0.56 (0.34, 0.92)	0.011	0.86 (0.74, 0.99)
Multivariable + BMI	1.00 (ref.)	1.08 (0.75, 1.54)	0.99 (0.70, 1.41)	0.85 (0.56, 1.30)	0.59 (0.36, 0.96)	0.017	0.84 (0.73, 0.98)
Multivariable + BMI & WC	1.00 (ref.)	1.01 (0.71, 1.45)	0.99 (0.70, 1.40)	0.84 (0.56, 1.28)	0.58 (0.35, 0.95)	0.019	0.84 (0.72, 0.98)
range	1.83-7.56	7.57-8.29	8.30-8.88	8.89-9.53	9.54-12.12		
N total (n=2568)	514	514	513	514	513		
NAFLD-score, *median* (iqr)§	-1.9 (-2.4, -1.3)	-1.8 (-2.3, -1.2)	-1.9 (-2.4, -1.3)	-2.0 (-2.5, -1.3)	-2.1 (-2.5, -1.6)		
N cases (score≥-0.640)	63	70	67	59	38		
Unadjusted	1.00 (ref.)	1.11 (0.81, 1.53)	1.07 (0.77, 1.47)	0.94 (0.67, 1.31)	0.60 (0.41, 0.89)	0.006	0.88 (0.79, 0.99)
Multivariable ‡	1.00 (ref.)	1.16 (0.83, 1.62)	1.16 (0.83, 1.61)	1.02 (0.72, 1.46)	0.66 (0.44, 1.00)	0.039	0.92 (0.81, 1.04)
Multivariable + BMI	1.00 (ref.)	1.27 (0.91, 1.76)	1.24 (0.89, 1.72)	1.16 (0.81, 1.65)	0.84 (0.55, 1.28)	0.34	0.98 (0.87, 1.11)
Multivariable + BMI & WC	1.00 (ref.)	1.25 (0.90, 1.73)	1.24 (0.89, 1.72)	1.16 (0.82, 1.65)	0.83 (0.54, 1.27)	0.34	0.99 (0.87, 1.12)

Abbreviations: iqr, interquartile range; SES, socio-economic status; BMI, body mass index; WC, waist circumference; NAFLD, non-alcoholic fatty liver disease.
Statistical analysis using Poisson regression with robust standard errors; results are expressed as risk ratios and (95% confidence intervals).
* In categorical analysis, the population was divided into five groups by quintiles (Q1-Q5) of the Mediterranean diet score; Standard deviation was 1.21 for the multivariable analyses.
† Calculated based on an algorithm including BMI, waist circumference, triglycerides, and gamma-glutamyl transferase.
‡ Adjusted for age (years), sex, marital status (single, married/cohabiting, and widowed/Separated/divorced), occupational status (working and not working), education level (university, high school, apprenticeship, and mandatory education), smoking status (never, former, and current), energy intake (kcal/day), total energy expenditure (kcal/day), and date of dietary assessment.
§ Calculated based on an algorithm including presence of the metabolic syndrome and type 2 diabetes, and concentrations of fasting serum insulin, fasting serum aspartate-aminotransferase (AST), and the AST/alanine-aminotransferase ratio.

For peer review only - http://bmjopen.bmj.com/site/about/guidelines.xhtml

Table S6 Prospective association of the Mediterranean diet score with the risk of hepatic steatosis, CoLaus study, Switzerland (n=1632), Sensitivity analysis while excluding participants with either indices high (using FLI>30 instead of FLI≥60).

	Risk ratio (95% CI) across quintiles of Mediterranean diet score*					*P*-trend	Risk ratio (95% CI) Per SD increase*
	Q1	Q2	Q3	Q4	Q5		
range	1.83-7.63	7.64-8.35	8.36-8.92	8.93-9.59	9.60-12.1		
N total	327	326	327	326	326		
Fatty liver index, median (iqr)†	14.9 (7.6, 24.8)	12.7 (7.4, 23.7)	12.3 (5.5, 22.3)	11.8 (6.3, 22.7)	10.2 (5.5, 21.8)		
N cases (score≥60)	9	5	2	2	3		
Unadjusted	1.00 (ref.)	0.56 (0.19, 1.65)	0.22 (0.05, 1.02)	0.22 (0.05, 1.02)	0.33 (0.09, 1.22)	0.047	0.66 (0.45, 0.97)
Multivariable ‡	1.00 (ref.)	0.63 (0.20, 2.00)	0.14 (0.02, 0.96)	0.27 (0.05, 1.32)	0.39 (0.09, 1.68)	0.096	0.72 (0.47, 1.10)
Multivariable + BMI	1.00 (ref.)	0.68 (0.22, 2.05)	0.13 (0.02, 0.98)	0.26 (0.06, 1.15)	0.41 (0.10, 1.70)	0.087	0.73 (0.48, 1.11)
Multivariable + BMI + WC	1.00 (ref.)	0.50 (0.18, 1.42)	0.12 (0.01, 0.98)	0.22 (0.05, 1.03)	0.39 (0.09, 1.67)	0.093	0.71 (0.46, 1.09)
NAFLD-score, median (iqr)§	-2.2 (-2.5, -1.8)	-2.2 (-2.5, -1.7)	-2.2 (-2.5, -1.8)	-2.2 (-2.6, -1.8)	-2.2 (-2.6, -1.8)		
N cases (score≥-0.640)	13	14	10	13	12		
Unadjusted	1.00 (ref.)	1.08 (0.52, 2.26)	0.77 (0.34, 1.73)	1.00 (0.47, 2.13)	0.93 (0.43, 2.00)	0.79	0.94 (0.73, 1.20)
Multivariable ‡	1.00 (ref.)	1.13 (0.55, 2.32)	0.79 (0.35, 1.76)	1.10 (0.52, 2.34)	0.98 (0.45, 2.15)	0.94	0.95 (0.73, 1.24)
Multivariable + BMI	1.00 (ref.)	1.25 (0.60, 2.60)	0.81 (0.36, 1.84)	1.20 (0.56, 2.58)	1.13 (0.51, 2.52)	0.82	0.98 (0.75, 1.28)
Multivariable + BMI + WC	1.00 (ref.)	1.17 (0.57, 2.44)	0.85 (0.37, 1.93)	1.13 (0.52, 2.45)	1.12 (0.50, 2.53)	0.84	0.98 (0.75, 1.28)

Abbreviations: iqr, interquartile range; BMI, body mass index; WC, waist circumference; NAFLD, non-alcoholic fatty liver disease.
Statistical analysis using Poisson regression with robust standard errors; results are expressed as risk ratios and (95% confidence intervals).
* In categorical analysis, the population was divided into five groups by quintiles (Q1-Q5) of the Mediterranean diet score; Standard deviation was 1.21 for the multivariable analyses.
† Calculated based on an algorithm including BMI, waist circumference, triglycerides, and gamma-glutamyl transferase.
‡ Adjusted for age (years), sex, marital status (single, married/cohabiting, and widowed/Separated/divorced), occupational status (working and not working), education level (university, high school, apprenticeship, and mandatory education), smoking status (never, former, and current), energy intake (kcal/day), total energy expenditure (kcal/day), and date of dietary assessment.
§ Calculated based on an algorithm including presence of the metabolic syndrome and type 2 diabetes, and concentrations of fasting serum insulin, fasting serum aspartate-aminotransferase (AST), and the AST/alanine-aminotransferase ratio.

For peer review only - http://bmjopen.bmj.com/site/about/guidelines.xhtml

Table S7 Association of the Mediterranean diet score with change in BMI and waist circumference, CoLaus study, Switzerland.

	β coefficient (95% CI) across quintiles of Mediterranean diet score*					*P*-trend	β coefficient (95% CI) Per SD increase*
	Q1	Q2	Q3	Q4	Q5		
range	1.83-7.63	7.64-8.35	8.36-8.92	8.93-9.59	9.60-12.1		
N total	458	458	457	458	457		
ΔBMI, mean±SD†	0.48 ± 1.62	0.61 ± 1.53	0.42 ± 1.44	0.50 ± 1.52	0.40 ± 1.52		
Multivariable ‡	1.00 (ref.)	0.12 (-0.08, 0.33)	-0.08 (-0.28, 0.13)	-0.04 (-0.25, 0.16)	-0.16 (-0.37, 0.04)	0.038	-0.08 (-0.15, -0.02)
ΔWaist circumference, mean±SD§	1.04 ± 6.53	0.74 ± 6.42	0.19 ± 6.00	0.57 ± 6.33	0.17 ± 6.19		
Multivariable ‡	1.00 (ref.)	-0.51 (-1.36, 0.34)	-0.85 (-1.69, 0.00)	-0.47 (-1.32, 0.38)	-0.82 (-1.68, 0.03)	0.10	-0.33 (-0.61, -0.06)

Abbreviations: BMI, Body mass index.

Statistical analysis using linear regression; results are expressed as β coefficients and (95% confidence intervals).

* In categorical analysis, the population was divided into five groups by quintiles (Q1-Q5) of the Mediterranean diet score; Standard deviation was 1.20 for the multivariable analyses.

† Calculated by subtracting BMI at baseline from BMI at follow-up.

‡ Adjusted for age (years), sex, marital status (single, married/cohabitant, and widowed/Separated/divorced), occupational status (working and not working), education level (university, high school, apprenticeship, and mandatory education), smoking status (never, former, and current), energy intake (kcal/day), total energy expenditure (kcal/day), and date of dietary assessment.

§ Calculated by subtracting waist circumference at baseline from waist circumference at follow-up.

For peer review only

Table S8 Association of the Mediterranean diet score with the GGT, ALT, and AST, CoLaus study, Switzerland.

	β coefficient (95% CI) across quintiles of Mediterranean diet score*					*P*-trend	β coefficient (95% CI) Per SD increase *
	Q1	Q2	Q3	Q4	Q5		
range	1.83-7.63	7.64-8.35	8.36-8.92	8.93-9.59	9.60-12.12		
N total	458	458	457	458	457		
GGT (U/l), *median* (iqr)	21 (15, 29)	20 (14, 29)	19 (14, 26)	18 (14, 28)	17 (13, 26)		
Multivariable †	1.00 (ref.)	-2.63 (-9.01, 3.76)	-5.71 (-12.07, 0.66)	-4.24 (-10.64, 2.16)	-6.03 (-12.45, 0.38)	0.063	-1.53 (-3.60, 0.53)
Multivariable + BMI	1.00 (ref.)	-2.86 (-9.24, 3.53)	-5.70 (-12.06, 0.66)	-4.50 (-10.90, 1.90)	-6.52 (-12.95, -0.09)	0.047	-1.66 (-3.73, 0.41)
Multivariable + BMI & WC	1.00 (ref.)	-2.93 (-9.32, 3.45)	-5.72 (-12.08, 0.64)	-4.54 (-10.94, 1.86)	-6.53 (-12.96, -0.10)	0.047	-1.65 (-3.72, 0.41)
ALT (U/l), *median* (iqr)	20 (16, 26)	20.5 (17, 26)	20 (16, 26)	19.5 (16, 25)	20 (16, 25)		
Multivariable †	1.00 (ref.)	0.03 (-0.02, 0.08)	0.03 (-0.02, 0.08)	-0.013 (-0.06, 0.04)	0.002 (-0.05, 0.05)	0.45	-0.006 (-0.02, 0.01)
Multivariable + BMI	1.00 (ref.)	0.04 (-0.01, 0.08)	0.03 (-0.02, 0.08)	-0.009 (-0.06, 0.04)	0.008 (-0.04, 0.06)	0.60	-0.005 (-0.02, 0.01)
Multivariable + BMI & WC	1.00 (ref.)	0.04 (-0.01, 0.08)	0.03 (-0.02, 0.08)	-0.01 (-0.06, 0.04)	0.008 (-0.04, 0.06)	0.60	-0.005 (-0.02, 0.01)
AST (U/l), *median* (iqr)	22 (19, 25)	22 (19, 26)	22 (19, 25)	22 (19, 26)	22 (19, 25)		
Multivariable †	1.00 (ref.)	0.02 (-0.01, 0.06)	0.004 (-0.03, 0.04)	0.011 (-0.03, 0.05)	0.015 (-0.02, 0.05)	0.66	0.003 (-0.01, 0.01)
Multivariable + BMI	1.00 (ref.)	0.02 (-0.02, 0.06)	0.004 (-0.03, 0.04)	0.009 (-0.03, 0.04)	0.011 (-0.03, 0.05)	0.80	0.002 (-0.01, 0.01)
Multivariable + BMI & WC	1.00 (ref.)	0.02 (-0.02, 0.06)	0.004 (-0.03, 0.04)	0.009 (-0.03, 0.04)	0.011 (-0.03, 0.05)	0.80	0.002 (-0.01, 0.01)

Abbreviations: iqr, interquartile range; GGT, gamma-glutamyl transferase; SES, socio economic status; BMI, body mass index; WC, waist circumference; ALT, Alanine transaminase; AST, aspartate-aminotransferase.

Statistical analysis using linear regression; results are expressed as β coefficients and (95% confidence intervals).

* In categorical analysis, the population was divided into five groups by quintiles (Q1-Q5) of the Mediterranean diet score; Standard deviation was 1.20 for the multivariable analyses.

† Adjusted for age (years), sex, marital status (single, married/cohabitant, and widowed/Separated/divorced), occupational status (working and not working), education level (university, high school, apprenticeship, and mandatory education), smoking status (never, former, and current), energy intake (kcal/day), total energy expenditure (kcal/day), and date of dietary assessment.

For peer review only

For peer review only - http://bmjopen.bmj.com/site/about/guidelines.xhtml

STROBE 2007 (v4) Statement—Checklist of items that should be included in reports of *cohort studies*

Section/Topic	Item #	Recommendation	Reported on page #
**Title and abstract**	1	(*a*) Indicate the study's design with a commonly used term in the title or the abstract	3
		(*b*) Provide in the abstract an informative and balanced summary of what was done and what was found	3
**Introduction**			
Background/rationale	2	Explain the scientific background and rationale for the investigation being reported	6
Objectives	3	State specific objectives, including any prespecified hypotheses	7
**Methods**			
Study design	4	Present key elements of study design early in the paper	7
Setting	5	Describe the setting, locations, and relevant dates, including periods of recruitment, exposure, follow-up, and data collection	7
Participants	6	(*a*) Give the eligibility criteria, and the sources and methods of selection of participants. Describe methods of follow-up	7; Figure S1, and table S1
		(*b*) For matched studies, give matching criteria and number of exposed and unexposed	N/A
Variables	7	Clearly define all outcomes, exposures, predictors, potential confounders, and effect modifiers. Give diagnostic criteria, if applicable	Pages 7 to 10
Data sources/ measurement	8*	For each variable of interest, give sources of data and details of methods of assessment (measurement). Describe comparability of assessment methods if there is more than one group	Pages 7 to 10
Bias	9	Describe any efforts to address potential sources of bias	Pages 10 & 11
Study size	10	Explain how the study size was arrived at	12; Figure S1
Quantitative variables	11	Explain how quantitative variables were handled in the analyses. If applicable, describe which groupings were chosen and why	Pages 7 to 10
Statistical methods	12	(*a*) Describe all statistical methods, including those used to control for confounding	Pages 10 & 11
		(*b*) Describe any methods used to examine subgroups and interactions	Pages 10 & 11
		(*c*) Explain how missing data were addressed	Pages 10 & 11
		(*d*) If applicable, explain how loss to follow-up was addressed	Pages 10 & 11
		(*e*) Describe any sensitivity analyses	Pages 10 & 11

For peer review only

**Results**			
Participants	13*	(a) Report numbers of individuals at each stage of study—eg numbers potentially eligible, examined for eligibility, confirmed eligible, included in the study, completing follow-up, and analysed	12
		(b) Give reasons for non-participation at each stage	N/A
		(c) Consider use of a flow diagram	Figure 1
Descriptive data	14*	(a) Give characteristics of study participants (eg demographic, clinical, social) and information on exposures and potential confounders	12; Table 1 and table S1 & S2
		(b) Indicate number of participants with missing data for each variable of interest	12
		(c) Summarise follow-up time (eg, average and total amount)	12
Outcome data	15*	Report numbers of outcome events or summary measures over time	12; Table S2
Main results	16	(*a*) Give unadjusted estimates and, if applicable, confounder-adjusted estimates and their precision (eg, 95% confidence interval). Make clear which confounders were adjusted for and why they were included	Pages 12 & 13; Table 2, Figure 1, and supplementary Tables and Figures
		(*b*) Report category boundaries when continuous variables were categorized	Pages 12 & 13; Table 2, Figure 1, and supplementary Tables and Figures
		(*c*) If relevant, consider translating estimates of relative risk into absolute risk for a meaningful time period	
Other analyses	17	Report other analyses done—eg analyses of subgroups and interactions, and sensitivity analyses	Pages 13 & 14; Supplementary Tables S3 to S8
**Discussion**			
Key results	18	Summarise key results with reference to study objectives	14
**Limitations**	19	Discuss limitations of the study, taking into account sources of potential bias or imprecision.	pages 17 & 18
Interpretation	20	Give a cautious overall interpretation of results considering objectives, limitations, multiplicity of analyses, results from similar studies, and other relevant evidence	Pages 14 to 18
Generalisability	21	Discuss the generalisability (external validity) of the study results	pages 17 & 18
**Other information**			
Funding	22	Give the source of funding and the role of the funders for the present study and, if applicable, for the original study on	2

For peer review only

		which the present article is based	

*Give information separately for cases and controls in case-control studies and, if applicable, for exposed and unexposed groups in cohort and cross-sectional studies.

Note: An Explanation and Elaboration article discusses each checklist item and gives methodological background and published examples of transparent reporting. The STROBE checklist is best used in conjunction with this article (freely available on the Web sites of PLoS Medicine at http://www.plosmedicine.org/, Annals of Internal Medicine at http://www.annals.org/, and Epidemiology at http://www.epidem.com/). Information on the STROBE Initiative is available at www.strobe-statement.org.

For peer review only

BMJ Open

The association between adherence to the Mediterranean diet and hepatic steatosis: the Swiss CoLaus prospective study

Journal:	*BMJ Open*
Manuscript ID	bmjopen-2020-040959.R1
Article Type:	Original research
Date Submitted by the Author:	15-Sep-2020
Complete List of Authors:	Khalatbari-Soltani, Saman; The University of Sydney School of Public Health, Faculty of Medicine and Health; ARC Centre for Excellence in Population Ageing Research<br>Marques-Vidal, Pedro; University Hospital of Lausanne, Department of Internal Medicine<br>Imamura, Fumiaki; University of Cambridge School of Clinical Medicine, Medical Research Council Epidemiology Unit<br>Forouhi, Nita; University of Cambridge School of Clinical Medicine, Medical Research Council Epidemiology Unit
**Primary Subject Heading**:	Epidemiology
Secondary Subject Heading:	Nutrition and metabolism, Gastroenterology and hepatology, Public health
Keywords:	NUTRITION & DIETETICS, EPIDEMIOLOGY, PUBLIC HEALTH, Hepatology < INTERNAL MEDICINE

SCHOLARONE™
Manuscripts

I, the Submitting Author has the right to grant and does grant on behalf of all authors of the Work (as defined in the below author licence), an exclusive licence and/or a non-exclusive licence for contributions from authors who are: i) UK Crown employees; ii) where BMJ has agreed a CC-BY licence shall apply, and/or iii) in accordance with the terms applicable for US Federal Government officers or employees acting as part of their official duties; on a worldwide, perpetual, irrevocable, royalty-free basis to BMJ Publishing Group Ltd ("BMJ") its licensees and where the relevant Journal is co-owned by BMJ to the co-owners of the Journal, to publish the Work in this journal and any other BMJ products and to exploit all rights, as set out in our licence.

The Submitting Author accepts and understands that any supply made under these terms is made by BMJ to the Submitting Author unless you are acting as an employee on behalf of your employer or a postgraduate student of an affiliated institution which is paying any applicable article publishing charge ("APC") for Open Access articles. Where the Submitting Author wishes to make the Work available on an Open Access basis (and intends to pay the relevant APC), the terms of reuse of such Open Access shall be governed by a Creative Commons licence – details of these licences and which Creative Commons licence will apply to this Work are set out in our licence referred to above.

Other than as permitted in any relevant BMJ Author's Self Archiving Policies, I confirm this Work has not been accepted for publication elsewhere, is not being considered for publication elsewhere and does not duplicate material already published. I confirm all authors consent to publication of this Work and authorise the granting of this licence.

For peer review only

TITLE PAGE

Title: The association between adherence to the Mediterranean diet and hepatic steatosis: the Swiss CoLaus prospective study

Saman Khalatbari-Soltani[1,2,3], Pedro Marques-Vidal[3], Fumiaki Imamura[4*], and Nita G. Forouhi[4*]

[1] The University of Sydney School of Public Health, Faculty of Medicine and Health, New South Wales, Australia; [2] ARC Centre of Excellence in Population Ageing Research (CEPAR), University of Sydney, Sydney, Australia; [3] Department of Internal Medicine, Internal Medicine, Lausanne University Hospital (CHUV), rue du Bugnon 46, 1011 Lausanne, Switzerland; [4] Medical Research Council Epidemiology Unit, University of Cambridge, School of Clinical Medicine, Institute of Metabolic Science, Cambridge, CB2 0QQ, UK.

*FL and NGF are joint senior authors with equal contribution

Correspondence to:

Saman Khalatbari-Soltani, ARC Centre of Excellence in Population Ageing Research (CEPAR), School of Public Heath, Faculty of Medicine and Health, the University of Sydney, NSW, 2006, Sydney Australia, +61 (0) 431 711 144, saman.khalatbarisoltani@sydney.edu.au.

Nita G. Forouhi, Medical Research Council Epidemiology Unit, University of Cambridge, School of Clinical Medicine, Institute of Metabolic Science, Cambridge, CB2 0QQ, UK, +44 (0) 1223 769145, nita.forouhi@mrc-epid.cam.ac.uk.

Running title: Mediterranean diet and hepatic steatosis.

Word count: 4026

For peer review only - http://bmjopen.bmj.com/site/about/guidelines.xhtml

Number of figures and tables: Two tables and one figure

For peer review only

For peer review only - http://bmjopen.bmj.com/site/about/guidelines.xhtml

ABSTRACT

Objective: The Mediterranean diet has been promoted as a healthy dietary pattern but, whether the Mediterranean diet may help to prevent hepatic steatosis is not clear. This study aimed to evaluate the prospective association between adherence to the Mediterranean diet and risk of hepatic steatosis.

Design: Population-based prospective cohort study.

Setting: The Swiss CoLaus study.

Participants: We evaluated 2288 adults (65.4% women, aged 55.8±10.0 years) without hepatic steatosis at first follow-up in 2009-2012. Adherence to the Mediterranean diet was scaled as the Mediterranean diet score (MDS) based on the Mediterranean-diet Pyramid ascertained with responses to food-frequency questionnaires.

Outcome measures: New onset of hepatic steatosis was ascertained by two indices separately: the fatty liver index (FLI, ≥60 points) and the non-alcoholic fatty liver disease (NAFLD) score (≥-0.640 points). Prospective associations between adherence to the Mediterranean diet and risk of hepatic steatosis were quantified using Poisson regression.

Results: During a mean 5.3-years of follow-up, hepatic steatosis was ascertained in 153 (6.7%) participants by FLI criteria and in 208 (9.1%) by NAFLD-score. After multivariable adjustment, higher adherence to MDS was associated with lower risk of hepatic steatosis based on FLI: risk ratio (95% confidence interval) 0.84 (0.73, 0.96) per one standard deviation of MDS; 0.85 (0.73, 0.99) adjusted for BMI; and 0.85 (0.71, 1.02) adjusted for both BMI and waist circumference. When using NAFLD-score, no significant association was found between MDS and risk of hepatic steatosis [0.95 (0.83, 1.09)].

Conclusion: A potential role of the Mediterranean diet in the prevention of hepatic steatosis is suggested by the inverse association observed between adherence to the Mediterranean diet and incidence of hepatic steatosis based on the FLI. The inconsistency of this association

For peer review only - http://bmjopen.bmj.com/site/about/guidelines.xhtml

when hepatic steatosis was assessed by NAFLD-score points to the need for accurate population-level assessment of fatty liver and its physiologic markers.

Keywords: Mediterranean diet; hepatic steatosis; fatty liver disease; prospective study.

For peer review only

For peer review only - http://bmjopen.bmj.com/site/about/guidelines.xhtml

STRENGHTS AND LIMITATION OF THE STUDY

- This study had the benefit of a relatively large sample size and an average of 5.3 years of follow-up.
- We applied a definition of the Mediterranean diet that has been shown to be valid in a non-Mediterranean population.
- Our ascertainment of hepatic steatosis was based on two indices that has been validated for use in large epidemiological studies.
- The precision of the two hepatic steatosis indices used is different and may be influenced by the presence of steatohepatitis or advanced liver fibrosis.
- Generalisability is limited because our findings relate to a single European population.

For peer review only

For peer review only - http://bmjopen.bmj.com/site/about/guidelines.xhtml

INTRODUCTION

Hepatic steatosis is the most common cause of liver disease [1]. In westernized countries, hepatic steatosis affects up to 34% of the general population and up to 74% of obese individuals, depending on the definition used [2–4]. Hepatic steatosis—fat content of more than 5% of liver volume—is the first recognizable stage of non-alcoholic fatty liver disease (NAFLD) [1]. Hepatic steatosis, particularly NAFLD, may progress to end-stage liver disease including fibrosis, cirrhosis, and hepatocellular carcinoma [5]. Moreover, as hepatic steatosis increases the risk of metabolic syndrome, type 2 diabetes, and cardiovascular disease (CVD), its prevention is of public health importance [6]. An unhealthy dietary pattern remains one of the primary targets of lifestyle modification for the prevention and management of hepatic steatosis and NAFLD [7,8].

The Mediterranean diet has been recently recommended for treatment of NAFLD [9]. In recent years, a growing body of evidence supports the idea that the Mediterranean diet may be the reference nutritional profile for the prevention of hepatic steatosis development [10–12]. Adherence to the Mediterranean diet has been reported to have a beneficial impact on risks of CVD [13,14], type 2 diabetes [15], and metabolic syndrome [16]. Trial evidence demonstrated the potential benefits of the Mediterranean diet against progress of hepatic steatosis focusing on individuals with existing hepatic steatosis, either alone [17–22] or associated with metabolic risk factors such as obesity or diabetes [22–25]. Research among those without clinically manifest hepatic steatosis is restricted to observational evidence, reporting an inverse association that greater adherence to a Mediterranean diet is associated with lower prevalence of hepatic steatosis [26,27]. However, the cross-sectional design of these studies limits inference for causal associations and can be used mainly for hypothesis generation. Relevant longitudinal evidence for the primary prevention of hepatic steatosis or NAFLD has been reported only by the Framingham Heart Study, with a significant inverse

For peer review only - http://bmjopen.bmj.com/site/about/guidelines.xhtml

association of adherence to the Mediterranean diet with risk of hepatic steatosis in 1521 adults over 6 years of follow-up [28], but evidence is lacking in Europe.

Given the limited evidence from population-based epidemiological studies thus far, we aimed to investigate the prospective association between adherence to the Mediterranean diet and the risk of developing hepatic steatosis among adults without clinically manifest hepatic steatosis. We hypothesized that greater adherence to the Mediterranean diet would reduce the risk of hepatic steatosis.

METHODS

Study population

We evaluated participants in the CoLaus study, an ongoing population-based cohort investigating the clinical, biological, and genetic determinants of CVD in the city of Lausanne, Switzerland [29]. Inclusion criteria of the recruitment were adults of European origin, aged 35 to 75 years [29]. There were three study phases: baseline recruitment in 2003-2006 (n=6733), the first follow-up in 2009-2012 (n=5064), and the second follow-up in 2014-2017 (n=4881). We conducted dietary assessment at the first follow-up and therefore considered the first follow-up as the study baseline. Fatty liver index (FLI) and NAFLD-score, two indices of hepatic steatosis, were available at baseline [27]. If participants met the joint criterion of FLI $\geq$ 60 or NAFLD-score $\geq$ -0.640 at baseline, we excluded them as prevalent cases (see below) (n=2036). We also excluded participants with missing information on diet, outcome, and covariates (n=740) (**Figure S1**).

Patient and public involvement

Patients and/or the public were not involved in the design, conduct, reporting or dissemination plans of this research.

For peer review only - http://bmjopen.bmj.com/site/about/guidelines.xhtml

For peer review only

Dietary assessment

Participants completed a self-administered, 97-item, semi-quantitative food frequency questionnaire (FFQ) about their habitual dietary intake over the last four weeks [30], the validity of which had been assessed in canton Geneva against 24-hour recalls [30,31]. For each item, participants were instructed to report consumption frequencies by selecting one of the seven frequency options from "less than once during the last 4 weeks" to "2 or more times per day" and by selecting a usual serving size (smaller, equal, or bigger to a reference size).

Mediterranean diet scores

We derived the pyramid-based Mediterranean diet score (MDS) as a measure of adherence to the Mediterranean diet from responses to the FFQ as we conducted previously [27]. This MDS is based on the Mediterranean Dietary Pyramid proposed by the Mediterranean Diet Foundation for both Mediterranean and non-Mediterranean countries and accounting for the traditional Mediterranean diet, contemporary lifestyle, and food environment [32]. We have previously reported that this MDS scoring algorithm predicted CVD incidence [33], as well as the prevalence of hepatic steatosis [27] in non-Mediterranean populations. Briefly, a continuous score of 0 to 1 was assigned for each recommended level of the 15 components of the pyramid (vegetables, legumes, and fish as healthy items; red meat, processed meat, potato, and sweets as unhealthy items; and fruits, nuts, cereals, eggs, dairy, white meat, and alcoholic beverages as items for which moderate consumption was recommended). The resulting MDS ranges between 0 and 15 on a continuous scale. The MDS calculation was adjusted to an energy intake of 2000 kcal/d (8.37 MJ/day) by applying a regression-residual technique for energy adjustment to each food group variable [33,34].

Ascertainment of hepatic steatosis

For peer review only

For peer review only - http://bmjopen.bmj.com/site/about/guidelines.xhtml

Two indices of hepatic steatosis were evaluated: fatty liver index (FLI) [35] and NAFLD liver fat score [36]. FLI was calculated based on a logistic function including body-mass index (BMI), waist circumference, fasting triglycerides, and gamma-glutamyl transferase (GGT) levels as follows:

$$FLI = 1 / (1+e^{-(0.953 \times \ln (\text{triglycerides}) + 0.139 \times BMI + 0.718 \times \ln (GGT) + 0.053 \times \text{waist circumference} - 15.745)})$$

FLI×100 ranges from 0 to 100. Presence of hepatic steatosis was defined by FLI ≥ 60, a value with a sensitivity of 61% and a specificity of 86% [35]. FLI was tested previously in comparison to ultrasonography with an area under the receiver operating characteristic curve of 0.78 (OR 95% CI: 0.77, 0.83) [37].

The NAFLD-score was calculated based on an algorithm including a logistic function with the presence of metabolic syndrome defined by criteria of International Diabetes Federation [38], presence of type 2 diabetes, and fasting concentrations of insulin, aspartate-aminotransferase (AST), and the AST/ alanine transaminase (ALT) ratio:

$$\text{NAFLD-score} = -2.89 + 1.18 \times \text{metabolic syndrome (yes/no)} + 0.45 \times \text{type 2 diabetes (yes /no)} + 0.15 \times \text{fasting-insulin (mU/L)} + 0.04 \times \text{fasting-AST (U/L)} - 0.94 \times AST/ALT$$

Presence of hepatic steatosis was defined by a NAFLD-score ≥ -0.640, a value with a sensitivity of 86% and a specificity of 71%, when compared to proton magnetic resonance imaging [36].

Assessment of covariates at baseline

Sociodemographic, lifestyle and health characteristics were collected by self-administered questionnaires. Age, sex, marital status, occupational status, and educational level were included as indicators of sociodemographic condition. Smoking status was classified as ‘never’, ‘former’, and ‘current’. Alcohol consumption was assessed by the

For peer review only - http://bmjopen.bmj.com/site/about/guidelines.xhtml

number of alcoholic beverage units consumed in the past week and further categorized as ‘abstainers’ (0 unit/week), ‘moderate’ (1–21 units/week for men, 1–14 for women), and ‘heavy’ (>21 units/week for men, >14 for women) drinkers (one unit corresponds to 8 g of alcohol). Physical activity was assessed with a self-administered quantitative physical activity frequency questionnaire [39]. Health characteristics included presence of metabolic syndrome and family history of diabetes. Anthropometric and blood pressure measurements were obtained using standard procedures and equipment as previously described [29]. Plasma triglycerides, high-density lipoprotein cholesterol, and glucose were measured using standard enzymatic methods and ALT, AST, and GGT were measured using reference methods as standardized by the International Federation of Clinical Chemistry.

Statistical analysis

Statistical analyses were performed using Stata (version 15; StataCorp, College Station, TX, USA) with a two-sided test with $\alpha=0.05$. Descriptive statistics were obtained in the participants included in this study in comparison to those excluded from this study. Cohen’s kappa statistics were calculated to assess the agreement between the FLI and NAFLD-score.

MDS as a measure of adherence to the Mediterranean diet was evaluated both categorically (quintiles) and continuously scaled as one SD unit. The association of MDS with the risk of hepatic steatosis was assessed using multivariable-adjusted Poisson regression models with robust standard errors and estimating risk ratios (RRs) and 95% confidence intervals (CIs). Models were adjusted for age, sex, marital status, occupational status, educational level, smoking status, energy intake, total energy expenditure, and date of dietary assessment (to adjust for seasonality). We further adjusted for BMI and waist circumference as potential confounders or factors on the causal pathway to assess the possible impact of

For peer review only - http://bmjopen.bmj.com/site/about/guidelines.xhtml

overall and central adiposity on the association of the Mediterranean diet and hepatic steatosis.

Additionally, we also adjusted for changes in BMI categories between baseline and follow-up; for alcohol consumption (units/week); for clinical variables of metabolic risk [blood pressure >130/85 mm Hg (yes/no), triglycerides >1.7 mmol/L (yes/no), high-density lipoprotein level <1.29 mmol/L for men and <1.03 mmol/L for women (yes/no), and glucose level ≥5.6 mmol/L (yes/no)] [38]; and for family history of diabetes and metabolic syndrome (only for FLI) to examine their influence on the association of interest.

Possible interactions between MDS and age, sex, BMI, and alcohol consumption were tested using the Wald test. Several sensitivity analyses were conducted to examine the robustness of the observed findings. First, to assess the role of alcohol consumption (as alcohol is a risk factor for fatty liver accumulation), we excluded the alcohol component from the MDS, while adjusting for alcohol consumption as a covariate. We took the same approaches for the other MDS components to assess the impact of each component on the observed associations. Second, we conducted separate analyses after excluding participants with BMI≥30 kg/m^2; implausible energy intake (<500 or >3500 kcal/day in women and <800 or >4000 kcal/day in men); excessive alcohol consumption; prevalent diabetes (defined as glycated haemoglobin≥48 mmol/mol, or fasting plasma glucose ≥7.0 mmol/L, or use of hypoglycaemic drugs or insulin); or probable secondary causes of hepatic steatosis such as hepatitis B or C, HIV, hepatotoxic, or autoimmune disease medications. We evaluated the robustness of the results to an alternative definition of prevalent hepatic steatosis. While we excluded participants with prevalent hepatic steatosis using the specified cut-offs of FLI or NAFLD-score in the primary analysis, we used each of the two indices separately in sensitivity analyses, whereby we evaluated 2652 adults in longitudinal analysis based on FLI;

For peer review only

For peer review only - http://bmjopen.bmj.com/site/about/guidelines.xhtml

and 2568 adults, based on NAFLD. Finally, we used more restrictive cutpoints and excluded participants with NAFLD-score ≥-0.640 or with FLI>30.

In a post-hoc analysis, due to inconsistency of the associations observed for FLI and NAFLD-score, we also calculated the hepatic steatosis index (HSI) [40] based on the ratio of AST/ALT, BMI, presence of type 2 diabetes, and sex:

$$HSI = 8*AST/ALT + BMI + 2\ (\text{presence of diabetes}) + 2\ (\text{if women})$$

Presence of hepatic steatosis was defined by a HSI>36. After excluding participants with HSI>36 at baseline (n=2674), we evaluated 2351 adults.

In a post-hoc analysis pertaining to sensitivity of the results to model covariates and for better understanding of potential mechanisms, longitudinal associations of MDS with follow-up measures of log-transformed GGT, ALT, and AST levels and with changes in BMI and waist circumference from baseline to follow-up were examined using multivariable-adjusted linear regression. These results were expressed as β coefficient (95% CIs) for changes in each measure per 1-SD difference in MDS.

RESULTS

Participant characteristics

Of the initial 5064 participants, 2776 (54.8%) were excluded, leaving 2288 participants (65.4% women; 55.8±10.0 years) for analysis. Participants included were more likely to be women, show higher sociodemographic characteristics and lower BMI, waist circumference, liver enzymes, or prevalence of metabolic syndrome in comparison to excluded individuals (**Table S1**). Being in the highest quintile of MDS was higher among women compared to men, positively correlated with sociodemographic characteristics, and

For peer review only

For peer review only - http://bmjopen.bmj.com/site/about/guidelines.xhtml

negatively correlated with being current smokers, heavy alcohol drinkers, and with BMI, waist circumference, GGT, and TG (**Table 1**).

Adherence to the Mediterranean diet and risk of hepatic steatosis

After a mean 5.3 (SD: 0.5) years of follow-up, there were 153 (6.7%) and 208 (9.1%) participants with hepatic steatosis based on FLI and NAFLD-score, respectively (**Table S2**). Case identification by FLI and NAFLD-score was modestly concordant (kappa=0.60).

Multivariable-adjusted analysis showed an inverse association between MDS quintiles and risk of hepatic steatosis based on FLI (p_{trend}<0.006) with RR (95% CI) comparing the top to the bottom category of 0.50 (0.28, 0.91). The inverse associations across quintiles of MDS weakened after adjustment for BMI (p_{trend}=0.031) or both BMI and waist circumference (p_{trend}=0.034) (**Table 2**): RR (95% CI)=0.61 (0.34, 1.09) and 0.60 (0.34, 1.08), respectively. In analyses using MDS as a continuous variable, the inverse association with risk of hepatic steatosis based on FLI [RR 0.84 (0.73, 0.96) per one SD of MDS] remained unchanged but getting imprecise after adjustment for BMI [RR 0.85 (0.73, 0.99)] and adjustment for both BMI and waist circumference [0.85 (0.71, 1.02)]. In sensitivity analysis, the magnitude of the inverse associations little changed after further adjustment for alcohol consumption, presence of metabolic syndrome, changes in BMI categories or clinical variables (medication use or prevalent diseases), while adjustment for BMI and clinical variables increased standard errors (**Table S3**).

Conversely, there was no association between MDS and the risk of hepatic steatosis defined by NAFLD-score criteria, with RRs (95% CIs) ranging from 0.93 (0.82, 1.05) to 1.00 (0.86, 1.17) over different regression models (**Table 2** and **Table S3**).

Interaction and sensitivity analyses

For peer review only - http://bmjopen.bmj.com/site/about/guidelines.xhtml

No significant interactions were found between MDS and age, sex, BMI, or alcohol consumption on risk of hepatic steatosis ($p_{interaction}$>0.05; results not shown). The contribution of each component of the MDS on risk of hepatic steatosis was assessed by sequential subtraction of components from the score (**Figure 1**). Excluding the components of the MDS did not substantially affect the inverse associations with hepatic steatosis based on FLI; the magnitude of the associations remained reasonably stable, but it became weaker (p>0.05) after excluding fruits, cereals, dairy products, red or processed meat, or alcohol.

In sensitivity analyses, when excluding the alcohol component from the MDS but adjusting for alcohol consumption as a covariate, the inverse associations between MDS and risk of hepatic steatosis based on FLI became weaker (**Table S4**). The primary results were not different when excluding participants with BMI≥30 kg/m^2, excessive alcohol consumption, or secondary causes of hepatic steatosis (**Table S4**). Excluding participants with implausible energy intakes weakened the associations (**Table S4**). The analysis of an alternative definition of prevalent hepatic steatosis, excluding participants with only high FLI score at baseline, did not alter the significant inverse association between MDS and risk of FLI-based hepatic steatosis (**Table S5**). In post-hoc analyses, there was an inverse association between MDS quintiles and risk of hepatic steatosis based on HSI (p_{trend}=0.070) with RR (95% CI) comparing the top to the bottom category of 0.70 (0.55, 0.91); a significant inverse association with HSI was observed per one SD increase in MDS [RR 0.90 (0.82, 0.98)] (**Table S5**). Effect sizes were of slightly higher magnitude when excluding those with FLI>30 or NAFLD-score≥-0.640 at baseline, but CIs were wider due to smaller sample size (**Table S6**).

For NAFLD-score, no significant associations were found in any of the sensitivity analyses (**Tables S4 and S5** and **Figure S2**). The sole exception was when participants with a high NAFLD-score at baseline were excluded, where an inverse association between MDS

For peer review only - http://bmjopen.bmj.com/site/about/guidelines.xhtml

quintiles and risk of hepatic steatosis was present (p_{trend}=0.039), but this association was attenuated to the null after adjustment for BMI (**Table S5**).

Longitudinal analyses for adiposity and markers of hepatic function

In post-hoc exploratory analyses there were inverse associations of MDS with changes in BMI [β coefficient (95% CIs) per one SD higher MDS of -0.08 (-0.15, -0.02)] and in waist circumference [-0.33 (-0.61, -0.06)] (**Table S7**). For the markers of hepatic function, MDS showed a trend toward inverse association with GGT levels (p_{trend}=0.047) [β coefficient (95% CIs) per one SD higher MDS of -1.66 (-3.73, 0.41)], but not with ALT or AST levels (**Table S8**).

DISCUSSION

In this first population-based European study among adults free from clinically manifest hepatic steatosis to report on the prospective association between adherence to the Mediterranean diet and risk of hepatic steatosis, we found an inverse association between MDS and risk of hepatic steatosis based on FLI criteria. This relationship was attenuated to the null when controlled for general and central adiposity assessed by the BMI and waist circumference. In contrast, there was no association between adherence to the Mediterranean diet and risk of hepatic steatosis based on NAFLD-score criteria.

Current findings in context of other evidence

Our finding based on FLI is consistent with the only published prospective study relating the Mediterranean diet to hepatic steatosis, where each SD increase in MDS was estimated to decrease the odds for incident hepatic steatosis by 26% (95% CI 10%-39%) [28]. By contrast, the point estimate of the effect size in our study was smaller [(16% (95% CI 4%-27%)]. A possible explanation partly lies in methodological differences. We used biochemical

For peer review only - http://bmjopen.bmj.com/site/about/guidelines.xhtml

and anthropometric markers to estimate hepatic steatosis in the current study, while the previous study used computed tomography assessment.

No association was found between adherence to the Mediterranean diet and risk of hepatic steatosis based on the NAFLD-score. For possible explanations based on the differences in their components, the FLI includes GGT, while NAFLD-score includes AST and AST/ALT ratio; the FLI includes adiposity markers, while the NAFLD-score does not; the FLI includes a lipid marker (triglycerides) while NAFLD-score includes markers of glycaemic status. Previous studies showed a modest association of GGT and ALT (but not AST) with the prevalence of hepatic steatosis [1]. Indeed, our analysis showed an inverse association between MDS and GGT levels, but not with AST or ALT, and these findings could explain the discrepancy between the two indices. Notably, our sensitivity analyses using different definitions of hepatic steatosis showed an inverse association between MDS quintiles and risk of hepatic steatosis based on NAFLD-score after excluding participants with high baseline NAFLD-score only. This could be explained by modest concordance between the two measures. Of note, there were no statistically significant associations between MDS and risk of hepatic steatosis based on FLI after excluding participants with FLI>30 at baseline, which led to a smaller sample size and consequently a lower statistical power in our study. In a post-hoc analysis, we found an inverse association between adherence to the Mediterranean diet and hepatic steatosis as assessed by HSI, an alternative score to detect hepatic steatosis. Although, case identification by HSI and FLI (kappa=0.27) or NAFLD-score (Kappa=0.18) was weakly concordant; our finding based on FLI is consistent with HSI. This could be explained potentially by BMI as one of the components of both FLI and HSI, highlighting the importance of obesity for incident hepatic steatosis [41].

The inverse association between MDS and risk of hepatic steatosis defined by FLI remained significant after adjusting for BMI only but became imprecise and not significant

For peer review only

after adjusting for both BMI and waist circumference or for changes in BMI over the follow-up period. These results suggest the collinearity between the central adiposity and hepatic steatosis as the central adiposity may partly reflect hepatic steatosis. This biologically plausible finding is in agreement with previous cross-sectional findings for MDS and prevalent hepatic steatosis in CoLaus study and the British Fenland Study we reported [27] and a study in Hong Kong [42]. Our finding that the MDS was negatively associated with an increase in BMI after 5.3 years of follow-up is consistent with the findings from the Framingham Heart Study which observed the same trend over 6-year follow-up [28]; and from Nurses' Health Study and Health Professionals' Follow-up Study evaluating 20-year longitudinal data with repeated self-reported measures of diet and adiposity measures [43].

Sequential subtraction of different components of the MDS showed that fruits, cereals, dairy products, red or processed meat, or alcohol partially accounted for the observed association. These findings agree with previous studies [26,28,44,45] including the Framingham Heart Study suggesting benefits of low consumption of red meat and high consumption of fruits or whole grains [28]; and a cross-sectional analysis from the PREDIMED study suggesting the benefit of low consumption of red meat [26]. Moreover, the Mediterranean diet is characterized by a moderate-to-high consumption of whole grains, which has been inversely associated with the likelihood of having NAFLD [46].

Possible mechanisms and implications

Hepatic steatosis is associated with a number of metabolic risk factors including insulin resistance, type 2 diabetes, dyslipidaemia, metabolic syndrome, and oxidative stress [47]. Mediterranean-diet associated phenolic compounds (phenolic acids and polyphenols) found in fruits and vegetables and high levels of monounsaturated fatty acids of olive oil have been shown to inhibit *de novo* lipogenesis, improve peripheral insulin sensitivity, and reduce

For peer review only - http://bmjopen.bmj.com/site/about/guidelines.xhtml

cardiovascular risk mainly due to their antioxidant, anti-inflammatory, and anti-fibrotic effects [18,48–51]. Moreover, different components of the Mediterranean diet, including omega 3 polyunsaturated fatty acids (PUFA), fibre, and antioxidant rich-foods, are inversely associated with hepatic steatosis [52,53]. One meta-analysis of interventional studies reported that omega-3 PUFA were negatively associated with hepatic steatosis [54]. The Mediterranean diet is also low in saturated fat, which has been demonstrated to increase hepatic triglycerides content and hepatic insulin resistance [55,56]. Finally, the high fibre content of the Mediterranean diet has been associated with reduced hepatic fat [18,52].

Hepatic steatosis is associated with cardiometabolic diseases and substantially impacts public health [13,15,16]. Thus, our finding of an inverse association between adherence to the Mediterranean diet and risk of hepatic steatosis would support the importance of dietary advice for the prevention of hepatic steatosis as well as its treatment. However, future work should confirm whether or not the clinical importance of the Mediterranean diet for the prevention of hepatic steatosis is independent of obesity or central adiposity.

Strengths and limitations

To our knowledge, this is the first European prospective study assessing the association between the Mediterranean diet and risk of hepatic steatosis. The study had the benefit of a relatively large sample size and an average of 5.3 years of follow-up, and applied a definition of the Mediterranean diet that has been shown to be valid in a non-Mediterranean population [33].

Several limitations of this study merit consideration. Measurement error and recall bias are inevitable when using self-reported dietary instruments, limiting the ability to precisely measure adherence to the Mediterranean diet, although adjustment for energy intake may have reduced the magnitude of measurement error [57]. We used diet data measured only

For peer review only - http://bmjopen.bmj.com/site/about/guidelines.xhtml

at baseline, and intra-individual variation over time might be present. However, we previously reported that dietary intake is stable in CoLaus study and in Switzerland in general [58,59].

Our ascertainment of hepatic steatosis was based on two indices, but not on liver biopsy or direct imaging assessment; hence, information bias (misdiagnosis) cannot be ruled out. Although liver biopsy is the gold standard for diagnosing hepatic steatosis, its use in apparently healthy participants would be unethical and unfeasible in a large population-based study. Further, previous studies have shown that FLI and NAFLD-score can accurately identify hepatic steatosis with good sensitivity and specificity, and these scores have been validated for use in large epidemiological studies [36,37,60,61]. But, although both FLI and NAFLD-score can indicate hepatic steatosis, their precision is variable [62]. Moreover, presence of steatohepatitis or advanced liver fibrosis could influence the relationship of FLI or NAFLD-score with hepatic steatosis [63]. Of note, our study was not designed to assess the role of adherence to the Mediterranean diet in hepatic fibrosis.

Although we adjusted for many relevant confounders and performed a series of sensitivity analyses, we cannot rule out residual confounding due to unmeasured variables or covariates measured with error. Generalisability is limited because included participants seemed to be healthier than those excluded, and our findings were obtained in a single European population. Still, they confirm the findings from a previous prospective study conducted in the US [28], and might serve as a reference for other studies.

Conclusion

Adherence to the Mediterranean diet was inversely associated with risk of hepatic steatosis based on the FLI, and the association was independent of several known risk factors. Conversely, the association was not observed when using different criteria specifying the NAFLD-score. These findings support recommendations on following the Mediterranean diet

For peer review only - http://bmjopen.bmj.com/site/about/guidelines.xhtml

for hepatic steatosis prevention in addition to the existing evidence for its benefit for cardiovascular disease prevention. Nonetheless, the findings also highlight the need for further research with more accurate measures of hepatic steatosis to replicate these findings in different populations and settings.

For peer review only

For peer review only - http://bmjopen.bmj.com/site/about/guidelines.xhtml

ABBREVIATIONS

NAFLD, non-alcoholic fatty liver disease; CVD; cardiovascular disease; FFQ, food frequency questionnaire; MDS, Mediterranean diet score; FLI, fatty liver index; BMI, body-mass index; TG, triglyceride; GGT, gamma-glutamyl transferase; AST, aspartate-aminotransferase; ALT, alanine transaminase; PUFA, polyunsaturated fatty acids.

DECLARATION

Acknowledgements: The authors are grateful to all the participants and staff of CoLaus study.

Competing interests: None.

Ethics approval and consent to participate: The institutional Ethics Committee of the University of Lausanne, which afterwards became the Ethics Commission of Canton Vaud (www.cer-vd.ch) approved the baseline CoLaus study (reference 16/03); the approval was renewed for the first (reference 33/09) and the second (reference 26/14) follow-ups.

The CoLaus study was performed in agreement with the Helsinki declaration and its former amendments, and all participants provided their written informed consent before entering the study.

Funding: The CoLaus study was and is supported by research grants from GlaxoSmithKline, the Faculty of Biology and Medicine of Lausanne, and the Swiss National Science Foundation (grants 33CSCO-122661, 33CS30-139468 and 33CS30-148401). SKS was supported by the Swiss National Science Foundation (Doc.Mobility number P1LAP3-171805). NGF and FI acknowledge core MRC support (MC_UU_12015/5), and NGF acknowledges NIHR

For peer review only

For peer review only - http://bmjopen.bmj.com/site/about/guidelines.xhtml

Biomedical Research Centre Cambridge: Nutrition, Diet, and Lifestyle Research Theme (IS-BRC-1215-20014).

Authors' contribution: SKS, FI, and NGF designed the study question and had full access to all the data in the study and took responsibility for the integrity and accuracy of the data. SKS performed the statistical analyses and she wrote the first draft with supervision from FI, PMV, and NGF. All authors: contributed to interpretation of data, revised the article critically for important intellectual content, and approved the final version of the manuscript.

Data availability statement: Non-identifiable individual-level data are available for researchers who seek to answer questions related to health and disease in the context of research projects who meet the criteria for data sharing by research committees. Please follow the instructions at https://www.colaus-psycolaus.ch/ for information on how to submit an application for gaining access to CoLaus data.

For peer review only

SUPPLEMENTARY INFORMATION

Figure S1 Sample selection flow chart.

Table S1 Characteristics of the participants included and excluded from the analysis, CoLaus study, Switzerland.

Table S2 Baseline characteristics of the participants according to the risk of hepatic steatosis, defined by the fatty liver index or the NAFLD-score, CoLaus study, Switzerland.

Table S3 Prospective association of the Mediterranean diet score with the risk of hepatic steatosis, CoLaus study, Switzerland: assessment of influence of other potential covariates.

Table S4 Sensitivity analyses for the association of the Mediterranean diet score with the risk of hepatic steatosis, CoLaus study, Switzerland.

Table S5 Prospective association of the Mediterranean diet score with the risk of hepatic steatosis, CoLaus study, Switzerland: sensitivity analysis while excluding participants with only one of the indices high at baseline and using hepatic steatosis index as an alternative score.

Table S6 Prospective association of the Mediterranean diet score with the risk of hepatic steatosis, CoLaus study, Switzerland (n=1632). Sensitivity analysis after excluding participants with either indices high (using FLI>30 instead of FLI≥60).

Table S7 Association of the Mediterranean diet score with change in BMI and waist circumference, CoLaus study, Switzerland.

Table S8 Association of the Mediterranean diet score with GGT, ALT, and AST, CoLaus study, Switzerland.

For peer review only

For peer review only - http://bmjopen.bmj.com/site/about/guidelines.xhtml

REFERENCES

1 Sattar N, Forrest E, Preiss D. Non-alcoholic fatty liver disease. *BMJ* 2014;**349**. doi:10.1136/bmj.g4596

2 Angulo P. Treatment of nonalcoholic fatty liver disease. *AnnHepatol* 2002;**1**:12–9.

3 Williamson RM, Price JF, Glancy S, *et al.* Prevalence of and Risk Factors for Hepatic Steatosis and Nonalcoholic Fatty Liver Disease in People With Type 2 Diabetes: the Edinburgh Type 2 Diabetes Study. *Diabetes Care* 2011;**34**:1139 LP – 1144.http://care.diabetesjournals.org/content/34/5/1139.abstract

4 Bellentani S, Scaglioni F, Marino M, *et al.* Epidemiology of non-alcoholic fatty liver disease. *Dig Dis* 2010;**28**:155–61. doi:10.1159/000282080

5 Angulo P, Keach JC, Batts KP, *et al.* Independent predictors of liver fibrosis in patients with nonalcoholic steatohepatitis. *Hepatology* 1999;**30**:1356–62. doi:10.1002/hep.510300604

6 Marchesini G, Petta S, Grave RD. Diet, Weight Loss, and Liver Health in Nonalcoholic Fatty Liver Disease: Pathophysiology, Evidence, and Practice. *Hepatology* 2016;**63plos**:2032–43. doi:10.1002/hep.28392

7 Marchesini G, Day CP, Dufour JF, *et al.* EASL-EASD-EASO Clinical Practice Guidelines for the management of non-alcoholic fatty liver disease. *J Hepatol* 2016;**64**:1388–402.http://ovidsp.ovid.com/athens/ovidweb.cgi?T=JS&CSC=Y&NEWS=N&PAGE=fulltext&D=emed18&AN=609669095

8 Chalasani N, Younossi Z, Lavine JE, *et al.* The diagnosis and management of non-alcoholic fatty liver disease: practice Guideline by the American Association for the Study of Liver Diseases, American College of Gastroenterology, and the American Gastroenterological Association. *Hepatology* 2012;**55**:2005–23. doi:10.1002/hep.25762

9 Sawangjit R, Chongmelaxme B, Phisalprapa P, *et al.* Comparative efficacy of interventions on nonalcoholic fatty liver disease (NAFLD): A PRISMA-compliant systematic review and network meta-analysis. *Med* 2016;**95**:e4529. doi:10.1097/md.0000000000004529

10 Plauth M, Bernal W, Dasarathy S, *et al.* ESPEN guideline on clinical nutrition in liver disease. *Clin Nutr* 2019;**38**:485–521. doi:https://doi.org/10.1016/j.clnu.2018.12.022

For peer review only - http://bmjopen.bmj.com/site/about/guidelines.xhtml

11 Abenavoli L, Di Renzo L, Boccuto L, *et al.* Health benefits of Mediterranean diet in nonalcoholic fatty liver disease. *Expert Rev Gastroenterol Hepatol* 2018;**12**:873–81. doi:10.1080/17474124.2018.1503947

12 Asbaghi O, Choghakhori R, Ashtary-Larky D, *et al.* Effects of the Mediterranean diet on cardiovascular risk factors in non-alcoholic fatty liver disease patients: A systematic review and meta-analysis. *Clin Nutr ESPEN* 2020;**37**:148–56. doi:10.1016/j.clnesp.2020.03.003

13 Estruch R, Ros E, Salas-Salvadó J, *et al.* Primary Prevention of Cardiovascular Disease with a Mediterranean Diet Supplemented with Extra-Virgin Olive Oil or Nuts. *N Engl J Med* 2018;**378**:e34. doi:10.1056/NEJMoa1800389

14 Sofi F, Cesari F, Abbate R, *et al.* Adherence to Mediterranean diet and health status: meta-analysis. *BMJ* 2008;**337**:a1344. doi:10.1136/bmj.a1344

15 Salas-Salvado J, Bullo M, Babio N, *et al.* Reduction in the incidence of type 2 diabetes with the Mediterranean diet: results of the PREDIMED-Reus nutrition intervention randomized trial. *Diabetes Care* 2011;**34**:14–9. doi:10.2337/dc10-1288

16 Esposito K, Kastorini C-M, Panagiotakos DB, *et al.* Mediterranean diet and metabolic syndrome: an updated systematic review. *Rev Endocr Metab Disord* 2013;**14**:255–63. doi:10.1007/s11154-013-9253-9

17 Ryan MC, Itsiopoulos C, Thodis T, *et al.* The Mediterranean diet improves hepatic steatosis and insulin sensitivity in individuals with non-alcoholic fatty liver disease. *J Hepatol* 2013;**59**:138–43. doi:10.1016/j.jhep.2013.02.012

18 Properzi C, O'Sullivan TA, Sherriff JL, *et al.* Ad libitum Mediterranean and Low Fat Diets both Significantly Reduce Hepatic Steatosis: a Randomized Controlled Trial. *Hepatology* Published Online First: May 2018. doi:10.1002/hep.30076

19 Misciagna G, del Pilar Diaz M, Caramia D V, *et al.* Effect of a low glycemic index Mediterranean diet on non-alcoholic fatty liver disease. A randomized controlled clinici trial. *J Nutr Heal AGING* 2017;**21**:404–12. doi:10.1007/s12603-016-0809-8

20 Trovato FM, Catalano D, Martines GF, *et al.* Mediterranean diet and non-alcoholic fatty liver disease: the need of extended and comprehensive interventions. *Clin Nutr* 2015;**34**:86–8.

For peer review only - http://bmjopen.bmj.com/site/about/guidelines.xhtml

doi:10.1016/j.clnu.2014.01.018

21 Gelli C, Tarocchi M, Abenavoli L, *et al.* Effect of a counseling-supported treatment with the Mediterranean diet and physical activity on the severity of the non-alcoholic fatty liver disease. *World J Gastroenterol* 2017;**23**:3150–62. doi:10.3748/wjg.v23.i17.3150

22 Baratta F, Pastori D, Polimeni L, *et al.* Adherence to mediterranean diet and non-alcoholic fatty liver disease: Impact on metabolic profile. *Nutr Metab Cardiovasc Dis* 2017;**27 (1)**:e8.http://ovidsp.ovid.com/athens/ovidweb.cgi?T=JS&CSC=Y&NEWS=N&PAGE=fulltext &D=emex&AN=614265212

23 Abenavoli L, Greco M, Nazionale I, *et al.* Effects of Mediterranean diet supplemented with silybin-vitamin E-phospholipid complex in overweight patients with non-alcoholic fatty liver disease. *Expert Rev Gastroenterol Hepatol* 2015;**9**:519–27. doi:10.1586/17474124.2015.1004312

24 Fraser A, Abel R, Lawlor DA, *et al.* A modified Mediterranean diet is associated with the greatest reduction in alanine aminotransferase levels in obese type 2 diabetes patients: results of a quasi-randomised controlled trial. *Diabetologia* 2008;**51**:1616–22. doi:10.1007/s00125-008-1049-1

25 Kontogianni MD, Tileli N, Margariti A, *et al.* Adherence to the Mediterranean diet is associated with the severity of non-alcoholic fatty liver disease. *Clin Nutr* 2014;**33**:678–83. doi:10.1016/j.clnu.2013.08.014

26 Cantero I, Abete I, Babio N, *et al.* Dietary Inflammatory Index and liver status in subjects with different adiposity levels within the PREDIMED trial. *Clin Nutr* Published Online First: 2017. doi:http://dx.doi.org/10.1016/j.clnu.2017.06.027

27 Khalatbari-Soltani S, Imamura F, Brage S, *et al.* The association between adherence to the Mediterranean diet and hepatic steatosis: cross-sectional analysis of two independent studies, the UK Fenland Study and the Swiss CoLaus Study. *BMC Med* 2019;**17**:19. doi:10.1186/s12916-019-1251-7

28 Ma J, Hennein R, Liu C, *et al.* Improved Diet Quality Associates With Reduction in Liver Fat, Particularly in Individuals With High Genetic Risk Scores for Nonalcoholic Fatty Liver

For peer review only

For peer review only - http://bmjopen.bmj.com/site/about/guidelines.xhtml

Disease. *Gastroenterology* 2018;**155**:107–17. doi:10.1053/j.gastro.2018.03.038

29 Firmann M, Mayor V, Vidal PM, *et al.* The CoLaus study: a population-based study to investigate the epidemiology and genetic determinants of cardiovascular risk factors and metabolic syndrome. *BMC Cardiovasc Disord* 2008;**8**:6. doi:10.1186/1471-2261-8-6

30 Morabia A, Bernstein M, Kumanyika S, *et al.* Développement et validation d'un questionnaire alimentaire semi-quantitatif à partir d'une enquête de population. *Soz Praventivmed* 1994;**39**:345–69. doi:10.1007/BF01299666

31 Bernstein L, Huot I MA. Amélioration des performances d'un questionnaire alimentaire semi-quantitatif comparé à un rappel des 24 heures. *Sante Publique (Paris)* 1995;**7**:403–13.

32 Bach-Faig A, Berry EM, Lairon D, *et al.* Mediterranean diet pyramid today. Science and cultural updates. *Public Health Nutr* 2011;**14**:2274–84. doi:10.1017/S1368980011002515

33 Tong TYN, Wareham NJ, Khaw K-T, *et al.* Prospective association of the Mediterranean diet with cardiovascular disease incidence and mortality and its population impact in a non-Mediterranean population: the EPIC-Norfolk study. *BMC Med* 2016;**14**:135. doi:10.1186/s12916-016-0677-4

34 Willett WC, Howe GR, Kushi LH. Adjustment for total energy intake in epidemiologic studies. *Am J Clin Nutr* 1997;**65**:1220S-1228S; discussion 1229S-1231S.

35 Bedogni G, Bellentani S, Miglioli L, *et al.* The Fatty Liver Index: a simple and accurate predictor of hepatic steatosis in the general population. *BMC Gastroenterol* 2006;**6**:33. doi:10.1186/1471-230X-6-33

36 Kotronen A, Peltonen M, Hakkarainen A, *et al.* Prediction of Non-Alcoholic Fatty Liver Disease and Liver Fat Using Metabolic and Genetic Factors. *Gastroenterology* 2009;**137**:865–72. doi:10.1053/j.gastro.2009.06.005

37 Ruhl CE, Everhart JE. Fatty liver indices in the multiethnic United States National Health and Nutrition Examination Survey. *Aliment Pharmacol Ther* 2015;**41**:65–76. doi:10.1111/apt.13012

38 Alberti KGMM, Zimmet P, Shaw J. Metabolic syndrome--a new world-wide definition. A Consensus Statement from the International Diabetes Federation. *Diabet Med* 2006;**23**:469–80.

For peer review only

doi:10.1111/j.1464-5491.2006.01858.x

39 Bernstein M, Sloutskis D, Kumanyika S, *et al.* Data-based approach for developing a physical activity frequency questionnaire. *Am J Epidemiol* 1998;**147**:147–54.

40 Lee J-H, Kim D, Kim HJ, *et al.* Hepatic steatosis index: a simple screening tool reflecting nonalcoholic fatty liver disease. *Dig liver Dis Off J Ital Soc Gastroenterol Ital Assoc Study Liver* 2010;**42**:503–8. doi:10.1016/j.dld.2009.08.002

41 Stefan N, Häring H-U, Cusi K. Non-alcoholic fatty liver disease: causes, diagnosis, cardiometabolic consequences, and treatment strategies. *lancet Diabetes Endocrinol* 2019;**7**:313–24. doi:10.1016/S2213-8587(18)30154-2

42 Chan R, Wong VW, Chu WC, *et al.* Diet-Quality Scores and Prevalence of Nonalcoholic Fatty Liver Disease: A Population Study Using Proton-Magnetic Resonance Spectroscopy. *PLoS One* 2015;**10**:e0139310. doi:10.1371/journal.pone.0139310

43 Fung TT, Pan A, Hou T, *et al.* Long-Term Change in Diet Quality Is Associated with Body Weight Change in Men and Women. *J Nutr* 2015;**145**:1850–6. doi:10.3945/jn.114.208785

44 Freedman ND, Cross AJ, McGlynn KA, *et al.* Association of meat and fat intake with liver disease and hepatocellular carcinoma in the NIH-AARP cohort. *J Natl Cancer Inst* 2010;**102**:1354–65. doi:10.1093/jnci/djq301

45 Georgoulis M, Kontogianni MD, Yiannakouris N. Mediterranean Diet and Diabetes: Prevention and Treatment. *Nutrients* 2014;**6**:1406–23. doi:10.3390/nu6041406

46 Georgoulis M, Kontogianni MD, Tileli N, *et al.* The impact of cereal grain consumption on the development and severity of non-alcoholic fatty liver disease. *Eur J Nutr* 2014;**53**:1727–35. doi:10.1007/s00394-014-0679-y

47 Abenavoli L, Milic N, Di Renzo L, *et al.* Metabolic aspects of adult patients with nonalcoholic fatty liver disease. *World J Gastroenterol* 2016;**22**:7006–16. doi:10.3748/wjg.v22.i31.7006

48 Salomone F, Godos J, Zelber-Sagi S. Natural antioxidants for non-alcoholic fatty liver disease: molecular targets and clinical perspectives. *LIVER Int* 2016;**36**:5–20. doi:10.1111/liv.12975

49 Abenavoli L, Milic N, Luzza F, *et al.* Polyphenols Treatment in Patients with Nonalcoholic Fatty Liver Disease. *J Transl Intern Med* 2017;**5**:144–7. doi:10.1515/jtim-2017-0027

For peer review only - http://bmjopen.bmj.com/site/about/guidelines.xhtml

50 Yang J, Fernández-Galilea M, Martínez-Fernández L, *et al.* Oxidative Stress and Non-Alcoholic Fatty Liver Disease: Effects of Omega-3 Fatty Acid Supplementation. *Nutrients* 2019;**11**. doi:10.3390/nu11040872

51 Salomone F, Ivancovsky-Wajcman D, Fliss-Isakov N, *et al.* Higher phenolic acid intake independently associates with lower prevalence of insulin resistance and non-alcoholic fatty liver disease. *JHEP Reports* 2020;**2**:100069. doi:https://doi.org/10.1016/j.jhepr.2020.100069

52 Zelber-Sagi S, Salomone F, Mlynarsky L. The Mediterranean dietary pattern as the diet of choice for non-alcoholic fatty liver disease: Evidence and plausible mechanisms. *Liver Int* 2017;**37**:936–49.

53 Mahady SE, George J. Exercise and diet in the management of nonalcoholic fatty liver disease. *Metab Exp* 2016;**65**:1172–82. doi:10.1016/j.metabol.2015.10.032

54 Parker HM, Johnson NA, Burdon CA, *et al.* Omega-3 supplementation and non-alcoholic fatty liver disease: A systematic review and meta-analysis. *J Hepatol* 2012;**56**:944–51. doi:10.1016/j.jhep.2011.08.018

55 Hernandez EA, Kahl S, Seelig A, *et al.* Acute dietary fat intake initiates alterations in energy metabolism and insulin resistance. *J Clin Invest* 2017;**127**:695–708.http://ovidsp.ovid.com/athens/ovidweb.cgi?T=JS&CSC=Y&NEWS=N&PAGE=fulltext&D=emex&AN=614623041

56 Bjermo H, Iggman D, Kullberg J, *et al.* Effects of n-6 PUFAs compared with SFAs on liver fat, lipoproteins, and inflammation in abdominal obesity: a randomized controlled trial. *Am J Clin Nutr* 2012;**95**:1003–12. doi:10.3945/ajcn.111.030114

57 Hu FB, Stampfer MJ, Rimm E, *et al.* Dietary fat anc coronary heart disease: a comparison of approaches for adjusting for total energy intake and modeling repeated dietary measurements. *Am J Epidemiol* 1999;**149**:531–40.

58 Marques-Vidal P, Quinteiros Fidalgo AS, Schneid Schuh D, *et al.* Lessons learned? Changes in dietary behavior after a coronary event. *Clin Nutr ESPEN* 2019;**29**:112–8. doi:https://doi.org/10.1016/j.clnesp.2018.11.010

59 Schneid Schuh D, Guessous I, Gaspoz J-M, *et al.* Twenty-four-year trends and determinants of

For peer review only - http://bmjopen.bmj.com/site/about/guidelines.xhtml

change in compliance with Swiss dietary guidelines. *Eur J Clin Nutr* 2019;**73**:859–68. doi:10.1038/s41430-018-0273-0

60 Koehler EM, Schouten JNL, Hansen BE, *et al.* External Validation of the Fatty Liver Index for Identifying Nonalcoholic Fatty Liver Disease in a Population-based Study. *Clin Gastroenterol Hepatol* 2013;**11**:1201–4.http://ovidsp.ovid.com/athens/ovidweb.cgi?T=JS&CSC=Y&NEWS=N&PAGE=fulltext&D=emed15&AN=52528553

61 Machado M V, Cortez-Pinto H. Non-invasive diagnosis of non-alcoholic fatty liver disease. A critical appraisal. *J Hepatol* 2013;**58**:1007–19. doi:10.1016/j.jhep.2012.11.021

62 Zelber-Sagi S, Webb M, Assy N, *et al.* Comparison of fatty liver index with noninvasive methods for steatosis detection and quantification. *World J Gastroenterol* 2013;**19**:57–64. doi:10.3748/wjg.v19.i1.57

63 Fedchuk L, Nascimbeni F, Pais R, *et al.* Performance and limitations of steatosis biomarkers in patients with nonalcoholic fatty liver disease. *Aliment Pharmacol Ther* 2014;**40**:1209–22. doi:10.1111/apt.12963

For peer review only

Table 1 Baseline characteristics of participants according to quintiles of the Mediterranean diet score, CoLaus study, Switzerland (n=2,288).

Characteristic	Quintiles of Mediterranean diet score* Q1 (n=458)	Q2 (n=458)	Q3 (n=457)	Q4 (n=458)	Q5 (n=457)	*P*-value*
Age (years)	57.1±10.6	56.7±9.9	56.4±10.5	55±9.8	53.7±8.9	<0.001
Women (%)	59.0	62.9	67.6	66.6	71.1	0.001
Marital status (%)						0.51
Single	17.7	14.2	16.8	17.5	16.4	
Married/cohabitant	53.9	55.7	59.1	57.2	57.3	
Widowed/separated/divorced	28.4	30.1	24.1	25.3	26.3	
Employed (%)	57.9	60.3	59.1	68.3	72.0	<0.001
Education (%)						
University	19.2	21.6	26.0	31.7	31.1	
High school	26.4	28.2	28.4	29.7	29.5	
Apprenticeship	39.3	35.2	34.8	28.2	28.4	
Mandatory education	15.1	15.1	10.7	10.5	10.9	<0.001
Current smokers (%)	24.7	21.8	22.3	16.2	15.5	0.014
Alcohol intake (%)†						
Abstainers	21.8	24.0	25.8	24.7	21.2	
Moderate	63.8	66.2	69.4	70.7	76.6	
Heavy	14.4	9.8	4.8	4.6	2.2	<0.001
Total energy intake (kcal/day)	1819±705	1812±675	1781±729	1821±653	1801±595	0.87
Protein (%energy)	15.8±3.3	15.9±4.1	15.3±3.0	15.2±3.0	14.7±2.6	<0.001
Carbohydrates (%energy)	45.3±9.2	45.9±9.1	47.3±8.0	47.6±8.2	49.0±7.8	<0.001
Fat (%energy)	33.6±6.5	34.5±6.9	34.6±6.7	34.5±6.5	34.1±6.9	0.11
TEE (kcal/day)	2562±589	2600±618	2564±602	2558±555	2589±565	0.84
Metabolic syndrome (%)‡	11.8	9.8	12.3	12.7	7.4	0.063

For peer review only - http://bmjopen.bmj.com/site/about/guidelines.xhtml

BMI (kg/m²)	24.0±2.7	23.8±2.9	23.9±3.0	23.6±2.8	23.3±2.7	0.003
Waist circumference (cm)	85.4±8.5	84.9±9.4	85.0±9.2	84.0±8.7	82.9±8.6	<0.001
Triglycerides (mmol/l)	1.1±0.5	1.0±0.5	1.1±0.5	1.0±0.5	1.0±0.5	
median (iqr)	1.0 (0.8, 1.3)	0.9 (0.7, 1.2)	1.0 (0.7, 1.3)	0.9 (0.7, 1.2)	0.9 (0.7, 1.2)	0.009
GGT (U/l)	25.0±16.5	25.1±17.9	23.2±14.5	24.0±17.9	21.5±12.1	0.002
median (iqr)	20 (15, 29)	21 (15, 28)	19 (14, 28)	19 (14, 26)	18 (14, 25)	
≥50 (%)	7.4	6.3	5.7	6.1	2.6	0.025
ALT (U/l)	21.3±7.8	22.1±8.9	22.5±9.7	21.3±8.7	21.5±8.7	0.12
median (iqr)	19 (16, 25)	20 (16, 26)	20 (16, 27)	19 (16, 25)	20 (16, 24)	
≥40 (%)	3.5	5.4	8.3	4.6	3.5	0.005
AST (U/l)	26.0±5.85	26.2±6.5	26.1±6.6	25.8±6.1	25.7±5.7	0.68
median (iqr)	25 (22, 29)	25 (22, 29)	25 (21, 29)	25 (22, 29)	25 (22, 29)	
≥37 (%)	4.1	8.3	6.3	5.0	4.4	0.038

Abbreviations: TEE, total energy expenditure; BMI, body mass index; iqr, interquartile range; GGT, gamma-glutamyl transferase; ALT, alanine aminotransferase; AST, aspartate aminotransferase.

* The population was divided into five groups by quintiles (Q1-Q5) of the Mediterranean diet score. P-values were computed by using ANOVA for continuous variables and Chi-square test for categorical variables.

† Alcohol consumption categorized as "abstainers" (0 unit/week), "moderate" (1–21 units/week for men, 1–14 for women), and "heavy drinkers" (>21 units/week for men, >14 for women).

‡ Metabolic syndrome defined according to the International Diabetic Federation (waist circumference ≥94 cm in men and ≥80 cm in women plus at least two of the following factors: serum triglycerides≥1.70 mmol/L or specific treatment for this lipid abnormality; serum high-density lipoprotein cholesterol <1.03 mmol/L in men and <1.29 mmol/L in women or specific treatment for this lipid abnormality; systolic blood pressure ≥130 mm Hg or diastolic blood pressure ≥85 mm Hg or treatment for previously diagnosed hypertension; and fasting plasma glucose ≥5.6 mmol/L or previously diagnosed type 2 diabetes).

For peer review only - http://bmjopen.bmj.com/site/about/guidelines.xhtml

Table 2 Prospective association of the Mediterranean diet score with the risk of hepatic steatosis, CoLaus study, Switzerland (n=2,288).

	Risk ratio (95% CI) across quintiles of Mediterranean diet score*					*P*-trend	Risk ratio (95% CI) Per SD increase*
	Q1	Q2	Q3	Q4	Q5		
range	1.83-7.63	7.64-8.35	8.36-8.92	8.93-9.59	9.60-12.1		
N total	458	458	457	458	457		
Fatty liver index, median (iqr)†	21.8 (10.4, 37.7)	20.4 (9.0, 39.4)	18.5 (8.9, 34.6)	18.1 (7.7, 33.1)	14.2 (6.6, 28.5)		
N cases (score≥60)	36	43	35	22	17		
Unadjusted	1.00 (ref.)	1.19 (0.78, 1.82)	0.97 (0.62, 1.52)	0.61 (0.37, 1.02)	0.47 (0.27, 0.83)	0.001	0.79 (0.70, 0.90)
Multivariable ‡	1.00 (ref.)	1.11 (0.72, 1.72)	1.07 (0.68, 1.66)	0.71 (0.42, 1.22)	0.50 (0.28, 0.91)	0.006	0.84 (0.73, 0.96)
Multivariable + BMI	1.00 (ref.)	1.12 (0.73, 1.71)	0.90 (0.58, 1.39)	0.74 (0.44, 1.24)	0.61 (0.34, 1.09)	0.031	0.85 (0.73, 0.99)
Multivariable + BMI + WC	1.00 (ref.)	1.07 (0.70, 1.64)	0.90 (0.59, 1.37)	0.74 (0.44, 1.26)	0.60 (0.34, 1.08)	0.034	0.85 (0.71, 1.02)
NAFLD-score, median (iqr)§	-2.1 (-2.4, -1.5)	-2.0 (-2.4, -1.4)	-2.0 (-2.4, -1.3)	-2.1 (-2.5, -1.5)	-2.1 (-2.5, -1.6)		
N cases (score≥-0.640)	41	46	51	38	32		
Unadjusted	1.00 (ref.)	1.12 (0.75, 1.67)	1.25 (0.84, 1.84)	0.93 (0.61, 1.41)	0.78 (0.50, 1.22)	0.17	0.93 (0.82, 1.05)
Multivariable ‡	1.00 (ref.)	1.13 (0.75, 1.70)	1.26 (0.85, 1.87)	1.01 (0.65, 1.56)	0.80 (0.50, 1.28)	0.28	0.95 (0.83, 1.09)
Multivariable + BMI	1.00 (ref.)	1.17 (0.78, 1.75)	1.17 (0.80, 1.71)	1.07 (0.69, 1.65)	0.95 (0.60, 1.52)	0.71	0.99 (0.86, 1.15)
Multivariable + BMI + WC	1.00 (ref.)	1.12 (0.75, 1.67)	1.17 (0.80, 1.72)	1.07 (0.70, 1.65)	0.96 (0.60, 1.53)	0.80	1.00 (0.86, 1.17)

Abbreviations: iqr, interquartile range; BMI, body mass index; WC, waist circumference; NAFLD, non-alcoholic fatty liver disease.

For peer review only - http://bmjopen.bmj.com/site/about/guidelines.xhtml

Statistical analysis using Poisson regression with robust standard errors; results are expressed as risk ratios and (95% confidence intervals).

* In categorical analysis, the population was divided into five groups by quintiles (Q1-Q5) of the Mediterranean diet score; Standard deviation was 1.20 for the multivariable analyses.

† Calculated based on an algorithm including BMI, waist circumference, triglycerides, and gamma-glutamyl transferase.

‡ Adjusted for age (years), sex, marital status (single, married/cohabiting, and widowed/Separated/divorced), occupational status (working and not working), education level (university, high school, apprenticeship, and mandatory education), smoking status (never, former, and current), energy intake (kcal/day), total energy expenditure (kcal/day), and date of dietary assessment.

§ Calculated based on an algorithm including presence of the metabolic syndrome and type 2 diabetes, and concentrations of fasting serum insulin, fasting serum aspartate-aminotransferase (AST), and the AST/alanine-aminotransferase ratio.

For peer review only

For peer review only - http://bmjopen.bmj.com/site/about/guidelines.xhtml

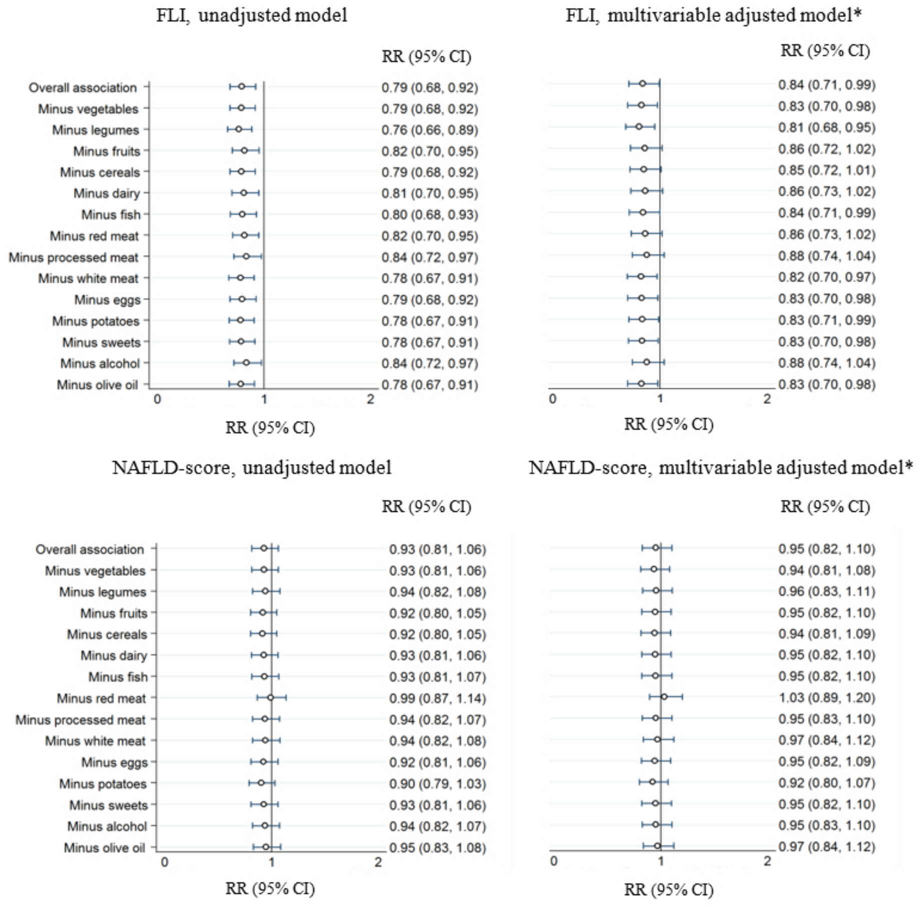

Figure 1 Prospective association of the Mediterranean diet score with the risk of hepatic steatosis, CoLaus study, Switzerland (n=2288): sensitivity analysis to examine influence of each component of Mediterranean diet.

Abbreviations: FLI, fatty liver index; NAFLD, non-alcoholic fatty liver disease.
Statistical analysis using Poisson regression with robust standard errors; results are expressed as risk ratios and (95% confidence intervals).
Risk ratio (RR) and 95% confidence intervals (CI) were estimated per one standard deviation of MDS (overall association) or of each MDS computed after excluding one component.
* Adjusted for age (years), sex, marital status (single, married/cohabiting, and widowed/Separated/divorced), occupational status (working and not working), education level (university, high school, apprenticeship, and mandatory education), smoking status (never, former, and current), energy intake (kcal/day), total energy expenditure (kcal/day), and date of dietary assessment.

Supplementary file

Title: The association between adherence to the Mediterranean diet and hepatic steatosis: the Swiss CoLaus prospective study

For peer review only

Table of Content

Figure S1 Sample selection flow chart. 3

Table S1 Characteristics of the participants included and excluded from the analysis, CoLaus study, Switzerland. 4

Table S2 Baseline characteristics of the participants according to the risk of hepatic steatosis defined by the fatty liver index or the NAFLD-score, CoLaus study, Switzerland. 5

Table S3 Prospective association of the Mediterranean diet score with the risk of hepatic steatosis, CoLaus study, Switzerland: assessment of influence of other potential covariates. 6

Table S4 Sensitivity analyses for the associations of the Mediterranean diet score with the risk of hepatic steatosis, CoLaus study, Switzerland. 8

Table S5 Prospective association of the Mediterranean diet score with the risk of hepatic steatosis, CoLaus study, Switzerland: sensitivity analysis while excluding participants with only one of the indices high at baseline and using hepatic steatosis index as an alternative score. 11

Table S6 Prospective association of the Mediterranean diet score with the risk of hepatic steatosis, CoLaus study, Switzerland (n=1632), Sensitivity analysis while excluding participants with either indices high (using FLI>30 instead of FLI≥60). 13

Table S7 Association of the Mediterranean diet score with change in BMI and waist circumference, CoLaus study, Switzerland. 14

Table S8 Association of the Mediterranean diet score with the GGT, ALT, and AST, CoLaus study, Switzerland. 15

For peer review only

Figure S1 Sample selection flow chart.

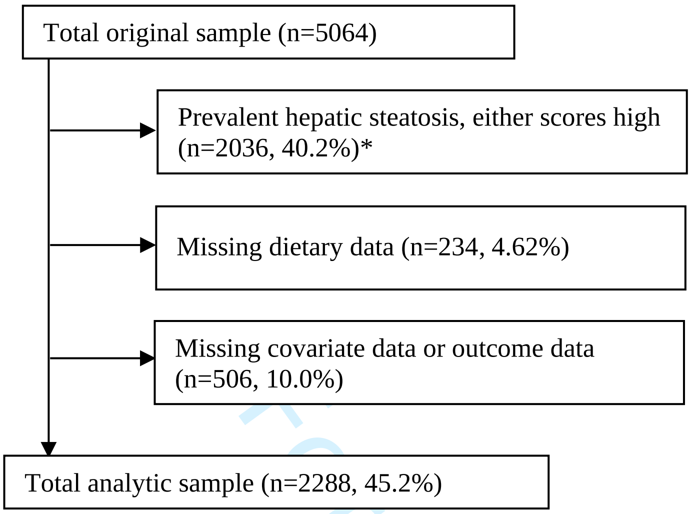

* Prevalent hepatic steatosis was defined as having either fatty liver index≥ 60 OR non-alcoholic fatty liver disease fatty liver score≥ -0.640.

For peer review only

Table S1 Characteristics of the participants included and excluded from the analysis, CoLaus study, Switzerland.

Characteristic	Included (n=2288)	Excluded (n=2776)	*P*-value*
Age, *years*	55.8±10.0	59.4±10.6	<0.001
Women (%)	65.4	43.6	<0.001
Marital status (%)†			0.038
Single	16.5	14.0	
Married/cohabiting	56.6	57.4	
Widowed/separated/divorced	26.8	28.6	
Employed (%)	63.5	50.4	<0.001
Education (%)†			<0.001
University	25.9	17.5	
High school	28.5	23.6	
Apprenticeship	33.2	37.4	
Mandatory education	12.5	21.4	
Current smoker (%)	20.1	23.1	<0.001
Alcohol consumption (%)†‡			<0.001
Abstainers	23.5	26.8	
Moderate	69.3	63.3	
Heavy	7.2	9.9	
Total energy intake (kcal/d)	1807±673	1853±784	0.033
Total protein (% energy)	15.4±3.3	15.6±3.5	0.015
Total carbohydrate (% energy)	47.0±8.6	45.4±9.1	<0.001
Total fat (% energy)	34.3±6.7	34.4±6.9	0.54
TEE (kcal/d)	2575±586	2790±669	<0.001
Metabolic syndrome (%)§	10.8	60.9	<0.001
BMI (kg/m^2)	23.7±2.8	28.3±4.8	<0.001
Waist circumference (cm)	84.4±8.9	98.3±12.5	<0.001
Triglycerides (mmol/l)	1.0±0.5	1.6±1.1	<0.001
median (iqr)	0.9 (0.7, 1.2)	1.4 (1.0, 2.0)	
GGT (U/l)	23.8±16.0	48.8±60.8	<0.001
median (iqr)	19 (14, 27)	32 (21, 53)	
≥50 (%)	5.6	27.5	<0.001
ALT (U/l)	21.8±8.8	32.5±20.7	<0.001
median (iqr)	20 (16, 25)	27 (20, 38)	
≥40 (%)	5.0	23.3	<0.001
AST (U/l)	25.9±6.2	31.6±14.1	<0.001
median (iqr)	25 (22, 29)	28 (24, 34)	
≥37 (%)	5.6	20.4	<0.001

Abbreviations: TEE, total energy expenditure; BMI, body mass index; iqr, interquartile range; GGT, gamma-glutamyl transferase; ALT, alanine aminotransferase; AST, aspartate aminotransferase.
Data are mean ± SD for continuous variables or percent for categorical variables, unless otherwise stated.
* P-value calculated using Chi-square test for categorical variables and student's t-test for continuous variables.
† Due to some missing data, percentages do not always add to 100%.
‡ Alcohol consumption categorized as "abstainers" (0 unit/week), "moderate" (1–21 units/week for men, 1–14 for women), and "heavy drinkers" (>21 units/week for men, >14 for women).
§ Metabolic syndrome defined according to the International Diabetic Federation (waist circumference ≥94 cm in men and ≥80 cm in women plus at least two of the following factors: serum triglycerides≥1.70 mmol/L or specific treatment for this lipid abnormality; serum high-density lipoprotein cholesterol <1.03 mmol/L in men and <1.29 mmol/L in women or specific treatment for this lipid abnormality; systolic blood pressure ≥130 mmHg or diastolic blood pressure ≥85 mmHg or treatment for previously diagnosed hypertension; and fasting plasma glucose ≥5.6 mmol/L or previously diagnosed type 2 diabetes).

For peer review only - http://bmjopen.bmj.com/site/about/guidelines.xhtml

Table S2 Baseline characteristics of the participants according to the risk of hepatic steatosis defined by the fatty liver index or the NAFLD-score, CoLaus study, Switzerland.

	Risk of hepatic steatosis					
	FLI			NAFLD-score		
Characteristic	No (n=2135)	Yes (n=153)	*P*-value*	No (n=2080)	Yes (n=208)	*P*-value*
Age, *years*	55.8±10.1	54.9±9.5	0.29	55.6±10	57.7±10.1	0.003
Women (%)	66.8	45.8	<0.001	66.7	52.9	<0.001
Marital status (%)†			0.86			0.79
Single	16.6	15.0		16.7	14.9	
Married/cohabiting	56.5	58.2		56.5	58.2	
Widowed/Separated/divorced	26.8	26.8		26.8	26.9	
Employed (%)	63.3	66.0	0.50	64.5	53.8	0.002
Education (%)†			0.013			0.33
University	26.7	15.0		26.4	20.7	
High school	28.3	30.1		28.3	29.8	
Apprenticeship	32.6	40.5		32.8	36.5	
Mandatory education	12.3	14.4		12.4	13.0	
Current smoker (%)	19.7	25.5	0.006	20.3	17.8	0.52
Alcohol consumption (%)†‡			0.15			0.001
Abstainers	23.3	26.1		22.5	33.7	
Moderate	69.7	63.4		70.4	58.7	
Heavy	6.9	10.5		7.1	7.7	
Total energy intake (kcal/d)	1800±655	1906±879	0.062	1794±654	1938±833	0.003
Protein (% energy)	15.3±3.1	16.1±5.0	0.006	15.3±3.2	16.0±4.1	0.006
Carbohydrate (% energy)	47.1±8.5	45.6±10.1	0.037	47.0±8.5	46.5±9.5	0.36
Fat (% energy)	34.3±6.7	33.8±7.2	0.37	34.2±6.7	34.4±6.6	0.69
TEE (kcal/d)	2552±572	2912±677	<0.001	2562±582	2699±616	0.002
Metabolic syndrome (%)§	9.9	22.9	<0.001	9.8	21.2	<0.001
BMI (kg/m^2)	23.5±2.7	26.8±2.6	<0.001	23.5±2.8	26.0±2.5	<0.001
Waist circumference (cm)	83.8±8.7	93.4±7.0	<0.001	83.7±8.7	92.0±7.4	<0.001
Triglycerides (mmol/l)	1.0±0.5	1.2±0.5	<0.001	1.0±0.5	1.2±0.5	<0.001
median (iqr)	0.9 (0.7, 1.2)	1.1 (0.7, 1.2)		0.9 (0.7, 1.2)	1.1 (0.8, 1.4)	
GGT (U/l)	23.1±15.2	32.4±22.2	<0.001	23.3±15.4	28.1±20.6	<0.001
median (iqr)	19 (14, 26)	26 (18, 40)		19 (14, 26)	22 (15, 34)	
≥50 (%)	4.9	15.0	<0.001	5.3	9.1	0.022
ALT (U/l)	21.6±8.8	23.5±8.9	0.013	21.3±8.4	26.0±11.6	<0.001
median (iqr)	19 (16, 25)	21 (17, 28)		19 (16, 25)	23 (18, 30)	
≥40 (%)	5.0	5.9	0.63	4.5	10.6	<0.001
AST (U/l)	25.9±6.2	26.3±6.3	0.50	25.9±6.2	26.9±6.2	0.026
median (iqr)	25 (22, 29)	25 (22, 29)		25 (22, 29)	26 (22, 30)	
≥37 (%)	5.5	7.2	0.39	5.3	8.2	0.096

Abbreviations: TEE, total energy expenditure; FLI, fatty liver index; NAFLD, non-alcoholic fatty liver disease; BMI, body mass index; iqr, interquartile range; GGT, gamma-glutamyl transferase; ALT, alanine aminotransferase; AST, aspartate Aminotransferase.

Data are mean ± SD for continuous variables or percent for categorical variables, unless otherwise stated.

* P-value calculated using Chi-square test for categorical variables and student's t-test for continuous variables.

† Due to some missing data, percentages do not always add to 100%.

‡ Alcohol consumption categorized as "abstainers" (0 unit/week), "moderate" (1–21 units/week for men, 1–14 for women), and "heavy drinkers" (>21 units/week for men, >14 for women).

§ Metabolic syndrome defined according to the International Diabetic Federation (waist circumference ≥94 cm in men and ≥80 cm in women plus at least two of the following factors: serum triglycerides≥1.70 mmol/L or specific treatment for this lipid abnormality; serum high-density lipoprotein cholesterol <1.03 mmol/L in men and <1.29 mmol/L in women or specific treatment for this lipid abnormality; systolic blood pressure ≥130 mm Hg or diastolic blood pressure ≥85 mm Hg or treatment for previously diagnosed hypertension; and fasting plasma glucose ≥5.6 mmol/L or previously diagnosed type 2 diabetes.

For peer review only - http://bmjopen.bmj.com/site/about/guidelines.xhtml

Table S3 Prospective association of the Mediterranean diet score with the risk of hepatic steatosis, CoLaus study, Switzerland: assessment of influence of other potential covariates.

	Risk ratio (95% CI) across quintiles of Mediterranean diet score*					*P*-trend	Risk ratio (95% CI) Per SD increase*
	Q1	Q2	Q3	Q4	Q5		
Range	1.83-7.63	7.64-8.35	8.36-8.92	8.93-9.59	9.60-12.1		
N total	458	458	457	458	457		
Fatty liver index†							
Different models							
N Cases (score≥60)	36	43	35	22	17		
Multivariable (Model 1)‡	1.00 (ref.)	1.11 (0.72, 1.72)	1.07 (0.68, 1.66)	0.71 (0.42, 1.22)	0.50 (0.28, 0.91)	0.006	0.84 (0.71, 0.99)
Model 1 + Changes in BMI categories§	1.00 (ref.)	1.11 (0.73, 1.69)	0.98 (0.64, 1.50)	0.73 (0.43, 1.24)	0.59 (0.32, 1.06)	0.024	0.85 (0.72, 1.02)
Model 1 + Alcohol	1.00 (ref.)	1.11 (0.72, 1.72)	1.06 (0.69, 1.65)	0.71 (0.41, 1.22)	0.50 (0.28, 0.91)	0.006	0.84 (0.70, 0.99)
Model 1 + Alcohol+ BMI‖	1.00 (ref.)	1.13 (0.74, 1.73)	0.92 (0.59, 1.42)	0.76 (0.45, 1.29)	0.63 (0.35, 1.14)	0.047	0.86 (0.72, 1.04)
Model 1 + Clinical variables**	1.00 (ref.)	1.30 (0.80, 2.11)	1.20 (0.74, 1.94)	0.77 (0.43, 1.39)	0.57 (0.30, 1.09)	0.023	0.84 (0.70, 1.01)
Model 1 + Clinical variables + BMI	1.00 (ref.)	1.28 (0.79, 2.07)	0.94 (0.58, 1.52)	0.80 (0.45, 1.41)	0.68 (0.36, 1.27)	0.076	0.86 (0.71, 1.04)
Model 1 + MetS††	1.00 (ref.)	1.14 (0.74, 1.75)	1.03 (0.66, 1.60)	0.68 (0.40, 1.16)	0.51 (0.28, 0.93)	0.005	0.83 (0.70, 0.99)
NAFLD-score‡‡							
Different models							
N Cases (score≥-0.640)	41	46	51	38	32		
Multivariable (Model 1)‡	1.00 (ref.)	1.13 (0.75, 1.70)	1.26 (0.85, 1.87)	1.01 (0.65, 1.56)	0.80 (0.50, 1.28)	0.28	0.95 (0.82, 1.10)
Model 1 + Changes in BMI categories§	1.00 (ref.)	1.13 (0.76, 1.69)	1.18 (0.80, 1.74)	1.04 (0.68, 1.60)	0.93 (0.58, 1.48)	0.65	0.99 (0.86, 1.16)
Model 1 + Alcohol	1.00 (ref.)	1.11 (0.73, 1.67)	1.22 (0.82, 1.82)	0.97 (0.63, 1.51)	0.77 (0.48, 1.23)	0.21	0.94 (0.81, 1.09)
Model 1 + Alcohol+ BMI‖	1.00 (ref.)	1.16 (0.78, 1.73)	1.15 (0.78, 1.68)	1.04 (0.68, 1.61)	0.93 (0.58, 1.48)	0.62	0.99 (0.85, 1.15)
Model 1 + Clinical variables**	1.00 (ref.)	1.20 (0.78, 1.84)	1.32 (0.88, 1.98)	0.99 (0.64, 1.55)	0.85 (0.52, 1.37)	0.33	0.95 (0.82, 1.11)
Model 1 + Clinical variables + BMI	1.00 (ref.)	1.21 (0.80, 1.84)	1.19 (0.80, 1.78)	1.06 (0.69, 1.64)	0.99 (0.61, 1.60)	0.76	0.99 (0.85, 1.16)

Abbreviations: SES, socio-economic status; BMI, body mass index; MetS, metabolic syndrome; NAFLD, non-alcoholic fatty liver disease; SD, standard deviation; CI, confidence interval.

Statistical analysis using Poisson regression with robust standard errors; results are expressed as risk ratios and (95% confidence intervals).

* In categorical analysis, the population was divided into five groups by quintiles (Q1-Q5) of the Mediterranean diet score; Standard deviation was 1.20 for different multivariable analyses.

† Calculated based on an algorithm including BMI, waist circumference, triglycerides, and gamma-glutamyl transferase.

‡ Adjusted for age (years), sex, marital status (single, married/cohabitant, and widowed/Separated/divorced), occupational status (working and not working), education level (university, high school, apprenticeship, and mandatory education), smoking status (never, former, and current), energy intake (kcal/day), total energy expenditure (kcal/day), and date of dietary assessment.

§ Changes in BMI categories defined as individuals with a normal BMI at baseline and follow-up, individuals with normal BMI at baseline and overweight or obese BMI at follow-up, individuals with overweight or obese BMI at baseline and at follow-up, or individuals with overweight or obese BMI at baseline and normal at follow-up.

‖ Results of further adjustment for waist circumference were broadly in line with of the further adjustment for BMI (data not shown).

**Family history of diabetes (yes/no), high blood pressure (yes/no), high triglyceride level (yes/no), low HDL level (yes/no), and high glucose level (yes/no).

†† Metabolic syndrome as defined by the International Diabetes Federation (yes/no).

For peer review only - http://bmjopen.bmj.com/site/about/guidelines.xhtml

‡‡ Calculated based on an algorithm including presence of the metabolic syndrome and type 2 diabetes, and concentrations of fasting serum insulin, fasting serum aspartate-aminotransferase (AST), and the AST/alanine-aminotransferase ratio.

For peer review only

For peer review only - http://bmjopen.bmj.com/site/about/guidelines.xhtml

Table S4 Sensitivity analyses for the associations of the Mediterranean diet score with the risk of hepatic steatosis, CoLaus study, Switzerland.

	Risk ratio (95% CI) across quintiles of Mediterranean diet score*						
	Q1	Q2	Q3	Q4	Q5	*P*-trend	Risk ratio (95% CI) Per SD increase*
Range	1.83-7.63	7.64-8.35	8.36-8.92	8.93-9.59	9.60-12.1		
N total	458	458	457	458	457		
Fatty liver index†							
Different models							
N Cases (score≥60)	36	43	35	22	17		
Multivariable (Model 1)‡	1.00 (ref.)	1.11 (0.72, 1.72)	1.07 (0.68, 1.66)	0.71 (0.42, 1.22)	0.50 (0.28, 0.91)	0.006	0.84 (0.71, 0.99)
Excluding alcohol from MDS component							
Model 1 + Alcohol	1.00 (ref.)	1.64 (1.04, 2.58)	1.08 (0.65, 1.80)	1.17 (0.70, 1.96)	0.65 (0.35, 1.18)	0.069	0.86 (0.73, 1.03)
Model 1 + Alcohol + BMI§	1.00 (ref.)	1.53 (0.98, 2.39)	0.94 (0.57, 1.54)	1.08 (0.67, 1.77)	0.75 (0.41, 1.35)	0.15	0.89 (0.75, 1.06)
Excluding participants with BMI≥30‖							
N Cases (score≥60)	35	40	28	19	16		
Model 1	1.00 (ref.)	1.08 (0.69, 1.68)	0.90 (0.56, 1.46)	0.64 (0.36, 1.12)	0.50 (0.27, 0.93)	0.005	0.83 (0.69, 0.98)
Model 1 + BMI§	1.00 (ref.)	1.02 (0.66, 1.58)	0.84 (0.53, 1.33)	0.65 (0.37, 1.14)	0.60 (0.33, 1.10)	0.031	0.85 (0.70, 1.02)
Excluding participants with excessive alcohol consumption**							
N Cases (score≥60)	33	42	34	21	17		
Model 1	1.00 (ref.)	1.17 (0.75, 1.84)	1.12 (0.71, 1.77)	0.70 (0.40, 1.23)	0.52 (0.28, 0.95)	0.007	0.82 (0.69, 0.98)
Model 1 + BMI§	1.00 (ref.)	1.17 (0.76, 1.81)	0.91 (0.58, 1.42)	0.73 (0.42, 1.24)	0.64 (0.35, 1.15)	0.037	0.85 (0.71, 1.02)
Excluding participants with implausible energy intake††							
N Cases (score≥60)	33	41	33	22	16		
Model 1	1.00 (ref.)	1.19 (0.63, 2.23)	1.16 (0.61, 2.20)	0.87 (0.45, 1.69)	0.69 (0.35, 1.38)	0.16	0.85 (0.71, 1.01)
Model 1 + BMI§	1.00 (ref.)	1.13 (0.60, 2.13)	0.90 (0.47, 1.73)	0.81 (0.43, 1.53)	0.74 (0.37, 1.49)	0.20	0.86 (0.72, 1.04)
Excluding participants with secondary causes of hepatic steatosis‡‡							
N Cases (score≥60)	37	42	35	22	17		
Model 1	1.00 (ref.)	1.18 (0.75, 1.85)	1.11 (0.70, 1.76)	0.78 (0.45, 1.34)	0.51 (0.27, 0.94)	0.010	0.85 (0.71, 1.01)
Model 1 + BMI§	1.00 (ref.)	1.17 (0.75, 1.83)	0.95 (0.60, 1.50)	0.78 (0.46, 1.31)	0.63 (0.34, 1.17)	0.050	0.86 (0.72, 1.04)
Excluding participants with diabetes§§							
N Cases (score≥60)	35	40	35	22	16		
Model 1	1.00 (ref.)	1.06 (0.67, 1.66)	1.11 (0.71, 1.73)	0.74 (0.43, 1.27)	0.49 (0.26, 0.91)	0.009	0.84 (0.71, 0.998)
Model 1 + BMI§	1.00 (ref.)	1.06 (0.69, 1.65)	0.92 (0.59, 1.43)	0.76 (0.45, 1.29)	0.58 (0.32, 1.06)	0.033	0.85 (0.71, 1.02)
NAFLD-score ¶¶							
Different models							
N Cases (score≥-0.640)	41	46	51	38	32		
Multivariable (Model 1)‡	1.00 (ref.)	1.13 (0.75, 1.70)	1.26 (0.85, 1.87)	1.01 (0.65, 1.56)	0.80 (0.50, 1.28)	0.28	0.95 (0.82, 1.10)

For peer review only - http://bmjopen.bmj.com/site/about/guidelines.xhtml

Excluding alcohol from MD component							
Model 1 + Alcohol	1.00 (ref.)	1.15 (0.78, 1.71)	0.92 (0.61, 1.41)	0.92 (0.59, 1.42)	0.89 (0.57, 1.38)	0.34	0.93 (0.81, 1.08)
Model 1 + Alcohol + BMI§	1.00 (ref.)	1.15 (0.78, 1.69)	0.88 (0.59, 1.32)	0.88 (0.57, 1.35)	1.02 (0.66, 1.57)	0.65	0.96 (0.83, 1.12)
Excluding participants with BMI≥30‖							
N Cases (score≥-0.640)	40	46	44	36	31		
Model 1	1.00 (ref.)	1.15 (0.76, 1.74)	1.12 (0.74, 1.69)	0.99 (0.63, 1.55)	0.81 (0.5, 1.30)	0.27	0.94 (0.81, 1.09)
Model 1 + BMI§	1.00 (ref.)	1.17 (0.78, 1.75)	1.09 (0.73, 1.63)	1.07 (0.69, 1.67)	0.97 (0.6, 1.55)	0.76	0.99 (0.85, 1.15)
Excluding participants with excessive alcohol consumption**							
N Cases (score≥-0.640)	38	43	50	37	32		
Model 1	1.00 (ref.)	1.10 (0.73, 1.68)	1.28 (0.85, 1.91)	1.00 (0.64, 1.56)	0.80 (0.5, 1.29)	0.32	0.96 (0.83, 1.11)
Model 1 + BMI§	1.00 (ref.)	1.15 (0.76, 1.74)	1.18 (0.80, 1.73)	1.06 (0.69, 1.65)	0.96 (0.6, 1.54)	0.75	1.00 (0.86, 1.17)
Excluding participants with implausible energy intake††							
N Cases (score≥-0.640)	39	46	48	38	30		
Model 1	1.00 (ref.)	1.32 (0.75, 2.35)	1.48 (0.84, 2.60)	1.06 (0.59, 1.91)	1.15 (0.64, 2.07)	0.92	0.95 (0.82, 1.10)
Model 1 + BMI§	1.00 (ref.)	1.34 (0.76, 2.38)	1.35 (0.77, 2.37)	1.06 (0.59, 1.89)	1.29 (0.72, 2.33)	0.67	0.99 (0.85, 1.16)
Excluding participants with secondary causes of hepatic steatosis‡‡							
N Cases (score≥-0.640)	41	46	50	37	31		
Model 1	1.00 (ref.)	1.19 (0.79, 1.81)	1.26 (0.84, 1.89)	1.06 (0.68, 1.65)	0.78 (0.48, 1.27)	0.25	0.95 (0.82, 1.10)
Model 1 + BMI§	1.00 (ref.)	1.23 (0.82, 1.85)	1.17 (0.79, 1.73)	1.11 (0.71, 1.72)	0.93 (0.57, 1.52)	0.66	0.99 (0.85, 1.16)
Excluding participants with diabetes§§							
N Cases (score≥-0.640)	37	43	50	36	30		
Model 1	1.00 (ref.)	1.17 (0.76, 1.79)	1.37 (0.90, 2.06)	1.05 (0.67, 1.66)	0.85 (0.52, 1.39)	0.43	0.97 (0.83, 1.12)
Model 1 + BMI§	1.00 (ref.)	1.21 (0.79, 1.85)	1.26 (0.85, 1.87)	1.11 (0.71, 1.75)	1.01 (0.62, 1.63)	0.89	1.01 (0.86, 1.18)

Abbreviations: SES, socio economic status; BMI, body mass index; MD, Mediterranean Diet; NAFLD, non-alcoholic fatty liver disease; SD, standard deviation; CI, confidence interval.

Statistical analysis using Poisson regression with robust standard errors; results are expressed as risk ratios and (95% confidence intervals).

* In categorical analysis, the population was divided into five groups by quintiles (Q1-Q5) of the Mediterranean diet score; Standard deviation was 1.41 after excluding alcohol from Mediterranean Diet score components, 1.20 after excluding participants with BMI≥30, 1.18 after excluding participants with excessive alcohol consumption, 1.20 after excluding participants with probable implausible energy intake or after excluding participants with secondary causes of hepatic steatosis or diabetes.

† Calculated based on an algorithm including BMI, waist circumference, triglycerides, and gamma-glutamyl transferase.

‡ Adjusted for age (years), sex, marital status (single, married/cohabitant, and widowed/Separated/divorced), occupational status (working and not working), education level (university, high school, apprenticeship, and mandatory education), smoking status (never, former, and current), energy intake (kcal/day), total energy expenditure (kcal/day), and date of dietary assessment.

§ Results of further adjustment for waist circumference were broadly in line with of the further adjustment for BMI (data not shown).

‖ Excluded 36 participants with BMI≥30 kg/m^2.

** Excessive alcohol consumption defined as >21 units per week for men and >14 units per weeks for women; excluded 60 participants with excess alcohol consumption (n=2228).

†† Implausible energy intake defined as <500 and <800 kcal or >3,500 or >4,000 kcal in women and men, respectively; excluded 41 participants with probable implausible energy intake (n=2247).

For peer review only - http://bmjopen.bmj.com/site/about/guidelines.xhtml

‡‡ Secondary causes of hepatic steatosis defined as having hepatitis B, C or HIV, and with hepatotoxic medications (Glucocorticoids, isoniazid, methotrexate, amiodarone, and tamoxifen); excluded 36 participants with probable secondary causes of hepatic steatosis (n=2252).
§§ Diabetes defined as glycated haemoglobin≥48 mmol/mol, or fasting plasma glucose ≥7.0 mmol/L, or use of hypoglycaemic drugs or insulin; excluded 26 participants with probable secondary causes of hepatic steatosis (n=2262).
¶¶ Calculated based on an algorithm including presence of the metabolic syndrome and type 2 diabetes, and concentrations of fasting serum insulin, fasting serum aspartate-aminotransferase (AST), and the AST/alanine-aminotransferase ratio.

For peer review only

For peer review only - http://bmjopen.bmj.com/site/about/guidelines.xhtml

Table S5 Prospective association of the Mediterranean diet score with the risk of hepatic steatosis, CoLaus study, Switzerland: sensitivity analysis while excluding participants with only one of the indices high at baseline and using hepatic steatosis index as an alternative score.

	Risk ratio (95% CI) across quintiles of Mediterranean diet score*					*P*-trend	Risk ratio (95% CI) Per SD increase *
	Q1	Q2	Q3	Q4	Q5		
range	1.83-7.64	7.64-8.34	8.35-8.92	8.93-9.57	9.58-12.18		
N total (n=2652)	531	530	531	530	530		
Fatty liver index, *median* (iqr)†	23.4 (11.1, 41.4)	24.1 (10.6, 44.7)	22.3 (9.9, 39.7)	20.2 (8.4, 38.4)	17.5 (7.3, 34.5)		
N cases (score≥60)	51	56	54	35	24		
Unadjusted	1.00 (ref.)	1.10 (0.77, 1.58)	1.06 (0.74, 1.52)	0.69 (0.45, 1.04)	0.47 (0.29, 0.75)	<0.001	0.79 (0.69, 0.89)
Multivariable ‡	1.00 (ref.)	1.12 (0.77, 1.63)	1.25 (0.86, 1.81)	0.88 (0.57, 1.35)	0.56 (0.34, 0.92)	0.011	0.86 (0.74, 0.99)
Multivariable + BMI	1.00 (ref.)	1.08 (0.75, 1.54)	0.99 (0.70, 1.41)	0.85 (0.56, 1.30)	0.59 (0.36, 0.96)	0.017	0.84 (0.73, 0.98)
Multivariable + BMI & WC	1.00 (ref.)	1.01 (0.71, 1.45)	0.99 (0.70, 1.40)	0.84 (0.56, 1.28)	0.58 (0.35, 0.95)	0.019	0.84 (0.72, 0.98)
range	1.83-7.56	7.57-8.29	8.30-8.88	8.89-9.53	9.54-12.12		
N total (n=2568)	514	514	513	514	513		
NAFLD-score, *median* (iqr)§	-1.9 (-2.4, -1.3)	-1.8 (-2.3, -1.2)	-1.9 (-2.4, -1.3)	-2.0 (-2.5, -1.3)	-2.1 (-2.5, -1.6)		
N cases (score≥-0.640)	63	70	67	59	38		
Unadjusted	1.00 (ref.)	1.11 (0.81, 1.53)	1.07 (0.77, 1.47)	0.94 (0.67, 1.31)	0.60 (0.41, 0.89)	0.006	0.88 (0.79, 0.99)
Multivariable ‡	1.00 (ref.)	1.16 (0.83, 1.62)	1.16 (0.83, 1.61)	1.02 (0.72, 1.46)	0.66 (0.44, 1.00)	0.039	0.92 (0.81, 1.04)
Multivariable + BMI	1.00 (ref.)	1.27 (0.91, 1.76)	1.24 (0.89, 1.72)	1.16 (0.81, 1.65)	0.84 (0.55, 1.28)	0.34	0.98 (0.87, 1.11)
Multivariable + BMI & WC	1.00 (ref.)	1.25 (0.90, 1.73)	1.24 (0.89, 1.72)	1.16 (0.82, 1.65)	0.83 (0.54, 1.27)	0.34	0.99 (0.87, 1.12)
range	1.83-7.15	7.16-8.07	8.08-8.76	8.77-9.47	9.48-12.18		
N total (n=2351)	471	470	470	470	470		
Hepatic steatosis index, *median* (iqr)†	32.4 (30.5, 34.4)	32.3 (30.3, 34.6)	32.5 (30.4, 34.5)	32.6 (30.6, 34.4)	32.4 (30.3, 34.1)		
N cases (score>36)	166	123	120	120	103		
Unadjusted	1.00 (ref.)	0.74 (0.61, 0.90)	0.72 (0.59, 0.88)	0.72 (0.59, 0.88)	0.62 (0.50, 0.76)	<0.001	0.85 (0.79, 0.90)
Multivariable ‡	1.00 (ref.)	0.77 (0.61, 0.96)	0.79 (0.63, 1.00)	0.83 (0.65, 1.04)	0.70 (0.55, 0.91)	0.070	0.90 (0.82, 0.98)

Abbreviations: iqr, interquartile range; SES, socio-economic status; BMI, body mass index; WC, waist circumference; NAFLD, non-alcoholic fatty liver disease.
Statistical analysis using Poisson regression with robust standard errors; results are expressed as risk ratios and (95% confidence intervals).
* In categorical analysis, the population was divided into five groups by quintiles (Q1-Q5) of the Mediterranean diet score; Standard deviation was 1.21 for the multivariable analyses.
† Calculated based on an algorithm including BMI, waist circumference, triglycerides, and gamma-glutamyl transferase.
‡ Adjusted for age (years), sex, marital status (single, married/cohabiting, and widowed/Separated/divorced), occupational status (working and not working), education level (university, high school, apprenticeship, and mandatory education), smoking status (never, former, and current), energy intake (kcal/day), total energy expenditure (kcal/day), and date of dietary assessment.

For peer review only

§ Calculated based on an algorithm including presence of the metabolic syndrome and type 2 diabetes, and concentrations of fasting serum insulin, fasting serum aspartate-aminotransferase (AST), and the AST/alanine-aminotransferase ratio.

For peer review only

Table S6 Prospective association of the Mediterranean diet score with the risk of hepatic steatosis, CoLaus study, Switzerland (n=1632), Sensitivity analysis while excluding participants with either indices high (using FLI>30 instead of FLI≥60).

	Risk ratio (95% CI) across quintiles of Mediterranean diet score*					*P*-trend	Risk ratio (95% CI) Per SD increase*
	Q1	Q2	Q3	Q4	Q5		
range	1.83-7.63	7.64-8.35	8.36-8.92	8.93-9.59	9.60-12.1		
N total	327	326	327	326	326		
Fatty liver index, median (iqr)†	14.9 (7.6, 24.8)	12.7 (7.4, 23.7)	12.3 (5.5, 22.3)	11.8 (6.3, 22.7)	10.2 (5.5, 21.8)		
N cases (score≥60)	9	5	2	2	3		
Unadjusted	1.00 (ref.)	0.56 (0.19, 1.65)	0.22 (0.05, 1.02)	0.22 (0.05, 1.02)	0.33 (0.09, 1.22)	0.047	0.66 (0.45, 0.97)
Multivariable ‡	1.00 (ref.)	0.63 (0.20, 2.00)	0.14 (0.02, 0.96)	0.27 (0.05, 1.32)	0.39 (0.09, 1.68)	0.096	0.72 (0.47, 1.10)
Multivariable + BMI	1.00 (ref.)	0.68 (0.22, 2.05)	0.13 (0.02, 0.98)	0.26 (0.06, 1.15)	0.41 (0.10, 1.70)	0.087	0.73 (0.48, 1.11)
Multivariable + BMI + WC	1.00 (ref.)	0.50 (0.18, 1.42)	0.12 (0.01, 0.98)	0.22 (0.05, 1.03)	0.39 (0.09, 1.67)	0.093	0.71 (0.46, 1.09)
NAFLD-score, median (iqr)§	-2.2 (-2.5, -1.8)	-2.2 (-2.5, -1.7)	-2.2 (-2.5, -1.8)	-2.2 (-2.6, -1.8)	-2.2 (-2.6, -1.8)		
N cases (score≥-0.640)	13	14	10	13	12		
Unadjusted	1.00 (ref.)	1.08 (0.52, 2.26)	0.77 (0.34, 1.73)	1.00 (0.47, 2.13)	0.93 (0.43, 2.00)	0.79	0.94 (0.73, 1.20)
Multivariable ‡	1.00 (ref.)	1.13 (0.55, 2.32)	0.79 (0.35, 1.76)	1.10 (0.52, 2.34)	0.98 (0.45, 2.15)	0.94	0.95 (0.73, 1.24)
Multivariable + BMI	1.00 (ref.)	1.25 (0.60, 2.60)	0.81 (0.36, 1.84)	1.20 (0.56, 2.58)	1.13 (0.51, 2.52)	0.82	0.98 (0.75, 1.28)
Multivariable + BMI + WC	1.00 (ref.)	1.17 (0.57, 2.44)	0.85 (0.37, 1.93)	1.13 (0.52, 2.45)	1.12 (0.50, 2.53)	0.84	0.98 (0.75, 1.28)

Abbreviations: iqr, interquartile range; BMI, body mass index; WC, waist circumference; NAFLD, non-alcoholic fatty liver disease.
Statistical analysis using Poisson regression with robust standard errors; results are expressed as risk ratios and (95% confidence intervals).
* In categorical analysis, the population was divided into five groups by quintiles (Q1-Q5) of the Mediterranean diet score; Standard deviation was 1.21 for the multivariable analyses.
† Calculated based on an algorithm including BMI, waist circumference, triglycerides, and gamma-glutamyl transferase.
‡ Adjusted for age (years), sex, marital status (single, married/cohabiting, and widowed/Separated/divorced), occupational status (working and not working), education level (university, high school, apprenticeship, and mandatory education), smoking status (never, former, and current), energy intake (kcal/day), total energy expenditure (kcal/day), and date of dietary assessment.
§ Calculated based on an algorithm including presence of the metabolic syndrome and type 2 diabetes, and concentrations of fasting serum insulin, fasting serum aspartate-aminotransferase (AST), and the AST/alanine-aminotransferase ratio.

For peer review only - http://bmjopen.bmj.com/site/about/guidelines.xhtml

Table S7 Association of the Mediterranean diet score with change in BMI and waist circumference, CoLaus study, Switzerland.

	β coefficient (95% CI) across quintiles of Mediterranean diet score*					*P*-trend	β coefficient (95% CI) Per SD increase*
	Q1	Q2	Q3	Q4	Q5		
range	1.83-7.63	7.64-8.35	8.36-8.92	8.93-9.59	9.60-12.1		
N total	458	458	457	458	457		
ΔBMI, mean±SD†	0.48 ± 1.62	0.61 ± 1.53	0.42 ± 1.44	0.50 ± 1.52	0.40 ± 1.52		
Multivariable ‡	1.00 (ref.)	0.12 (-0.08, 0.33)	-0.08 (-0.28, 0.13)	-0.04 (-0.25, 0.16)	-0.16 (-0.37, 0.04)	0.038	-0.08 (-0.15, -0.02)
ΔWaist circumference, mean±SD§	1.04 ± 6.53	0.74 ± 6.42	0.19 ± 6.00	0.57 ± 6.33	0.17 ± 6.19		
Multivariable ‡	1.00 (ref.)	-0.51 (-1.36, 0.34)	-0.85 (-1.69, 0.00)	-0.47 (-1.32, 0.38)	-0.82 (-1.68, 0.03)	0.10	-0.33 (-0.61, -0.06)

Abbreviations: BMI, Body mass index.
Statistical analysis using linear regression; results are expressed as β coefficients and (95% confidence intervals).
* In categorical analysis, the population was divided into five groups by quintiles (Q1-Q5) of the Mediterranean diet score; Standard deviation was 1.20 for the multivariable analyses.
† Calculated by subtracting BMI at baseline from BMI at follow-up.
‡ Adjusted for age (years), sex, marital status (single, married/cohabitant, and widowed/Separated/divorced), occupational status (working and not working), education level (university, high school, apprenticeship, and mandatory education), smoking status (never, former, and current), energy intake (kcal/day), total energy expenditure (kcal/day), and date of dietary assessment.
§ Calculated by subtracting waist circumference at baseline from waist circumference at follow-up.

For peer review only

Table S8 Association of the Mediterranean diet score with the GGT, ALT, and AST, CoLaus study, Switzerland.

	β coefficient (95% CI) across quintiles of Mediterranean diet score*					*P*-trend	β coefficient (95% CI) Per SD increase *
	Q1	Q2	Q3	Q4	Q5		
range	1.83-7.63	7.64-8.35	8.36-8.92	8.93-9.59	9.60-12.12		
N total	458	458	457	458	457		
GGT (U/l), *median* (iqr)	21 (15, 29)	20 (14, 29)	19 (14, 26)	18 (14, 28)	17 (13, 26)		
Multivariable †	1.00 (ref.)	-2.63 (-9.01, 3.76)	-5.71 (-12.07, 0.66)	-4.24 (-10.64, 2.16)	-6.03 (-12.45, 0.38)	0.063	-1.53 (-3.60, 0.53)
Multivariable + BMI	1.00 (ref.)	-2.86 (-9.24, 3.53)	-5.70 (-12.06, 0.66)	-4.50 (-10.90, 1.90)	-6.52 (-12.95, -0.09)	0.047	-1.66 (-3.73, 0.41)
Multivariable + BMI & WC	1.00 (ref.)	-2.93 (-9.32, 3.45)	-5.72 (-12.08, 0.64)	-4.54 (-10.94, 1.86)	-6.53 (-12.96, -0.10)	0.047	-1.65 (-3.72, 0.41)
ALT (U/l), *median* (iqr)	20 (16, 26)	20.5 (17, 26)	20 (16, 26)	19.5 (16, 25)	20 (16, 25)		
Multivariable †	1.00 (ref.)	0.03 (-0.02, 0.08)	0.03 (-0.02, 0.08)	-0.013 (-0.06, 0.04)	0.002 (-0.05, 0.05)	0.45	-0.006 (-0.02, 0.01)
Multivariable + BMI	1.00 (ref.)	0.04 (-0.01, 0.08)	0.03 (-0.02, 0.08)	-0.009 (-0.06, 0.04)	0.008 (-0.04, 0.06)	0.60	-0.005 (-0.02, 0.01)
Multivariable + BMI & WC	1.00 (ref.)	0.04 (-0.01, 0.08)	0.03 (-0.02, 0.08)	-0.01 (-0.06, 0.04)	0.008 (-0.04, 0.06)	0.60	-0.005 (-0.02, 0.01)
AST (U/l), *median* (iqr)	22 (19, 25)	22 (19, 26)	22 (19, 25)	22 (19, 26)	22 (19, 25)		
Multivariable †	1.00 (ref.)	0.02 (-0.01, 0.06)	0.004 (-0.03, 0.04)	0.011 (-0.03, 0.05)	0.015 (-0.02, 0.05)	0.66	0.003 (-0.01, 0.01)
Multivariable + BMI	1.00 (ref.)	0.02 (-0.02, 0.06)	0.004 (-0.03, 0.04)	0.009 (-0.03, 0.04)	0.011 (-0.03, 0.05)	0.80	0.002 (-0.01, 0.01)
Multivariable + BMI & WC	1.00 (ref.)	0.02 (-0.02, 0.06)	0.004 (-0.03, 0.04)	0.009 (-0.03, 0.04)	0.011 (-0.03, 0.05)	0.80	0.002 (-0.01, 0.01)

Abbreviations: iqr, interquartile range; GGT, gamma-glutamyl transferase; SES, socio economic status; BMI, body mass index; WC, waist circumference; ALT, Alanine transaminase; AST, aspartate-aminotransferase.

Statistical analysis using linear regression; results are expressed as β coefficients and (95% confidence intervals).

* In categorical analysis, the population was divided into five groups by quintiles (Q1-Q5) of the Mediterranean diet score; Standard deviation was 1.20 for the multivariable analyses.

† Adjusted for age (years), sex, marital status (single, married/cohabitant, and widowed/Separated/divorced), occupational status (working and not working), education level (university, high school, apprenticeship, and mandatory education), smoking status (never, former, and current), energy intake (kcal/day), total energy expenditure (kcal/day), and date of dietary assessment.

For peer review only

STROBE 2007 (v4) Statement—Checklist of items that should be included in reports of *cohort studies*

Section/Topic	Item #	Recommendation	Reported on page #
**Title and abstract**	1	(*a*) Indicate the study's design with a commonly used term in the title or the abstract	3
		(*b*) Provide in the abstract an informative and balanced summary of what was done and what was found	3
**Introduction**			
Background/rationale	2	Explain the scientific background and rationale for the investigation being reported	6
Objectives	3	State specific objectives, including any prespecified hypotheses	7
**Methods**			
Study design	4	Present key elements of study design early in the paper	7
Setting	5	Describe the setting, locations, and relevant dates, including periods of recruitment, exposure, follow-up, and data collection	7
Participants	6	(*a*) Give the eligibility criteria, and the sources and methods of selection of participants. Describe methods of follow-up	7; Figure S1, and table S1
		(*b*) For matched studies, give matching criteria and number of exposed and unexposed	N/A
Variables	7	Clearly define all outcomes, exposures, predictors, potential confounders, and effect modifiers. Give diagnostic criteria, if applicable	Pages 7 to 10
Data sources/ measurement	8*	For each variable of interest, give sources of data and details of methods of assessment (measurement). Describe comparability of assessment methods if there is more than one group	Pages 7 to 10
Bias	9	Describe any efforts to address potential sources of bias	Pages 10 & 11
Study size	10	Explain how the study size was arrived at	12; Figure S1
Quantitative variables	11	Explain how quantitative variables were handled in the analyses. If applicable, describe which groupings were chosen and why	Pages 7 to 10
Statistical methods	12	(*a*) Describe all statistical methods, including those used to control for confounding	Pages 10 & 11
		(*b*) Describe any methods used to examine subgroups and interactions	Pages 10 & 11
		(*c*) Explain how missing data were addressed	Pages 10 & 11
		(*d*) If applicable, explain how loss to follow-up was addressed	Pages 10 & 11
		(*e*) Describe any sensitivity analyses	Pages 10 & 11

For peer review only

**Results**			
Participants	13*	(a) Report numbers of individuals at each stage of study—eg numbers potentially eligible, examined for eligibility, confirmed eligible, included in the study, completing follow-up, and analysed	12
		(b) Give reasons for non-participation at each stage	N/A
		(c) Consider use of a flow diagram	Figure 1
Descriptive data	14*	(a) Give characteristics of study participants (eg demographic, clinical, social) and information on exposures and potential confounders	12; Table 1 and table S1 & S2
		(b) Indicate number of participants with missing data for each variable of interest	12
		(c) Summarise follow-up time (eg, average and total amount)	12
Outcome data	15*	Report numbers of outcome events or summary measures over time	12; Table S2
Main results	16	(*a*) Give unadjusted estimates and, if applicable, confounder-adjusted estimates and their precision (eg, 95% confidence interval). Make clear which confounders were adjusted for and why they were included	Pages 12 & 13; Table 2, Figure 1, and supplementary Tables and Figures
		(*b*) Report category boundaries when continuous variables were categorized	Pages 12 & 13; Table 2, Figure 1, and supplementary Tables and Figures
		(*c*) If relevant, consider translating estimates of relative risk into absolute risk for a meaningful time period	
Other analyses	17	Report other analyses done—eg analyses of subgroups and interactions, and sensitivity analyses	Pages 13 & 14; Supplementary Tables S3 to S8
**Discussion**			
Key results	18	Summarise key results with reference to study objectives	14
**Limitations**	19	Discuss limitations of the study, taking into account sources of potential bias or imprecision.	pages 17 & 18
Interpretation	20	Give a cautious overall interpretation of results considering objectives, limitations, multiplicity of analyses, results from similar studies, and other relevant evidence	Pages 14 to 18
Generalisability	21	Discuss the generalisability (external validity) of the study results	pages 17 & 18
**Other information**			
Funding	22	Give the source of funding and the role of the funders for the present study and, if applicable, for the original study on	2

For peer review only

		which the present article is based	

*Give information separately for cases and controls in case-control studies and, if applicable, for exposed and unexposed groups in cohort and cross-sectional studies.

Note: An Explanation and Elaboration article discusses each checklist item and gives methodological background and published examples of transparent reporting. The STROBE checklist is best used in conjunction with this article (freely available on the Web sites of PLoS Medicine at http://www.plosmedicine.org/, Annals of Internal Medicine at http://www.annals.org/, and Epidemiology at http://www.epidem.com/). Information on the STROBE Initiative is available at www.strobe-statement.org.

For peer review only

The association between adherence to the Mediterranean diet and hepatic steatosis: the Swiss CoLaus prospective cohort study

Journal:	*BMJ Open*
Manuscript ID	bmjopen-2020-040959.R2
Article Type:	Original research
Date Submitted by the Author:	28-Oct-2020

Complete List of Authors:	Khalatbari-Soltani, Saman; The University of Sydney School of Public Health, Faculty of Medicine and Health; ARC Centre for Excellence in Population Ageing Research<br>Marques-Vidal, Pedro; University Hospital of Lausanne, Department of Internal Medicine<br>Imamura, Fumiaki; University of Cambridge School of Clinical Medicine, Medical Research Council Epidemiology Unit<br>Forouhi, Nita; University of Cambridge School of Clinical Medicine, Medical Research Council Epidemiology Unit

**Primary Subject Heading**:	Epidemiology
Secondary Subject Heading:	Nutrition and metabolism, Gastroenterology and hepatology, Public health
Keywords:	NUTRITION & DIETETICS, EPIDEMIOLOGY, PUBLIC HEALTH, Hepatology < INTERNAL MEDICINE

SCHOLARONE™ Manuscripts

I, the Submitting Author has the right to grant and does grant on behalf of all authors of the Work (as defined in the below author licence), an exclusive licence and/or a non-exclusive licence for contributions from authors who are: i) UK Crown employees; ii) where BMJ has agreed a CC-BY licence shall apply, and/or iii) in accordance with the terms applicable for US Federal Government officers or employees acting as part of their official duties; on a worldwide, perpetual, irrevocable, royalty-free basis to BMJ Publishing Group Ltd ("BMJ") its licensees and where the relevant Journal is co-owned by BMJ to the co-owners of the Journal, to publish the Work in this journal and any other BMJ products and to exploit all rights, as set out in our licence.

The Submitting Author accepts and understands that any supply made under these terms is made by BMJ to the Submitting Author unless you are acting as an employee on behalf of your employer or a postgraduate student of an affiliated institution which is paying any applicable article publishing charge ("APC") for Open Access articles. Where the Submitting Author wishes to make the Work available on an Open Access basis (and intends to pay the relevant APC), the terms of reuse of such Open Access shall be governed by a Creative Commons licence – details of these licences and which Creative Commons licence will apply to this Work are set out in our licence referred to above.

Other than as permitted in any relevant BMJ Author's Self Archiving Policies, I confirm this Work has not been accepted for publication elsewhere, is not being considered for publication elsewhere and does not duplicate material already published. I confirm all authors consent to publication of this Work and authorise the granting of this licence.

For peer review only

TITLE PAGE

Title: The association between adherence to the Mediterranean diet and hepatic steatosis: the Swiss CoLaus prospective cohort study

Saman Khalatbari-Soltani[1,2,3], Pedro Marques-Vidal[3], Fumiaki Imamura[4*], and Nita G. Forouhi[4*]

[1] The University of Sydney School of Public Health, Faculty of Medicine and Health, New South Wales, Australia; [2] ARC Centre of Excellence in Population Ageing Research (CEPAR), University of Sydney, Sydney, Australia; [3] Department of Internal Medicine, Internal Medicine, Lausanne University Hospital (CHUV), rue du Bugnon 46, 1011 Lausanne, Switzerland; [4] Medical Research Council Epidemiology Unit, University of Cambridge, School of Clinical Medicine, Institute of Metabolic Science, Cambridge, CB2 0QQ, UK.

*FL and NGF are joint senior authors with equal contribution

Correspondence to:

Saman Khalatbari-Soltani, ARC Centre of Excellence in Population Ageing Research (CEPAR), School of Public Heath, Faculty of Medicine and Health, the University of Sydney, NSW, 2006, Sydney Australia, +61 (0) 431 711 144, saman.khalatbarisoltani@sydney.edu.au.

Nita G. Forouhi, Medical Research Council Epidemiology Unit, University of Cambridge, School of Clinical Medicine, Institute of Metabolic Science, Cambridge, CB2 0QQ, UK, +44 (0) 1223 769145, nita.forouhi@mrc-epid.cam.ac.uk.

Running title: Mediterranean diet and hepatic steatosis.

Word count: 4090

For peer review only

For peer review only - http://bmjopen.bmj.com/site/about/guidelines.xhtml

Number of figures and tables: Two tables and one figure

For peer review only

For peer review only - http://bmjopen.bmj.com/site/about/guidelines.xhtml

ABSTRACT

Objective: The Mediterranean diet has been promoted as a healthy dietary pattern but, whether the Mediterranean diet may help to prevent hepatic steatosis is not clear. This study aimed to evaluate the prospective association between adherence to the Mediterranean diet and risk of hepatic steatosis.

Design: Population-based prospective cohort study.

Setting: The Swiss CoLaus study.

Participants: We evaluated 2288 adults (65.4% women, aged 55.8±10.0 years) without hepatic steatosis at first follow-up in 2009-2012. Adherence to the Mediterranean diet was scaled as the Mediterranean diet score (MDS) based on the Mediterranean-diet Pyramid ascertained with responses to food-frequency questionnaires.

Outcome measures: New onset of hepatic steatosis was ascertained by two indices separately: the fatty liver index (FLI, ≥60 points) and the non-alcoholic fatty liver disease (NAFLD) score (≥-0.640 points). Prospective associations between adherence to the Mediterranean diet and risk of hepatic steatosis were quantified using Poisson regression.

Results: During a mean 5.3-years of follow-up, hepatic steatosis was ascertained in 153 (6.7%) participants by FLI criteria and in 208 (9.1%) by NAFLD-score. After multivariable adjustment, higher adherence to MDS was associated with lower risk of hepatic steatosis based on FLI: risk ratio (95% confidence interval) 0.84 (0.73, 0.96) per one standard deviation of MDS; 0.85 (0.73, 0.99) adjusted for BMI; and 0.85 (0.71, 1.02) adjusted for both BMI and waist circumference. When using NAFLD-score, no significant association was found between MDS and risk of hepatic steatosis [0.95 (0.83, 1.09)].

Conclusion: A potential role of the Mediterranean diet in the prevention of hepatic steatosis is suggested by the inverse association observed between adherence to the Mediterranean diet and incidence of hepatic steatosis based on the FLI. The inconsistency of this association

For peer review only

For peer review only - http://bmjopen.bmj.com/site/about/guidelines.xhtml

when hepatic steatosis was assessed by NAFLD-score points to the need for accurate population-level assessment of fatty liver and its physiologic markers.

Keywords: Mediterranean diet; hepatic steatosis; fatty liver disease; prospective study.

For peer review only

For peer review only - http://bmjopen.bmj.com/site/about/guidelines.xhtml

STRENGHTS AND LIMITATION OF THE STUDY

- This study had the benefit of a relatively large sample size and an average of 5.3 years of follow-up.
- We applied a definition of the Mediterranean diet that has been shown to be valid in a non-Mediterranean population.
- Our ascertainment of hepatic steatosis was based on two indices that has been validated for use in large epidemiological studies.
- The precision of the two hepatic steatosis indices used is different and may be influenced by the presence of steatohepatitis or advanced liver fibrosis.
- Generalisability is limited because our findings relate to a single European population.

For peer review only

For peer review only - http://bmjopen.bmj.com/site/about/guidelines.xhtml

INTRODUCTION

Hepatic steatosis is the most common cause of liver disease [1]. In westernized countries, hepatic steatosis affects up to 34% of the general population and up to 74% of obese individuals, depending on the definition used [2–4]. Hepatic steatosis—fat content of more than 5% of liver volume—is the first recognizable stage of non-alcoholic fatty liver disease (NAFLD) [1]. Hepatic steatosis, particularly NAFLD, may progress to end-stage liver disease including fibrosis, cirrhosis, and hepatocellular carcinoma [5]. Moreover, as hepatic steatosis increases the risk of metabolic syndrome, type 2 diabetes, and cardiovascular disease (CVD), its prevention is of public health importance [6]. An unhealthy dietary pattern remains one of the primary targets of lifestyle modification for the prevention and management of hepatic steatosis and NAFLD [7,8].

The Mediterranean diet has been recently recommended for treatment of NAFLD [9]. In recent years, a growing body of evidence supports the idea that the Mediterranean diet may be the reference nutritional profile for the prevention of hepatic steatosis development [10–12]. Adherence to the Mediterranean diet has been reported to have a beneficial impact on risks of CVD [13,14], type 2 diabetes [15], and metabolic syndrome [16]. Trial evidence demonstrated the potential benefits of the Mediterranean diet against progress of hepatic steatosis focusing on individuals with existing hepatic steatosis, either alone [17–22] or associated with metabolic risk factors such as obesity or diabetes [22–25]. Research among those without clinically manifest hepatic steatosis is restricted to observational evidence, reporting an inverse association that greater adherence to a Mediterranean diet is associated with lower prevalence of hepatic steatosis [26,27]. However, the cross-sectional design of these studies limits inference for causal associations and can be used mainly for hypothesis generation. Relevant longitudinal evidence for the primary prevention of hepatic steatosis or NAFLD has been reported only by the Framingham Heart Study, with a significant inverse

For peer review only - http://bmjopen.bmj.com/site/about/guidelines.xhtml

association of adherence to the Mediterranean diet with risk of hepatic steatosis in 1521 adults over 6 years of follow-up [28], but evidence is lacking in Europe.

Given the limited evidence from population-based epidemiological studies thus far, we aimed to investigate the prospective association between adherence to the Mediterranean diet and the risk of developing hepatic steatosis among adults without clinically manifest hepatic steatosis. We hypothesized that greater adherence to the Mediterranean diet would reduce the risk of hepatic steatosis.

METHODS

Study population

We evaluated participants in the CoLaus study, an ongoing population-based cohort investigating the clinical, biological, and genetic determinants of CVD in the city of Lausanne, Switzerland [29]. Inclusion criteria of the recruitment were adults of European origin, aged 35 to 75 years [29]. There were three study phases: baseline recruitment in 2003-2006 (n=6733), the first follow-up in 2009-2012 (n=5064), and the second follow-up in 2014-2017 (n=4881). We conducted dietary assessment at the first follow-up and therefore considered the first follow-up as the study baseline. Fatty liver index (FLI) and NAFLD-score, two indices of hepatic steatosis, were available at baseline [27]. If participants met the joint criterion of FLI ≥ 60 or NAFLD-score ≥ -0.640 at baseline, we excluded them as prevalent cases (see below) (n=2036). We also excluded participants with missing information on diet, outcome, and covariates (n=740) (**Figure S1**).

Patient and public involvement

Patients and/or the public were not involved in the design, conduct, reporting or dissemination plans of this research.

For peer review only - http://bmjopen.bmj.com/site/about/guidelines.xhtml

Dietary assessment

Participants completed a self-administered, 97-item, semi-quantitative food frequency questionnaire (FFQ) about their habitual dietary intake over the last four weeks [30], the validity of which had been assessed in canton Geneva against 24-hour recalls [30,31]. For each item, participants were instructed to report consumption frequencies by selecting one of the seven frequency options from “less than once during the last 4 weeks” to “2 or more times per day” and by selecting a usual serving size (smaller, equal, or bigger to a reference size).

Mediterranean diet scores

We derived the pyramid-based Mediterranean diet score (MDS) as a measure of adherence to the Mediterranean diet from responses to the FFQ as we conducted previously [27]. This MDS is based on the Mediterranean Dietary Pyramid proposed by the Mediterranean Diet Foundation for both Mediterranean and non-Mediterranean countries and accounting for the traditional Mediterranean diet, contemporary lifestyle, and food environment [32]. We have previously reported that this MDS scoring algorithm predicted CVD incidence [33], as well as the prevalence of hepatic steatosis [27] in non-Mediterranean populations. Briefly, a continuous score of 0 to 1 was assigned for each recommended level of the 15 components of the pyramid (vegetables, legumes, and fish as healthy items; red meat, processed meat, potato, and sweets as unhealthy items; and fruits, nuts, cereals, eggs, dairy, white meat, and alcoholic beverages as items for which moderate consumption was recommended). The resulting MDS ranges between 0 and 15 on a continuous scale. The MDS calculation was adjusted to an energy intake of 2000 kcal/d (8.37 MJ/day) by applying a regression-residual technique for energy adjustment to each food group variable [33,34].

Ascertainment of hepatic steatosis

For peer review only - http://bmjopen.bmj.com/site/about/guidelines.xhtml

Two indices of hepatic steatosis were evaluated: fatty liver index (FLI) [35] and NAFLD liver fat score [36]. FLI was calculated based on a logistic function including body-mass index (BMI), waist circumference, fasting triglycerides, and gamma-glutamyl transferase (GGT) levels as follows:

$$FLI = 1 / (1+e^{-(0.953 \times \ln(\text{triglycerides}) + 0.139 \times BMI + 0.718 \times \ln(GGT) + 0.053 \times \text{waist circumference} - 15.745)})$$

FLI×100 ranges from 0 to 100. Presence of hepatic steatosis was defined by FLI ≥ 60, a value with a sensitivity of 61% and a specificity of 86% [35]. FLI was tested previously in comparison to ultrasonography with an area under the receiver operating characteristic curve of 0.78 (OR 95% CI: 0.77, 0.83) [37].

The NAFLD-score was calculated based on an algorithm including a logistic function with the presence of metabolic syndrome defined by criteria of International Diabetes Federation [38], presence of type 2 diabetes, and fasting concentrations of insulin, aspartate-aminotransferase (AST), and the AST/ alanine transaminase (ALT) ratio:

$$\text{NAFLD-score} = -2.89 + 1.18 \times \text{metabolic syndrome (yes/no)} + 0.45 \times \text{type 2 diabetes (yes /no)} + 0.15 \times \text{fasting-insulin (mU/L)} + 0.04 \times \text{fasting-AST (U/L)} - 0.94 \times AST/ALT$$

Presence of hepatic steatosis was defined by a NAFLD-score ≥ -0.640, a value with a sensitivity of 86% and a specificity of 71%, when compared to proton magnetic resonance imaging [36].

Assessment of covariates at baseline

Sociodemographic, lifestyle and health characteristics were collected by self-administered questionnaires. Age, sex, marital status, occupational status, and educational level were included as indicators of sociodemographic condition. Smoking status was classified as ‘never’, ‘former’, and ‘current’. Alcohol consumption was assessed by the

For peer review only - http://bmjopen.bmj.com/site/about/guidelines.xhtml

number of alcoholic beverage units consumed in the past week and further categorized as ‘abstainers’ (0 unit/week), ‘moderate’ (1–21 units/week for men, 1–14 for women), and ‘heavy’ (>21 units/week for men, >14 for women) drinkers (one unit corresponds to 8 g of alcohol). Physical activity was assessed with a self-administered quantitative physical activity frequency questionnaire [39]. Health characteristics included presence of metabolic syndrome and family history of diabetes. Anthropometric and blood pressure measurements were obtained using standard procedures and equipment as previously described [29]. Plasma triglycerides, high-density lipoprotein cholesterol, and glucose were measured using standard enzymatic methods and ALT, AST, and GGT were measured using reference methods as standardized by the International Federation of Clinical Chemistry.

Statistical analysis

Statistical analyses were performed using Stata (version 15; StataCorp, College Station, TX, USA) with a two-sided test with $\alpha=0.05$. Descriptive statistics were obtained in the participants included in this study in comparison to those excluded from this study. Cohen’s kappa statistics were calculated to assess the agreement between the FLI and NAFLD-score.

MDS as a measure of adherence to the Mediterranean diet was evaluated both categorically (quintiles) and continuously scaled as one SD unit. The association of MDS with the risk of hepatic steatosis was assessed using multivariable-adjusted Poisson regression models with robust standard errors and estimating risk ratios (RRs) and 95% confidence intervals (CIs). Models were adjusted for age, sex, marital status, occupational status, educational level, smoking status, energy intake, total energy expenditure, and date of dietary assessment (to adjust for seasonality). We further adjusted for BMI and waist circumference as potential confounders or factors on the causal pathway to assess the possible impact of

For peer review only - http://bmjopen.bmj.com/site/about/guidelines.xhtml

overall and central adiposity on the association of the Mediterranean diet and hepatic steatosis.

Additionally, we also adjusted for changes in BMI categories between baseline and follow-up; for alcohol consumption (units/week); for clinical variables of metabolic risk [blood pressure >130/85 mm Hg (yes/no), triglycerides >1.7 mmol/L (yes/no), high-density lipoprotein level <1.29 mmol/L for men and <1.03 mmol/L for women (yes/no), and glucose level ≥5.6 mmol/L (yes/no)] [38]; and for family history of diabetes and metabolic syndrome (only for FLI) to examine their influence on the association of interest.

Possible interactions between MDS and age, sex, BMI, and alcohol consumption were tested using the Wald test. Several sensitivity analyses were conducted to examine the robustness of the observed findings. First, to assess the role of alcohol consumption (as alcohol is a risk factor for fatty liver accumulation), we excluded the alcohol component from the MDS, while adjusting for alcohol consumption as a covariate. We took the same approaches for the other MDS components to assess the impact of each component on the observed associations. Second, we conducted separate analyses after excluding participants with BMI≥30 kg/m^2; implausible energy intake (<500 or >3500 kcal/day in women and <800 or >4000 kcal/day in men); excessive alcohol consumption; prevalent diabetes (defined as glycated haemoglobin≥48 mmol/mol, or fasting plasma glucose ≥7.0 mmol/L, or use of hypoglycaemic drugs or insulin); or probable secondary causes of hepatic steatosis such as hepatitis B or C, HIV, hepatotoxic, or autoimmune disease medications. We evaluated the robustness of the results to an alternative definition of prevalent hepatic steatosis. While we excluded participants with prevalent hepatic steatosis using the specified cut-offs of FLI or NAFLD-score in the primary analysis, we used each of the two indices separately in sensitivity analyses, whereby we evaluated 2652 adults in longitudinal analysis based on FLI;

For peer review only - http://bmjopen.bmj.com/site/about/guidelines.xhtml

and 2568 adults, based on NAFLD. Finally, we used more restrictive cutpoints and excluded participants with NAFLD-score ≥-0.640 or with FLI>30.

In a post-hoc analysis, due to inconsistency of the associations observed for FLI and NAFLD-score, we also calculated the hepatic steatosis index (HSI) [40] based on the ratio of AST/ALT, BMI, presence of type 2 diabetes, and sex:

HSI = 8*AST/ALT + BMI + 2 (presence of diabetes) + 2 (if women)

Presence of hepatic steatosis was defined by a HSI>36. After excluding participants with HSI>36 at baseline (n=2674), we evaluated 2351 adults.

In a post-hoc analysis pertaining to sensitivity of the results to model covariates and for better understanding of potential mechanisms, longitudinal associations of MDS with follow-up measures of log-transformed GGT, ALT, and AST levels and with changes in BMI and waist circumference from baseline to follow-up were examined using multivariable-adjusted linear regression. These results were expressed as β coefficient (95% CIs) for changes in each measure per 1-SD difference in MDS.

RESULTS

Participant characteristics

Of the initial 5064 participants, 2776 (54.8%) were excluded, leaving 2288 participants (65.4% women; 55.8±10.0 years) for analysis. Participants included were more likely to be women, show higher sociodemographic characteristics and lower BMI, waist circumference, liver enzymes, or prevalence of metabolic syndrome in comparison to excluded individuals (**Table S1**). Being in the highest quintile of MDS was higher among women compared to men, positively correlated with sociodemographic characteristics, and

For peer review only

For peer review only - http://bmjopen.bmj.com/site/about/guidelines.xhtml

negatively correlated with being current smokers, heavy alcohol drinkers, and with BMI, waist circumference, GGT, and TG (**Table 1**).

Adherence to the Mediterranean diet and risk of hepatic steatosis

After a mean 5.3 (SD: 0.5) years of follow-up, there were 153 (6.7%) and 208 (9.1%) participants with hepatic steatosis based on FLI and NAFLD-score, respectively (**Table S2**). Case identification by FLI and NAFLD-score was modestly concordant (kappa=0.60).

Multivariable-adjusted analysis showed an inverse association between MDS quintiles and risk of hepatic steatosis based on FLI (p_{trend}<0.006) with RR (95% CI) comparing the top to the bottom category of 0.50 (0.28, 0.91). The inverse associations across quintiles of MDS weakened after adjustment for BMI (p_{trend}=0.031) or both BMI and waist circumference (p_{trend}=0.034) (**Table 2**): RR (95% CI)=0.61 (0.34, 1.09) and 0.60 (0.34, 1.08), respectively. In analyses using MDS as a continuous variable, the inverse association with risk of hepatic steatosis based on FLI [RR 0.84 (0.73, 0.96) per one SD of MDS] remained unchanged but getting imprecise after adjustment for BMI [RR 0.85 (0.73, 0.99)] and adjustment for both BMI and waist circumference [0.85 (0.71, 1.02)]. In sensitivity analysis, the magnitude of the inverse associations little changed after further adjustment for alcohol consumption, presence of metabolic syndrome, changes in BMI categories or clinical variables (medication use or prevalent diseases), while adjustment for BMI and clinical variables increased standard errors (**Table S3**).

Conversely, there was no association between MDS and the risk of hepatic steatosis defined by NAFLD-score criteria, with RRs (95% CIs) ranging from 0.93 (0.82, 1.05) to 1.00 (0.86, 1.17) over different regression models (**Table 2** and **Table S3**).

Interaction and sensitivity analyses

For peer review only - http://bmjopen.bmj.com/site/about/guidelines.xhtml

No significant interactions were found between MDS and age, sex, BMI, or alcohol consumption on risk of hepatic steatosis ($p_{interaction}$>0.05; results not shown). The contribution of each component of the MDS on risk of hepatic steatosis was assessed by sequential subtraction of components from the score (**Figure 1**). Excluding the components of the MDS did not substantially affect the inverse associations with hepatic steatosis based on FLI; the magnitude of the associations remained reasonably stable, but it became weaker (p>0.05) after excluding fruits, cereals, dairy products, red or processed meat, or alcohol.

In sensitivity analyses, when excluding the alcohol component from the MDS but adjusting for alcohol consumption as a covariate, the inverse associations between MDS and risk of hepatic steatosis based on FLI became weaker (**Table S4**). The primary results were not different when excluding participants with BMI≥30 kg/m^2, excessive alcohol consumption, or secondary causes of hepatic steatosis (**Table S4**). Excluding participants with implausible energy intakes weakened the associations (**Table S4**). The analysis of an alternative definition of prevalent hepatic steatosis, excluding participants with only high FLI score at baseline, did not alter the significant inverse association between MDS and risk of FLI-based hepatic steatosis (**Table S5**). In post-hoc analyses, there was an inverse association between MDS quintiles and risk of hepatic steatosis based on HSI (p_{trend}=0.070) with RR (95% CI) comparing the top to the bottom category of 0.70 (0.55, 0.91); a significant inverse association with HSI was observed per one SD increase in MDS [RR 0.90 (0.82, 0.98)] (**Table S5**). Effect sizes were of slightly higher magnitude when excluding those with FLI>30 or NAFLD-score≥-0.640 at baseline, but CIs were wider due to smaller sample size (**Table S6**).

For NAFLD-score, no significant associations were found in any of the sensitivity analyses (**Tables S4 and S5**). The sole exception was when participants with a high NAFLD-score at baseline were excluded, where an inverse association between MDS quintiles and risk

For peer review only - http://bmjopen.bmj.com/site/about/guidelines.xhtml

of hepatic steatosis was present (p_{trend}=0.039), but this association was attenuated to the null after adjustment for BMI (**Table S5**).

Longitudinal analyses for adiposity and markers of hepatic function

In post-hoc exploratory analyses there were inverse associations of MDS with changes in BMI [β coefficient (95% CIs) per one SD higher MDS of -0.08 (-0.15, -0.02)] and in waist circumference [-0.33 (-0.61, -0.06)] (**Table S7**). For the markers of hepatic function, MDS showed a trend toward inverse association with GGT levels (p_{trend}=0.047) [β coefficient (95% CIs) per one SD higher MDS of -1.66 (-3.73, 0.41)], but not with ALT or AST levels (**Table S8**).

DISCUSSION

In this first population-based European study among adults free from clinically manifest hepatic steatosis to report on the prospective association between adherence to the Mediterranean diet and risk of hepatic steatosis, we found an inverse association between MDS and risk of hepatic steatosis based on FLI criteria. This relationship was attenuated to the null when controlled for general and central adiposity assessed by the BMI and waist circumference. In contrast, there was no association between adherence to the Mediterranean diet and risk of hepatic steatosis based on NAFLD-score criteria.

Current findings in context of other evidence

Our finding based on FLI is consistent with the only published prospective study relating the Mediterranean diet to hepatic steatosis, where each SD increase in MDS was estimated to decrease the odds for incident hepatic steatosis by 26% (95% CI 10%-39%) [28]. By contrast, the point estimate of the effect size in our study was smaller [(16% (95% CI 4%-27%)]. A possible explanation partly lies in methodological differences. We used biochemical

For peer review only - http://bmjopen.bmj.com/site/about/guidelines.xhtml

and anthropometric markers to estimate hepatic steatosis in the current study, while the previous study used computed tomography assessment.

No association was found between adherence to the Mediterranean diet and risk of hepatic steatosis based on the NAFLD-score. For possible explanations based on the differences in their components, the FLI includes GGT, while NAFLD-score includes AST and AST/ALT ratio; the FLI includes adiposity markers, while the NAFLD-score does not; the FLI includes a lipid marker (triglycerides) while NAFLD-score includes markers of glycaemic status. Previous studies showed a modest association of GGT and ALT (but not AST) with the prevalence of hepatic steatosis [1]. Indeed, our analysis showed an inverse association between MDS and GGT levels, but not with AST or ALT, and these findings could explain the discrepancy between the two indices. Notably, our sensitivity analyses using different definitions of hepatic steatosis showed an inverse association between MDS quintiles and risk of hepatic steatosis based on NAFLD-score after excluding participants with high baseline NAFLD-score only. This could be explained by modest concordance between the two measures. Of note, there were no statistically significant associations between MDS and risk of hepatic steatosis based on FLI after excluding participants with FLI>30 at baseline, which led to a smaller sample size and consequently a lower statistical power in our study. In a post-hoc analysis, we found an inverse association between adherence to the Mediterranean diet and hepatic steatosis as assessed by HSI, an alternative score to detect hepatic steatosis. Although, case identification by HSI and FLI (kappa=0.27) or NAFLD-score (Kappa=0.18) was weakly concordant; our finding based on FLI is consistent with HSI. This could be explained potentially by BMI as one of the components of both FLI and HSI, highlighting the importance of obesity for incident hepatic steatosis [41].

The inverse association between MDS and risk of hepatic steatosis defined by FLI remained significant after adjusting for BMI only but became imprecise and not significant

For peer review only - http://bmjopen.bmj.com/site/about/guidelines.xhtml

after adjusting for both BMI and waist circumference or for changes in BMI over the follow-up period. These results suggest the collinearity between the central adiposity and hepatic steatosis as the central adiposity may partly reflect hepatic steatosis. This biologically plausible finding is in agreement with previous cross-sectional findings for MDS and prevalent hepatic steatosis in CoLaus study and the British Fenland Study we reported [27] and a study in Hong Kong [42]. Our finding that the MDS was negatively associated with an increase in BMI after 5.3 years of follow-up is consistent with the findings from the Framingham Heart Study which observed the same trend over 6-year follow-up [28]; and from Nurses' Health Study and Health Professionals' Follow-up Study evaluating 20-year longitudinal data with repeated self-reported measures of diet and adiposity measures [43].

Sequential subtraction of different components of the MDS showed that fruits, cereals, dairy products, red or processed meat, or alcohol partially accounted for the observed association. These findings agree with previous studies [26,28,44,45] including the Framingham Heart Study suggesting benefits of low consumption of red meat and high consumption of fruits or whole grains [28]; and a cross-sectional analysis from the PREDIMED study suggesting the benefit of low consumption of red meat [26]. Moreover, the Mediterranean diet is characterized by a moderate-to-high consumption of whole grains, which has been inversely associated with the likelihood of having NAFLD [46].

Possible mechanisms and implications

Hepatic steatosis is associated with a number of metabolic risk factors including insulin resistance, type 2 diabetes, dyslipidaemia, metabolic syndrome, and oxidative stress [47]. Mediterranean-diet associated phenolic compounds (phenolic acids and polyphenols) found in fruits and vegetables and high levels of monounsaturated fatty acids of olive oil have been shown to inhibit *de* novo lipogenesis, improve peripheral insulin sensitivity, and reduce

For peer review only

For peer review only - http://bmjopen.bmj.com/site/about/guidelines.xhtml

cardiovascular risk mainly due to their antioxidant, anti-inflammatory, and anti-fibrotic effects [18,48–51]. Moreover, different components of the Mediterranean diet, including omega 3 polyunsaturated fatty acids (PUFA), fibre, and antioxidant rich-foods, are inversely associated with hepatic steatosis [52,53]. One meta-analysis of interventional studies reported that omega-3 PUFA were negatively associated with hepatic steatosis [54]. The Mediterranean diet is also low in saturated fat, which has been demonstrated to increase hepatic triglycerides content and hepatic insulin resistance [55,56]. Finally, the high fibre content of the Mediterranean diet has been associated with reduced hepatic fat [18,52].

Hepatic steatosis is associated with cardiometabolic diseases and substantially impacts public health [13,15,16]. Thus, our finding of an inverse association between adherence to the Mediterranean diet and risk of hepatic steatosis would support the importance of dietary advice for the prevention of hepatic steatosis as well as its treatment. However, future work should confirm whether or not the clinical importance of the Mediterranean diet for the prevention of hepatic steatosis is independent of obesity or central adiposity.

Strengths and *limitations*

To our knowledge, this is the first European prospective study assessing the association between the Mediterranean diet and risk of hepatic steatosis. The study had the benefit of a relatively large sample size and an average of 5.3 years of follow-up, and applied a definition of the Mediterranean diet that has been shown to be valid in a non-Mediterranean population [33].

Several limitations of this study merit consideration. Measurement error and recall bias are inevitable when using self-reported dietary instruments, limiting the ability to precisely measure adherence to the Mediterranean diet, although adjustment for energy intake may have reduced the magnitude of measurement error [57]. We used diet data measured only

For peer review only

For peer review only - http://bmjopen.bmj.com/site/about/guidelines.xhtml

at baseline but recognise that intra-individual variation over time might be present which would be expected to weaken the observed associations and hence our findings may be biased towards the null. However, in CoLaus, average change in estimated total energy intake from first to second follow-up was 51 kcal/day and changes for each macronutrient (expressed as % of total energy intake) were about 1% (data not shown). Thus, dietary intake in CoLaus was relatively stable, suggesting that the lack of availability of repeat dietary measures is unlikely to alter our findings substantially.

Our ascertainment of hepatic steatosis was based on two indices, but not on liver biopsy or direct imaging assessment; hence, information bias (misdiagnosis) cannot be ruled out. Although liver biopsy is the gold standard for diagnosing hepatic steatosis, its use in apparently healthy participants would be unethical and unfeasible in a large population-based study. Further, previous studies have shown that FLI and NAFLD-score can accurately identify hepatic steatosis with good sensitivity and specificity, and these scores have been validated for use in large epidemiological studies [36,37,58,59]. But, although both FLI and NAFLD-score can indicate hepatic steatosis, their precision is variable [60]. Moreover, presence of steatohepatitis or advanced liver fibrosis could influence the relationship of FLI or NAFLD-score with hepatic steatosis [61]. Of note, our study was not designed to assess the role of adherence to the Mediterranean diet in hepatic fibrosis.

Although we adjusted for many relevant confounders and performed a series of sensitivity analyses, we cannot rule out residual confounding due to unmeasured variables or covariates measured with error. Generalisability is limited because included participants seemed to be healthier than those excluded, and our findings were obtained in a single European population. Still, they confirm the findings from a previous prospective study conducted in the US [28], and might serve as a reference for other studies.

For peer review only - http://bmjopen.bmj.com/site/about/guidelines.xhtml

Conclusion

Adherence to the Mediterranean diet was inversely associated with risk of hepatic steatosis based on the FLI, and the association was independent of several known risk factors. Conversely, the association was not observed when using different criteria specifying the NAFLD-score. These findings support recommendations on following the Mediterranean diet for hepatic steatosis prevention in addition to the existing evidence for its benefit for cardiovascular disease prevention. Nonetheless, the findings also highlight the need for further research with more accurate measures of hepatic steatosis to replicate these findings in different populations and settings.

For peer review only - http://bmjopen.bmj.com/site/about/guidelines.xhtml

ABBREVIATIONS

NAFLD, non-alcoholic fatty liver disease; CVD; cardiovascular disease; FFQ, food frequency questionnaire; MDS, Mediterranean diet score; FLI, fatty liver index; BMI, body-mass index; TG, triglyceride; GGT, gamma-glutamyl transferase; AST, aspartate-aminotransferase; ALT, alanine transaminase; PUFA, polyunsaturated fatty acids.

DECLARATION

Acknowledgements: The authors are grateful to all the participants and staff of CoLaus study.

Competing interests: None.

Ethics approval and consent to participate: The institutional Ethics Committee of the University of Lausanne, which afterwards became the Ethics Commission of Canton Vaud (www.cer-vd.ch) approved the baseline CoLaus study (reference 16/03); the approval was renewed for the first (reference 33/09) and the second (reference 26/14) follow-ups.

The CoLaus study was performed in agreement with the Helsinki declaration and its former amendments, and all participants provided their written informed consent before entering the study.

Funding: The CoLaus study was and is supported by research grants from GlaxoSmithKline, the Faculty of Biology and Medicine of Lausanne, and the Swiss National Science Foundation (grants 33CSCO-122661, 33CS30-139468 and 33CS30-148401). SKS was supported by the Swiss National Science Foundation (Doc.Mobility number P1LAP3-171805). NGF and FI acknowledge core MRC support (MC_UU_12015/5), and NGF acknowledges NIHR

For peer review only

Biomedical Research Centre Cambridge: Nutrition, Diet, and Lifestyle Research Theme (IS-BRC-1215-20014).

Authors' contribution: SKS, FI, and NGF designed the study question and had full access to all the data in the study and took responsibility for the integrity and accuracy of the data. SKS performed the statistical analyses and she wrote the first draft with supervision from FI, PMV, and NGF. All authors: contributed to interpretation of data, revised the article critically for important intellectual content, and approved the final version of the manuscript.

Data availability statement: Non-identifiable individual-level data are available for researchers who seek to answer questions related to health and disease in the context of research projects who meet the criteria for data sharing by research committees. Please follow the instructions at https://www.colaus-psycolaus.ch/ for information on how to submit an application for gaining access to CoLaus data.

For peer review only

For peer review only - http://bmjopen.bmj.com/site/about/guidelines.xhtml

SUPPLEMENTARY INFORMATION

Figure S1 Sample selection flow chart.

Table S1 Characteristics of the participants included and excluded from the analysis, CoLaus study, Switzerland.

Table S2 Baseline characteristics of the participants according to the risk of hepatic steatosis, defined by the fatty liver index or the NAFLD-score, CoLaus study, Switzerland.

Table S3 Prospective association of the Mediterranean diet score with the risk of hepatic steatosis, CoLaus study, Switzerland: assessment of influence of other potential covariates.

Table S4 Sensitivity analyses for the association of the Mediterranean diet score with the risk of hepatic steatosis, CoLaus study, Switzerland.

Table S5 Prospective association of the Mediterranean diet score with the risk of hepatic steatosis, CoLaus study, Switzerland: sensitivity analysis while excluding participants with only one of the indices high at baseline and using hepatic steatosis index as an alternative score.

Table S6 Prospective association of the Mediterranean diet score with the risk of hepatic steatosis, CoLaus study, Switzerland (n=1632). Sensitivity analysis after excluding participants with either indices high (using FLI>30 instead of FLI≥60).

Table S7 Association of the Mediterranean diet score with change in BMI and waist circumference, CoLaus study, Switzerland.

Table S8 Association of the Mediterranean diet score with GGT, ALT, and AST, CoLaus study, Switzerland.

For peer review only

For peer review only - http://bmjopen.bmj.com/site/about/guidelines.xhtml

Figure caption

Figure 1 Prospective association of the Mediterranean diet score with the risk of hepatic steatosis, CoLaus study, Switzerland (n=2288): sensitivity analysis to examine influence of each component of Mediterranean diet.

Abbreviations: FLI, fatty liver index; NAFLD, non-alcoholic fatty liver disease.

Statistical analysis using Poisson regression with robust standard errors; results are expressed as risk ratios and (95% confidence intervals).

Risk ratio (RR) and 95% confidence intervals (CI) were estimated per one standard deviation of MDS (overall association) or of each MDS computed after excluding one component.

* Adjusted for age (years), sex, marital status (single, married/cohabiting, and widowed/Separated/divorced), occupational status (working and not working), education level (university, high school, apprenticeship, and mandatory education), smoking status (never, former, and current), energy intake (kcal/day), total energy expenditure (kcal/day), and date of dietary assessment.

For peer review only

For peer review only - http://bmjopen.bmj.com/site/about/guidelines.xhtml

REFERENCES

1 Sattar N, Forrest E, Preiss D. Non-alcoholic fatty liver disease. *BMJ* 2014;**349**. doi:10.1136/bmj.g4596

2 Angulo P. Nonalcoholic fatty liver disease. *N Engl J Med* 2002;**346**:12–9. doi:10.1056/NEJMra011775

3 Williamson RM, Price JF, Glancy S, *et al.* Prevalence of and Risk Factors for Hepatic Steatosis and Nonalcoholic Fatty Liver Disease in People With Type 2 Diabetes: the Edinburgh Type 2 Diabetes Study. *Diabetes Care* 2011;**34**:1139 LP – 1144.http://care.diabetesjournals.org/content/34/5/1139.abstract

4 Bellentani S, Scaglioni F, Marino M, *et al.* Epidemiology of non-alcoholic fatty liver disease. *Dig Dis* 2010;**28**:155–61. doi:10.1159/000282080

5 Angulo P, Keach JC, Batts KP, *et al.* Independent predictors of liver fibrosis in patients with nonalcoholic steatohepatitis. *Hepatology* 1999;**30**:1356–62. doi:10.1002/hep.510300604

6 Marchesini G, Petta S, Grave RD. Diet, Weight Loss, and Liver Health in Nonalcoholic Fatty Liver Disease: Pathophysiology, Evidence, and Practice. *Hepatology* 2016;**63plos**:2032–43. doi:10.1002/hep.28392

7 Marchesini G, Day CP, Dufour JF, *et al.* EASL–EASD–EASO Clinical Practice Guidelines for the management of non-alcoholic fatty liver disease. *J Hepatol* 2016;**64**:1388–402. doi:10.1016/j.jhep.2015.11.004

8 Chalasani N, Younossi Z, Lavine JE, *et al.* The diagnosis and management of non-alcoholic fatty liver disease: practice Guideline by the American Association for the Study of Liver Diseases, American College of Gastroenterology, and the American Gastroenterological Association. *Hepatology* 2012;**55**:2005–23. doi:10.1002/hep.25762

9 Sawangjit R, Chongmelaxme B, Phisalprapa P, *et al.* Comparative efficacy of interventions on nonalcoholic fatty liver disease (NAFLD): A PRISMA-compliant systematic review and network meta-analysis. *Med* 2016;**95**:e4529. doi:10.1097/md.0000000000004529

10 Plauth M, Bernal W, Dasarathy S, *et al.* ESPEN guideline on clinical nutrition in liver disease. *Clin Nutr* 2019;**38**:485–521. doi:https://doi.org/10.1016/j.clnu.2018.12.022

For peer review only

For peer review only - http://bmjopen.bmj.com/site/about/guidelines.xhtml

11 Abenavoli L, Di Renzo L, Boccuto L, *et al.* Health benefits of Mediterranean diet in nonalcoholic fatty liver disease. *Expert Rev Gastroenterol Hepatol* 2018;**12**:873–81. doi:10.1080/17474124.2018.1503947

12 Asbaghi O, Choghakhori R, Ashtary-Larky D, *et al.* Effects of the Mediterranean diet on cardiovascular risk factors in non-alcoholic fatty liver disease patients: A systematic review and meta-analysis. *Clin Nutr ESPEN* 2020;**37**:148–56. doi:10.1016/j.clnesp.2020.03.003

13 Estruch R, Ros E, Salas-Salvadó J, *et al.* Primary Prevention of Cardiovascular Disease with a Mediterranean Diet Supplemented with Extra-Virgin Olive Oil or Nuts. *N Engl J Med* 2018;**378**:e34. doi:10.1056/NEJMoa1800389

14 Sofi F, Cesari F, Abbate R, *et al.* Adherence to Mediterranean diet and health status: meta-analysis. *BMJ* 2008;**337**:a1344. doi:10.1136/bmj.a1344

15 Salas-Salvado J, Bullo M, Babio N, *et al.* Reduction in the incidence of type 2 diabetes with the Mediterranean diet: results of the PREDIMED-Reus nutrition intervention randomized trial. *Diabetes Care* 2011;**34**:14–9. doi:10.2337/dc10-1288

16 Esposito K, Kastorini C-M, Panagiotakos DB, *et al.* Mediterranean diet and metabolic syndrome: an updated systematic review. *Rev Endocr Metab Disord* 2013;**14**:255–63. doi:10.1007/s11154-013-9253-9

17 Ryan MC, Itsiopoulos C, Thodis T, *et al.* The Mediterranean diet improves hepatic steatosis and insulin sensitivity in individuals with non-alcoholic fatty liver disease. *J Hepatol* 2013;**59**:138–43. doi:10.1016/j.jhep.2013.02.012

18 Properzi C, O'Sullivan TA, Sherriff JL, *et al.* Ad Libitum Mediterranean and Low-Fat Diets Both Significantly Reduce Hepatic Steatosis: A Randomized Controlled Trial. *Hepatology* 2018;**68**:1741–54. doi:10.1002/hep.30076

19 Misciagna G, del Pilar Diaz M, Caramia D V, *et al.* Effect of a low glycemic index Mediterranean diet on non-alcoholic fatty liver disease. A randomized controlled clinici trial. *J Nutr Heal AGING* 2017;**21**:404–12. doi:10.1007/s12603-016-0809-8

20 Trovato FM, Catalano D, Martines GF, *et al.* Mediterranean diet and non-alcoholic fatty liver disease: the need of extended and comprehensive interventions. *Clin Nutr* 2015;**34**:86–8.

For peer review only - http://bmjopen.bmj.com/site/about/guidelines.xhtml

doi:10.1016/j.clnu.2014.01.018

21 Gelli C, Tarocchi M, Abenavoli L, *et al.* Effect of a counseling-supported treatment with the Mediterranean diet and physical activity on the severity of the non-alcoholic fatty liver disease. *World J Gastroenterol* 2017;**23**:3150–62. doi:10.3748/wjg.v23.i17.3150

22 Baratta F, Pastori D, Polimeni L, *et al.* Adherence to mediterranean diet and non-alcoholic fatty liver disease: Impact on metabolic profile. *Nutr Metab Cardiovasc Dis* 2017;**27 (1)**:e8.http://ovidsp.ovid.com/athens/ovidweb.cgi?T=JS&CSC=Y&NEWS=N&PAGE=fulltext&D=emex&AN=614265212

23 Abenavoli L, Greco M, Nazionale I, *et al.* Effects of Mediterranean diet supplemented with silybin-vitamin E-phospholipid complex in overweight patients with non-alcoholic fatty liver disease. *Expert Rev Gastroenterol Hepatol* 2015;**9**:519–27. doi:10.1586/17474124.2015.1004312

24 Fraser A, Abel R, Lawlor DA, *et al.* A modified Mediterranean diet is associated with the greatest reduction in alanine aminotransferase levels in obese type 2 diabetes patients: results of a quasi-randomised controlled trial. *Diabetologia* 2008;**51**:1616–22. doi:10.1007/s00125-008-1049-1

25 Kontogianni MD, Tileli N, Margariti A, *et al.* Adherence to the Mediterranean diet is associated with the severity of non-alcoholic fatty liver disease. *Clin Nutr* 2014;**33**:678–83. doi:10.1016/j.clnu.2013.08.014

26 Cantero I, Abete I, Babio N, *et al.* Dietary Inflammatory Index and liver status in subjects with different adiposity levels within the PREDIMED trial. *Clin Nutr* Published Online First: 2017. doi:http://dx.doi.org/10.1016/j.clnu.2017.06.027

27 Khalatbari-Soltani S, Imamura F, Brage S, *et al.* The association between adherence to the Mediterranean diet and hepatic steatosis: cross-sectional analysis of two independent studies, the UK Fenland Study and the Swiss CoLaus Study. *BMC Med* 2019;**17**:19. doi:10.1186/s12916-019-1251-7

28 Ma J, Hennein R, Liu C, *et al.* Improved Diet Quality Associates With Reduction in Liver Fat, Particularly in Individuals With High Genetic Risk Scores for Nonalcoholic Fatty Liver

For peer review only - http://bmjopen.bmj.com/site/about/guidelines.xhtml

Disease. *Gastroenterology* 2018;**155**:107–17. doi:10.1053/j.gastro.2018.03.038

29 Firmann M, Mayor V, Vidal PM, *et al.* The CoLaus study: a population-based study to investigate the epidemiology and genetic determinants of cardiovascular risk factors and metabolic syndrome. *BMC Cardiovasc Disord* 2008;**8**:6. doi:10.1186/1471-2261-8-6

30 Morabia A, Bernstein M, Kumanyika S, *et al.* Développement et validation d'un questionnaire alimentaire semi-quantitatif à partir d'une enquête de population. *Soz Praventivmed* 1994;**39**:345–69. doi:10.1007/BF01299666

31 Bernstein L, Huot I MA, Bernstein L, Huot I, *et al.* Amélioration des performances d'un questionnaire alimentaire semi-quantitatif comparé à un rappel des 24 heures. *Sante Publique (Paris)* 1995;**7**:403–13.

32 Bach-Faig A, Berry EM, Lairon D, *et al.* Mediterranean diet pyramid today. Science and cultural updates. *Public Health Nutr* 2011;**14**:2274–84. doi:10.1017/S1368980011002515

33 Tong TYN, Wareham NJ, Khaw K-T, *et al.* Prospective association of the Mediterranean diet with cardiovascular disease incidence and mortality and its population impact in a non-Mediterranean population: the EPIC-Norfolk study. *BMC Med* 2016;**14**:135. doi:10.1186/s12916-016-0677-4

34 Willett WC, Howe GR, Kushi LH. Adjustment for total energy intake in epidemiologic studies. *Am J Clin Nutr* 1997;**65**:1220S-1228S; discussion 1229S-1231S.

35 Bedogni G, Bellentani S, Miglioli L, *et al.* The Fatty Liver Index: a simple and accurate predictor of hepatic steatosis in the general population. *BMC Gastroenterol* 2006;**6**:33. doi:10.1186/1471-230X-6-33

36 Kotronen A, Peltonen M, Hakkarainen A, *et al.* Prediction of Non-Alcoholic Fatty Liver Disease and Liver Fat Using Metabolic and Genetic Factors. *Gastroenterology* 2009;**137**:865–72. doi:http://dx.doi.org/10.1053/j.gastro.2009.06.005

37 Ruhl CE, Everhart JE. Fatty liver indices in the multiethnic United States National Health and Nutrition Examination Survey. *Aliment Pharmacol Ther* 2015;**41**:65–76. doi:10.1111/apt.13012

38 Alberti KGMM, Zimmet P, Shaw J. Metabolic syndrome--a new world-wide definition. A

For peer review only - http://bmjopen.bmj.com/site/about/guidelines.xhtml

For peer review only

Consensus Statement from the International Diabetes Federation. *Diabet Med* 2006;**23**:469–80. doi:10.1111/j.1464-5491.2006.01858.x

39 Bernstein M, Sloutskis D, Kumanyika S, *et al.* Data-based approach for developing a physical activity frequency questionnaire. *Am J Epidemiol* 1998;**147**:147–54.

40 Lee J-H, Kim D, Kim HJ, *et al.* Hepatic steatosis index: a simple screening tool reflecting nonalcoholic fatty liver disease. *Dig liver Dis Off J Ital Soc Gastroenterol Ital Assoc Study Liver* 2010;**42**:503–8. doi:10.1016/j.dld.2009.08.002

41 Stefan N, Häring H-U, Cusi K. Non-alcoholic fatty liver disease: causes, diagnosis, cardiometabolic consequences, and treatment strategies. *lancet Diabetes Endocrinol* 2019;**7**:313–24. doi:10.1016/S2213-8587(18)30154-2

42 Chan R, Wong VW-S, Chu WC-W, *et al.* Diet-Quality Scores and Prevalence of Nonalcoholic Fatty Liver Disease: A Population Study Using Proton-Magnetic Resonance Spectroscopy. *PLoS One* 2015;**10**:1–14. doi:10.1371/journal.pone.0139310

43 Fung TT, Pan AA, Hou T, *et al.* Long-Term Change in Diet Quality Is Associated with Body Weight Change in Men and Women. *J Nutr* 2015;**145**:1850–6. doi:10.3945/jn.114.208785

44 Freedman ND, Cross AJ, McGlynn KA, *et al.* Association of meat and fat intake with liver disease and hepatocellular carcinoma in the NIH-AARP cohort. *J Natl Cancer Inst* 2010;**102**:1354–65. doi:10.1093/jnci/djq301

45 Georgoulis M, Kontogianni MD, Yiannakouris N. Mediterranean Diet and Diabetes: Prevention and Treatment. *Nutrients* 2014;**6**:1406–23. doi:10.3390/nu6041406

46 Georgoulis M, Kontogianni MD, Tileli N, *et al.* The impact of cereal grain consumption on the development and severity of non-alcoholic fatty liver disease. *Eur J Nutr* 2014;**53**:1727–35. doi:10.1007/s00394-014-0679-y

47 Abenavoli L, Milic N, Di Renzo L, *et al.* Metabolic aspects of adult patients with nonalcoholic fatty liver disease. *World J Gastroenterol* 2016;**22**:7006–16. doi:10.3748/wjg.v22.i31.7006

48 Salomone F, Godos J, Zelber-Sagi S. Natural antioxidants for non-alcoholic fatty liver disease: molecular targets and clinical perspectives. *LIVER Int* 2016;**36**:5–20. doi:10.1111/liv.12975

49 Abenavoli L, Milic N, Luzza F, *et al.* Polyphenols Treatment in Patients with Nonalcoholic

For peer review only

For peer review only - http://bmjopen.bmj.com/site/about/guidelines.xhtml

Fatty Liver Disease. *J Transl Intern Med* 2017;**5**:144–7. doi:10.1515/jtim-2017-0027

50 Yang J, Fernández-Galilea M, Martínez-Fernández L, *et al.* Oxidative Stress and Non-Alcoholic Fatty Liver Disease: Effects of Omega-3 Fatty Acid Supplementation. *Nutrients* 2019;**11**. doi:10.3390/nu11040872

51 Salomone F, Ivancovsky-Wajcman D, Fliss-Isakov N, *et al.* Higher phenolic acid intake independently associates with lower prevalence of insulin resistance and non-alcoholic fatty liver disease. *JHEP Reports* 2020;**2**:100069. doi:https://doi.org/10.1016/j.jhepr.2020.100069

52 Zelber-Sagi S, Salomone F, Mlynarsky L. The Mediterranean dietary pattern as the diet of choice for non-alcoholic fatty liver disease: Evidence and plausible mechanisms. *Liver Int* 2017;**37**:936–49. doi:10.1111/liv.13435

53 Mahady SE, George J. Exercise and diet in the management of nonalcoholic fatty liver disease. *Metab Exp* 2016;**65**:1172–82. doi:10.1016/j.metabol.2015.10.032

54 Parker HM, Johnson NA, Burdon CA, *et al.* Omega-3 supplementation and non-alcoholic fatty liver disease: A systematic review and meta-analysis. *J Hepatol* 2012;**56**:944–51. doi:10.1016/j.jhep.2011.08.018

55 Hernandez EA, Kahl S, Seelig A, *et al.* Acute dietary fat intake initiates alterations in energy metabolism and insulin resistance. *J Clin Invest* 2017;**127**:695–708.http://ovidsp.ovid.com/athens/ovidweb.cgi?T=JS&CSC=Y&NEWS=N&PAGE=fulltext&D=emex&AN=614623041

56 Bjermo H, Iggman D, Kullberg J, *et al.* Effects of n-6 PUFAs compared with SFAs on liver fat, lipoproteins, and inflammation in abdominal obesity: a randomized controlled trial. *Am J Clin Nutr* 2012;**95**:1003–12. doi:10.3945/ajcn.111.030114

57 Hu FB, Stampfer MJ, Rimm E, *et al.* Dietary fat and coronary heart disease: a comparison of approaches for adjusting for total energy intake and modeling repeated dietary measurements. *Am J Epidemiol* 1999;**149**:531–40.

58 Koehler EM, Schouten JNL, Hansen BE, *et al.* External Validation of the Fatty Liver Index for Identifying Nonalcoholic Fatty Liver Disease in a Population-based Study. *Clin Gastroenterol Hepatol* 2013;**11**:1201–4. doi:10.1016/j.cgh.2012.12.031

For peer review only

For peer review only - http://bmjopen.bmj.com/site/about/guidelines.xhtml

59 Machado M V, Cortez-Pinto H. Non-invasive diagnosis of non-alcoholic fatty liver disease. A critical appraisal. *J Hepatol* 2013;**58**:1007–19. doi:10.1016/j.jhep.2012.11.021

60 Zelber-Sagi S, Webb M, Assy N, *et al.* Comparison of fatty liver index with noninvasive methods for steatosis detection and quantification. *World J Gastroenterol* 2013;**19**:57–64. doi:10.3748/wjg.v19.i1.57

61 Fedchuk L, Nascimbeni F, Pais R, *et al.* Performance and limitations of steatosis biomarkers in patients with nonalcoholic fatty liver disease. *Aliment Pharmacol Ther* 2014;**40**:1209–22. doi:10.1111/apt.12963

For peer review only

For peer review only - http://bmjopen.bmj.com/site/about/guidelines.xhtml

Table 1 Baseline characteristics of participants according to quintiles of the Mediterranean diet score, CoLaus study, Switzerland (n=2,288).

	Quintiles of Mediterranean diet score*					
Characteristic	Q1 (n=458)	Q2 (n=458)	Q3 (n=457)	Q4 (n=458)	Q5 (n=457)	*P*-value*
Age (years)	57.1±10.6	56.7±9.9	56.4±10.5	55±9.8	53.7±8.9	<0.001
Women (%)	59.0	62.9	67.6	66.6	71.1	0.001
Marital status (%)						0.51
Single	17.7	14.2	16.8	17.5	16.4	
Married/cohabitant	53.9	55.7	59.1	57.2	57.3	
Widowed/separated/divorced	28.4	30.1	24.1	25.3	26.3	
Employed (%)	57.9	60.3	59.1	68.3	72.0	<0.001
Education (%)						
University	19.2	21.6	26.0	31.7	31.1	
High school	26.4	28.2	28.4	29.7	29.5	
Apprenticeship	39.3	35.2	34.8	28.2	28.4	
Mandatory education	15.1	15.1	10.7	10.5	10.9	<0.001
Current smokers (%)	24.7	21.8	22.3	16.2	15.5	0.014
Alcohol intake (%)†						
Abstainers	21.8	24.0	25.8	24.7	21.2	
Moderate	63.8	66.2	69.4	70.7	76.6	
Heavy	14.4	9.8	4.8	4.6	2.2	<0.001
Total energy intake (kcal/day)	1819±705	1812±675	1781±729	1821±653	1801±595	0.87
Protein (%energy)	15.8±3.3	15.9±4.1	15.3±3.0	15.2±3.0	14.7±2.6	<0.001
Carbohydrates (%energy)	45.3±9.2	45.9±9.1	47.3±8.0	47.6±8.2	49.0±7.8	<0.001
Fat (%energy)	33.6±6.5	34.5±6.9	34.6±6.7	34.5±6.5	34.1±6.9	0.11
TEE (kcal/day)	2562±589	2600±618	2564±602	2558±555	2589±565	0.84
Metabolic syndrome (%)‡	11.8	9.8	12.3	12.7	7.4	0.063

For peer review only - http://bmjopen.bmj.com/site/about/guidelines.xhtml

BMI (kg/m^2)	24.0±2.7	23.8±2.9	23.9±3.0	23.6±2.8	23.3±2.7	0.003
Waist circumference (cm)	85.4±8.5	84.9±9.4	85.0±9.2	84.0±8.7	82.9±8.6	<0.001
Triglycerides (mmol/l)	1.1±0.5	1.0±0.5	1.1±0.5	1.0±0.5	1.0±0.5	
median (iqr)	1.0 (0.8, 1.3)	0.9 (0.7, 1.2)	1.0 (0.7, 1.3)	0.9 (0.7, 1.2)	0.9 (0.7, 1.2)	0.009
GGT (U/l)	25.0±16.5	25.1±17.9	23.2±14.5	24.0±17.9	21.5±12.1	0.002
median (iqr)	20 (15, 29)	21 (15, 28)	19 (14, 28)	19 (14, 26)	18 (14, 25)	
≥50 (%)	7.4	6.3	5.7	6.1	2.6	0.025
ALT (U/l)	21.3±7.8	22.1±8.9	22.5±9.7	21.3±8.7	21.5±8.7	0.12
median (iqr)	19 (16, 25)	20 (16, 26)	20 (16, 27)	19 (16, 25)	20 (16, 24)	
≥40 (%)	3.5	5.4	8.3	4.6	3.5	0.005
AST (U/l)	26.0±5.85	26.2±6.5	26.1±6.6	25.8±6.1	25.7±5.7	0.68
median (iqr)	25 (22, 29)	25 (22, 29)	25 (21, 29)	25 (22, 29)	25 (22, 29)	
≥37 (%)	4.1	8.3	6.3	5.0	4.4	0.038

Abbreviations: TEE, total energy expenditure; BMI, body mass index; iqr, interquartile range; GGT, gamma-glutamyl transferase; ALT, alanine aminotransferase; AST, aspartate aminotransferase.

* The population was divided into five groups by quintiles (Q1-Q5) of the Mediterranean diet score. P-values were computed by using ANOVA for continuous variables and Chi-square test for categorical variables.

† Alcohol consumption categorized as "abstainers" (0 unit/week), "moderate" (1–21 units/week for men, 1–14 for women), and "heavy drinkers" (>21 units/week for men, >14 for women).

‡ Metabolic syndrome defined according to the International Diabetic Federation (waist circumference ≥94 cm in men and ≥80 cm in women plus at least two of the following factors: serum triglycerides≥1.70 mmol/L or specific treatment for this lipid abnormality; serum high-density lipoprotein cholesterol <1.03 mmol/L in men and <1.29 mmol/L in women or specific treatment for this lipid abnormality; systolic blood pressure ≥130 mm Hg or diastolic blood pressure ≥85 mm Hg or treatment for previously diagnosed hypertension; and fasting plasma glucose ≥5.6 mmol/L or previously diagnosed type 2 diabetes).

For peer review only - http://bmjopen.bmj.com/site/about/guidelines.xhtml

Table 2 Prospective association of the Mediterranean diet score with the risk of hepatic steatosis, CoLaus study, Switzerland (n=2,288).

	Risk ratio (95% CI) across quintiles of Mediterranean diet score*					*P*-trend	Risk ratio (95% CI) Per SD increase*
	Q1	Q2	Q3	Q4	Q5		
range	1.83-7.63	7.64-8.35	8.36-8.92	8.93-9.59	9.60-12.1		
N total	458	458	457	458	457		
Fatty liver index, median (iqr)†	21.8 (10.4, 37.7)	20.4 (9.0, 39.4)	18.5 (8.9, 34.6)	18.1 (7.7, 33.1)	14.2 (6.6, 28.5)		
N cases (score≥60)	36	43	35	22	17		
Unadjusted	1.00 (ref.)	1.19 (0.78, 1.82)	0.97 (0.62, 1.52)	0.61 (0.37, 1.02)	0.47 (0.27, 0.83)	0.001	0.79 (0.70, 0.90)
Multivariable ‡	1.00 (ref.)	1.11 (0.72, 1.72)	1.07 (0.68, 1.66)	0.71 (0.42, 1.22)	0.50 (0.28, 0.91)	0.006	0.84 (0.73, 0.96)
Multivariable + BMI	1.00 (ref.)	1.12 (0.73, 1.71)	0.90 (0.58, 1.39)	0.74 (0.44, 1.24)	0.61 (0.34, 1.09)	0.031	0.85 (0.73, 0.99)
Multivariable + BMI + WC	1.00 (ref.)	1.07 (0.70, 1.64)	0.90 (0.59, 1.37)	0.74 (0.44, 1.26)	0.60 (0.34, 1.08)	0.034	0.85 (0.71, 1.02)
NAFLD-score, median (iqr)§	-2.1 (-2.4, -1.5)	-2.0 (-2.4, -1.4)	-2.0 (-2.4, -1.3)	-2.1 (-2.5, -1.5)	-2.1 (-2.5, -1.6)		
N cases (score≥-0.640)	41	46	51	38	32		
Unadjusted	1.00 (ref.)	1.12 (0.75, 1.67)	1.25 (0.84, 1.84)	0.93 (0.61, 1.41)	0.78 (0.50, 1.22)	0.17	0.93 (0.82, 1.05)
Multivariable ‡	1.00 (ref.)	1.13 (0.75, 1.70)	1.26 (0.85, 1.87)	1.01 (0.65, 1.56)	0.80 (0.50, 1.28)	0.28	0.95 (0.83, 1.09)
Multivariable + BMI	1.00 (ref.)	1.17 (0.78, 1.75)	1.17 (0.80, 1.71)	1.07 (0.69, 1.65)	0.95 (0.60, 1.52)	0.71	0.99 (0.86, 1.15)
Multivariable + BMI + WC	1.00 (ref.)	1.12 (0.75, 1.67)	1.17 (0.80, 1.72)	1.07 (0.70, 1.65)	0.96 (0.60, 1.53)	0.80	1.00 (0.86, 1.17)

Abbreviations: iqr, interquartile range; BMI, body mass index; WC, waist circumference; NAFLD, non-alcoholic fatty liver disease.

For peer review only - http://bmjopen.bmj.com/site/about/guidelines.xhtml

Statistical analysis using Poisson regression with robust standard errors; results are expressed as risk ratios and (95% confidence intervals).

* In categorical analysis, the population was divided into five groups by quintiles (Q1-Q5) of the Mediterranean diet score; Standard deviation was 1.20 for the multivariable analyses.

† Calculated based on an algorithm including BMI, waist circumference, triglycerides, and gamma-glutamyl transferase.

‡ Adjusted for age (years), sex, marital status (single, married/cohabiting, and widowed/Separated/divorced), occupational status (working and not working), education level (university, high school, apprenticeship, and mandatory education), smoking status (never, former, and current), energy intake (kcal/day), total energy expenditure (kcal/day), and date of dietary assessment.

§ Calculated based on an algorithm including presence of the metabolic syndrome and type 2 diabetes, and concentrations of fasting serum insulin, fasting serum aspartate-aminotransferase (AST), and the AST/alanine-aminotransferase ratio.

For peer review only

For peer review only - http://bmjopen.bmj.com/site/about/guidelines.xhtml

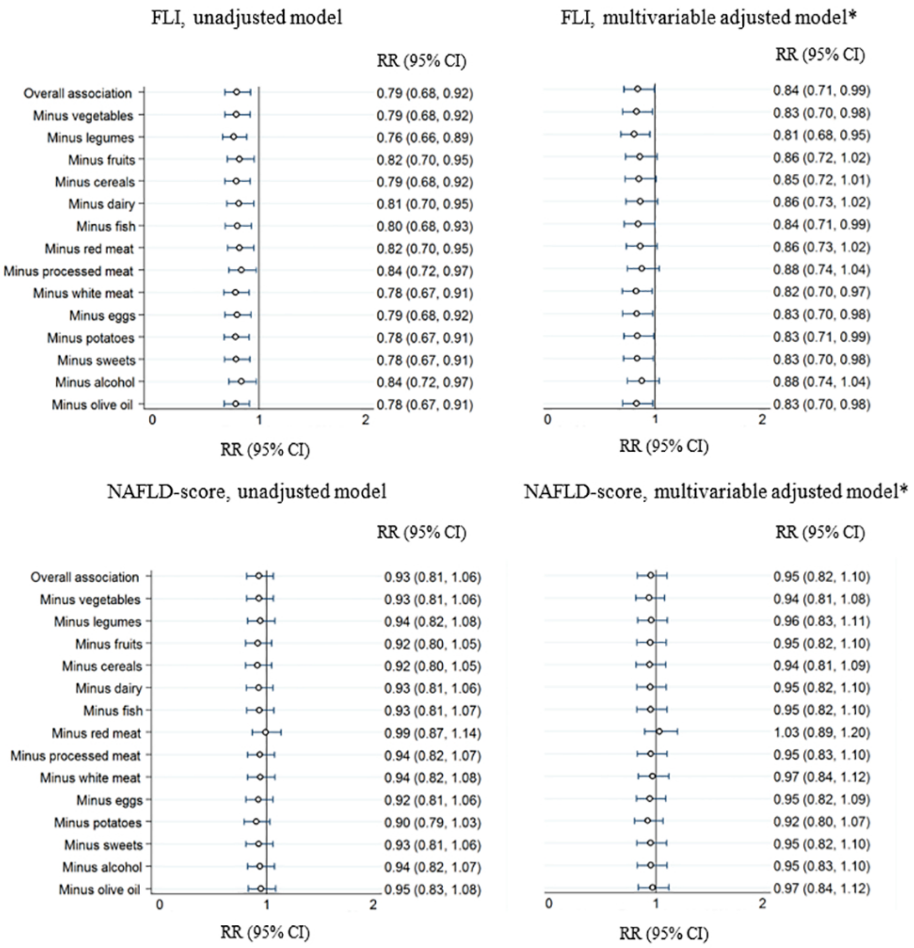

Figure 1 Prospective association of the Mediterranean diet score with the risk of hepatic steatosis, CoLaus study, Switzerland (n=2288): sensitivity analysis to examine influence of each component of Mediterranean diet.

Abbreviations: FLI, fatty liver index; NAFLD, non-alcoholic fatty liver disease.
Statistical analysis using Poisson regression with robust standard errors; results are expressed as risk ratios and (95% confidence intervals).
Risk ratio (RR) and 95% confidence intervals (CI) were estimated per one standard deviation of MDS (overall association) or of each MDS computed after excluding one component.
* Adjusted for age (years), sex, marital status (single, married/cohabiting, and widowed/Separated/divorced), occupational status (working and not working), education level (university, high school, apprenticeship, and mandatory education), smoking status (never, former, and current), energy intake (kcal/day), total energy expenditure (kcal/day), and date of dietary assessment.

Supplementary file

Title: The association between adherence to the Mediterranean diet and hepatic steatosis: the Swiss CoLaus prospective cohort study

For peer review only

Table of Content

Figure S1 Sample selection flow chart. ... 3

Table S1 Characteristics of the participants included and excluded from the analysis, CoLaus study, Switzerland. ... 4

Table S2 Baseline characteristics of the participants according to the risk of hepatic steatosis defined by the fatty liver index or the NAFLD-score, CoLaus study, Switzerland. ... 5

Table S3 Prospective association of the Mediterranean diet score with the risk of hepatic steatosis, CoLaus study, Switzerland: assessment of influence of other potential covariates. ... 6

Table S4 Sensitivity analyses for the associations of the Mediterranean diet score with the risk of hepatic steatosis, CoLaus study, Switzerland. ... 8

Table S5 Prospective association of the Mediterranean diet score with the risk of hepatic steatosis, CoLaus study, Switzerland: sensitivity analysis while excluding participants with only one of the indices high at baseline and using hepatic steatosis index as an alternative score. ... 11

Table S6 Prospective association of the Mediterranean diet score with the risk of hepatic steatosis, CoLaus study, Switzerland (n=1632), Sensitivity analysis while excluding participants with either indices high (using FLI>30 instead of FLI≥60). ... 13

Table S7 Association of the Mediterranean diet score with change in BMI and waist circumference, CoLaus study, Switzerland. ... 14

Table S8 Association of the Mediterranean diet score with the GGT, ALT, and AST, CoLaus study, Switzerland. ... 15

For peer review only

Figure S1 Sample selection flow chart.

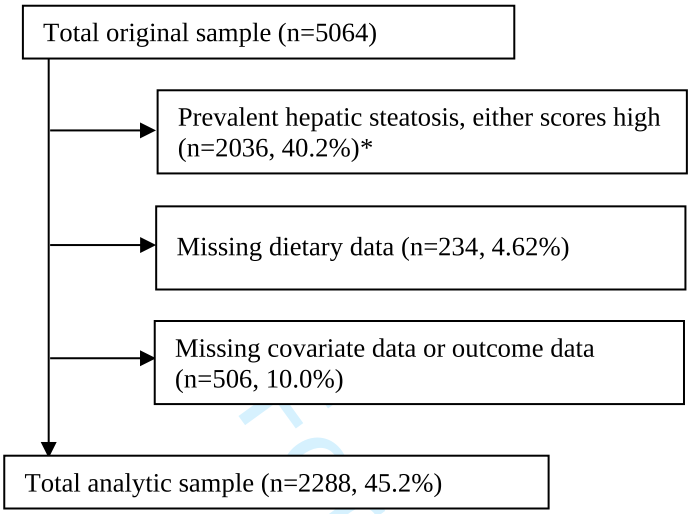

* Prevalent hepatic steatosis was defined as having either fatty liver index≥ 60 OR non-alcoholic fatty liver disease fatty liver score≥ -0.640.

For peer review only

Table S1 Characteristics of the participants included and excluded from the analysis, CoLaus study, Switzerland.

Characteristic	Included (n=2288)	Excluded (n=2776)	*P*-value*
Age, *years*	55.8±10.0	59.4±10.6	<0.001
Women (%)	65.4	43.6	<0.001
Marital status (%)†			0.038
Single	16.5	14.0	
Married/cohabiting	56.6	57.4	
Widowed/separated/divorced	26.8	28.6	
Employed (%)	63.5	50.4	<0.001
Education (%)†			<0.001
University	25.9	17.5	
High school	28.5	23.6	
Apprenticeship	33.2	37.4	
Mandatory education	12.5	21.4	
Current smoker (%)	20.1	23.1	<0.001
Alcohol consumption (%)†‡			<0.001
Abstainers	23.5	26.8	
Moderate	69.3	63.3	
Heavy	7.2	9.9	
Total energy intake (kcal/d)	1807±673	1853±784	0.033
Total protein (% energy)	15.4±3.3	15.6±3.5	0.015
Total carbohydrate (% energy)	47.0±8.6	45.4±9.1	<0.001
Total fat (% energy)	34.3±6.7	34.4±6.9	0.54
TEE (kcal/d)	2575±586	2790±669	<0.001
Metabolic syndrome (%)§	10.8	60.9	<0.001
BMI (kg/m^2)	23.7±2.8	28.3±4.8	<0.001
Waist circumference (cm)	84.4±8.9	98.3±12.5	<0.001
Triglycerides (mmol/l)	1.0±0.5	1.6±1.1	<0.001
median (iqr)	0.9 (0.7, 1.2)	1.4 (1.0, 2.0)	
GGT (U/l)	23.8±16.0	48.8±60.8	<0.001
median (iqr)	19 (14, 27)	32 (21, 53)	
≥50 (%)	5.6	27.5	<0.001
ALT (U/l)	21.8±8.8	32.5±20.7	<0.001
median (iqr)	20 (16, 25)	27 (20, 38)	
≥40 (%)	5.0	23.3	<0.001
AST (U/l)	25.9±6.2	31.6±14.1	<0.001
median (iqr)	25 (22, 29)	28 (24, 34)	
≥37 (%)	5.6	20.4	<0.001

Abbreviations: TEE, total energy expenditure; BMI, body mass index; iqr, interquartile range; GGT, gamma-glutamyl transferase; ALT, alanine aminotransferase; AST, aspartate aminotransferase.
Data are mean ± SD for continuous variables or percent for categorical variables, unless otherwise stated.
* P-value calculated using Chi-square test for categorical variables and student's t-test for continuous variables.
† Due to some missing data, percentages do not always add to 100%.
‡ Alcohol consumption categorized as "abstainers" (0 unit/week), "moderate" (1–21 units/week for men, 1–14 for women), and "heavy drinkers" (>21 units/week for men, >14 for women).
§ Metabolic syndrome defined according to the International Diabetic Federation (waist circumference ≥94 cm in men and ≥80 cm in women plus at least two of the following factors: serum triglycerides≥1.70 mmol/L or specific treatment for this lipid abnormality; serum high-density lipoprotein cholesterol <1.03 mmol/L in men and <1.29 mmol/L in women or specific treatment for this lipid abnormality; systolic blood pressure ≥130 mmHg or diastolic blood pressure ≥85 mmHg or treatment for previously diagnosed hypertension; and fasting plasma glucose ≥5.6 mmol/L or previously diagnosed type 2 diabetes).

For peer review only - http://bmjopen.bmj.com/site/about/guidelines.xhtml

Table S2 Baseline characteristics of the participants according to the risk of hepatic steatosis defined by the fatty liver index or the NAFLD-score, CoLaus study, Switzerland.

	Risk of hepatic steatosis					
	FLI			NAFLD-score		
Characteristic	No (n=2135)	Yes (n=153)	*P*-value*	No (n=2080)	Yes (n=208)	*P*-value*
Age, *years*	55.8±10.1	54.9±9.5	0.29	55.6±10	57.7±10.1	0.003
Women (%)	66.8	45.8	<0.001	66.7	52.9	<0.001
Marital status (%)†			0.86			0.79
Single	16.6	15.0		16.7	14.9	
Married/cohabiting	56.5	58.2		56.5	58.2	
Widowed/Separated/divorced	26.8	26.8		26.8	26.9	
Employed (%)	63.3	66.0	0.50	64.5	53.8	0.002
Education (%)†			0.013			0.33
University	26.7	15.0		26.4	20.7	
High school	28.3	30.1		28.3	29.8	
Apprenticeship	32.6	40.5		32.8	36.5	
Mandatory education	12.3	14.4		12.4	13.0	
Current smoker (%)	19.7	25.5	0.006	20.3	17.8	0.52
Alcohol consumption (%)†‡			0.15			0.001
Abstainers	23.3	26.1		22.5	33.7	
Moderate	69.7	63.4		70.4	58.7	
Heavy	6.9	10.5		7.1	7.7	
Total energy intake (kcal/d)	1800±655	1906±879	0.062	1794±654	1938±833	0.003
Protein (% energy)	15.3±3.1	16.1±5.0	0.006	15.3±3.2	16.0±4.1	0.006
Carbohydrate (% energy)	47.1±8.5	45.6±10.1	0.037	47.0±8.5	46.5±9.5	0.36
Fat (% energy)	34.3±6.7	33.8±7.2	0.37	34.2±6.7	34.4±6.6	0.69
TEE (kcal/d)	2552±572	2912±677	<0.001	2562±582	2699±616	0.002
Metabolic syndrome (%)§	9.9	22.9	<0.001	9.8	21.2	<0.001
BMI (kg/m^2)	23.5±2.7	26.8±2.6	<0.001	23.5±2.8	26.0±2.5	<0.001
Waist circumference (cm)	83.8±8.7	93.4±7.0	<0.001	83.7±8.7	92.0±7.4	<0.001
Triglycerides (mmol/l)	1.0±0.5	1.2±0.5	<0.001	1.0±0.5	1.2±0.5	<0.001
median (iqr)	0.9 (0.7, 1.2)	1.1 (0.7, 1.2)		0.9 (0.7, 1.2)	1.1 (0.8, 1.4)	
GGT (U/l)	23.1±15.2	32.4±22.2	<0.001	23.3±15.4	28.1±20.6	<0.001
median (iqr)	19 (14, 26)	26 (18, 40)		19 (14, 26)	22 (15, 34)	
≥50 (%)	4.9	15.0	<0.001	5.3	9.1	0.022
ALT (U/l)	21.6±8.8	23.5±8.9	0.013	21.3±8.4	26.0±11.6	<0.001
median (iqr)	19 (16, 25)	21 (17, 28)		19 (16, 25)	23 (18, 30)	
≥40 (%)	5.0	5.9	0.63	4.5	10.6	<0.001
AST (U/l)	25.9±6.2	26.3±6.3	0.50	25.9±6.2	26.9±6.2	0.026
median (iqr)	25 (22, 29)	25 (22, 29)		25 (22, 29)	26 (22, 30)	
≥37 (%)	5.5	7.2	0.39	5.3	8.2	0.096

Abbreviations: TEE, total energy expenditure; FLI, fatty liver index; NAFLD, non-alcoholic fatty liver disease; BMI, body mass index; iqr, interquartile range; GGT, gamma-glutamyl transferase; ALT, alanine aminotransferase; AST, aspartate Aminotransferase.

Data are mean ± SD for continuous variables or percent for categorical variables, unless otherwise stated.

* P-value calculated using Chi-square test for categorical variables and student’s t-test for continuous variables.

† Due to some missing data, percentages do not always add to 100%.

‡ Alcohol consumption categorized as “abstainers” (0 unit/week), “moderate” (1–21 units/week for men, 1–14 for women), and “heavy drinkers” (>21 units/week for men, >14 for women).

§ Metabolic syndrome defined according to the International Diabetic Federation (waist circumference ≥94 cm in men and ≥80 cm in women plus at least two of the following factors: serum triglycerides≥1.70 mmol/L or specific treatment for this lipid abnormality; serum high-density lipoprotein cholesterol <1.03 mmol/L in men and <1.29 mmol/L in women or specific treatment for this lipid abnormality; systolic blood pressure ≥130 mm Hg or diastolic blood pressure ≥85 mm Hg or treatment for previously diagnosed hypertension; and fasting plasma glucose ≥5.6 mmol/L or previously diagnosed type 2 diabetes.

For peer review only - http://bmjopen.bmj.com/site/about/guidelines.xhtml

Table S3 Prospective association of the Mediterranean diet score with the risk of hepatic steatosis, CoLaus study, Switzerland: assessment of influence of other potential covariates.

	Risk ratio (95% CI) across quintiles of Mediterranean diet score*					*P*-trend	Risk ratio (95% CI) Per SD increase*
	Q1	Q2	Q3	Q4	Q5		
Range	1.83-7.63	7.64-8.35	8.36-8.92	8.93-9.59	9.60-12.1		
N total	458	458	457	458	457		
Fatty liver index†							
Different models							
N Cases (score≥60)	36	43	35	22	17		
Multivariable (Model 1)‡	1.00 (ref.)	1.11 (0.72, 1.72)	1.07 (0.68, 1.66)	0.71 (0.42, 1.22)	0.50 (0.28, 0.91)	0.006	0.84 (0.71, 0.99)
Model 1 + Changes in BMI categories§	1.00 (ref.)	1.11 (0.73, 1.69)	0.98 (0.64, 1.50)	0.73 (0.43, 1.24)	0.59 (0.32, 1.06)	0.024	0.85 (0.72, 1.02)
Model 1 + Alcohol	1.00 (ref.)	1.11 (0.72, 1.72)	1.06 (0.69, 1.65)	0.71 (0.41, 1.22)	0.50 (0.28, 0.91)	0.006	0.84 (0.70, 0.99)
Model 1 + Alcohol+ BMI‖	1.00 (ref.)	1.13 (0.74, 1.73)	0.92 (0.59, 1.42)	0.76 (0.45, 1.29)	0.63 (0.35, 1.14)	0.047	0.86 (0.72, 1.04)
Model 1 + Clinical variables**	1.00 (ref.)	1.30 (0.80, 2.11)	1.20 (0.74, 1.94)	0.77 (0.43, 1.39)	0.57 (0.30, 1.09)	0.023	0.84 (0.70, 1.01)
Model 1 + Clinical variables + BMI	1.00 (ref.)	1.28 (0.79, 2.07)	0.94 (0.58, 1.52)	0.80 (0.45, 1.41)	0.68 (0.36, 1.27)	0.076	0.86 (0.71, 1.04)
Model 1 + MetS††	1.00 (ref.)	1.14 (0.74, 1.75)	1.03 (0.66, 1.60)	0.68 (0.40, 1.16)	0.51 (0.28, 0.93)	0.005	0.83 (0.70, 0.99)
NAFLD-score‡‡							
Different models							
N Cases (score≥-0.640)	41	46	51	38	32		
Multivariable (Model 1)‡	1.00 (ref.)	1.13 (0.75, 1.70)	1.26 (0.85, 1.87)	1.01 (0.65, 1.56)	0.80 (0.50, 1.28)	0.28	0.95 (0.82, 1.10)
Model 1 + Changes in BMI categories§	1.00 (ref.)	1.13 (0.76, 1.69)	1.18 (0.80, 1.74)	1.04 (0.68, 1.60)	0.93 (0.58, 1.48)	0.65	0.99 (0.86, 1.16)
Model 1 + Alcohol	1.00 (ref.)	1.11 (0.73, 1.67)	1.22 (0.82, 1.82)	0.97 (0.63, 1.51)	0.77 (0.48, 1.23)	0.21	0.94 (0.81, 1.09)
Model 1 + Alcohol+ BMI‖	1.00 (ref.)	1.16 (0.78, 1.73)	1.15 (0.78, 1.68)	1.04 (0.68, 1.61)	0.93 (0.58, 1.48)	0.62	0.99 (0.85, 1.15)
Model 1 + Clinical variables**	1.00 (ref.)	1.20 (0.78, 1.84)	1.32 (0.88, 1.98)	0.99 (0.64, 1.55)	0.85 (0.52, 1.37)	0.33	0.95 (0.82, 1.11)
Model 1 + Clinical variables + BMI	1.00 (ref.)	1.21 (0.80, 1.84)	1.19 (0.80, 1.78)	1.06 (0.69, 1.64)	0.99 (0.61, 1.60)	0.76	0.99 (0.85, 1.16)

Abbreviations: SES, socio-economic status; BMI, body mass index; MetS, metabolic syndrome; NAFLD, non-alcoholic fatty liver disease; SD, standard deviation; CI, confidence interval.

Statistical analysis using Poisson regression with robust standard errors; results are expressed as risk ratios and (95% confidence intervals).

* In categorical analysis, the population was divided into five groups by quintiles (Q1-Q5) of the Mediterranean diet score; Standard deviation was 1.20 for different multivariable analyses.

† Calculated based on an algorithm including BMI, waist circumference, triglycerides, and gamma-glutamyl transferase.

‡ Adjusted for age (years), sex, marital status (single, married/cohabitant, and widowed/Separated/divorced), occupational status (working and not working), education level (university, high school, apprenticeship, and mandatory education), smoking status (never, former, and current), energy intake (kcal/day), total energy expenditure (kcal/day), and date of dietary assessment.

§ Changes in BMI categories defined as individuals with a normal BMI at baseline and follow-up, individuals with normal BMI at baseline and overweight or obese BMI at follow-up, individuals with overweight or obese BMI at baseline and at follow-up, or individuals with overweight or obese BMI at baseline and normal at follow-up.

‖ Results of further adjustment for waist circumference were broadly in line with of the further adjustment for BMI (data not shown).

**Family history of diabetes (yes/no), high blood pressure (yes/no), high triglyceride level (yes/no), low HDL level (yes/no), and high glucose level (yes/no).

†† Metabolic syndrome as defined by the International Diabetes Federation (yes/no).

For peer review only - http://bmjopen.bmj.com/site/about/guidelines.xhtml

‡‡ Calculated based on an algorithm including presence of the metabolic syndrome and type 2 diabetes, and concentrations of fasting serum insulin, fasting serum aspartate-aminotransferase (AST), and the AST/alanine-aminotransferase ratio.

For peer review only

Table S4 Sensitivity analyses for the associations of the Mediterranean diet score with the risk of hepatic steatosis, CoLaus study, Switzerland.

	Risk ratio (95% CI) across quintiles of Mediterranean diet score*						
	Q1	Q2	Q3	Q4	Q5	*P*-trend	Risk ratio (95% CI) Per SD increase*
Range	1.83-7.63	7.64-8.35	8.36-8.92	8.93-9.59	9.60-12.1		
N total	458	458	457	458	457		
Fatty liver index†							
Different models							
N Cases (score≥60)	36	43	35	22	17		
Multivariable (Model 1)‡	1.00 (ref.)	1.11 (0.72, 1.72)	1.07 (0.68, 1.66)	0.71 (0.42, 1.22)	0.50 (0.28, 0.91)	0.006	0.84 (0.71, 0.99)
Excluding alcohol from MDS component							
Model 1 + Alcohol	1.00 (ref.)	1.64 (1.04, 2.58)	1.08 (0.65, 1.80)	1.17 (0.70, 1.96)	0.65 (0.35, 1.18)	0.069	0.86 (0.73, 1.03)
Model 1 + Alcohol + BMI§	1.00 (ref.)	1.53 (0.98, 2.39)	0.94 (0.57, 1.54)	1.08 (0.67, 1.77)	0.75 (0.41, 1.35)	0.15	0.89 (0.75, 1.06)
Excluding participants with BMI≥30‖							
N Cases (score≥60)	35	40	28	19	16		
Model 1	1.00 (ref.)	1.08 (0.69, 1.68)	0.90 (0.56, 1.46)	0.64 (0.36, 1.12)	0.50 (0.27, 0.93)	0.005	0.83 (0.69, 0.98)
Model 1 + BMI§	1.00 (ref.)	1.02 (0.66, 1.58)	0.84 (0.53, 1.33)	0.65 (0.37, 1.14)	0.60 (0.33, 1.10)	0.031	0.85 (0.70, 1.02)
Excluding participants with excessive alcohol consumption**							
N Cases (score≥60)	33	42	34	21	17		
Model 1	1.00 (ref.)	1.17 (0.75, 1.84)	1.12 (0.71, 1.77)	0.70 (0.40, 1.23)	0.52 (0.28, 0.95)	0.007	0.82 (0.69, 0.98)
Model 1 + BMI§	1.00 (ref.)	1.17 (0.76, 1.81)	0.91 (0.58, 1.42)	0.73 (0.42, 1.24)	0.64 (0.35, 1.15)	0.037	0.85 (0.71, 1.02)
Excluding participants with implausible energy intake††							
N Cases (score≥60)	33	41	33	22	16		
Model 1	1.00 (ref.)	1.19 (0.63, 2.23)	1.16 (0.61, 2.20)	0.87 (0.45, 1.69)	0.69 (0.35, 1.38)	0.16	0.85 (0.71, 1.01)
Model 1 + BMI§	1.00 (ref.)	1.13 (0.60, 2.13)	0.90 (0.47, 1.73)	0.81 (0.43, 1.53)	0.74 (0.37, 1.49)	0.20	0.86 (0.72, 1.04)
Excluding participants with secondary causes of hepatic steatosis‡‡							
N Cases (score≥60)	37	42	35	22	17		
Model 1	1.00 (ref.)	1.18 (0.75, 1.85)	1.11 (0.70, 1.76)	0.78 (0.45, 1.34)	0.51 (0.27, 0.94)	0.010	0.85 (0.71, 1.01)
Model 1 + BMI§	1.00 (ref.)	1.17 (0.75, 1.83)	0.95 (0.60, 1.50)	0.78 (0.46, 1.31)	0.63 (0.34, 1.17)	0.050	0.86 (0.72, 1.04)
Excluding participants with diabetes§§							
N Cases (score≥60)	35	40	35	22	16		
Model 1	1.00 (ref.)	1.06 (0.67, 1.66)	1.11 (0.71, 1.73)	0.74 (0.43, 1.27)	0.49 (0.26, 0.91)	0.009	0.84 (0.71, 0.998)
Model 1 + BMI§	1.00 (ref.)	1.06 (0.69, 1.65)	0.92 (0.59, 1.43)	0.76 (0.45, 1.29)	0.58 (0.32, 1.06)	0.033	0.85 (0.71, 1.02)
NAFLD-score ¶¶							
Different models							
N Cases (score≥-0.640)	41	46	51	38	32		
Multivariable (Model 1)‡	1.00 (ref.)	1.13 (0.75, 1.70)	1.26 (0.85, 1.87)	1.01 (0.65, 1.56)	0.80 (0.50, 1.28)	0.28	0.95 (0.82, 1.10)

For peer review only - http://bmjopen.bmj.com/site/about/guidelines.xhtml

Excluding alcohol from MD component							
Model 1 + Alcohol	1.00 (ref.)	1.15 (0.78, 1.71)	0.92 (0.61, 1.41)	0.92 (0.59, 1.42)	0.89 (0.57, 1.38)	0.34	0.93 (0.81, 1.08)
Model 1 + Alcohol + BMI§	1.00 (ref.)	1.15 (0.78, 1.69)	0.88 (0.59, 1.32)	0.88 (0.57, 1.35)	1.02 (0.66, 1.57)	0.65	0.96 (0.83, 1.12)
Excluding participants with BMI≥30‖							
N Cases (score≥-0.640)	40	46	44	36	31		
Model 1	1.00 (ref.)	1.15 (0.76, 1.74)	1.12 (0.74, 1.69)	0.99 (0.63, 1.55)	0.81 (0.5, 1.30)	0.27	0.94 (0.81, 1.09)
Model 1 + BMI§	1.00 (ref.)	1.17 (0.78, 1.75)	1.09 (0.73, 1.63)	1.07 (0.69, 1.67)	0.97 (0.6, 1.55)	0.76	0.99 (0.85, 1.15)
Excluding participants with excessive alcohol consumption**							
N Cases (score≥-0.640)	38	43	50	37	32		
Model 1	1.00 (ref.)	1.10 (0.73, 1.68)	1.28 (0.85, 1.91)	1.00 (0.64, 1.56)	0.80 (0.5, 1.29)	0.32	0.96 (0.83, 1.11)
Model 1 + BMI§	1.00 (ref.)	1.15 (0.76, 1.74)	1.18 (0.80, 1.73)	1.06 (0.69, 1.65)	0.96 (0.6, 1.54)	0.75	1.00 (0.86, 1.17)
Excluding participants with implausible energy intake††							
N Cases (score≥-0.640)	39	46	48	38	30		
Model 1	1.00 (ref.)	1.32 (0.75, 2.35)	1.48 (0.84, 2.60)	1.06 (0.59, 1.91)	1.15 (0.64, 2.07)	0.92	0.95 (0.82, 1.10)
Model 1 + BMI§	1.00 (ref.)	1.34 (0.76, 2.38)	1.35 (0.77, 2.37)	1.06 (0.59, 1.89)	1.29 (0.72, 2.33)	0.67	0.99 (0.85, 1.16)
Excluding participants with secondary causes of hepatic steatosis‡‡							
N Cases (score≥-0.640)	41	46	50	37	31		
Model 1	1.00 (ref.)	1.19 (0.79, 1.81)	1.26 (0.84, 1.89)	1.06 (0.68, 1.65)	0.78 (0.48, 1.27)	0.25	0.95 (0.82, 1.10)
Model 1 + BMI§	1.00 (ref.)	1.23 (0.82, 1.85)	1.17 (0.79, 1.73)	1.11 (0.71, 1.72)	0.93 (0.57, 1.52)	0.66	0.99 (0.85, 1.16)
Excluding participants with diabetes§§							
N Cases (score≥-0.640)	37	43	50	36	30		
Model 1	1.00 (ref.)	1.17 (0.76, 1.79)	1.37 (0.90, 2.06)	1.05 (0.67, 1.66)	0.85 (0.52, 1.39)	0.43	0.97 (0.83, 1.12)
Model 1 + BMI§	1.00 (ref.)	1.21 (0.79, 1.85)	1.26 (0.85, 1.87)	1.11 (0.71, 1.75)	1.01 (0.62, 1.63)	0.89	1.01 (0.86, 1.18)

Abbreviations: SES, socio economic status; BMI, body mass index; MD, Mediterranean Diet; NAFLD, non-alcoholic fatty liver disease; SD, standard deviation; CI, confidence interval.

Statistical analysis using Poisson regression with robust standard errors; results are expressed as risk ratios and (95% confidence intervals).

* In categorical analysis, the population was divided into five groups by quintiles (Q1-Q5) of the Mediterranean diet score; Standard deviation was 1.41 after excluding alcohol from Mediterranean Diet score components, 1.20 after excluding participants with BMI≥30, 1.18 after excluding participants with excessive alcohol consumption, 1.20 after excluding participants with probable implausible energy intake or after excluding participants with secondary causes of hepatic steatosis or diabetes.

† Calculated based on an algorithm including BMI, waist circumference, triglycerides, and gamma-glutamyl transferase.

‡ Adjusted for age (years), sex, marital status (single, married/cohabitant, and widowed/Separated/divorced), occupational status (working and not working), education level (university, high school, apprenticeship, and mandatory education), smoking status (never, former, and current), energy intake (kcal/day), total energy expenditure (kcal/day), and date of dietary assessment.

§ Results of further adjustment for waist circumference were broadly in line with of the further adjustment for BMI (data not shown).

‖ Excluded 36 participants with BMI≥30 kg/m^2.

** Excessive alcohol consumption defined as >21 units per week for men and >14 units per weeks for women; excluded 60 participants with excess alcohol consumption (n=2228).

†† Implausible energy intake defined as <500 and <800 kcal or >3,500 or >4,000 kcal in women and men, respectively; excluded 41 participants with probable implausible energy intake (n=2247).

For peer review only - http://bmjopen.bmj.com/site/about/guidelines.xhtml

‡‡ Secondary causes of hepatic steatosis defined as having hepatitis B, C or HIV, and with hepatotoxic medications (Glucocorticoids, isoniazid, methotrexate, amiodarone, and tamoxifen); excluded 36 participants with probable secondary causes of hepatic steatosis (n=2252).
§§ Diabetes defined as glycated haemoglobin≥48 mmol/mol, or fasting plasma glucose ≥7.0 mmol/L, or use of hypoglycaemic drugs or insulin; excluded 26 participants with probable secondary causes of hepatic steatosis (n=2262).
¶¶ Calculated based on an algorithm including presence of the metabolic syndrome and type 2 diabetes, and concentrations of fasting serum insulin, fasting serum aspartate-aminotransferase (AST), and the AST/alanine-aminotransferase ratio.

For peer review only

Table S5 Prospective association of the Mediterranean diet score with the risk of hepatic steatosis, CoLaus study, Switzerland: sensitivity analysis while excluding participants with only one of the indices high at baseline and using hepatic steatosis index as an alternative score.

	Risk ratio (95% CI) across quintiles of Mediterranean diet score*					*P*-trend	Risk ratio (95% CI) Per SD increase *
	Q1	Q2	Q3	Q4	Q5		
range	1.83-7.64	7.64-8.34	8.35-8.92	8.93-9.57	9.58-12.18		
N total (n=2652)	531	530	531	530	530		
Fatty liver index, *median* (iqr)†	23.4 (11.1, 41.4)	24.1 (10.6, 44.7)	22.3 (9.9, 39.7)	20.2 (8.4, 38.4)	17.5 (7.3, 34.5)		
N cases (score≥60)	51	56	54	35	24		
Unadjusted	1.00 (ref.)	1.10 (0.77, 1.58)	1.06 (0.74, 1.52)	0.69 (0.45, 1.04)	0.47 (0.29, 0.75)	<0.001	0.79 (0.69, 0.89)
Multivariable ‡	1.00 (ref.)	1.12 (0.77, 1.63)	1.25 (0.86, 1.81)	0.88 (0.57, 1.35)	0.56 (0.34, 0.92)	0.011	0.86 (0.74, 0.99)
Multivariable + BMI	1.00 (ref.)	1.08 (0.75, 1.54)	0.99 (0.70, 1.41)	0.85 (0.56, 1.30)	0.59 (0.36, 0.96)	0.017	0.84 (0.73, 0.98)
Multivariable + BMI & WC	1.00 (ref.)	1.01 (0.71, 1.45)	0.99 (0.70, 1.40)	0.84 (0.56, 1.28)	0.58 (0.35, 0.95)	0.019	0.84 (0.72, 0.98)
range	1.83-7.56	7.57-8.29	8.30-8.88	8.89-9.53	9.54-12.12		
N total (n=2568)	514	514	513	514	513		
NAFLD-score, *median* (iqr)§	-1.9 (-2.4, -1.3)	-1.8 (-2.3, -1.2)	-1.9 (-2.4, -1.3)	-2.0 (-2.5, -1.3)	-2.1 (-2.5, -1.6)		
N cases (score≥-0.640)	63	70	67	59	38		
Unadjusted	1.00 (ref.)	1.11 (0.81, 1.53)	1.07 (0.77, 1.47)	0.94 (0.67, 1.31)	0.60 (0.41, 0.89)	0.006	0.88 (0.79, 0.99)
Multivariable ‡	1.00 (ref.)	1.16 (0.83, 1.62)	1.16 (0.83, 1.61)	1.02 (0.72, 1.46)	0.66 (0.44, 1.00)	0.039	0.92 (0.81, 1.04)
Multivariable + BMI	1.00 (ref.)	1.27 (0.91, 1.76)	1.24 (0.89, 1.72)	1.16 (0.81, 1.65)	0.84 (0.55, 1.28)	0.34	0.98 (0.87, 1.11)
Multivariable + BMI & WC	1.00 (ref.)	1.25 (0.90, 1.73)	1.24 (0.89, 1.72)	1.16 (0.82, 1.65)	0.83 (0.54, 1.27)	0.34	0.99 (0.87, 1.12)
range	1.83-7.15	7.16-8.07	8.08-8.76	8.77-9.47	9.48-12.18		
N total (n=2351)	471	470	470	470	470		
Hepatic steatosis index, *median* (iqr)†	32.4 (30.5, 34.4)	32.3 (30.3, 34.6)	32.5 (30.4, 34.5)	32.6 (30.6, 34.4)	32.4 (30.3, 34.1)		
N cases (score>36)	166	123	120	120	103		
Unadjusted	1.00 (ref.)	0.74 (0.61, 0.90)	0.72 (0.59, 0.88)	0.72 (0.59, 0.88)	0.62 (0.50, 0.76)	<0.001	0.85 (0.79, 0.90)
Multivariable ‡	1.00 (ref.)	0.77 (0.61, 0.96)	0.79 (0.63, 1.00)	0.83 (0.65, 1.04)	0.70 (0.55, 0.91)	0.070	0.90 (0.82, 0.98)

Abbreviations: iqr, interquartile range; SES, socio-economic status; BMI, body mass index; WC, waist circumference; NAFLD, non-alcoholic fatty liver disease.
Statistical analysis using Poisson regression with robust standard errors; results are expressed as risk ratios and (95% confidence intervals).
* In categorical analysis, the population was divided into five groups by quintiles (Q1-Q5) of the Mediterranean diet score; Standard deviation was 1.21 for the multivariable analyses.
† Calculated based on an algorithm including BMI, waist circumference, triglycerides, and gamma-glutamyl transferase.
‡ Adjusted for age (years), sex, marital status (single, married/cohabiting, and widowed/Separated/divorced), occupational status (working and not working), education level (university, high school, apprenticeship, and mandatory education), smoking status (never, former, and current), energy intake (kcal/day), total energy expenditure (kcal/day), and date of dietary assessment.

For peer review only - http://bmjopen.bmj.com/site/about/guidelines.xhtml

§ Calculated based on an algorithm including presence of the metabolic syndrome and type 2 diabetes, and concentrations of fasting serum insulin, fasting serum aspartate-aminotransferase (AST), and the AST/alanine-aminotransferase ratio.

For peer review only

Table S6 Prospective association of the Mediterranean diet score with the risk of hepatic steatosis, CoLaus study, Switzerland (n=1632), Sensitivity analysis while excluding participants with either indices high (using FLI>30 instead of FLI≥60).

	Risk ratio (95% CI) across quintiles of Mediterranean diet score*					*P*-trend	Risk ratio (95% CI) Per SD increase*
	Q1	Q2	Q3	Q4	Q5		
range	1.83-7.63	7.64-8.35	8.36-8.92	8.93-9.59	9.60-12.1		
N total	327	326	327	326	326		
Fatty liver index, median (iqr)†	14.9 (7.6, 24.8)	12.7 (7.4, 23.7)	12.3 (5.5, 22.3)	11.8 (6.3, 22.7)	10.2 (5.5, 21.8)		
N cases (score≥60)	9	5	2	2	3		
Unadjusted	1.00 (ref.)	0.56 (0.19, 1.65)	0.22 (0.05, 1.02)	0.22 (0.05, 1.02)	0.33 (0.09, 1.22)	0.047	0.66 (0.45, 0.97)
Multivariable ‡	1.00 (ref.)	0.63 (0.20, 2.00)	0.14 (0.02, 0.96)	0.27 (0.05, 1.32)	0.39 (0.09, 1.68)	0.096	0.72 (0.47, 1.10)
Multivariable + BMI	1.00 (ref.)	0.68 (0.22, 2.05)	0.13 (0.02, 0.98)	0.26 (0.06, 1.15)	0.41 (0.10, 1.70)	0.087	0.73 (0.48, 1.11)
Multivariable + BMI + WC	1.00 (ref.)	0.50 (0.18, 1.42)	0.12 (0.01, 0.98)	0.22 (0.05, 1.03)	0.39 (0.09, 1.67)	0.093	0.71 (0.46, 1.09)
NAFLD-score, median (iqr)§	-2.2 (-2.5, -1.8)	-2.2 (-2.5, -1.7)	-2.2 (-2.5, -1.8)	-2.2 (-2.6, -1.8)	-2.2 (-2.6, -1.8)		
N cases (score≥-0.640)	13	14	10	13	12		
Unadjusted	1.00 (ref.)	1.08 (0.52, 2.26)	0.77 (0.34, 1.73)	1.00 (0.47, 2.13)	0.93 (0.43, 2.00)	0.79	0.94 (0.73, 1.20)
Multivariable ‡	1.00 (ref.)	1.13 (0.55, 2.32)	0.79 (0.35, 1.76)	1.10 (0.52, 2.34)	0.98 (0.45, 2.15)	0.94	0.95 (0.73, 1.24)
Multivariable + BMI	1.00 (ref.)	1.25 (0.60, 2.60)	0.81 (0.36, 1.84)	1.20 (0.56, 2.58)	1.13 (0.51, 2.52)	0.82	0.98 (0.75, 1.28)
Multivariable + BMI + WC	1.00 (ref.)	1.17 (0.57, 2.44)	0.85 (0.37, 1.93)	1.13 (0.52, 2.45)	1.12 (0.50, 2.53)	0.84	0.98 (0.75, 1.28)

Abbreviations: iqr, interquartile range; BMI, body mass index; WC, waist circumference; NAFLD, non-alcoholic fatty liver disease.
Statistical analysis using Poisson regression with robust standard errors; results are expressed as risk ratios and (95% confidence intervals).
* In categorical analysis, the population was divided into five groups by quintiles (Q1-Q5) of the Mediterranean diet score; Standard deviation was 1.21 for the multivariable analyses.
† Calculated based on an algorithm including BMI, waist circumference, triglycerides, and gamma-glutamyl transferase.
‡ Adjusted for age (years), sex, marital status (single, married/cohabiting, and widowed/Separated/divorced), occupational status (working and not working), education level (university, high school, apprenticeship, and mandatory education), smoking status (never, former, and current), energy intake (kcal/day), total energy expenditure (kcal/day), and date of dietary assessment.
§ Calculated based on an algorithm including presence of the metabolic syndrome and type 2 diabetes, and concentrations of fasting serum insulin, fasting serum aspartate-aminotransferase (AST), and the AST/alanine-aminotransferase ratio.

For peer review only

For peer review only - http://bmjopen.bmj.com/site/about/guidelines.xhtml

Table S7 Association of the Mediterranean diet score with change in BMI and waist circumference, CoLaus study, Switzerland.

	β coefficient (95% CI) across quintiles of Mediterranean diet score*					*P*-trend	β coefficient (95% CI) Per SD increase*
	Q1	Q2	Q3	Q4	Q5		
range	1.83-7.63	7.64-8.35	8.36-8.92	8.93-9.59	9.60-12.1		
N total	458	458	457	458	457		
ΔBMI, mean±SD†	0.48 ± 1.62	0.61 ± 1.53	0.42 ± 1.44	0.50 ± 1.52	0.40 ± 1.52		
Multivariable ‡	1.00 (ref.)	0.12 (-0.08, 0.33)	-0.08 (-0.28, 0.13)	-0.04 (-0.25, 0.16)	-0.16 (-0.37, 0.04)	0.038	-0.08 (-0.15, -0.02)
ΔWaist circumference, mean±SD§	1.04 ± 6.53	0.74 ± 6.42	0.19 ± 6.00	0.57 ± 6.33	0.17 ± 6.19		
Multivariable ‡	1.00 (ref.)	-0.51 (-1.36, 0.34)	-0.85 (-1.69, 0.00)	-0.47 (-1.32, 0.38)	-0.82 (-1.68, 0.03)	0.10	-0.33 (-0.61, -0.06)

Abbreviations: BMI, Body mass index.

Statistical analysis using linear regression; results are expressed as β coefficients and (95% confidence intervals).

* In categorical analysis, the population was divided into five groups by quintiles (Q1-Q5) of the Mediterranean diet score; Standard deviation was 1.20 for the multivariable analyses.

† Calculated by subtracting BMI at baseline from BMI at follow-up.

‡ Adjusted for age (years), sex, marital status (single, married/cohabitant, and widowed/Separated/divorced), occupational status (working and not working), education level (university, high school, apprenticeship, and mandatory education), smoking status (never, former, and current), energy intake (kcal/day), total energy expenditure (kcal/day), and date of dietary assessment.

§ Calculated by subtracting waist circumference at baseline from waist circumference at follow-up.

For peer review only

Table S8 Association of the Mediterranean diet score with the GGT, ALT, and AST, CoLaus study, Switzerland.

	β coefficient (95% CI) across quintiles of Mediterranean diet score*					*P*-trend	β coefficient (95% CI) Per SD increase *
	Q1	Q2	Q3	Q4	Q5		
range	1.83-7.63	7.64-8.35	8.36-8.92	8.93-9.59	9.60-12.12		
N total	458	458	457	458	457		
GGT (U/l), *median* (iqr)	21 (15, 29)	20 (14, 29)	19 (14, 26)	18 (14, 28)	17 (13, 26)		
Multivariable †	1.00 (ref.)	-2.63 (-9.01, 3.76)	-5.71 (-12.07, 0.66)	-4.24 (-10.64, 2.16)	-6.03 (-12.45, 0.38)	0.063	-1.53 (-3.60, 0.53)
Multivariable + BMI	1.00 (ref.)	-2.86 (-9.24, 3.53)	-5.70 (-12.06, 0.66)	-4.50 (-10.90, 1.90)	-6.52 (-12.95, -0.09)	0.047	-1.66 (-3.73, 0.41)
Multivariable + BMI & WC	1.00 (ref.)	-2.93 (-9.32, 3.45)	-5.72 (-12.08, 0.64)	-4.54 (-10.94, 1.86)	-6.53 (-12.96, -0.10)	0.047	-1.65 (-3.72, 0.41)
ALT (U/l), *median* (iqr)	20 (16, 26)	20.5 (17, 26)	20 (16, 26)	19.5 (16, 25)	20 (16, 25)		
Multivariable †	1.00 (ref.)	0.03 (-0.02, 0.08)	0.03 (-0.02, 0.08)	-0.013 (-0.06, 0.04)	0.002 (-0.05, 0.05)	0.45	-0.006 (-0.02, 0.01)
Multivariable + BMI	1.00 (ref.)	0.04 (-0.01, 0.08)	0.03 (-0.02, 0.08)	-0.009 (-0.06, 0.04)	0.008 (-0.04, 0.06)	0.60	-0.005 (-0.02, 0.01)
Multivariable + BMI & WC	1.00 (ref.)	0.04 (-0.01, 0.08)	0.03 (-0.02, 0.08)	-0.01 (-0.06, 0.04)	0.008 (-0.04, 0.06)	0.60	-0.005 (-0.02, 0.01)
AST (U/l), *median* (iqr)	22 (19, 25)	22 (19, 26)	22 (19, 25)	22 (19, 26)	22 (19, 25)		
Multivariable †	1.00 (ref.)	0.02 (-0.01, 0.06)	0.004 (-0.03, 0.04)	0.011 (-0.03, 0.05)	0.015 (-0.02, 0.05)	0.66	0.003 (-0.01, 0.01)
Multivariable + BMI	1.00 (ref.)	0.02 (-0.02, 0.06)	0.004 (-0.03, 0.04)	0.009 (-0.03, 0.04)	0.011 (-0.03, 0.05)	0.80	0.002 (-0.01, 0.01)
Multivariable + BMI & WC	1.00 (ref.)	0.02 (-0.02, 0.06)	0.004 (-0.03, 0.04)	0.009 (-0.03, 0.04)	0.011 (-0.03, 0.05)	0.80	0.002 (-0.01, 0.01)

Abbreviations: iqr, interquartile range; GGT, gamma-glutamyl transferase; SES, socio economic status; BMI, body mass index; WC, waist circumference; ALT, Alanine transaminase; AST, aspartate-aminotransferase.

Statistical analysis using linear regression; results are expressed as β coefficients and (95% confidence intervals).

* In categorical analysis, the population was divided into five groups by quintiles (Q1-Q5) of the Mediterranean diet score; Standard deviation was 1.20 for the multivariable analyses.

† Adjusted for age (years), sex, marital status (single, married/cohabitant, and widowed/Separated/divorced), occupational status (working and not working), education level (university, high school, apprenticeship, and mandatory education), smoking status (never, former, and current), energy intake (kcal/day), total energy expenditure (kcal/day), and date of dietary assessment.

For peer review only - http://bmjopen.bmj.com/site/about/guidelines.xhtml

STROBE 2007 (v4) Statement—Checklist of items that should be included in reports of *cohort studies*

Section/Topic	Item #	Recommendation	Reported on page #
**Title and abstract**	1	(*a*) Indicate the study's design with a commonly used term in the title or the abstract	3
		(*b*) Provide in the abstract an informative and balanced summary of what was done and what was found	3
**Introduction**			
Background/rationale	2	Explain the scientific background and rationale for the investigation being reported	6
Objectives	3	State specific objectives, including any prespecified hypotheses	7
**Methods**			
Study design	4	Present key elements of study design early in the paper	7
Setting	5	Describe the setting, locations, and relevant dates, including periods of recruitment, exposure, follow-up, and data collection	7
Participants	6	(*a*) Give the eligibility criteria, and the sources and methods of selection of participants. Describe methods of follow-up	7; Figure S1, and table S1
		(*b*) For matched studies, give matching criteria and number of exposed and unexposed	N/A
Variables	7	Clearly define all outcomes, exposures, predictors, potential confounders, and effect modifiers. Give diagnostic criteria, if applicable	Pages 7 to 10
Data sources/ measurement	8*	For each variable of interest, give sources of data and details of methods of assessment (measurement). Describe comparability of assessment methods if there is more than one group	Pages 7 to 10
Bias	9	Describe any efforts to address potential sources of bias	Pages 10 & 11
Study size	10	Explain how the study size was arrived at	12; Figure S1
Quantitative variables	11	Explain how quantitative variables were handled in the analyses. If applicable, describe which groupings were chosen and why	Pages 7 to 10
Statistical methods	12	(*a*) Describe all statistical methods, including those used to control for confounding	Pages 10 & 11
		(*b*) Describe any methods used to examine subgroups and interactions	Pages 10 & 11
		(*c*) Explain how missing data were addressed	Pages 10 & 11
		(*d*) If applicable, explain how loss to follow-up was addressed	Pages 10 & 11
		(*e*) Describe any sensitivity analyses	Pages 10 & 11

For peer review only

**Results**			
Participants	13*	(a) Report numbers of individuals at each stage of study—eg numbers potentially eligible, examined for eligibility, confirmed eligible, included in the study, completing follow-up, and analysed	12
		(b) Give reasons for non-participation at each stage	N/A
		(c) Consider use of a flow diagram	Figure 1
Descriptive data	14*	(a) Give characteristics of study participants (eg demographic, clinical, social) and information on exposures and potential confounders	12; Table 1 and table S1 & S2
		(b) Indicate number of participants with missing data for each variable of interest	12
		(c) Summarise follow-up time (eg, average and total amount)	12
Outcome data	15*	Report numbers of outcome events or summary measures over time	12; Table S2
Main results	16	(*a*) Give unadjusted estimates and, if applicable, confounder-adjusted estimates and their precision (eg, 95% confidence interval). Make clear which confounders were adjusted for and why they were included	Pages 12 & 13; Table 2, Figure 1, and supplementary Tables and Figures
		(*b*) Report category boundaries when continuous variables were categorized	Pages 12 & 13; Table 2, Figure 1, and supplementary Tables and Figures
		(*c*) If relevant, consider translating estimates of relative risk into absolute risk for a meaningful time period	
Other analyses	17	Report other analyses done—eg analyses of subgroups and interactions, and sensitivity analyses	Pages 13 & 14; Supplementary Tables S3 to S8
**Discussion**			
Key results	18	Summarise key results with reference to study objectives	14
**Limitations**	19	Discuss limitations of the study, taking into account sources of potential bias or imprecision.	pages 17 & 18
Interpretation	20	Give a cautious overall interpretation of results considering objectives, limitations, multiplicity of analyses, results from similar studies, and other relevant evidence	Pages 14 to 18
Generalisability	21	Discuss the generalisability (external validity) of the study results	pages 17 & 18
**Other information**			
Funding	22	Give the source of funding and the role of the funders for the present study and, if applicable, for the original study on	2

For peer review only

		which the present article is based	

*Give information separately for cases and controls in case-control studies and, if applicable, for exposed and unexposed groups in cohort and cross-sectional studies.

Note: An Explanation and Elaboration article discusses each checklist item and gives methodological background and published examples of transparent reporting. The STROBE checklist is best used in conjunction with this article (freely available on the Web sites of PLoS Medicine at http://www.plosmedicine.org/, Annals of Internal Medicine at http://www.annals.org/, and Epidemiology at http://www.epidem.com/). Information on the STROBE Initiative is available at www.strobe-statement.org.

For peer review only

BMJ Open

The association between adherence to the Mediterranean diet and hepatic steatosis: the Swiss CoLaus prospective cohort study

Journal:	*BMJ Open*
Manuscript ID	bmjopen-2020-040959.R3
Article Type:	Original research
Date Submitted by the Author:	13-Nov-2020
Complete List of Authors:	Khalatbari-Soltani, Saman; The University of Sydney School of Public Health, Faculty of Medicine and Health; ARC Centre for Excellence in Population Ageing Research<br>Marques-Vidal, Pedro; University Hospital of Lausanne, Department of Internal Medicine<br>Imamura, Fumiaki; University of Cambridge School of Clinical Medicine, Medical Research Council Epidemiology Unit<br>Forouhi, Nita; University of Cambridge School of Clinical Medicine, Medical Research Council Epidemiology Unit
**Primary Subject Heading**:	Epidemiology
Secondary Subject Heading:	Nutrition and metabolism, Gastroenterology and hepatology, Public health
Keywords:	NUTRITION & DIETETICS, EPIDEMIOLOGY, PUBLIC HEALTH, Hepatology < INTERNAL MEDICINE

SCHOLARONE™
Manuscripts

I, the Submitting Author has the right to grant and does grant on behalf of all authors of the Work (as defined in the below author licence), an exclusive licence and/or a non-exclusive licence for contributions from authors who are: i) UK Crown employees; ii) where BMJ has agreed a CC-BY licence shall apply, and/or iii) in accordance with the terms applicable for US Federal Government officers or employees acting as part of their official duties; on a worldwide, perpetual, irrevocable, royalty-free basis to BMJ Publishing Group Ltd ("BMJ") its licensees and where the relevant Journal is co-owned by BMJ to the co-owners of the Journal, to publish the Work in this journal and any other BMJ products and to exploit all rights, as set out in our licence.

The Submitting Author accepts and understands that any supply made under these terms is made by BMJ to the Submitting Author unless you are acting as an employee on behalf of your employer or a postgraduate student of an affiliated institution which is paying any applicable article publishing charge ("APC") for Open Access articles. Where the Submitting Author wishes to make the Work available on an Open Access basis (and intends to pay the relevant APC), the terms of reuse of such Open Access shall be governed by a Creative Commons licence – details of these licences and which Creative Commons licence will apply to this Work are set out in our licence referred to above.

Other than as permitted in any relevant BMJ Author's Self Archiving Policies, I confirm this Work has not been accepted for publication elsewhere, is not being considered for publication elsewhere and does not duplicate material already published. I confirm all authors consent to publication of this Work and authorise the granting of this licence.

For peer review only

TITLE PAGE

Title: The association between adherence to the Mediterranean diet and hepatic steatosis: the Swiss CoLaus prospective cohort study

Saman Khalatbari-Soltani[1,2,3], Pedro Marques-Vidal[3], Fumiaki Imamura[4*], and Nita G. Forouhi[4*]

[1] The University of Sydney School of Public Health, Faculty of Medicine and Health, New South Wales, Australia; [2] ARC Centre of Excellence in Population Ageing Research (CEPAR), University of Sydney, Sydney, Australia; [3] Department of Internal Medicine, Internal Medicine, Lausanne University Hospital (CHUV), rue du Bugnon 46, 1011 Lausanne, Switzerland; [4] Medical Research Council Epidemiology Unit, University of Cambridge, School of Clinical Medicine, Institute of Metabolic Science, Cambridge, CB2 0QQ, UK.

*FL and NGF are joint senior authors with equal contribution

Correspondence to:

Saman Khalatbari-Soltani, ARC Centre of Excellence in Population Ageing Research (CEPAR), School of Public Heath, Faculty of Medicine and Health, the University of Sydney, NSW, 2006, Sydney Australia, +61 (0) 431 711 144, saman.khalatbarisoltani@sydney.edu.au.

Nita G. Forouhi, Medical Research Council Epidemiology Unit, University of Cambridge, School of Clinical Medicine, Institute of Metabolic Science, Cambridge, CB2 0QQ, UK, +44 (0) 1223 769145, nita.forouhi@mrc-epid.cam.ac.uk.

Running title: Mediterranean diet and hepatic steatosis.

Word count: 4202

For peer review only

22 **Number of figures and tables:** Two tables and one figure

For peer review only

For peer review only - http://bmjopen.bmj.com/site/about/guidelines.xhtml

ABSTRACT

Objective: The Mediterranean diet has been promoted as a healthy dietary pattern but, whether the Mediterranean diet may help to prevent hepatic steatosis is not clear. This study aimed to evaluate the prospective association between adherence to the Mediterranean diet and risk of hepatic steatosis.

Design: Population-based prospective cohort study.

Setting: The Swiss CoLaus study.

Participants: We evaluated 2288 adults (65.4% women, aged 55.8±10.0 years) without hepatic steatosis at first follow-up in 2009-2012. Adherence to the Mediterranean diet was scaled as the Mediterranean diet score (MDS) based on the Mediterranean-diet Pyramid ascertained with responses to food-frequency questionnaires.

Outcome measures: New onset of hepatic steatosis was ascertained by two indices separately: the fatty liver index (FLI, ≥60 points) and the non-alcoholic fatty liver disease (NAFLD) score (≥-0.640 points). Prospective associations between adherence to the Mediterranean diet and risk of hepatic steatosis were quantified using Poisson regression.

Results: During a mean 5.3-years of follow-up, hepatic steatosis was ascertained in 153 (6.7%) participants by FLI criteria and in 208 (9.1%) by NAFLD-score. After multivariable adjustment, higher adherence to MDS was associated with lower risk of hepatic steatosis based on FLI: risk ratio (95% confidence interval) 0.84 (0.73, 0.96) per one standard deviation of MDS; 0.85 (0.73, 0.99) adjusted for BMI; and 0.85 (0.71, 1.02) adjusted for both BMI and waist circumference. When using NAFLD-score, no significant association was found between MDS and risk of hepatic steatosis [0.95 (0.83, 1.09)].

Conclusion: A potential role of the Mediterranean diet in the prevention of hepatic steatosis is suggested by the inverse association observed between adherence to the Mediterranean diet and incidence of hepatic steatosis based on the FLI. The inconsistency of this association

For peer review only - http://bmjopen.bmj.com/site/about/guidelines.xhtml

when hepatic steatosis was assessed by NAFLD-score points to the need for accurate population-level assessment of fatty liver and its physiologic markers.

Keywords: Mediterranean diet; hepatic steatosis; fatty liver disease; prospective study.

For peer review only

For peer review only - http://bmjopen.bmj.com/site/about/guidelines.xhtml

STRENGTHS AND LIMITATIONS OF THE STUDY

- This study had the benefit of a relatively large sample size and an average of 5.3 years of follow-up.
- We applied a definition of the Mediterranean diet that has been shown to be valid in a non-Mediterranean population.
- Our ascertainment of hepatic steatosis was based on two indices that have been validated for use in large epidemiological studies.
- We used dietary data measured only once at baseline, and intra-individual variation over time might be present which may weaken the observed associations towards the null; however, dietary intake in CoLaus was relatively stable, suggesting that lack of repeated dietary measures is unlikely to alter our findings substantially.
- The precision of the two hepatic steatosis indices used is different and may be influenced by the presence of steatohepatitis or advanced liver fibrosis.

For peer review only

INTRODUCTION

Hepatic steatosis is the most common cause of liver disease [1]. In westernized countries, hepatic steatosis affects up to 34% of the general population and up to 74% of obese individuals, depending on the definition used [2–4]. Hepatic steatosis—fat content of more than 5% of liver volume—is the first recognizable stage of non-alcoholic fatty liver disease (NAFLD) [1]. Hepatic steatosis, particularly NAFLD, may progress to end-stage liver disease including fibrosis, cirrhosis, and hepatocellular carcinoma [5]. Moreover, as hepatic steatosis increases the risk of metabolic syndrome, type 2 diabetes, and cardiovascular disease (CVD), its prevention is of public health importance [6]. An unhealthy dietary pattern remains one of the primary targets of lifestyle modification for the prevention and management of hepatic steatosis and NAFLD [7,8].

The Mediterranean diet has been recently recommended for treatment of NAFLD [9]. In recent years, a growing body of evidence supports the idea that the Mediterranean diet may be the reference nutritional profile for the prevention of hepatic steatosis development [10–12]. Adherence to the Mediterranean diet has been reported to have a beneficial impact on risks of CVD [13,14], type 2 diabetes [15], and metabolic syndrome [16]. Trial evidence demonstrated the potential benefits of the Mediterranean diet against progress of hepatic steatosis focusing on individuals with existing hepatic steatosis, either alone [17–22] or associated with metabolic risk factors such as obesity or diabetes [22–25]. Research among those without clinically manifest hepatic steatosis is restricted to observational evidence, reporting an inverse association that greater adherence to a Mediterranean diet is associated with lower prevalence of hepatic steatosis [26,27]. However, the cross-sectional design of these studies limits inference for causal associations and can be used mainly for hypothesis generation. Relevant longitudinal evidence for the primary prevention of hepatic steatosis or NAFLD has been reported only by the Framingham Heart Study, with a significant inverse

For peer review only

association of adherence to the Mediterranean diet with risk of hepatic steatosis in 1521 adults over 6 years of follow-up [28], but evidence is lacking in Europe.

Given the limited evidence from population-based epidemiological studies thus far, we aimed to investigate the prospective association between adherence to the Mediterranean diet and the risk of developing hepatic steatosis among adults without clinically manifest hepatic steatosis. We hypothesized that greater adherence to the Mediterranean diet would reduce the risk of hepatic steatosis.

For peer review only

METHODS

Study population

We evaluated participants in the CoLaus study, an ongoing population-based cohort investigating the clinical, biological, and genetic determinants of CVD in the city of Lausanne, Switzerland [29]. Inclusion criteria of the recruitment were adults of European origin, aged 35 to 75 years [29]. There were three study phases: baseline recruitment in 2003-2006 (n=6733), the first follow-up in 2009-2012 (n=5064), and the second follow-up in 2014-2017 (n=4881). We conducted dietary assessment at the first follow-up and therefore considered the first follow-up as the study baseline. Fatty liver index (FLI) and NAFLD-score, two indices of hepatic steatosis, were available at baseline [27]. If participants met the joint criterion of FLI $\geq$ 60 or NAFLD-score $\geq$ -0.640 at baseline, we excluded them as prevalent cases (see below) (n=2036). We also excluded participants with missing information on diet, outcome, and covariates (n=740) (**Figure S1**).

Patient and public involvement

Patients and/or the public were not involved in the design, conduct, reporting or dissemination plans of this research.

For peer review only - http://bmjopen.bmj.com/site/about/guidelines.xhtml

Dietary assessment

Participants completed a self-administered, 97-item, semi-quantitative food frequency questionnaire (FFQ) about their habitual dietary intake over the last four weeks [30], the validity of which had been assessed in canton Geneva against 24-hour recalls [30,31]. For each item, participants were instructed to report consumption frequencies by selecting one of the seven frequency options from "less than once during the last 4 weeks" to "2 or more times per day" and by selecting a usual serving size (smaller, equal, or bigger to a reference size).

Mediterranean diet scores

We derived the pyramid-based Mediterranean diet score (MDS) as a measure of adherence to the Mediterranean diet from responses to the FFQ as we conducted previously [27]. This MDS is based on the Mediterranean Dietary Pyramid proposed by the Mediterranean Diet Foundation for both Mediterranean and non-Mediterranean countries and accounting for the traditional Mediterranean diet, contemporary lifestyle, and food environment [32]. We have previously reported that this MDS scoring algorithm predicted CVD incidence [33], as well as the prevalence of hepatic steatosis [27] in non-Mediterranean populations. Briefly, a continuous score of 0 to 1 was assigned for each recommended level of the 15 components of the pyramid (vegetables, legumes, and fish as healthy items; red meat, processed meat, potato, and sweets as unhealthy items; and fruits, nuts, cereals, eggs, dairy, white meat, and alcoholic beverages as items for which moderate consumption was recommended). The resulting MDS ranges between 0 and 15 on a continuous scale. The MDS calculation was adjusted to an energy intake of 2000 kcal/d (8.37 MJ/day) by applying a regression-residual technique for energy adjustment to each food group variable [33,34].

Ascertainment of hepatic steatosis

For peer review only - http://bmjopen.bmj.com/site/about/guidelines.xhtml

Two indices of hepatic steatosis were evaluated: fatty liver index (FLI) [35] and NAFLD liver fat score [36]. FLI was calculated based on a logistic function including body-mass index (BMI), waist circumference, fasting triglycerides, and gamma-glutamyl transferase (GGT) levels as follows:

$$FLI = 1 / (1+e^{-(0.953 \times \ln(\text{triglycerides}) + 0.139 \times BMI + 0.718 \times \ln(GGT) + 0.053 \times \text{waist circumference} - 15.745)})$$

FLI×100 ranges from 0 to 100. Presence of hepatic steatosis was defined by FLI ≥ 60, a value with a sensitivity of 61% and a specificity of 86% [35]. FLI was tested previously in comparison to ultrasonography with an area under the receiver operating characteristic curve of 0.78 (OR 95% CI: 0.77, 0.83) [37].

The NAFLD-score was calculated based on an algorithm including a logistic function with the presence of metabolic syndrome defined by criteria of International Diabetes Federation [38], presence of type 2 diabetes, and fasting concentrations of insulin, aspartate-aminotransferase (AST), and the AST/ alanine transaminase (ALT) ratio:

$$\text{NAFLD-score} = -2.89 + 1.18 \times \text{metabolic syndrome (yes/no)} + 0.45 \times \text{type 2 diabetes (yes /no)} + 0.15 \times \text{fasting-insulin (mU/L)} + 0.04 \times \text{fasting-AST (U/L)} - 0.94 \times AST/ALT$$

Presence of hepatic steatosis was defined by a NAFLD-score ≥ -0.640, a value with a sensitivity of 86% and a specificity of 71%, when compared to proton magnetic resonance imaging [36].

Assessment of covariates at baseline

Sociodemographic, lifestyle and health characteristics were collected by self-administered questionnaires. Age, sex, marital status, occupational status, and educational level were included as indicators of sociodemographic condition. Smoking status was classified as ‘never’, ‘former’, and ‘current’. Alcohol consumption was assessed by the

For peer review only - http://bmjopen.bmj.com/site/about/guidelines.xhtml

number of alcoholic beverage units consumed in the past week and further categorized as ‘abstainers’ (0 unit/week), ‘moderate’ (1–21 units/week for men, 1–14 for women), and ‘heavy’ (>21 units/week for men, >14 for women) drinkers (one unit corresponds to 8 g of alcohol). Physical activity was assessed with a self-administered quantitative physical activity frequency questionnaire [39]. Health characteristics included presence of metabolic syndrome and family history of diabetes. Anthropometric and blood pressure measurements were obtained using standard procedures and equipment as previously described [29]. Plasma triglycerides, high-density lipoprotein cholesterol, and glucose were measured using standard enzymatic methods and ALT, AST, and GGT were measured using reference methods as standardized by the International Federation of Clinical Chemistry.

Statistical analysis

Statistical analyses were performed using Stata (version 15; StataCorp, College Station, TX, USA) with a two-sided test with α=0.05. Descriptive statistics were obtained in the participants included in this study in comparison to those excluded from this study. Cohen’s kappa statistics were calculated to assess the agreement between the FLI and NAFLD-score.

MDS as a measure of adherence to the Mediterranean diet was evaluated both categorically (quintiles) and continuously scaled as one SD unit. The association of MDS with the risk of hepatic steatosis was assessed using multivariable-adjusted Poisson regression models with robust standard errors and estimating risk ratios (RRs) and 95% confidence intervals (CIs). Models were adjusted for age, sex, marital status, occupational status, educational level, smoking status, energy intake, total energy expenditure, and date of dietary assessment (to adjust for seasonality). We further adjusted for BMI and waist circumference as potential confounders or factors on the causal pathway to assess the possible impact of

For peer review only - http://bmjopen.bmj.com/site/about/guidelines.xhtml

overall and central adiposity on the association of the Mediterranean diet and hepatic steatosis.

Additionally, we also adjusted for changes in BMI categories between baseline and follow-up; for alcohol consumption (units/week); for clinical variables of metabolic risk [blood pressure >130/85 mm Hg (yes/no), triglycerides >1.7 mmol/L (yes/no), high-density lipoprotein level <1.29 mmol/L for men and <1.03 mmol/L for women (yes/no), and glucose level ≥5.6 mmol/L (yes/no)] [38]; and for family history of diabetes and metabolic syndrome (only for FLI) to examine their influence on the association of interest.

Possible interactions between MDS and age, sex, BMI, and alcohol consumption were tested using the Wald test. Several sensitivity analyses were conducted to examine the robustness of the observed findings. First, to assess the role of alcohol consumption (as alcohol is a risk factor for fatty liver accumulation), we excluded the alcohol component from the MDS, while adjusting for alcohol consumption as a covariate. We took the same approaches for the other MDS components to assess the impact of each component on the observed associations. Second, we conducted separate analyses after excluding participants with BMI≥30 kg/m^2; implausible energy intake (<500 or >3500 kcal/day in women and <800 or >4000 kcal/day in men); excessive alcohol consumption; prevalent diabetes (defined as glycated haemoglobin≥48 mmol/mol, or fasting plasma glucose ≥7.0 mmol/L, or use of hypoglycaemic drugs or insulin); or probable secondary causes of hepatic steatosis such as hepatitis B or C, HIV, hepatotoxic, or autoimmune disease medications. We evaluated the robustness of the results to an alternative definition of prevalent hepatic steatosis. While we excluded participants with prevalent hepatic steatosis using the specified cut-offs of FLI or NAFLD-score in the primary analysis, we used each of the two indices separately in sensitivity analyses, whereby we evaluated 2652 adults in longitudinal analysis based on FLI;

For peer review only - http://bmjopen.bmj.com/site/about/guidelines.xhtml

and 2568 adults, based on NAFLD. Finally, we used more restrictive cutpoints and excluded participants with NAFLD-score ≥-0.640 or with FLI>30.

In a post-hoc analysis, due to inconsistency of the associations observed for FLI and NAFLD-score, we also calculated the hepatic steatosis index (HSI) [40] based on the ratio of AST/ALT, BMI, presence of type 2 diabetes, and sex:

$$HSI = 8*AST/ALT + BMI + 2\ (\text{presence of diabetes}) + 2\ (\text{if women})$$

Presence of hepatic steatosis was defined by a HSI>36. After excluding participants with HSI>36 at baseline (n=2674), we evaluated 2351 adults.

In a post-hoc analysis pertaining to sensitivity of the results to model covariates and for better understanding of potential mechanisms, longitudinal associations of MDS with follow-up measures of log-transformed GGT, ALT, and AST levels and with changes in BMI and waist circumference from baseline to follow-up were examined using multivariable-adjusted linear regression. These results were expressed as β coefficient (95% CIs) for changes in each measure per 1-SD difference in MDS.

RESULTS

Participant characteristics

Of the initial 5064 participants, 2776 (54.8%) were excluded, leaving 2288 participants (65.4% women; 55.8±10.0 years) for analysis. Participants included were more likely to be women, show higher sociodemographic characteristics and lower BMI, waist circumference, liver enzymes, or prevalence of metabolic syndrome in comparison to excluded individuals (**Table S1**). Being in the highest quintile of MDS was higher among women compared to men, positively correlated with sociodemographic characteristics, and

For peer review only

For peer review only - http://bmjopen.bmj.com/site/about/guidelines.xhtml

negatively correlated with being current smokers, heavy alcohol drinkers, and with BMI, waist circumference, GGT, and TG (**Table 1**).

Adherence to the Mediterranean diet and risk of hepatic steatosis

After a mean 5.3 (SD: 0.5) years of follow-up, there were 153 (6.7%) and 208 (9.1%) participants with hepatic steatosis based on FLI and NAFLD-score, respectively (**Table S2**). Case identification by FLI and NAFLD-score was modestly concordant (kappa=0.60).

Multivariable-adjusted analysis showed an inverse association between MDS quintiles and risk of hepatic steatosis based on FLI (p_{trend}<0.006) with RR (95% CI) comparing the top to the bottom category of 0.50 (0.28, 0.91). The inverse associations across quintiles of MDS weakened after adjustment for BMI (p_{trend}=0.031) or both BMI and waist circumference (p_{trend}=0.034) (**Table 2**): RR (95% CI)=0.61 (0.34, 1.09) and 0.60 (0.34, 1.08), respectively. In analyses using MDS as a continuous variable, the inverse association with risk of hepatic steatosis based on FLI [RR 0.84 (0.73, 0.96) per one SD of MDS] remained unchanged but getting imprecise after adjustment for BMI [RR 0.85 (0.73, 0.99)] and adjustment for both BMI and waist circumference [0.85 (0.71, 1.02)]. In sensitivity analysis, the magnitude of the inverse associations little changed after further adjustment for alcohol consumption, presence of metabolic syndrome, changes in BMI categories or clinical variables (medication use or prevalent diseases), while adjustment for BMI and clinical variables increased standard errors (**Table S3**).

Conversely, there was no association between MDS and the risk of hepatic steatosis defined by NAFLD-score criteria, with RRs (95% CIs) ranging from 0.93 (0.82, 1.05) to 1.00 (0.86, 1.17) over different regression models (**Table 2** and **Table S3**).

Interaction and sensitivity analyses

For peer review only - http://bmjopen.bmj.com/site/about/guidelines.xhtml

No significant interactions were found between MDS and age, sex, BMI, or alcohol consumption on risk of hepatic steatosis ($p_{interaction}$>0.05; results not shown). The contribution of each component of the MDS on risk of hepatic steatosis was assessed by sequential subtraction of components from the score (**Figure 1**). Excluding the components of the MDS did not substantially affect the inverse associations with hepatic steatosis based on FLI; the magnitude of the associations remained reasonably stable, but it became weaker (p>0.05) after excluding fruits, cereals, dairy products, red or processed meat, or alcohol.

In sensitivity analyses, when excluding the alcohol component from the MDS but adjusting for alcohol consumption as a covariate, the inverse associations between MDS and risk of hepatic steatosis based on FLI became weaker (**Table S4**). The primary results were not different when excluding participants with BMI≥30 kg/m^2, excessive alcohol consumption, or secondary causes of hepatic steatosis (**Table S4**). Excluding participants with implausible energy intakes weakened the associations (**Table S4**). The analysis of an alternative definition of prevalent hepatic steatosis, excluding participants with only high FLI score at baseline, did not alter the significant inverse association between MDS and risk of FLI-based hepatic steatosis (**Table S5**). In post-hoc analyses, there was an inverse association between MDS quintiles and risk of hepatic steatosis based on HSI (p_{trend}=0.070) with RR (95% CI) comparing the top to the bottom category of 0.70 (0.55, 0.91); a significant inverse association with HSI was observed per one SD increase in MDS [RR 0.90 (0.82, 0.98)] (**Table S5**). Effect sizes were of slightly higher magnitude when excluding those with FLI>30 or NAFLD-score≥-0.640 at baseline, but CIs were wider due to smaller sample size (**Table S6**).

For NAFLD-score, no significant associations were found in any of the sensitivity analyses (**Tables S4 and S5**). The sole exception was when participants with a high NAFLD-score at baseline were excluded, where an inverse association between MDS quintiles and risk

For peer review only - http://bmjopen.bmj.com/site/about/guidelines.xhtml

of hepatic steatosis was present (p_{trend}=0.039), but this association was attenuated to the null after adjustment for BMI (**Table S5**).

Longitudinal analyses for adiposity and markers of hepatic function

In post-hoc exploratory analyses there were inverse associations of MDS with changes in BMI [β coefficient (95% CIs) per one SD higher MDS of -0.08 (-0.15, -0.02)] and in waist circumference [-0.33 (-0.61, -0.06)] (**Table S7**). For the markers of hepatic function, MDS showed a trend toward inverse association with GGT levels (p_{trend}=0.047) [β coefficient (95% CIs) per one SD higher MDS of -1.66 (-3.73, 0.41)], but not with ALT or AST levels (**Table S8**).

DISCUSSION

In this first population-based European study among adults free from clinically manifest hepatic steatosis to report on the prospective association between adherence to the Mediterranean diet and risk of hepatic steatosis, we found an inverse association between MDS and risk of hepatic steatosis based on FLI criteria. This relationship was attenuated to the null when controlled for general and central adiposity assessed by the BMI and waist circumference. In contrast, there was no association between adherence to the Mediterranean diet and risk of hepatic steatosis based on NAFLD-score criteria.

Current findings in context of other evidence

Our finding based on FLI is consistent with the only published prospective study relating the Mediterranean diet to hepatic steatosis, where each SD increase in MDS was estimated to decrease the odds for incident hepatic steatosis by 26% (95% CI 10%-39%) [28]. By contrast, the point estimate of the effect size in our study was smaller [(16% (95% CI 4%-27%)]. A possible explanation partly lies in methodological differences. We used biochemical

For peer review only - http://bmjopen.bmj.com/site/about/guidelines.xhtml

and anthropometric markers to estimate hepatic steatosis in the current study, while the previous study used computed tomography assessment.

No association was found between adherence to the Mediterranean diet and risk of hepatic steatosis based on the NAFLD-score. For possible explanations based on the differences in their components, the FLI includes GGT, while NAFLD-score includes AST and AST/ALT ratio; the FLI includes adiposity markers, while the NAFLD-score does not; the FLI includes a lipid marker (triglycerides) while NAFLD-score includes markers of glycaemic status. Previous studies showed a modest association of GGT and ALT (but not AST) with the prevalence of hepatic steatosis [1]. Indeed, our analysis showed an inverse association between MDS and GGT levels, but not with AST or ALT, and these findings could explain the discrepancy between the two indices. Notably, our sensitivity analyses using different definitions of hepatic steatosis showed an inverse association between MDS quintiles and risk of hepatic steatosis based on NAFLD-score after excluding participants with high baseline NAFLD-score only. This could be explained by modest concordance between the two measures. Of note, there were no statistically significant associations between MDS and risk of hepatic steatosis based on FLI after excluding participants with FLI>30 at baseline, which led to a smaller sample size and consequently a lower statistical power in our study. In a post-hoc analysis, we found an inverse association between adherence to the Mediterranean diet and hepatic steatosis as assessed by HSI, an alternative score to detect hepatic steatosis. Although, case identification by HSI and FLI (kappa=0.27) or NAFLD-score (Kappa=0.18) was weakly concordant; our finding based on FLI is consistent with HSI. This could be explained potentially by BMI as one of the components of both FLI and HSI, highlighting the importance of obesity for incident hepatic steatosis [41].

The inverse association between MDS and risk of hepatic steatosis defined by FLI remained significant after adjusting for BMI only but became imprecise and not significant

For peer review only

after adjusting for both BMI and waist circumference or for changes in BMI over the follow-up period. These results suggest the collinearity between the central adiposity and hepatic steatosis as the central adiposity may partly reflect hepatic steatosis. This biologically plausible finding is in agreement with previous cross-sectional findings for MDS and prevalent hepatic steatosis in CoLaus study and the British Fenland Study we reported [27] and a study in Hong Kong [42]. Our finding that the MDS was negatively associated with an increase in BMI after 5.3 years of follow-up is consistent with the findings from the Framingham Heart Study which observed the same trend over 6-year follow-up [28]; and from Nurses' Health Study and Health Professionals' Follow-up Study evaluating 20-year longitudinal data with repeated self-reported measures of diet and adiposity measures [43].

Sequential subtraction of different components of the MDS showed that fruits, cereals, dairy products, red or processed meat, or alcohol partially accounted for the observed association. These findings agree with previous studies [26,28,44,45] including the Framingham Heart Study suggesting benefits of low consumption of red meat and high consumption of fruits or whole grains [28]; and a cross-sectional analysis from the PREDIMED study suggesting the benefit of low consumption of red meat [26]. Moreover, the Mediterranean diet is characterized by a moderate-to-high consumption of whole grains, which has been inversely associated with the likelihood of having NAFLD [46].

Possible mechanisms and implications

Hepatic steatosis is associated with a number of metabolic risk factors including insulin resistance, type 2 diabetes, dyslipidaemia, metabolic syndrome, and oxidative stress [47]. Mediterranean-diet associated phenolic compounds (phenolic acids and polyphenols) found in fruits and vegetables and high levels of monounsaturated fatty acids of olive oil have been shown to inhibit *de* novo lipogenesis, improve peripheral insulin sensitivity, and reduce

For peer review only - http://bmjopen.bmj.com/site/about/guidelines.xhtml

cardiovascular risk mainly due to their antioxidant, anti-inflammatory, and anti-fibrotic effects [18,48–51]. Moreover, different components of the Mediterranean diet, including omega 3 polyunsaturated fatty acids (PUFA), fibre, and antioxidant rich-foods, are inversely associated with hepatic steatosis [52,53]. One meta-analysis of interventional studies reported that omega-3 PUFA were negatively associated with hepatic steatosis [54]. The Mediterranean diet is also low in saturated fat, which has been demonstrated to increase hepatic triglycerides content and hepatic insulin resistance [55,56]. Finally, the high fibre content of the Mediterranean diet has been associated with reduced hepatic fat [18,52].

Hepatic steatosis is associated with cardiometabolic diseases and substantially impacts public health [13,15,16]. Thus, our finding of an inverse association between adherence to the Mediterranean diet and risk of hepatic steatosis would support the importance of dietary advice for the prevention of hepatic steatosis as well as its treatment. However, future work should confirm whether or not the clinical importance of the Mediterranean diet for the prevention of hepatic steatosis is independent of obesity or central adiposity.

Strengths and limitations

To our knowledge, this is the first European prospective study assessing the association between the Mediterranean diet and risk of hepatic steatosis. The study had the benefit of a relatively large sample size and an average of 5.3 years of follow-up, and applied a definition of the Mediterranean diet that has been shown to be valid in a non-Mediterranean population [33].

Several limitations of this study merit consideration. Measurement error and recall bias are inevitable when using self-reported dietary instruments, limiting the ability to precisely measure adherence to the Mediterranean diet, although adjustment for energy intake may have reduced the magnitude of measurement error [57]. We used diet data measured only

For peer review only

For peer review only - http://bmjopen.bmj.com/site/about/guidelines.xhtml

at baseline but recognise that intra-individual variation over time might be present which would be expected to weaken the observed associations and hence our findings may be biased towards the null. However, in CoLaus, average change in estimated total energy intake from first to second follow-up was 51 kcal/day and changes for each macronutrient (expressed as % of total energy intake) were about 1% (data not shown). Thus, dietary intake in CoLaus was relatively stable, suggesting that the lack of availability of repeat dietary measures is unlikely to alter our findings substantially.

Our ascertainment of hepatic steatosis was based on two indices, but not on liver biopsy or direct imaging assessment; hence, information bias (misdiagnosis) cannot be ruled out. Although liver biopsy is the gold standard for diagnosing hepatic steatosis, its use in apparently healthy participants would be unethical and unfeasible in a large population-based study. Further, previous studies have shown that FLI and NAFLD-score can accurately identify hepatic steatosis with good sensitivity and specificity, and these scores have been validated for use in large epidemiological studies [36,37,58,59]. But, although both FLI and NAFLD-score can indicate hepatic steatosis, their precision is variable [60]. Moreover, presence of steatohepatitis or advanced liver fibrosis could influence the relationship of FLI or NAFLD-score with hepatic steatosis [61]. Of note, our study was not designed to assess the role of adherence to the Mediterranean diet in hepatic fibrosis.

Although we adjusted for many relevant confounders and performed a series of sensitivity analyses, we cannot rule out residual confounding due to unmeasured variables or covariates measured with error. On the other hand, our adjustment for BMI and WC as markers of general and central adiposity may potentially be an over-adjustment if adiposity is on the causal pathway between dietary adherence and hepatic steatosis. However, since FLI may approximate hepatic steatosis with a degree of imprecision, adjusting for adiposity in these analyses may not represent adjusting the association between diet and steatosis directly,

For peer review only - http://bmjopen.bmj.com/site/about/guidelines.xhtml

but through a hepatic steatosis index that already includes adiposity measures in its definition. Nevertheless, our analytical approach is comprehensive, showing the results for crude analyses, followed by multivariable adjustment without and with further adjustment for adiposity markers. Future research with repeat measurements should further investigate this issue. Generalisability of our findings is limited because included participants seemed to be healthier than those excluded, and our findings were obtained in a single European population. Still, they confirm the findings from a previous prospective study conducted in the US [28], and might serve as a reference for other studies.

Conclusion

Adherence to the Mediterranean diet was inversely associated with risk of hepatic steatosis based on the FLI, and the association was independent of several known risk factors. Conversely, the association was not observed when using different criteria specifying the NAFLD-score. These findings support recommendations on following the Mediterranean diet for hepatic steatosis prevention in addition to the existing evidence for its benefit for cardiovascular disease prevention. Nonetheless, the findings also highlight the need for further research with more accurate measures of hepatic steatosis to replicate these findings in different populations and settings.

For peer review only - http://bmjopen.bmj.com/site/about/guidelines.xhtml

ABBREVIATIONS

NAFLD, non-alcoholic fatty liver disease; CVD; cardiovascular disease; FFQ, food frequency questionnaire; MDS, Mediterranean diet score; FLI, fatty liver index; BMI, body-mass index; TG, triglyceride; GGT, gamma-glutamyl transferase; AST, aspartate-aminotransferase; ALT, alanine transaminase; PUFA, polyunsaturated fatty acids.

DECLARATION

Acknowledgements: The authors are grateful to all the participants and staff of CoLaus study.

Competing interests: None.

Ethics approval and consent to participate: The institutional Ethics Committee of the University of Lausanne, which afterwards became the Ethics Commission of Canton Vaud (www.cer-vd.ch) approved the baseline CoLaus study (reference 16/03); the approval was renewed for the first (reference 33/09) and the second (reference 26/14) follow-ups.

The CoLaus study was performed in agreement with the Helsinki declaration and its former amendments, and all participants provided their written informed consent before entering the study.

Funding: The CoLaus study was and is supported by research grants from GlaxoSmithKline, the Faculty of Biology and Medicine of Lausanne, and the Swiss National Science Foundation (grants 33CSCO-122661, 33CS30-139468 and 33CS30-148401). SKS was supported by the Swiss National Science Foundation (Doc.Mobility number P1LAP3-171805). NGF and FI acknowledge core MRC support (MC_UU_12015/5), and NGF acknowledges NIHR

For peer review only

For peer review only - http://bmjopen.bmj.com/site/about/guidelines.xhtml

Biomedical Research Centre Cambridge: Nutrition, Diet, and Lifestyle Research Theme (IS-BRC-1215-20014).

Authors' contribution: SKS, FI, and NGF designed the study question and had full access to all the data in the study and took responsibility for the integrity and accuracy of the data. SKS performed the statistical analyses and she wrote the first draft with supervision from FI, PMV, and NGF. All authors: contributed to interpretation of data, revised the article critically for important intellectual content, and approved the final version of the manuscript.

Data availability statement: Non-identifiable individual-level data are available for researchers who seek to answer questions related to health and disease in the context of research projects who meet the criteria for data sharing by research committees. Please follow the instructions at https://www.colaus-psycolaus.ch/ for information on how to submit an application for gaining access to CoLaus data.

For peer review only

SUPPLEMENTARY INFORMATION

Figure S1 Sample selection flow chart.

Table S1 Characteristics of the participants included and excluded from the analysis, CoLaus study, Switzerland.

Table S2 Baseline characteristics of the participants according to the risk of hepatic steatosis, defined by the fatty liver index or the NAFLD-score, CoLaus study, Switzerland.

Table S3 Prospective association of the Mediterranean diet score with the risk of hepatic steatosis, CoLaus study, Switzerland: assessment of influence of other potential covariates.

Table S4 Sensitivity analyses for the association of the Mediterranean diet score with the risk of hepatic steatosis, CoLaus study, Switzerland.

Table S5 Prospective association of the Mediterranean diet score with the risk of hepatic steatosis, CoLaus study, Switzerland: sensitivity analysis while excluding participants with only one of the indices high at baseline and using hepatic steatosis index as an alternative score.

Table S6 Prospective association of the Mediterranean diet score with the risk of hepatic steatosis, CoLaus study, Switzerland (n=1632). Sensitivity analysis after excluding participants with either indices high (using FLI>30 instead of FLI≥60).

Table S7 Association of the Mediterranean diet score with change in BMI and waist circumference, CoLaus study, Switzerland.

Table S8 Association of the Mediterranean diet score with GGT, ALT, and AST, CoLaus study, Switzerland.

For peer review only

For peer review only - http://bmjopen.bmj.com/site/about/guidelines.xhtml

Figure caption

Figure 1 Prospective association of the Mediterranean diet score with the risk of hepatic steatosis, CoLaus study, Switzerland (n=2288): sensitivity analysis to examine influence of each component of Mediterranean diet.

Abbreviations: FLI, fatty liver index; NAFLD, non-alcoholic fatty liver disease.

Statistical analysis using Poisson regression with robust standard errors; results are expressed as risk ratios and (95% confidence intervals).

Risk ratio (RR) and 95% confidence intervals (CI) were estimated per one standard deviation of MDS (overall association) or of each MDS computed after excluding one component.

* Adjusted for age (years), sex, marital status (single, married/cohabiting, and widowed/Separated/divorced), occupational status (working and not working), education level (university, high school, apprenticeship, and mandatory education), smoking status (never, former, and current), energy intake (kcal/day), total energy expenditure (kcal/day), and date of dietary assessment.

For peer review only

REFERENCES

1 Sattar N, Forrest E, Preiss D. Non-alcoholic fatty liver disease. *BMJ* 2014;**349**. doi:10.1136/bmj.g4596

2 Angulo P. Nonalcoholic fatty liver disease. *N Engl J Med* 2002;**346**:12–9. doi:10.1056/NEJMra011775

3 Williamson RM, Price JF, Glancy S, *et al.* Prevalence of and Risk Factors for Hepatic Steatosis and Nonalcoholic Fatty Liver Disease in People With Type 2 Diabetes: the Edinburgh Type 2 Diabetes Study. *Diabetes Care* 2011;**34**:1139 LP – 1144.http://care.diabetesjournals.org/content/34/5/1139.abstract

4 Bellentani S, Scaglioni F, Marino M, *et al.* Epidemiology of non-alcoholic fatty liver disease. *Dig Dis* 2010;**28**:155–61. doi:10.1159/000282080

5 Angulo P, Keach JC, Batts KP, *et al.* Independent predictors of liver fibrosis in patients with nonalcoholic steatohepatitis. *Hepatology* 1999;**30**:1356–62. doi:10.1002/hep.510300604

6 Marchesini G, Petta S, Grave RD. Diet, Weight Loss, and Liver Health in Nonalcoholic Fatty Liver Disease: Pathophysiology, Evidence, and Practice. *Hepatology* 2016;**63plos**:2032–43. doi:10.1002/hep.28392

7 Marchesini G, Day CP, Dufour JF, *et al.* EASL–EASD–EASO Clinical Practice Guidelines for the management of non-alcoholic fatty liver disease. *J Hepatol* 2016;**64**:1388–402. doi:10.1016/j.jhep.2015.11.004

8 Chalasani N, Younossi Z, Lavine JE, *et al.* The diagnosis and management of non-alcoholic fatty liver disease: practice Guideline by the American Association for the Study of Liver Diseases, American College of Gastroenterology, and the American Gastroenterological Association. *Hepatology* 2012;**55**:2005–23. doi:10.1002/hep.25762

9 Sawangjit R, Chongmelaxme B, Phisalprapa P, *et al.* Comparative efficacy of interventions on nonalcoholic fatty liver disease (NAFLD): A PRISMA-compliant systematic review and network meta-analysis. *Med* 2016;**95**:e4529. doi:10.1097/md.0000000000004529

10 Plauth M, Bernal W, Dasarathy S, *et al.* ESPEN guideline on clinical nutrition in liver disease. *Clin Nutr* 2019;**38**:485–521. doi:https://doi.org/10.1016/j.clnu.2018.12.022

For peer review only

For peer review only - http://bmjopen.bmj.com/site/about/guidelines.xhtml

11 Abenavoli L, Di Renzo L, Boccuto L, *et al.* Health benefits of Mediterranean diet in nonalcoholic fatty liver disease. *Expert Rev Gastroenterol Hepatol* 2018;**12**:873–81. doi:10.1080/17474124.2018.1503947

12 Asbaghi O, Choghakhori R, Ashtary-Larky D, *et al.* Effects of the Mediterranean diet on cardiovascular risk factors in non-alcoholic fatty liver disease patients: A systematic review and meta-analysis. *Clin Nutr ESPEN* 2020;**37**:148–56. doi:10.1016/j.clnesp.2020.03.003

13 Estruch R, Ros E, Salas-Salvadó J, *et al.* Primary Prevention of Cardiovascular Disease with a Mediterranean Diet Supplemented with Extra-Virgin Olive Oil or Nuts. *N Engl J Med* 2018;**378**:e34. doi:10.1056/NEJMoa1800389

14 Sofi F, Cesari F, Abbate R, *et al.* Adherence to Mediterranean diet and health status: meta-analysis. *BMJ* 2008;**337**:a1344. doi:10.1136/bmj.a1344

15 Salas-Salvado J, Bullo M, Babio N, *et al.* Reduction in the incidence of type 2 diabetes with the Mediterranean diet: results of the PREDIMED-Reus nutrition intervention randomized trial. *Diabetes Care* 2011;**34**:14–9. doi:10.2337/dc10-1288

16 Esposito K, Kastorini C-M, Panagiotakos DB, *et al.* Mediterranean diet and metabolic syndrome: an updated systematic review. *Rev Endocr Metab Disord* 2013;**14**:255–63. doi:10.1007/s11154-013-9253-9

17 Ryan MC, Itsiopoulos C, Thodis T, *et al.* The Mediterranean diet improves hepatic steatosis and insulin sensitivity in individuals with non-alcoholic fatty liver disease. *J Hepatol* 2013;**59**:138–43. doi:10.1016/j.jhep.2013.02.012

18 Properzi C, O'Sullivan TA, Sherriff JL, *et al.* Ad Libitum Mediterranean and Low-Fat Diets Both Significantly Reduce Hepatic Steatosis: A Randomized Controlled Trial. *Hepatology* 2018;**68**:1741–54. doi:10.1002/hep.30076

19 Misciagna G, del Pilar Diaz M, Caramia D V, *et al.* Effect of a low glycemic index Mediterranean diet on non-alcoholic fatty liver disease. A randomized controlled clinici trial. *J Nutr Heal AGING* 2017;**21**:404–12. doi:10.1007/s12603-016-0809-8

20 Trovato FM, Catalano D, Martines GF, *et al.* Mediterranean diet and non-alcoholic fatty liver disease: the need of extended and comprehensive interventions. *Clin Nutr* 2015;**34**:86–8.

For peer review only - http://bmjopen.bmj.com/site/about/guidelines.xhtml

doi:10.1016/j.clnu.2014.01.018

21 Gelli C, Tarocchi M, Abenavoli L, *et al.* Effect of a counseling-supported treatment with the Mediterranean diet and physical activity on the severity of the non-alcoholic fatty liver disease. *World J Gastroenterol* 2017;**23**:3150–62. doi:10.3748/wjg.v23.i17.3150

22 Baratta F, Pastori D, Polimeni L, *et al.* Adherence to mediterranean diet and non-alcoholic fatty liver disease: Impact on metabolic profile. *Nutr Metab Cardiovasc Dis* 2017;**27 (1)**:e8.http://ovidsp.ovid.com/athens/ovidweb.cgi?T=JS&CSC=Y&NEWS=N&PAGE=fulltext&D=emex&AN=614265212

23 Abenavoli L, Greco M, Nazionale I, *et al.* Effects of Mediterranean diet supplemented with silybin-vitamin E-phospholipid complex in overweight patients with non-alcoholic fatty liver disease. *Expert Rev Gastroenterol Hepatol* 2015;**9**:519–27. doi:10.1586/17474124.2015.1004312

24 Fraser A, Abel R, Lawlor DA, *et al.* A modified Mediterranean diet is associated with the greatest reduction in alanine aminotransferase levels in obese type 2 diabetes patients: results of a quasi-randomised controlled trial. *Diabetologia* 2008;**51**:1616–22. doi:10.1007/s00125-008-1049-1

25 Kontogianni MD, Tileli N, Margariti A, *et al.* Adherence to the Mediterranean diet is associated with the severity of non-alcoholic fatty liver disease. *Clin Nutr* 2014;**33**:678–83. doi:10.1016/j.clnu.2013.08.014

26 Cantero I, Abete I, Babio N, *et al.* Dietary Inflammatory Index and liver status in subjects with different adiposity levels within the PREDIMED trial. *Clin Nutr* Published Online First: 2017. doi:http://dx.doi.org/10.1016/j.clnu.2017.06.027

27 Khalatbari-Soltani S, Imamura F, Brage S, *et al.* The association between adherence to the Mediterranean diet and hepatic steatosis: cross-sectional analysis of two independent studies, the UK Fenland Study and the Swiss CoLaus Study. *BMC Med* 2019;**17**:19. doi:10.1186/s12916-019-1251-7

28 Ma J, Hennein R, Liu C, *et al.* Improved Diet Quality Associates With Reduction in Liver Fat, Particularly in Individuals With High Genetic Risk Scores for Nonalcoholic Fatty Liver

For peer review only

Disease. *Gastroenterology* 2018;**155**:107–17. doi:10.1053/j.gastro.2018.03.038

29 Firmann M, Mayor V, Vidal PM, *et al.* The CoLaus study: a population-based study to investigate the epidemiology and genetic determinants of cardiovascular risk factors and metabolic syndrome. *BMC Cardiovasc Disord* 2008;**8**:6. doi:10.1186/1471-2261-8-6

30 Morabia A, Bernstein M, Kumanyika S, *et al.* Développement et validation d'un questionnaire alimentaire semi-quantitatif à partir d'une enquête de population. *Soz Praventivmed* 1994;**39**:345–69. doi:10.1007/BF01299666

31 Bernstein L, Huot I MA, Bernstein L, Huot I, *et al.* Amélioration des performances d'un questionnaire alimentaire semi-quantitatif comparé à un rappel des 24 heures. *Sante Publique (Paris)* 1995;**7**:403–13.

32 Bach-Faig A, Berry EM, Lairon D, *et al.* Mediterranean diet pyramid today. Science and cultural updates. *Public Health Nutr* 2011;**14**:2274–84. doi:10.1017/S1368980011002515

33 Tong TYN, Wareham NJ, Khaw K-T, *et al.* Prospective association of the Mediterranean diet with cardiovascular disease incidence and mortality and its population impact in a non-Mediterranean population: the EPIC-Norfolk study. *BMC Med* 2016;**14**:135. doi:10.1186/s12916-016-0677-4

34 Willett WC, Howe GR, Kushi LH. Adjustment for total energy intake in epidemiologic studies. *Am J Clin Nutr* 1997;**65**:1220S-1228S; discussion 1229S-1231S.

35 Bedogni G, Bellentani S, Miglioli L, *et al.* The Fatty Liver Index: a simple and accurate predictor of hepatic steatosis in the general population. *BMC Gastroenterol* 2006;**6**:33. doi:10.1186/1471-230X-6-33

36 Kotronen A, Peltonen M, Hakkarainen A, *et al.* Prediction of Non-Alcoholic Fatty Liver Disease and Liver Fat Using Metabolic and Genetic Factors. *Gastroenterology* 2009;**137**:865–72. doi:http://dx.doi.org/10.1053/j.gastro.2009.06.005

37 Ruhl CE, Everhart JE. Fatty liver indices in the multiethnic United States National Health and Nutrition Examination Survey. *Aliment Pharmacol Ther* 2015;**41**:65–76. doi:10.1111/apt.13012

38 Alberti KGMM, Zimmet P, Shaw J. Metabolic syndrome--a new world-wide definition. A

For peer review only

For peer review only - http://bmjopen.bmj.com/site/about/guidelines.xhtml

Consensus Statement from the International Diabetes Federation. *Diabet Med* 2006;**23**:469–80. doi:10.1111/j.1464-5491.2006.01858.x

39 Bernstein M, Sloutskis D, Kumanyika S, *et al.* Data-based approach for developing a physical activity frequency questionnaire. *Am J Epidemiol* 1998;**147**:147–54.

40 Lee J-H, Kim D, Kim HJ, *et al.* Hepatic steatosis index: a simple screening tool reflecting nonalcoholic fatty liver disease. *Dig liver Dis Off J Ital Soc Gastroenterol Ital Assoc Study Liver* 2010;**42**:503–8. doi:10.1016/j.dld.2009.08.002

41 Stefan N, Häring H-U, Cusi K. Non-alcoholic fatty liver disease: causes, diagnosis, cardiometabolic consequences, and treatment strategies. *lancet Diabetes Endocrinol* 2019;**7**:313–24. doi:10.1016/S2213-8587(18)30154-2

42 Chan R, Wong VW-S, Chu WC-W, *et al.* Diet-Quality Scores and Prevalence of Nonalcoholic Fatty Liver Disease: A Population Study Using Proton-Magnetic Resonance Spectroscopy. *PLoS One* 2015;**10**:1–14. doi:10.1371/journal.pone.0139310

43 Fung TT, Pan AA, Hou T, *et al.* Long-Term Change in Diet Quality Is Associated with Body Weight Change in Men and Women. *J Nutr* 2015;**145**:1850–6. doi:10.3945/jn.114.208785

44 Freedman ND, Cross AJ, McGlynn KA, *et al.* Association of meat and fat intake with liver disease and hepatocellular carcinoma in the NIH-AARP cohort. *J Natl Cancer Inst* 2010;**102**:1354–65. doi:10.1093/jnci/djq301

45 Georgoulis M, Kontogianni MD, Yiannakouris N. Mediterranean Diet and Diabetes: Prevention and Treatment. *Nutrients* 2014;**6**:1406–23. doi:10.3390/nu6041406

46 Georgoulis M, Kontogianni MD, Tileli N, *et al.* The impact of cereal grain consumption on the development and severity of non-alcoholic fatty liver disease. *Eur J Nutr* 2014;**53**:1727–35. doi:10.1007/s00394-014-0679-y

47 Abenavoli L, Milic N, Di Renzo L, *et al.* Metabolic aspects of adult patients with nonalcoholic fatty liver disease. *World J Gastroenterol* 2016;**22**:7006–16. doi:10.3748/wjg.v22.i31.7006

48 Salomone F, Godos J, Zelber-Sagi S. Natural antioxidants for non-alcoholic fatty liver disease: molecular targets and clinical perspectives. *LIVER Int* 2016;**36**:5–20. doi:10.1111/liv.12975

49 Abenavoli L, Milic N, Luzza F, *et al.* Polyphenols Treatment in Patients with Nonalcoholic

For peer review only

Fatty Liver Disease. *J Transl Intern Med* 2017;**5**:144–7. doi:10.1515/jtim-2017-0027

50 Yang J, Fernández-Galilea M, Martínez-Fernández L, *et al.* Oxidative Stress and Non-Alcoholic Fatty Liver Disease: Effects of Omega-3 Fatty Acid Supplementation. *Nutrients* 2019;**11**. doi:10.3390/nu11040872

51 Salomone F, Ivancovsky-Wajcman D, Fliss-Isakov N, *et al.* Higher phenolic acid intake independently associates with lower prevalence of insulin resistance and non-alcoholic fatty liver disease. *JHEP Reports* 2020;**2**:100069. doi:https://doi.org/10.1016/j.jhepr.2020.100069

52 Zelber-Sagi S, Salomone F, Mlynarsky L. The Mediterranean dietary pattern as the diet of choice for non-alcoholic fatty liver disease: Evidence and plausible mechanisms. *Liver Int* 2017;**37**:936–49. doi:10.1111/liv.13435

53 Mahady SE, George J. Exercise and diet in the management of nonalcoholic fatty liver disease. *Metab Exp* 2016;**65**:1172–82. doi:10.1016/j.metabol.2015.10.032

54 Parker HM, Johnson NA, Burdon CA, *et al.* Omega-3 supplementation and non-alcoholic fatty liver disease: A systematic review and meta-analysis. *J Hepatol* 2012;**56**:944–51. doi:10.1016/j.jhep.2011.08.018

55 Hernandez EA, Kahl S, Seelig A, *et al.* Acute dietary fat intake initiates alterations in energy metabolism and insulin resistance. *J Clin Invest* 2017;**127**:695–708.http://ovidsp.ovid.com/athens/ovidweb.cgi?T=JS&CSC=Y&NEWS=N&PAGE=fulltext&D=emex&AN=614623041

56 Bjermo H, Iggman D, Kullberg J, *et al.* Effects of n-6 PUFAs compared with SFAs on liver fat, lipoproteins, and inflammation in abdominal obesity: a randomized controlled trial. *Am J Clin Nutr* 2012;**95**:1003–12. doi:10.3945/ajcn.111.030114

57 Hu FB, Stampfer MJ, Rimm E, *et al.* Dietary fat and coronary heart disease: a comparison of approaches for adjusting for total energy intake and modeling repeated dietary measurements. *Am J Epidemiol* 1999;**149**:531–40.

58 Koehler EM, Schouten JNL, Hansen BE, *et al.* External Validation of the Fatty Liver Index for Identifying Nonalcoholic Fatty Liver Disease in a Population-based Study. *Clin Gastroenterol Hepatol* 2013;**11**:1201–4. doi:10.1016/j.cgh.2012.12.031

For peer review only

59 Machado M V, Cortez-Pinto H. Non-invasive diagnosis of non-alcoholic fatty liver disease. A critical appraisal. *J Hepatol* 2013;**58**:1007–19. doi:10.1016/j.jhep.2012.11.021

60 Zelber-Sagi S, Webb M, Assy N, *et al.* Comparison of fatty liver index with noninvasive methods for steatosis detection and quantification. *World J Gastroenterol* 2013;**19**:57–64. doi:10.3748/wjg.v19.i1.57

61 Fedchuk L, Nascimbeni F, Pais R, *et al.* Performance and limitations of steatosis biomarkers in patients with nonalcoholic fatty liver disease. *Aliment Pharmacol Ther* 2014;**40**:1209–22. doi:10.1111/apt.12963

For peer review only

For peer review only - http://bmjopen.bmj.com/site/about/guidelines.xhtml

Table 1 Baseline characteristics of participants according to quintiles of the Mediterranean diet score, CoLaus study, Switzerland (n=2,288).

	Quintiles of Mediterranean diet score*					
Characteristic	Q1 (n=458)	Q2 (n=458)	Q3 (n=457)	Q4 (n=458)	Q5 (n=457)	*P*-value*
Age (years)	57.1±10.6	56.7±9.9	56.4±10.5	55±9.8	53.7±8.9	<0.001
Women (%)	59.0	62.9	67.6	66.6	71.1	0.001
Marital status (%)						0.51
Single	17.7	14.2	16.8	17.5	16.4	
Married/cohabitant	53.9	55.7	59.1	57.2	57.3	
Widowed/separated/divorced	28.4	30.1	24.1	25.3	26.3	
Employed (%)	57.9	60.3	59.1	68.3	72.0	<0.001
Education (%)						
University	19.2	21.6	26.0	31.7	31.1	
High school	26.4	28.2	28.4	29.7	29.5	
Apprenticeship	39.3	35.2	34.8	28.2	28.4	
Mandatory education	15.1	15.1	10.7	10.5	10.9	<0.001
Current smokers (%)	24.7	21.8	22.3	16.2	15.5	0.014
Alcohol intake (%)†						
Abstainers	21.8	24.0	25.8	24.7	21.2	
Moderate	63.8	66.2	69.4	70.7	76.6	
Heavy	14.4	9.8	4.8	4.6	2.2	<0.001
Total energy intake (kcal/day)	1819±705	1812±675	1781±729	1821±653	1801±595	0.87
Protein (%energy)	15.8±3.3	15.9±4.1	15.3±3.0	15.2±3.0	14.7±2.6	<0.001
Carbohydrates (%energy)	45.3±9.2	45.9±9.1	47.3±8.0	47.6±8.2	49.0±7.8	<0.001
Fat (%energy)	33.6±6.5	34.5±6.9	34.6±6.7	34.5±6.5	34.1±6.9	0.11
TEE (kcal/day)	2562±589	2600±618	2564±602	2558±555	2589±565	0.84
Metabolic syndrome (%)‡	11.8	9.8	12.3	12.7	7.4	0.063

For peer review only - http://bmjopen.bmj.com/site/about/guidelines.xhtml

BMI (kg/m^2)	24.0±2.7	23.8±2.9	23.9±3.0	23.6±2.8	23.3±2.7	0.003
Waist circumference (cm)	85.4±8.5	84.9±9.4	85.0±9.2	84.0±8.7	82.9±8.6	<0.001
Triglycerides (mmol/l)	1.1±0.5	1.0±0.5	1.1±0.5	1.0±0.5	1.0±0.5	
median (iqr)	1.0 (0.8, 1.3)	0.9 (0.7, 1.2)	1.0 (0.7, 1.3)	0.9 (0.7, 1.2)	0.9 (0.7, 1.2)	0.009
GGT (U/l)	25.0±16.5	25.1±17.9	23.2±14.5	24.0±17.9	21.5±12.1	0.002
median (iqr)	20 (15, 29)	21 (15, 28)	19 (14, 28)	19 (14, 26)	18 (14, 25)	
≥50 (%)	7.4	6.3	5.7	6.1	2.6	0.025
ALT (U/l)	21.3±7.8	22.1±8.9	22.5±9.7	21.3±8.7	21.5±8.7	0.12
median (iqr)	19 (16, 25)	20 (16, 26)	20 (16, 27)	19 (16, 25)	20 (16, 24)	
≥40 (%)	3.5	5.4	8.3	4.6	3.5	0.005
AST (U/l)	26.0±5.85	26.2±6.5	26.1±6.6	25.8±6.1	25.7±5.7	0.68
median (iqr)	25 (22, 29)	25 (22, 29)	25 (21, 29)	25 (22, 29)	25 (22, 29)	
≥37 (%)	4.1	8.3	6.3	5.0	4.4	0.038

Abbreviations: TEE, total energy expenditure; BMI, body mass index; iqr, interquartile range; GGT, gamma-glutamyl transferase; ALT, alanine aminotransferase; AST, aspartate aminotransferase.

* The population was divided into five groups by quintiles (Q1-Q5) of the Mediterranean diet score. P-values were computed by using ANOVA for continuous variables and Chi-square test for categorical variables.

† Alcohol consumption categorized as “abstainers” (0 unit/week), “moderate” (1–21 units/week for men, 1–14 for women), and “heavy drinkers” (>21 units/week for men, >14 for women).

‡ Metabolic syndrome defined according to the International Diabetic Federation (waist circumference ≥94 cm in men and ≥80 cm in women plus at least two of the following factors: serum triglycerides≥1.70 mmol/L or specific treatment for this lipid abnormality; serum high-density lipoprotein cholesterol <1.03 mmol/L in men and <1.29 mmol/L in women or specific treatment for this lipid abnormality; systolic blood pressure ≥130 mm Hg or diastolic blood pressure ≥85 mm Hg or treatment for previously diagnosed hypertension; and fasting plasma glucose ≥5.6 mmol/L or previously diagnosed type 2 diabetes).

For peer review only - http://bmjopen.bmj.com/site/about/guidelines.xhtml

Table 2 Prospective association of the Mediterranean diet score with the risk of hepatic steatosis, CoLaus study, Switzerland (n=2,288).

	Risk ratio (95% CI) across quintiles of Mediterranean diet score*					*P*-trend	Risk ratio (95% CI) Per SD increase*
	Q1	Q2	Q3	Q4	Q5		
range	1.83-7.63	7.64-8.35	8.36-8.92	8.93-9.59	9.60-12.1		
N total	458	458	457	458	457		
Fatty liver index, median (iqr)†	21.8 (10.4, 37.7)	20.4 (9.0, 39.4)	18.5 (8.9, 34.6)	18.1 (7.7, 33.1)	14.2 (6.6, 28.5)		
N cases (score≥60)	36	43	35	22	17		
Unadjusted	1.00 (ref.)	1.19 (0.78, 1.82)	0.97 (0.62, 1.52)	0.61 (0.37, 1.02)	0.47 (0.27, 0.83)	0.001	0.79 (0.70, 0.90)
Multivariable ‡	1.00 (ref.)	1.11 (0.72, 1.72)	1.07 (0.68, 1.66)	0.71 (0.42, 1.22)	0.50 (0.28, 0.91)	0.006	0.84 (0.73, 0.96)
Multivariable + BMI	1.00 (ref.)	1.12 (0.73, 1.71)	0.90 (0.58, 1.39)	0.74 (0.44, 1.24)	0.61 (0.34, 1.09)	0.031	0.85 (0.73, 0.99)
Multivariable + BMI + WC	1.00 (ref.)	1.07 (0.70, 1.64)	0.90 (0.59, 1.37)	0.74 (0.44, 1.26)	0.60 (0.34, 1.08)	0.034	0.85 (0.71, 1.02)
NAFLD-score, median (iqr)§	-2.1 (-2.4, -1.5)	-2.0 (-2.4, -1.4)	-2.0 (-2.4, -1.3)	-2.1 (-2.5, -1.5)	-2.1 (-2.5, -1.6)		
N cases (score≥-0.640)	41	46	51	38	32		
Unadjusted	1.00 (ref.)	1.12 (0.75, 1.67)	1.25 (0.84, 1.84)	0.93 (0.61, 1.41)	0.78 (0.50, 1.22)	0.17	0.93 (0.82, 1.05)
Multivariable ‡	1.00 (ref.)	1.13 (0.75, 1.70)	1.26 (0.85, 1.87)	1.01 (0.65, 1.56)	0.80 (0.50, 1.28)	0.28	0.95 (0.83, 1.09)
Multivariable + BMI	1.00 (ref.)	1.17 (0.78, 1.75)	1.17 (0.80, 1.71)	1.07 (0.69, 1.65)	0.95 (0.60, 1.52)	0.71	0.99 (0.86, 1.15)
Multivariable + BMI + WC	1.00 (ref.)	1.12 (0.75, 1.67)	1.17 (0.80, 1.72)	1.07 (0.70, 1.65)	0.96 (0.60, 1.53)	0.80	1.00 (0.86, 1.17)

Abbreviations: iqr, interquartile range; BMI, body mass index; WC, waist circumference; NAFLD, non-alcoholic fatty liver disease.

For peer review only - http://bmjopen.bmj.com/site/about/guidelines.xhtml

Statistical analysis using Poisson regression with robust standard errors; results are expressed as risk ratios and (95% confidence intervals).

* In categorical analysis, the population was divided into five groups by quintiles (Q1-Q5) of the Mediterranean diet score; Standard deviation was 1.20 for the multivariable analyses.

† Calculated based on an algorithm including BMI, waist circumference, triglycerides, and gamma-glutamyl transferase.

‡ Adjusted for age (years), sex, marital status (single, married/cohabiting, and widowed/Separated/divorced), occupational status (working and not working), education level (university, high school, apprenticeship, and mandatory education), smoking status (never, former, and current), energy intake (kcal/day), total energy expenditure (kcal/day), and date of dietary assessment.

§ Calculated based on an algorithm including presence of the metabolic syndrome and type 2 diabetes, and concentrations of fasting serum insulin, fasting serum aspartate-aminotransferase (AST), and the AST/alanine-aminotransferase ratio.

For peer review only

For peer review only - http://bmjopen.bmj.com/site/about/guidelines.xhtml

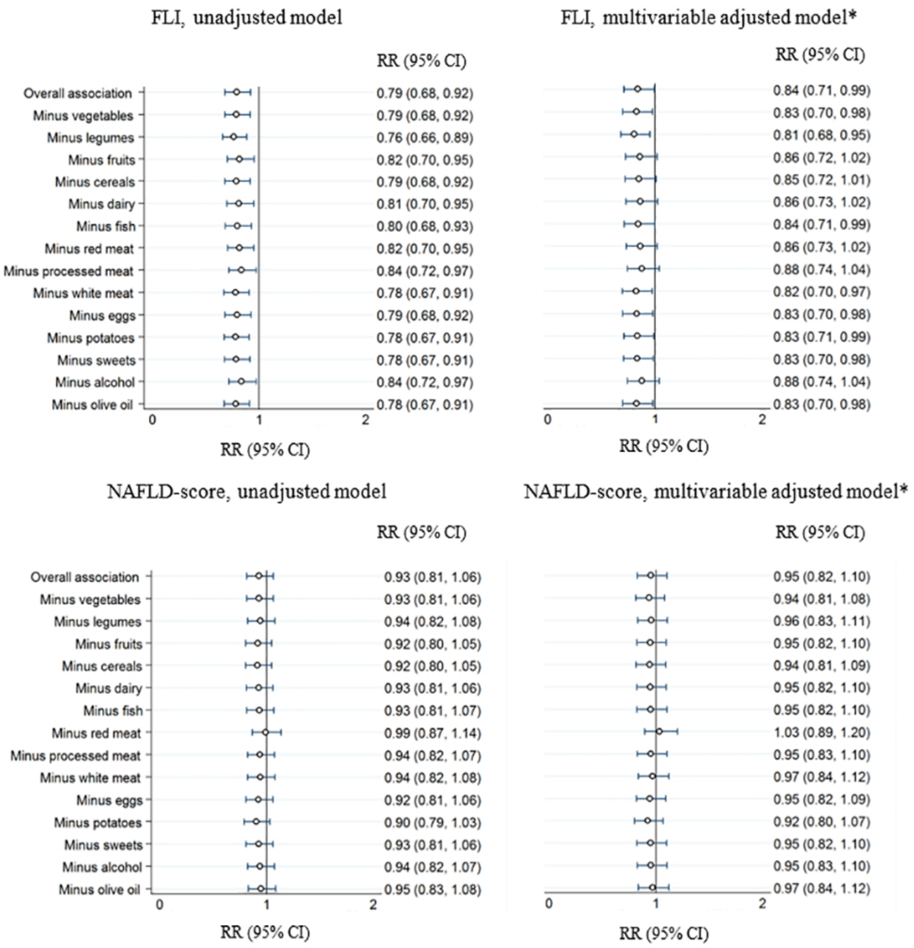

Figure 1 Prospective association of the Mediterranean diet score with the risk of hepatic steatosis, CoLaus study, Switzerland (n=2288): sensitivity analysis to examine influence of each component of Mediterranean diet.

Abbreviations: FLI, fatty liver index; NAFLD, non-alcoholic fatty liver disease.
Statistical analysis using Poisson regression with robust standard errors; results are expressed as risk ratios and (95% confidence intervals).
Risk ratio (RR) and 95% confidence intervals (CI) were estimated per one standard deviation of MDS (overall association) or of each MDS computed after excluding one component.
* Adjusted for age (years), sex, marital status (single, married/cohabiting, and widowed/Separated/divorced), occupational status (working and not working), education level (university, high school, apprenticeship, and mandatory education), smoking status (never, former, and current), energy intake (kcal/day), total energy expenditure (kcal/day), and date of dietary assessment.

Supplementary file

Title: The association between adherence to the Mediterranean diet and hepatic steatosis: the Swiss CoLaus prospective cohort study

For peer review only

Table of Content

Figure S1 Sample selection flow chart. 3

Table S1 Characteristics of the participants included and excluded from the analysis, CoLaus study, Switzerland. 4

Table S2 Baseline characteristics of the participants according to the risk of hepatic steatosis defined by the fatty liver index or the NAFLD-score, CoLaus study, Switzerland. 5

Table S3 Prospective association of the Mediterranean diet score with the risk of hepatic steatosis, CoLaus study, Switzerland: assessment of influence of other potential covariates. 6

Table S4 Sensitivity analyses for the associations of the Mediterranean diet score with the risk of hepatic steatosis, CoLaus study, Switzerland. 8

Table S5 Prospective association of the Mediterranean diet score with the risk of hepatic steatosis, CoLaus study, Switzerland: sensitivity analysis while excluding participants with only one of the indices high at baseline and using hepatic steatosis index as an alternative score. 11

Table S6 Prospective association of the Mediterranean diet score with the risk of hepatic steatosis, CoLaus study, Switzerland (n=1632), Sensitivity analysis while excluding participants with either indices high (using FLI>30 instead of FLI≥60). 13

Table S7 Association of the Mediterranean diet score with change in BMI and waist circumference, CoLaus study, Switzerland. 14

Table S8 Association of the Mediterranean diet score with the GGT, ALT, and AST, CoLaus study, Switzerland. 15

For peer review only

Figure S1 Sample selection flow chart.

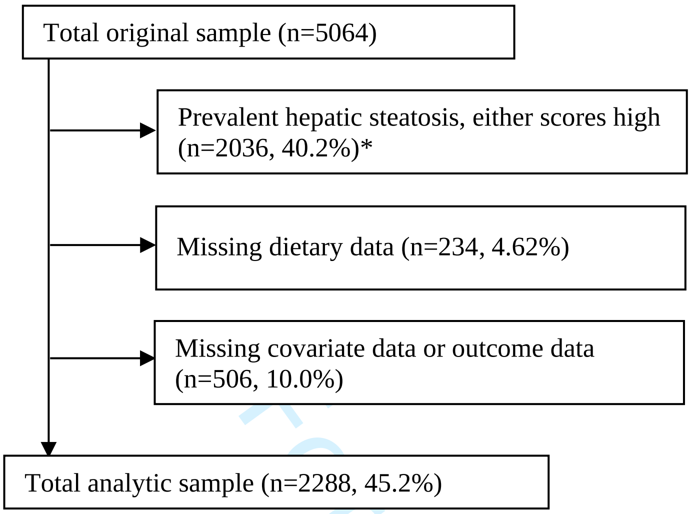

* Prevalent hepatic steatosis was defined as having either fatty liver index≥ 60 OR non-alcoholic fatty liver disease fatty liver score≥ -0.640.

For peer review only

Table S1 Characteristics of the participants included and excluded from the analysis, CoLaus study, Switzerland.

Characteristic	Included (n=2288)	Excluded (n=2776)	*P*-value*
Age, *years*	55.8±10.0	59.4±10.6	<0.001
Women (%)	65.4	43.6	<0.001
Marital status (%)†			0.038
Single	16.5	14.0	
Married/cohabiting	56.6	57.4	
Widowed/separated/divorced	26.8	28.6	
Employed (%)	63.5	50.4	<0.001
Education (%)†			<0.001
University	25.9	17.5	
High school	28.5	23.6	
Apprenticeship	33.2	37.4	
Mandatory education	12.5	21.4	
Current smoker (%)	20.1	23.1	<0.001
Alcohol consumption (%)†‡			<0.001
Abstainers	23.5	26.8	
Moderate	69.3	63.3	
Heavy	7.2	9.9	
Total energy intake (kcal/d)	1807±673	1853±784	0.033
Total protein (% energy)	15.4±3.3	15.6±3.5	0.015
Total carbohydrate (% energy)	47.0±8.6	45.4±9.1	<0.001
Total fat (% energy)	34.3±6.7	34.4±6.9	0.54
TEE (kcal/d)	2575±586	2790±669	<0.001
Metabolic syndrome (%)§	10.8	60.9	<0.001
BMI (kg/m^2)	23.7±2.8	28.3±4.8	<0.001
Waist circumference (cm)	84.4±8.9	98.3±12.5	<0.001
Triglycerides (mmol/l)	1.0±0.5	1.6±1.1	<0.001
median (iqr)	0.9 (0.7, 1.2)	1.4 (1.0, 2.0)	
GGT (U/l)	23.8±16.0	48.8±60.8	<0.001
median (iqr)	19 (14, 27)	32 (21, 53)	
≥50 (%)	5.6	27.5	<0.001
ALT (U/l)	21.8±8.8	32.5±20.7	<0.001
median (iqr)	20 (16, 25)	27 (20, 38)	
≥40 (%)	5.0	23.3	<0.001
AST (U/l)	25.9±6.2	31.6±14.1	<0.001
median (iqr)	25 (22, 29)	28 (24, 34)	
≥37 (%)	5.6	20.4	<0.001

Abbreviations: TEE, total energy expenditure; BMI, body mass index; iqr, interquartile range; GGT, gamma-glutamyl transferase; ALT, alanine aminotransferase; AST, aspartate aminotransferase.

Data are mean ± SD for continuous variables or percent for categorical variables, unless otherwise stated.

* P-value calculated using Chi-square test for categorical variables and student's t-test for continuous variables.

† Due to some missing data, percentages do not always add to 100%.

‡ Alcohol consumption categorized as "abstainers" (0 unit/week), "moderate" (1–21 units/week for men, 1–14 for women), and "heavy drinkers" (>21 units/week for men, >14 for women).

§ Metabolic syndrome defined according to the International Diabetic Federation (waist circumference ≥94 cm in men and ≥80 cm in women plus at least two of the following factors: serum triglycerides≥1.70 mmol/L or specific treatment for this lipid abnormality; serum high-density lipoprotein cholesterol <1.03 mmol/L in men and <1.29 mmol/L in women or specific treatment for this lipid abnormality; systolic blood pressure ≥130 mmHg or diastolic blood pressure ≥85 mmHg or treatment for previously diagnosed hypertension; and fasting plasma glucose ≥5.6 mmol/L or previously diagnosed type 2 diabetes).

For peer review only

Table S2 Baseline characteristics of the participants according to the risk of hepatic steatosis defined by the fatty liver index or the NAFLD-score, CoLaus study, Switzerland.

	Risk of hepatic steatosis					
	FLI			NAFLD-score		
Characteristic	No (n=2135)	Yes (n=153)	*P*-value*	No (n=2080)	Yes (n=208)	*P*-value*
Age, *years*	55.8±10.1	54.9±9.5	0.29	55.6±10	57.7±10.1	0.003
Women (%)	66.8	45.8	<0.001	66.7	52.9	<0.001
Marital status (%)†			0.86			0.79
Single	16.6	15.0		16.7	14.9	
Married/cohabiting	56.5	58.2		56.5	58.2	
Widowed/Separated/divorced	26.8	26.8		26.8	26.9	
Employed (%)	63.3	66.0	0.50	64.5	53.8	0.002
Education (%)†			0.013			0.33
University	26.7	15.0		26.4	20.7	
High school	28.3	30.1		28.3	29.8	
Apprenticeship	32.6	40.5		32.8	36.5	
Mandatory education	12.3	14.4		12.4	13.0	
Current smoker (%)	19.7	25.5	0.006	20.3	17.8	0.52
Alcohol consumption (%)†‡			0.15			0.001
Abstainers	23.3	26.1		22.5	33.7	
Moderate	69.7	63.4		70.4	58.7	
Heavy	6.9	10.5		7.1	7.7	
Total energy intake (kcal/d)	1800±655	1906±879	0.062	1794±654	1938±833	0.003
Protein (% energy)	15.3±3.1	16.1±5.0	0.006	15.3±3.2	16.0±4.1	0.006
Carbohydrate (% energy)	47.1±8.5	45.6±10.1	0.037	47.0±8.5	46.5±9.5	0.36
Fat (% energy)	34.3±6.7	33.8±7.2	0.37	34.2±6.7	34.4±6.6	0.69
TEE (kcal/d)	2552±572	2912±677	<0.001	2562±582	2699±616	0.002
Metabolic syndrome (%)§	9.9	22.9	<0.001	9.8	21.2	<0.001
BMI (kg/m^2)	23.5±2.7	26.8±2.6	<0.001	23.5±2.8	26.0±2.5	<0.001
Waist circumference (cm)	83.8±8.7	93.4±7.0	<0.001	83.7±8.7	92.0±7.4	<0.001
Triglycerides (mmol/l)	1.0±0.5	1.2±0.5	<0.001	1.0±0.5	1.2±0.5	<0.001
median (iqr)	0.9 (0.7, 1.2)	1.1 (0.7, 1.2)		0.9 (0.7, 1.2)	1.1 (0.8, 1.4)	
GGT (U/l)	23.1±15.2	32.4±22.2	<0.001	23.3±15.4	28.1±20.6	<0.001
median (iqr)	19 (14, 26)	26 (18, 40)		19 (14, 26)	22 (15, 34)	
≥50 (%)	4.9	15.0	<0.001	5.3	9.1	0.022
ALT (U/l)	21.6±8.8	23.5±8.9	0.013	21.3±8.4	26.0±11.6	<0.001
median (iqr)	19 (16, 25)	21 (17, 28)		19 (16, 25)	23 (18, 30)	
≥40 (%)	5.0	5.9	0.63	4.5	10.6	<0.001
AST (U/l)	25.9±6.2	26.3±6.3	0.50	25.9±6.2	26.9±6.2	0.026
median (iqr)	25 (22, 29)	25 (22, 29)		25 (22, 29)	26 (22, 30)	
≥37 (%)	5.5	7.2	0.39	5.3	8.2	0.096

Abbreviations: TEE, total energy expenditure; FLI, fatty liver index; NAFLD, non-alcoholic fatty liver disease; BMI, body mass index; iqr, interquartile range; GGT, gamma-glutamyl transferase; ALT, alanine aminotransferase; AST, aspartate Aminotransferase.

Data are mean ± SD for continuous variables or percent for categorical variables, unless otherwise stated.

* P-value calculated using Chi-square test for categorical variables and student's t-test for continuous variables.

† Due to some missing data, percentages do not always add to 100%.

‡ Alcohol consumption categorized as "abstainers" (0 unit/week), "moderate" (1–21 units/week for men, 1–14 for women), and "heavy drinkers" (>21 units/week for men, >14 for women).

§ Metabolic syndrome defined according to the International Diabetic Federation (waist circumference ≥94 cm in men and ≥80 cm in women plus at least two of the following factors: serum triglycerides≥1.70 mmol/L or specific treatment for this lipid abnormality; serum high-density lipoprotein cholesterol <1.03 mmol/L in men and <1.29 mmol/L in women or specific treatment for this lipid abnormality; systolic blood pressure ≥130 mm Hg or diastolic blood pressure ≥85 mm Hg or treatment for previously diagnosed hypertension; and fasting plasma glucose ≥5.6 mmol/L or previously diagnosed type 2 diabetes.

For peer review only - http://bmjopen.bmj.com/site/about/guidelines.xhtml

Table S3 Prospective association of the Mediterranean diet score with the risk of hepatic steatosis, CoLaus study, Switzerland: assessment of influence of other potential covariates.

	Risk ratio (95% CI) across quintiles of Mediterranean diet score*					*P*-trend	Risk ratio (95% CI) Per SD increase*
	Q1	Q2	Q3	Q4	Q5		
Range	1.83-7.63	7.64-8.35	8.36-8.92	8.93-9.59	9.60-12.1		
N total	458	458	457	458	457		
Fatty liver index†							
Different models							
N Cases (score≥60)	36	43	35	22	17		
Multivariable (Model 1)‡	1.00 (ref.)	1.11 (0.72, 1.72)	1.07 (0.68, 1.66)	0.71 (0.42, 1.22)	0.50 (0.28, 0.91)	0.006	0.84 (0.71, 0.99)
Model 1 + Changes in BMI categories§	1.00 (ref.)	1.11 (0.73, 1.69)	0.98 (0.64, 1.50)	0.73 (0.43, 1.24)	0.59 (0.32, 1.06)	0.024	0.85 (0.72, 1.02)
Model 1 + Alcohol	1.00 (ref.)	1.11 (0.72, 1.72)	1.06 (0.69, 1.65)	0.71 (0.41, 1.22)	0.50 (0.28, 0.91)	0.006	0.84 (0.70, 0.99)
Model 1 + Alcohol+ BMI‖	1.00 (ref.)	1.13 (0.74, 1.73)	0.92 (0.59, 1.42)	0.76 (0.45, 1.29)	0.63 (0.35, 1.14)	0.047	0.86 (0.72, 1.04)
Model 1 + Clinical variables**	1.00 (ref.)	1.30 (0.80, 2.11)	1.20 (0.74, 1.94)	0.77 (0.43, 1.39)	0.57 (0.30, 1.09)	0.023	0.84 (0.70, 1.01)
Model 1 + Clinical variables + BMI	1.00 (ref.)	1.28 (0.79, 2.07)	0.94 (0.58, 1.52)	0.80 (0.45, 1.41)	0.68 (0.36, 1.27)	0.076	0.86 (0.71, 1.04)
Model 1 + MetS††	1.00 (ref.)	1.14 (0.74, 1.75)	1.03 (0.66, 1.60)	0.68 (0.40, 1.16)	0.51 (0.28, 0.93)	0.005	0.83 (0.70, 0.99)
NAFLD-score‡‡							
Different models							
N Cases (score≥-0.640)	41	46	51	38	32		
Multivariable (Model 1)‡	1.00 (ref.)	1.13 (0.75, 1.70)	1.26 (0.85, 1.87)	1.01 (0.65, 1.56)	0.80 (0.50, 1.28)	0.28	0.95 (0.82, 1.10)
Model 1 + Changes in BMI categories§	1.00 (ref.)	1.13 (0.76, 1.69)	1.18 (0.80, 1.74)	1.04 (0.68, 1.60)	0.93 (0.58, 1.48)	0.65	0.99 (0.86, 1.16)
Model 1 + Alcohol	1.00 (ref.)	1.11 (0.73, 1.67)	1.22 (0.82, 1.82)	0.97 (0.63, 1.51)	0.77 (0.48, 1.23)	0.21	0.94 (0.81, 1.09)
Model 1 + Alcohol+ BMI‖	1.00 (ref.)	1.16 (0.78, 1.73)	1.15 (0.78, 1.68)	1.04 (0.68, 1.61)	0.93 (0.58, 1.48)	0.62	0.99 (0.85, 1.15)
Model 1 + Clinical variables**	1.00 (ref.)	1.20 (0.78, 1.84)	1.32 (0.88, 1.98)	0.99 (0.64, 1.55)	0.85 (0.52, 1.37)	0.33	0.95 (0.82, 1.11)
Model 1 + Clinical variables + BMI	1.00 (ref.)	1.21 (0.80, 1.84)	1.19 (0.80, 1.78)	1.06 (0.69, 1.64)	0.99 (0.61, 1.60)	0.76	0.99 (0.85, 1.16)

Abbreviations: SES, socio-economic status; BMI, body mass index; MetS, metabolic syndrome; NAFLD, non-alcoholic fatty liver disease; SD, standard deviation; CI, confidence interval.

Statistical analysis using Poisson regression with robust standard errors; results are expressed as risk ratios and (95% confidence intervals).

* In categorical analysis, the population was divided into five groups by quintiles (Q1-Q5) of the Mediterranean diet score; Standard deviation was 1.20 for different multivariable analyses.

† Calculated based on an algorithm including BMI, waist circumference, triglycerides, and gamma-glutamyl transferase.

‡ Adjusted for age (years), sex, marital status (single, married/cohabitant, and widowed/Separated/divorced), occupational status (working and not working), education level (university, high school, apprenticeship, and mandatory education), smoking status (never, former, and current), energy intake (kcal/day), total energy expenditure (kcal/day), and date of dietary assessment.

§ Changes in BMI categories defined as individuals with a normal BMI at baseline and follow-up, individuals with normal BMI at baseline and overweight or obese BMI at follow-up, individuals with overweight or obese BMI at baseline and at follow-up, or individuals with overweight or obese BMI at baseline and normal at follow-up.

‖ Results of further adjustment for waist circumference were broadly in line with of the further adjustment for BMI (data not shown).

**Family history of diabetes (yes/no), high blood pressure (yes/no), high triglyceride level (yes/no), low HDL level (yes/no), and high glucose level (yes/no).

†† Metabolic syndrome as defined by the International Diabetes Federation (yes/no).

For peer review only - http://bmjopen.bmj.com/site/about/guidelines.xhtml

‡‡ Calculated based on an algorithm including presence of the metabolic syndrome and type 2 diabetes, and concentrations of fasting serum insulin, fasting serum aspartate-aminotransferase (AST), and the AST/alanine-aminotransferase ratio.

For peer review only

Table S4 Sensitivity analyses for the associations of the Mediterranean diet score with the risk of hepatic steatosis, CoLaus study, Switzerland.

	Risk ratio (95% CI) across quintiles of Mediterranean diet score*						
	Q1	Q2	Q3	Q4	Q5	*P*-trend	Risk ratio (95% CI) Per SD increase*
Range	1.83-7.63	7.64-8.35	8.36-8.92	8.93-9.59	9.60-12.1		
N total	458	458	457	458	457		
Fatty liver index†							
Different models							
N Cases (score≥60)	36	43	35	22	17		
Multivariable (Model 1)‡	1.00 (ref.)	1.11 (0.72, 1.72)	1.07 (0.68, 1.66)	0.71 (0.42, 1.22)	0.50 (0.28, 0.91)	0.006	0.84 (0.71, 0.99)
Excluding alcohol from MDS component							
Model 1 + Alcohol	1.00 (ref.)	1.64 (1.04, 2.58)	1.08 (0.65, 1.80)	1.17 (0.70, 1.96)	0.65 (0.35, 1.18)	0.069	0.86 (0.73, 1.03)
Model 1 + Alcohol + BMI§	1.00 (ref.)	1.53 (0.98, 2.39)	0.94 (0.57, 1.54)	1.08 (0.67, 1.77)	0.75 (0.41, 1.35)	0.15	0.89 (0.75, 1.06)
Excluding participants with BMI≥30‖							
N Cases (score≥60)	35	40	28	19	16		
Model 1	1.00 (ref.)	1.08 (0.69, 1.68)	0.90 (0.56, 1.46)	0.64 (0.36, 1.12)	0.50 (0.27, 0.93)	0.005	0.83 (0.69, 0.98)
Model 1 + BMI§	1.00 (ref.)	1.02 (0.66, 1.58)	0.84 (0.53, 1.33)	0.65 (0.37, 1.14)	0.60 (0.33, 1.10)	0.031	0.85 (0.70, 1.02)
Excluding participants with excessive alcohol consumption**							
N Cases (score≥60)	33	42	34	21	17		
Model 1	1.00 (ref.)	1.17 (0.75, 1.84)	1.12 (0.71, 1.77)	0.70 (0.40, 1.23)	0.52 (0.28, 0.95)	0.007	0.82 (0.69, 0.98)
Model 1 + BMI§	1.00 (ref.)	1.17 (0.76, 1.81)	0.91 (0.58, 1.42)	0.73 (0.42, 1.24)	0.64 (0.35, 1.15)	0.037	0.85 (0.71, 1.02)
Excluding participants with implausible energy intake††							
N Cases (score≥60)	33	41	33	22	16		
Model 1	1.00 (ref.)	1.19 (0.63, 2.23)	1.16 (0.61, 2.20)	0.87 (0.45, 1.69)	0.69 (0.35, 1.38)	0.16	0.85 (0.71, 1.01)
Model 1 + BMI§	1.00 (ref.)	1.13 (0.60, 2.13)	0.90 (0.47, 1.73)	0.81 (0.43, 1.53)	0.74 (0.37, 1.49)	0.20	0.86 (0.72, 1.04)
Excluding participants with secondary causes of hepatic steatosis‡‡							
N Cases (score≥60)	37	42	35	22	17		
Model 1	1.00 (ref.)	1.18 (0.75, 1.85)	1.11 (0.70, 1.76)	0.78 (0.45, 1.34)	0.51 (0.27, 0.94)	0.010	0.85 (0.71, 1.01)
Model 1 + BMI§	1.00 (ref.)	1.17 (0.75, 1.83)	0.95 (0.60, 1.50)	0.78 (0.46, 1.31)	0.63 (0.34, 1.17)	0.050	0.86 (0.72, 1.04)
Excluding participants with diabetes§§							
N Cases (score≥60)	35	40	35	22	16		
Model 1	1.00 (ref.)	1.06 (0.67, 1.66)	1.11 (0.71, 1.73)	0.74 (0.43, 1.27)	0.49 (0.26, 0.91)	0.009	0.84 (0.71, 0.998)
Model 1 + BMI§	1.00 (ref.)	1.06 (0.69, 1.65)	0.92 (0.59, 1.43)	0.76 (0.45, 1.29)	0.58 (0.32, 1.06)	0.033	0.85 (0.71, 1.02)
NAFLD-score ¶¶							
Different models							
N Cases (score≥-0.640)	41	46	51	38	32		
Multivariable (Model 1)‡	1.00 (ref.)	1.13 (0.75, 1.70)	1.26 (0.85, 1.87)	1.01 (0.65, 1.56)	0.80 (0.50, 1.28)	0.28	0.95 (0.82, 1.10)

For peer review only - http://bmjopen.bmj.com/site/about/guidelines.xhtml

Excluding alcohol from MD component							
Model 1 + Alcohol	1.00 (ref.)	1.15 (0.78, 1.71)	0.92 (0.61, 1.41)	0.92 (0.59, 1.42)	0.89 (0.57, 1.38)	0.34	0.93 (0.81, 1.08)
Model 1 + Alcohol + BMI§	1.00 (ref.)	1.15 (0.78, 1.69)	0.88 (0.59, 1.32)	0.88 (0.57, 1.35)	1.02 (0.66, 1.57)	0.65	0.96 (0.83, 1.12)
Excluding participants with BMI≥30‖							
N Cases (score≥-0.640)	40	46	44	36	31		
Model 1	1.00 (ref.)	1.15 (0.76, 1.74)	1.12 (0.74, 1.69)	0.99 (0.63, 1.55)	0.81 (0.5, 1.30)	0.27	0.94 (0.81, 1.09)
Model 1 + BMI§	1.00 (ref.)	1.17 (0.78, 1.75)	1.09 (0.73, 1.63)	1.07 (0.69, 1.67)	0.97 (0.6, 1.55)	0.76	0.99 (0.85, 1.15)
Excluding participants with excessive alcohol consumption**							
N Cases (score≥-0.640)	38	43	50	37	32		
Model 1	1.00 (ref.)	1.10 (0.73, 1.68)	1.28 (0.85, 1.91)	1.00 (0.64, 1.56)	0.80 (0.5, 1.29)	0.32	0.96 (0.83, 1.11)
Model 1 + BMI§	1.00 (ref.)	1.15 (0.76, 1.74)	1.18 (0.80, 1.73)	1.06 (0.69, 1.65)	0.96 (0.6, 1.54)	0.75	1.00 (0.86, 1.17)
Excluding participants with implausible energy intake††							
N Cases (score≥-0.640)	39	46	48	38	30		
Model 1	1.00 (ref.)	1.32 (0.75, 2.35)	1.48 (0.84, 2.60)	1.06 (0.59, 1.91)	1.15 (0.64, 2.07)	0.92	0.95 (0.82, 1.10)
Model 1 + BMI§	1.00 (ref.)	1.34 (0.76, 2.38)	1.35 (0.77, 2.37)	1.06 (0.59, 1.89)	1.29 (0.72, 2.33)	0.67	0.99 (0.85, 1.16)
Excluding participants with secondary causes of hepatic steatosis‡‡							
N Cases (score≥-0.640)	41	46	50	37	31		
Model 1	1.00 (ref.)	1.19 (0.79, 1.81)	1.26 (0.84, 1.89)	1.06 (0.68, 1.65)	0.78 (0.48, 1.27)	0.25	0.95 (0.82, 1.10)
Model 1 + BMI§	1.00 (ref.)	1.23 (0.82, 1.85)	1.17 (0.79, 1.73)	1.11 (0.71, 1.72)	0.93 (0.57, 1.52)	0.66	0.99 (0.85, 1.16)
Excluding participants with diabetes§§							
N Cases (score≥-0.640)	37	43	50	36	30		
Model 1	1.00 (ref.)	1.17 (0.76, 1.79)	1.37 (0.90, 2.06)	1.05 (0.67, 1.66)	0.85 (0.52, 1.39)	0.43	0.97 (0.83, 1.12)
Model 1 + BMI§	1.00 (ref.)	1.21 (0.79, 1.85)	1.26 (0.85, 1.87)	1.11 (0.71, 1.75)	1.01 (0.62, 1.63)	0.89	1.01 (0.86, 1.18)

Abbreviations: SES, socio economic status; BMI, body mass index; MD, Mediterranean Diet; NAFLD, non-alcoholic fatty liver disease; SD, standard deviation; CI, confidence interval.

Statistical analysis using Poisson regression with robust standard errors; results are expressed as risk ratios and (95% confidence intervals).

* In categorical analysis, the population was divided into five groups by quintiles (Q1-Q5) of the Mediterranean diet score; Standard deviation was 1.41 after excluding alcohol from Mediterranean Diet score components, 1.20 after excluding participants with BMI≥30, 1.18 after excluding participants with excessive alcohol consumption, 1.20 after excluding participants with probable implausible energy intake or after excluding participants with secondary causes of hepatic steatosis or diabetes.

† Calculated based on an algorithm including BMI, waist circumference, triglycerides, and gamma-glutamyl transferase.

‡ Adjusted for age (years), sex, marital status (single, married/cohabitant, and widowed/Separated/divorced), occupational status (working and not working), education level (university, high school, apprenticeship, and mandatory education), smoking status (never, former, and current), energy intake (kcal/day), total energy expenditure (kcal/day), and date of dietary assessment.

§ Results of further adjustment for waist circumference were broadly in line with of the further adjustment for BMI (data not shown).

‖ Excluded 36 participants with BMI≥30 kg/m^2.

** Excessive alcohol consumption defined as >21 units per week for men and >14 units per weeks for women; excluded 60 participants with excess alcohol consumption (n=2228).

†† Implausible energy intake defined as <500 and <800 kcal or >3,500 or >4,000 kcal in women and men, respectively; excluded 41 participants with probable implausible energy intake (n=2247).

For peer review only

‡‡ Secondary causes of hepatic steatosis defined as having hepatitis B, C or HIV, and with hepatotoxic medications (Glucocorticoids, isoniazid, methotrexate, amiodarone, and tamoxifen); excluded 36 participants with probable secondary causes of hepatic steatosis (n=2252).
§§ Diabetes defined as glycated haemoglobin≥48 mmol/mol, or fasting plasma glucose ≥7.0 mmol/L, or use of hypoglycaemic drugs or insulin; excluded 26 participants with probable secondary causes of hepatic steatosis (n=2262).
¶¶ Calculated based on an algorithm including presence of the metabolic syndrome and type 2 diabetes, and concentrations of fasting serum insulin, fasting serum aspartate-aminotransferase (AST), and the AST/alanine-aminotransferase ratio.

For peer review only

For peer review only - http://bmjopen.bmj.com/site/about/guidelines.xhtml

Table S5 Prospective association of the Mediterranean diet score with the risk of hepatic steatosis, CoLaus study, Switzerland: sensitivity analysis while excluding participants with only one of the indices high at baseline and using hepatic steatosis index as an alternative score.

	Risk ratio (95% CI) across quintiles of Mediterranean diet score*					*P*-trend	Risk ratio (95% CI) Per SD increase *
	Q1	Q2	Q3	Q4	Q5		
range	1.83-7.64	7.64-8.34	8.35-8.92	8.93-9.57	9.58-12.18		
N total (n=2652)	531	530	531	530	530		
Fatty liver index, *median* (iqr)†	23.4 (11.1, 41.4)	24.1 (10.6, 44.7)	22.3 (9.9, 39.7)	20.2 (8.4, 38.4)	17.5 (7.3, 34.5)		
N cases (score≥60)	51	56	54	35	24		
Unadjusted	1.00 (ref.)	1.10 (0.77, 1.58)	1.06 (0.74, 1.52)	0.69 (0.45, 1.04)	0.47 (0.29, 0.75)	<0.001	0.79 (0.69, 0.89)
Multivariable ‡	1.00 (ref.)	1.12 (0.77, 1.63)	1.25 (0.86, 1.81)	0.88 (0.57, 1.35)	0.56 (0.34, 0.92)	0.011	0.86 (0.74, 0.99)
Multivariable + BMI	1.00 (ref.)	1.08 (0.75, 1.54)	0.99 (0.70, 1.41)	0.85 (0.56, 1.30)	0.59 (0.36, 0.96)	0.017	0.84 (0.73, 0.98)
Multivariable + BMI & WC	1.00 (ref.)	1.01 (0.71, 1.45)	0.99 (0.70, 1.40)	0.84 (0.56, 1.28)	0.58 (0.35, 0.95)	0.019	0.84 (0.72, 0.98)
range	1.83-7.56	7.57-8.29	8.30-8.88	8.89-9.53	9.54-12.12		
N total (n=2568)	514	514	513	514	513		
NAFLD-score, *median* (iqr)§	-1.9 (-2.4, -1.3)	-1.8 (-2.3, -1.2)	-1.9 (-2.4, -1.3)	-2.0 (-2.5, -1.3)	-2.1 (-2.5, -1.6)		
N cases (score≥-0.640)	63	70	67	59	38		
Unadjusted	1.00 (ref.)	1.11 (0.81, 1.53)	1.07 (0.77, 1.47)	0.94 (0.67, 1.31)	0.60 (0.41, 0.89)	0.006	0.88 (0.79, 0.99)
Multivariable ‡	1.00 (ref.)	1.16 (0.83, 1.62)	1.16 (0.83, 1.61)	1.02 (0.72, 1.46)	0.66 (0.44, 1.00)	0.039	0.92 (0.81, 1.04)
Multivariable + BMI	1.00 (ref.)	1.27 (0.91, 1.76)	1.24 (0.89, 1.72)	1.16 (0.81, 1.65)	0.84 (0.55, 1.28)	0.34	0.98 (0.87, 1.11)
Multivariable + BMI & WC	1.00 (ref.)	1.25 (0.90, 1.73)	1.24 (0.89, 1.72)	1.16 (0.82, 1.65)	0.83 (0.54, 1.27)	0.34	0.99 (0.87, 1.12)
range	1.83-7.15	7.16-8.07	8.08-8.76	8.77-9.47	9.48-12.18		
N total (n=2351)	471	470	470	470	470		
Hepatic steatosis index, *median* (iqr)†	32.4 (30.5, 34.4)	32.3 (30.3, 34.6)	32.5 (30.4, 34.5)	32.6 (30.6, 34.4)	32.4 (30.3, 34.1)		
N cases (score>36)	166	123	120	120	103		
Unadjusted	1.00 (ref.)	0.74 (0.61, 0.90)	0.72 (0.59, 0.88)	0.72 (0.59, 0.88)	0.62 (0.50, 0.76)	<0.001	0.85 (0.79, 0.90)
Multivariable ‡	1.00 (ref.)	0.77 (0.61, 0.96)	0.79 (0.63, 1.00)	0.83 (0.65, 1.04)	0.70 (0.55, 0.91)	0.070	0.90 (0.82, 0.98)

Abbreviations: iqr, interquartile range; SES, socio-economic status; BMI, body mass index; WC, waist circumference; NAFLD, non-alcoholic fatty liver disease.
Statistical analysis using Poisson regression with robust standard errors; results are expressed as risk ratios and (95% confidence intervals).
* In categorical analysis, the population was divided into five groups by quintiles (Q1-Q5) of the Mediterranean diet score; Standard deviation was 1.21 for the multivariable analyses.
† Calculated based on an algorithm including BMI, waist circumference, triglycerides, and gamma-glutamyl transferase.
‡ Adjusted for age (years), sex, marital status (single, married/cohabiting, and widowed/Separated/divorced), occupational status (working and not working), education level (university, high school, apprenticeship, and mandatory education), smoking status (never, former, and current), energy intake (kcal/day), total energy expenditure (kcal/day), and date of dietary assessment.

For peer review only - http://bmjopen.bmj.com/site/about/guidelines.xhtml

§ Calculated based on an algorithm including presence of the metabolic syndrome and type 2 diabetes, and concentrations of fasting serum insulin, fasting serum aspartate-aminotransferase (AST), and the AST/alanine-aminotransferase ratio.

For peer review only

For peer review only - http://bmjopen.bmj.com/site/about/guidelines.xhtml

Table S6 Prospective association of the Mediterranean diet score with the risk of hepatic steatosis, CoLaus study, Switzerland (n=1632), Sensitivity analysis while excluding participants with either indices high (using FLI>30 instead of FLI≥60).

	Risk ratio (95% CI) across quintiles of Mediterranean diet score*					*P*-trend	Risk ratio (95% CI) Per SD increase*
	Q1	Q2	Q3	Q4	Q5		
range	1.83-7.63	7.64-8.35	8.36-8.92	8.93-9.59	9.60-12.1		
N total	327	326	327	326	326		
Fatty liver index, median (iqr)†	14.9 (7.6, 24.8)	12.7 (7.4, 23.7)	12.3 (5.5, 22.3)	11.8 (6.3, 22.7)	10.2 (5.5, 21.8)		
N cases (score≥60)	9	5	2	2	3		
Unadjusted	1.00 (ref.)	0.56 (0.19, 1.65)	0.22 (0.05, 1.02)	0.22 (0.05, 1.02)	0.33 (0.09, 1.22)	0.047	0.66 (0.45, 0.97)
Multivariable ‡	1.00 (ref.)	0.63 (0.20, 2.00)	0.14 (0.02, 0.96)	0.27 (0.05, 1.32)	0.39 (0.09, 1.68)	0.096	0.72 (0.47, 1.10)
Multivariable + BMI	1.00 (ref.)	0.68 (0.22, 2.05)	0.13 (0.02, 0.98)	0.26 (0.06, 1.15)	0.41 (0.10, 1.70)	0.087	0.73 (0.48, 1.11)
Multivariable + BMI + WC	1.00 (ref.)	0.50 (0.18, 1.42)	0.12 (0.01, 0.98)	0.22 (0.05, 1.03)	0.39 (0.09, 1.67)	0.093	0.71 (0.46, 1.09)
NAFLD-score, median (iqr)§	-2.2 (-2.5, -1.8)	-2.2 (-2.5, -1.7)	-2.2 (-2.5, -1.8)	-2.2 (-2.6, -1.8)	-2.2 (-2.6, -1.8)		
N cases (score≥-0.640)	13	14	10	13	12		
Unadjusted	1.00 (ref.)	1.08 (0.52, 2.26)	0.77 (0.34, 1.73)	1.00 (0.47, 2.13)	0.93 (0.43, 2.00)	0.79	0.94 (0.73, 1.20)
Multivariable ‡	1.00 (ref.)	1.13 (0.55, 2.32)	0.79 (0.35, 1.76)	1.10 (0.52, 2.34)	0.98 (0.45, 2.15)	0.94	0.95 (0.73, 1.24)
Multivariable + BMI	1.00 (ref.)	1.25 (0.60, 2.60)	0.81 (0.36, 1.84)	1.20 (0.56, 2.58)	1.13 (0.51, 2.52)	0.82	0.98 (0.75, 1.28)
Multivariable + BMI + WC	1.00 (ref.)	1.17 (0.57, 2.44)	0.85 (0.37, 1.93)	1.13 (0.52, 2.45)	1.12 (0.50, 2.53)	0.84	0.98 (0.75, 1.28)

Abbreviations: iqr, interquartile range; BMI, body mass index; WC, waist circumference; NAFLD, non-alcoholic fatty liver disease.
Statistical analysis using Poisson regression with robust standard errors; results are expressed as risk ratios and (95% confidence intervals).
* In categorical analysis, the population was divided into five groups by quintiles (Q1-Q5) of the Mediterranean diet score; Standard deviation was 1.21 for the multivariable analyses.
† Calculated based on an algorithm including BMI, waist circumference, triglycerides, and gamma-glutamyl transferase.
‡ Adjusted for age (years), sex, marital status (single, married/cohabiting, and widowed/Separated/divorced), occupational status (working and not working), education level (university, high school, apprenticeship, and mandatory education), smoking status (never, former, and current), energy intake (kcal/day), total energy expenditure (kcal/day), and date of dietary assessment.
§ Calculated based on an algorithm including presence of the metabolic syndrome and type 2 diabetes, and concentrations of fasting serum insulin, fasting serum aspartate-aminotransferase (AST), and the AST/alanine-aminotransferase ratio.

For peer review only - http://bmjopen.bmj.com/site/about/guidelines.xhtml

Table S7 Association of the Mediterranean diet score with change in BMI and waist circumference, CoLaus study, Switzerland.

	β coefficient (95% CI) across quintiles of Mediterranean diet score*					*P*-trend	β coefficient (95% CI) Per SD increase*
	Q1	Q2	Q3	Q4	Q5		
range	1.83-7.63	7.64-8.35	8.36-8.92	8.93-9.59	9.60-12.1		
N total	458	458	457	458	457		
ΔBMI, mean±SD†	0.48 ± 1.62	0.61 ± 1.53	0.42 ± 1.44	0.50 ± 1.52	0.40 ± 1.52		
Multivariable ‡	1.00 (ref.)	0.12 (-0.08, 0.33)	-0.08 (-0.28, 0.13)	-0.04 (-0.25, 0.16)	-0.16 (-0.37, 0.04)	0.038	-0.08 (-0.15, -0.02)
ΔWaist circumference, mean±SD§	1.04 ± 6.53	0.74 ± 6.42	0.19 ± 6.00	0.57 ± 6.33	0.17 ± 6.19		
Multivariable ‡	1.00 (ref.)	-0.51 (-1.36, 0.34)	-0.85 (-1.69, 0.00)	-0.47 (-1.32, 0.38)	-0.82 (-1.68, 0.03)	0.10	-0.33 (-0.61, -0.06)

Abbreviations: BMI, Body mass index.
Statistical analysis using linear regression; results are expressed as β coefficients and (95% confidence intervals).
* In categorical analysis, the population was divided into five groups by quintiles (Q1-Q5) of the Mediterranean diet score; Standard deviation was 1.20 for the multivariable analyses.
† Calculated by subtracting BMI at baseline from BMI at follow-up.
‡ Adjusted for age (years), sex, marital status (single, married/cohabitant, and widowed/Separated/divorced), occupational status (working and not working), education level (university, high school, apprenticeship, and mandatory education), smoking status (never, former, and current), energy intake (kcal/day), total energy expenditure (kcal/day), and date of dietary assessment.
§ Calculated by subtracting waist circumference at baseline from waist circumference at follow-up.

For peer review only

Table S8 Association of the Mediterranean diet score with the GGT, ALT, and AST, CoLaus study, Switzerland.

	β coefficient (95% CI) across quintiles of Mediterranean diet score*					*P*-trend	β coefficient (95% CI) Per SD increase *
	Q1	Q2	Q3	Q4	Q5		
range	1.83-7.63	7.64-8.35	8.36-8.92	8.93-9.59	9.60-12.12		
N total	458	458	457	458	457		
GGT (U/l), *median* (iqr)	21 (15, 29)	20 (14, 29)	19 (14, 26)	18 (14, 28)	17 (13, 26)		
Multivariable †	1.00 (ref.)	-2.63 (-9.01, 3.76)	-5.71 (-12.07, 0.66)	-4.24 (-10.64, 2.16)	-6.03 (-12.45, 0.38)	0.063	-1.53 (-3.60, 0.53)
Multivariable + BMI	1.00 (ref.)	-2.86 (-9.24, 3.53)	-5.70 (-12.06, 0.66)	-4.50 (-10.90, 1.90)	-6.52 (-12.95, -0.09)	0.047	-1.66 (-3.73, 0.41)
Multivariable + BMI & WC	1.00 (ref.)	-2.93 (-9.32, 3.45)	-5.72 (-12.08, 0.64)	-4.54 (-10.94, 1.86)	-6.53 (-12.96, -0.10)	0.047	-1.65 (-3.72, 0.41)
ALT (U/l), *median* (iqr)	20 (16, 26)	20.5 (17, 26)	20 (16, 26)	19.5 (16, 25)	20 (16, 25)		
Multivariable †	1.00 (ref.)	0.03 (-0.02, 0.08)	0.03 (-0.02, 0.08)	-0.013 (-0.06, 0.04)	0.002 (-0.05, 0.05)	0.45	-0.006 (-0.02, 0.01)
Multivariable + BMI	1.00 (ref.)	0.04 (-0.01, 0.08)	0.03 (-0.02, 0.08)	-0.009 (-0.06, 0.04)	0.008 (-0.04, 0.06)	0.60	-0.005 (-0.02, 0.01)
Multivariable + BMI & WC	1.00 (ref.)	0.04 (-0.01, 0.08)	0.03 (-0.02, 0.08)	-0.01 (-0.06, 0.04)	0.008 (-0.04, 0.06)	0.60	-0.005 (-0.02, 0.01)
AST (U/l), *median* (iqr)	22 (19, 25)	22 (19, 26)	22 (19, 25)	22 (19, 26)	22 (19, 25)		
Multivariable †	1.00 (ref.)	0.02 (-0.01, 0.06)	0.004 (-0.03, 0.04)	0.011 (-0.03, 0.05)	0.015 (-0.02, 0.05)	0.66	0.003 (-0.01, 0.01)
Multivariable + BMI	1.00 (ref.)	0.02 (-0.02, 0.06)	0.004 (-0.03, 0.04)	0.009 (-0.03, 0.04)	0.011 (-0.03, 0.05)	0.80	0.002 (-0.01, 0.01)
Multivariable + BMI & WC	1.00 (ref.)	0.02 (-0.02, 0.06)	0.004 (-0.03, 0.04)	0.009 (-0.03, 0.04)	0.011 (-0.03, 0.05)	0.80	0.002 (-0.01, 0.01)

Abbreviations: iqr, interquartile range; GGT, gamma-glutamyl transferase; SES, socio economic status; BMI, body mass index; WC, waist circumference; ALT, Alanine transaminase; AST, aspartate-aminotransferase.

Statistical analysis using linear regression; results are expressed as β coefficients and (95% confidence intervals).

* In categorical analysis, the population was divided into five groups by quintiles (Q1-Q5) of the Mediterranean diet score; Standard deviation was 1.20 for the multivariable analyses.

† Adjusted for age (years), sex, marital status (single, married/cohabitant, and widowed/Separated/divorced), occupational status (working and not working), education level (university, high school, apprenticeship, and mandatory education), smoking status (never, former, and current), energy intake (kcal/day), total energy expenditure (kcal/day), and date of dietary assessment.

For peer review only - http://bmjopen.bmj.com/site/about/guidelines.xhtml

STROBE 2007 (v4) Statement—Checklist of items that should be included in reports of *cohort studies*

Section/Topic	Item #	Recommendation	Reported on page #
**Title and abstract**	1	(*a*) Indicate the study's design with a commonly used term in the title or the abstract	3
		(*b*) Provide in the abstract an informative and balanced summary of what was done and what was found	3
**Introduction**			
Background/rationale	2	Explain the scientific background and rationale for the investigation being reported	6
Objectives	3	State specific objectives, including any prespecified hypotheses	7
**Methods**			
Study design	4	Present key elements of study design early in the paper	7
Setting	5	Describe the setting, locations, and relevant dates, including periods of recruitment, exposure, follow-up, and data collection	7
Participants	6	(*a*) Give the eligibility criteria, and the sources and methods of selection of participants. Describe methods of follow-up	7; Figure S1, and table S1
		(*b*) For matched studies, give matching criteria and number of exposed and unexposed	N/A
Variables	7	Clearly define all outcomes, exposures, predictors, potential confounders, and effect modifiers. Give diagnostic criteria, if applicable	Pages 7 to 10
Data sources/ measurement	8*	For each variable of interest, give sources of data and details of methods of assessment (measurement). Describe comparability of assessment methods if there is more than one group	Pages 7 to 10
Bias	9	Describe any efforts to address potential sources of bias	Pages 10 & 11
Study size	10	Explain how the study size was arrived at	12; Figure S1
Quantitative variables	11	Explain how quantitative variables were handled in the analyses. If applicable, describe which groupings were chosen and why	Pages 7 to 10
Statistical methods	12	(*a*) Describe all statistical methods, including those used to control for confounding	Pages 10 & 11
		(*b*) Describe any methods used to examine subgroups and interactions	Pages 10 & 11
		(*c*) Explain how missing data were addressed	Pages 10 & 11
		(*d*) If applicable, explain how loss to follow-up was addressed	Pages 10 & 11
		(*e*) Describe any sensitivity analyses	Pages 10 & 11

For peer review only

**Results**			
Participants	13*	(a) Report numbers of individuals at each stage of study—eg numbers potentially eligible, examined for eligibility, confirmed eligible, included in the study, completing follow-up, and analysed	12
		(b) Give reasons for non-participation at each stage	N/A
		(c) Consider use of a flow diagram	Figure 1
Descriptive data	14*	(a) Give characteristics of study participants (eg demographic, clinical, social) and information on exposures and potential confounders	12; Table 1 and table S1 & S2
		(b) Indicate number of participants with missing data for each variable of interest	12
		(c) Summarise follow-up time (eg, average and total amount)	12
Outcome data	15*	Report numbers of outcome events or summary measures over time	12; Table S2
Main results	16	(*a*) Give unadjusted estimates and, if applicable, confounder-adjusted estimates and their precision (eg, 95% confidence interval). Make clear which confounders were adjusted for and why they were included	Pages 12 & 13; Table 2, Figure 1, and supplementary Tables and Figures
		(*b*) Report category boundaries when continuous variables were categorized	Pages 12 & 13; Table 2, Figure 1, and supplementary Tables and Figures
		(*c*) If relevant, consider translating estimates of relative risk into absolute risk for a meaningful time period	
Other analyses	17	Report other analyses done—eg analyses of subgroups and interactions, and sensitivity analyses	Pages 13 & 14; Supplementary Tables S3 to S8
**Discussion**			
Key results	18	Summarise key results with reference to study objectives	14
**Limitations**	19	Discuss limitations of the study, taking into account sources of potential bias or imprecision.	pages 17 & 18
Interpretation	20	Give a cautious overall interpretation of results considering objectives, limitations, multiplicity of analyses, results from similar studies, and other relevant evidence	Pages 14 to 18
Generalisability	21	Discuss the generalisability (external validity) of the study results	pages 17 & 18
**Other information**			
Funding	22	Give the source of funding and the role of the funders for the present study and, if applicable, for the original study on	2

For peer review only

		which the present article is based	

*Give information separately for cases and controls in case-control studies and, if applicable, for exposed and unexposed groups in cohort and cross-sectional studies.

Note: An Explanation and Elaboration article discusses each checklist item and gives methodological background and published examples of transparent reporting. The STROBE checklist is best used in conjunction with this article (freely available on the Web sites of PLoS Medicine at http://www.plosmedicine.org/, Annals of Internal Medicine at http://www.annals.org/, and Epidemiology at http://www.epidem.com/). Information on the STROBE Initiative is available at www.strobe-statement.org.

For peer review only
